# Potential Similarities in Sex Difference in Key Genes and Their Expression, Network, EQTL and Pathways between COVID-19 and Chronic Kidney Disease Based on Mouse Model

**DOI:** 10.3390/jpm12071190

**Published:** 2022-07-21

**Authors:** Zhuo Yu, Jingyu Zhan, Wei Dong, Lu Lu, Monica M. Jablonski, Lotfi Aleya, Jingyu Chen, Peiqing Zhang, Hong Chen, Weikuan Gu

**Affiliations:** 1 Heilongjiang Academy of Traditional Chinese Medicine, Sanfu Road 142, Xiangfang District, Harbin 150040, China; yuzhuo0722@163.com (Z.Y.); zhangpeiqing@163.com (P.Z.); 2 Department of Gastroenterology, Heilongjiang Academy of Traditional Chinese Medicine, Harbin 150001, China; Zhanjingyu1234@163.com; 3 Department of Orthopedic Surgery and BME-Campbell Clinic, University of Tennessee Health Science Center, Memphis, TN 38163, USA; wdong6@uthsc.edu; 4 Department of Gynecological Radiotherapy, Harbin Medical University Cancer Hospital, Harbin 150081, China; 5 Department of Genetics, Genomics and Informatics, University of Tennessee Health Science Center, Memphis, TN 38163, USA; llu@uthsc.edu; 6 Department of Ophthalmology, University of Tennessee Health Science Center, Memphis, TN 38163, USA; mjablon1@uthsc.edu; 7 Chrono-Environnement Laboratory, UMR CNRS 6249, Bourgogne Franche-Comté University, Université de Franche-Comté 16, Route de Gray, F-25030 Besançon, France; lotfi.aleya@univ-fcomte.fr; 8 Department of Chinese Medicine, First Clinical School of Medicine, Heilongjiang University of Chinese Medicine, Harbin 150040, China; m18804526981@163.com; 9 Research Service, Memphis VA Medical Center, 1030 Jefferson Avenue, Memphis, TN 38104, USA

**Keywords:** chronic kidney disease, COVID-19, sex difference, gene, eQT, pathway

## Abstract

COVID-19 and chronic kidney disease (CKD) share similarity in sex bias and key genes in the disease pathway of sex difference. We investigated the sex difference of molecular pathways of four key players of these two diseases using an existing large set of whole genome expression profiles from the kidneys of female and male mouse models. Our data show that there is little to no correlation at the whole genome expression level between female and male mice among these four genes. There are considerable sex differences among genes in upstream regulation, *Ace2* complex interaction, and downstream pathways. Snap25 and Plcb4 may play important roles in the regulation of the expression level of *Adam17*, *Tmprss2*, and *Cd146* in females. In males, Adh4 is a candidate gene for the regulation of *Adam17*, while Asl, Auts2, and Rabger1 are candidates for *Tmprss2*. Within the *Ace2* complex, *Cd146* directly influences the expression level of *Adam17* and *Ace2* in the female, while in the male Adam potentially has a stronger influence on *Ace2* than that of *Tmprss2*. Among the top 100 most related genes, only one or two genes from four key genes and 11 from the control B-Actin were found to be the same between sexes. Among the top 10 sets of genes in the downstream pathway of *Ace2*, only two sets are the same between the sexes. We concluded that these known key genes and novel genes in CKD may play significant roles in the sex difference in the CKD and COVID-19 disease pathways.

## 1. Introduction

Studies on important genes in the pathological pathways of COVID-19 have shown a surprising coincidence with the important genes in chronic kidney disease (CKD) [1,2,3,4,5,6,7]. In particular, several key genes have been reported from pathological pathway of both diseases, such as angiotensin-converting enzyme 2 (ACE2), A disintegrin and metalloproteinase domain 17 (ADAM17), transmembrane serine protease 2 (TMPRSS2), and melanoma adhesion molecule (CD146/MCAM). In addition, mortality caused by COVID-19 has a sex difference, with a worse rate in men [8,9]. Evidently, it has been known that sex difference exists in CKD. For example, the proportion of women with predialysis CKD is higher than that of men; kidney function declines faster in men than women, and mortality is higher among men at all levels of predialysis CKD, in particular among old men [1,2,3]. While lifestyle and habits influence the sex difference of CKD, biological and genetic differences between men and women also play an important role. A crucial question is whether the sex difference in COVID-19 is related to the major genes in CKD pathways.

Considerable studies have been focused on the potential molecular mechanisms of sex difference of CKD. Direct actions of sex hormones on cellular metabolism have been implicated as the major influence on the sex difference of CKD [4]. Several genes, such as ACE2, has been extensively investigated [5,6]. ACE2 is known to express in various human organs, and its organ- and cell-specific expression suggests that it may play a role in the regulation of renal function. Most importantly, it has been known as critical protective gene for COVID-19 [7]. Monteil and collaborators showed that SARS-CoV-2 can directly infect engineered human blood vessel organoids and human kidney organoids, which can be inhibited by hrsACE2 [8,10]. Evidently, COVID-19 has a more devastating impact on the health of older adults, and especially older men are at risk of death or acutely severe disease [11].

ACE2 can be shed by two proteases, ADAM17 and TMPRSS2. TMPRSS2-cleaved ACE2 allows SARS-CoV-2 cell entry, whereas ADAM17-cleaved ACE2 offers protection to organs [12]. ADAM17 is highly expressed in distal renal tubules and increased in the whole kidney in diabetic models [13]. The ACE2/ADAM17/TMPRSS2 interplay has been suggested as the main risk factor for COVID-19 [14,15,16,17]. The diet- and sex-dependent modulation of ACE2 and TMPRSS2 expression in the lower respiratory tract and esophagus has been reported [18,19]. However, the sex differential expression and pathways in kidney has not been investigated.

Furthermore, an important gene, CD146, has been found critical to CKD failure [20,21]. Interestingly, a study found that both mild and severe COVID-19 patients had significantly less apoptotic CD146 + circulating endothelial cells (CECs) compared with healthy controls. Recovered COVID-19 patients had significantly less CD146 + CECs per milliliter in comparison with heathy controls as well as to mild and severe COVID-19 patients [22]. There are currently no reports on the functional roles of ACE2/ADAM17/TMPRSS2 and CD146 in sex differences of disease, neither on CDK nor on COVID-19. Other factors, such as hypoxia inducible factor 1 (HIF-1) [23,24], heme oxygenase (HO) [25], transforming growth factor-beta 1 (TGF-β1) [26], nuclear factor kappa B (NF-κB) [27] have also been reported to play important roles on COVID-19.

We hypothesize that interplay of ACE2/ADAM17/TMPRSS2 and CD146 in the kidney may lead to sex differences in CKD as well as in COVID-19 by their sex differential expression and pathways. To explore whether there is such a possibility, a population of female and male whole genome expression profiles of the kidney is essential to compare the expression levels of these genes at population level and to construct potential different pathways in the female and male population. Currently, no population in humans is big enough for such an analysis.

Animal models have been used to illustrate the molecular mechanisms of human diseases. The BXD strains of mouse model have become one of the preeminent genetic reference populations to understand the genetic variations and pathways of variety of diseases and phenotypes including sex difference [28,29,30]. Particularly regarding CKD, whole genome expression profiles from female and male of more than 70 BXD strains are currently available at the GeneNetwork (http://www.genenetwork.org (accessed between 4 December 2020–6 March 2021)). This study takes advantage of the whole genome expression profiles of kidney of large number of female and male mouse strains to explore potential connections between sex differential expressions and important genes in CKD and COVID-19.

## 2. Materials and Methods

### 2.1. Data Sets

Two sets of data of mice from GeneNetwork were used for this analysis: Mouse kidney M430v2 Male (Aug06) RMA and Mouse kidney M430v2 Female (Aug06) RMA. Both sets of data were proceeded with the Affymetrix Mouse Genome 430 2.0 array which consists of 992, 936 useful 25-nucleotide probes represent approximately 39,000 transcripts and the majority of known genes and expressed sequence tag. The female set includes two parental strains, C57BL/6J and DBA/2J, one F1, 49 BXD recombinant inbred (RI) strains, and 8 other strains. The male set includes two parental strains, one F1, 35 BXD RI strains and the same 8 other strains as that of the female.

### 2.2. Probe Locations and Numbers of Transcripts of Genes in Mouse Genome

As multiple probes exist among three genes, *Ace2*, *Adam17*, and *Tmprss2*, except *Cd146* in our study, the structure of these genes in mouse genome was examined to evaluate potential role of each probe in the detection of expression levels of the gene. Data of Mouse genome (GRCm39) at Ensembl genome browser 104 (https://useast.ensembl.org/index.html) were used to search each gene to obtain information on splice variants and transcripts. The information was used to assess the location of probes in transcripts and numbers of transcripts of a probe is from.

### 2.3. Comparison of Expression Levels of Four Genes between Female and Male Mice

Expression levels of each probe of four genes, *Ace2*, *Adam17*, *Tmprss2* and *Cd146*, from female and male mice is compared separately. Comparisons are on both the expression patterns and average levels of each probe. Because the expression levels of these probes in comparison are from the same set of study with the same process platform, no adjustment was made before comparison.

### 2.4. eQTL Mapping

Transcriptomic expressions QTL (eQTL), which regulate the expression level of each of the four genes, are conducted using interval mapping platform at the GeneNetwork website. The procedure follows our previous publication and contains three major steps [28]. First, all possible probes of each of these four genes were identified from the kidney genome sets. Second, transcriptome maps for each probe were built using interval mapping tool. The 2000 Bootstrap and 1000 permutation tests were used to ensure the strength and consistency of the mapping. In GeneNetwork, the permutation test is used to estimate suggestive and significant linkage scores while the Bootstrap Test is used for the precision of the QTL location. Outlier data identified by the GeneNetwork system were excluded from the procedure of linkage analysis. At last step, genomic regions and locations on chromosomes of the eQTL were compared among the four genes and between the female and the male.

### 2.5. Matrix Comparison and Pathway Analysis among Probes

For probes of the same genes from female and male, a comparison matrix was used to obtain information of their correlation using the Metrix function of GeneNetwork [29]. The expression data of probes of *Ace2*/*Adam17*/*Tmprss2*, and *Cd146* from female and male kidney were acquired by searching gene names. These expression data were converted into an Excel spreadsheet and the data of correlations among these genes were obtained using our previous method [30]. Correlations then were analyzed to categorize the positive and negative associations between female and male among these genes. The network graph was produced from probes of each gene. The network graph was used to demonstrate the most likely association, either positive or negative, among the probes of the same genes between sexes and among different genes.

### 2.6. Expanded Matrix Comparison and Pathway Analysis

After comparing the four major genes, other known relative genes, such as HIF-1, HO, TGF-β1, and NF-κB, were incorporated into the matrix for the construction of an expanded matrix. The probes of these additional genes and the four key genes themselves were next used for matrix and pathway analysis, performed in the same manner as for the initial analysis on these genes. The matrices and network graph were assessed for genes that are significantly correlated with any of the probes.

### 2.7. Selection of Potential Targets of COVID-19 Disease

Using one probe of known sequence from *Ace2*/*Adam17*/*Tmprss2*, and *Cd146*, further comparison was conducted with the expression levels of the top 100 corrected genetic elements to each of these four genes. For each probe, the top 100 elements of Spearman’s Sample Correlation, rho, whose expression levels mostly correlated with that of the expression level of each probe were identified with spearman rank method. Correlative matrix with all other probes was built using Gene Network.

## 3. Results

### 3.1. Gene Structure and Probes of Ace2, Adam17, Tmprss2 and Cd146 in Mouse Genome 

The complexity between multiple probes and multiple transcripts of genes is the key issue in our analysis. Because different probes are from different parts of the gene, each probe may represent a unique set of transcripts of a gene. Accordingly, we first examined the probes and transcripts of each gene (Supplementary Table S1). A total of 12 probes were included in the study for the expression levels of these four genes in kidney.

Three transcripts are produced from *Ace2* gene: *Ace2*-202. *Ace2*-201, and *Ace2*-203. The lengths of three transcripts are 3578, 3422, and 1714 bps, with 19, 18, and 12 exons, respectively. Three probes of *Ace2* from GeneNetwork were identified and used in the genome expression profiling. They are 1,452,138_a_at, detecting the expression of last 2~6 and 11 exons of *Ace2*; 1,425,102_a_at; for 3’-UTR and last four exons; and 1,425,103_a_at, for middle to distal 3’-UTR. The transcript *Ace2*-203 does not include the 3′-UTR. Thus, probe 1,425,103 may be able to detect the expression levels of two transcripts, *Ace2*-202 and *Ace2*-201.

Ten transcripts are produced from *Adam17* gene: from *Adam17*-201 to *Adam17*-210. The length of these transcripts varies greatly, from as short as 375 to as long as 4469 bps. Four probes were identified from the GeneNetwork for both sexes. These 4 probes are 1,421,857 (last 4 exons), 1,421,858 (mid distal 3’ UTR), 1,421,859 (proximal 3-UTR), and 1,445,500 (intron). As seven of these transcripts contain sequences of 3’ UTR, probes 1,421,858 and 1,421,859 represent these transcripts, including the three transcripts which have functional protein sequences, while 1,445,500 may represent most of the probes. 

Six transcripts are produced by *Tmprss2* gene: from *Tmprss2*-201 to *Tmprss2*-206. Two of them, *Tmprss2*-201 and *Tmprss2*-205 have functional protein sequences. Four probes were found from GeneNetwork. They are 1,458,347 (distal 3′ UTR), 1,419,154 (intron), 1,459,510 (intron), and 1,449,369 (exons 10, 11, and 12). Hence, 1,458,347, 1,419,154, 1,459,510 may represent the overall expression levels of *Tmprss2*.

Six transcripts are produced by *Cd146* (Mcam) gene: from Mcam-201 to Mcam-206. Four of them, Mcam-201, 202, 206 and 204 have protein translations. However, protein sequences in only Mcam-201 and 202 are complete sequences with function and only these two transcripts have the complete 3′ UTR sequences. Only one probe, 1,416,357 (mid 3′ UTR), exists in the GeneNetwork. Therefore, the probe is the representative of the expression of two transcripts from *Cd146*, Mcam-201, and 202.

### 3.2. Expression Levels of Ace2, Adam17, Tmprss2 and Cd146 in the Female and Male Mouse Strains

For the comparison of expression levels of probes, we paired each probe between female and male mice. Because their sequences and locations in each pair are the same, there is no complexity between the nature and location of probe and components of transcripts.

**Figure 1:**
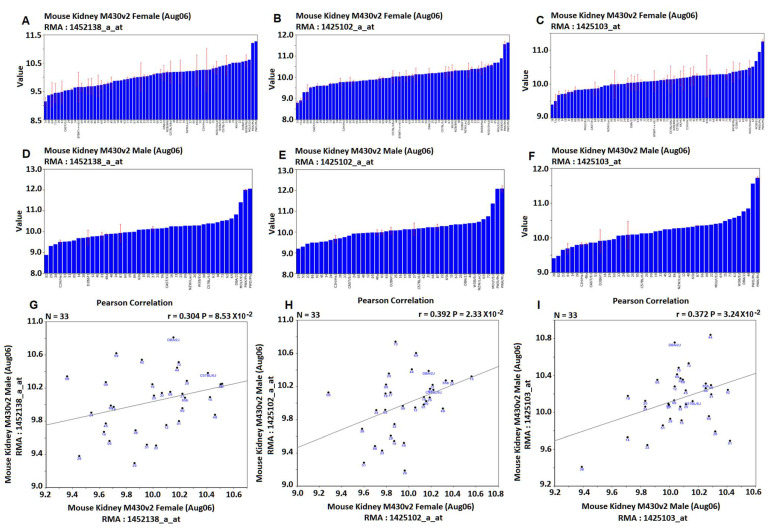
Expression levels of the *Ace2* in the female and male mice. (**A**–**C**) are relative expression levels in the female mice determined by microarray’s program. (**D**–**F**) are relative expression levels in the male mice. (**G**–**I**) are the correlation between female and male mice. The Y axis on the left of (**A**–**F**) are the relative expression levels of each prob, while the X axis are the classic strain names and strain number of BXD RI strains.

For *Ace2*, the same three probes were found from data of both female and male mice (Figure 1, the six graphic pictures on the top two rows). Although the mean expression levels between female and male mice are similar, their distributions of the expression levels among strains are different. The correlations of expression levels of these three probes between female and male are 0.304, 0.392, and 0.372, with *p* values of 0.0853, 0.0232, and 0.0324, respectively (Figure 1). Thus, there is a positive but weak correlation in the *Ace2* expression levels between female and male mice. Thus, considerable differences exist between female and male mice.

For *Adam17*, similar to that of *Ace2*, there is no strong correlation in the expression levels between female and male mice. R values are 0.006, 0.330, 0.227, and 0.445, with *p* values of 0.972, 0.061, 0.205, and 0.0086, respectively (Supplementary Figure S1). 

For *Tmprss2*, only one probe, 1,458,347, has a positive correlation between female and male mice, with r value of 0.468 and *p* value of 0.0054. The other three probes showed non or weak correlations, with r values of 0.320, 0.182 and −0.109, and *p* values of 0.096, 0.314, and 0.548, respectively (Supplementary Figure S2).

For CD146 (Mcam), there is no correlation between the expression levels of female and male mice. The r value is 0.296 with a *p* value of 0.0948 (Supplementary Figure S3).

### 3.3. Gene Network among Ace2 Adam17 Tmprss2 and Cd146

**Figure 2:**
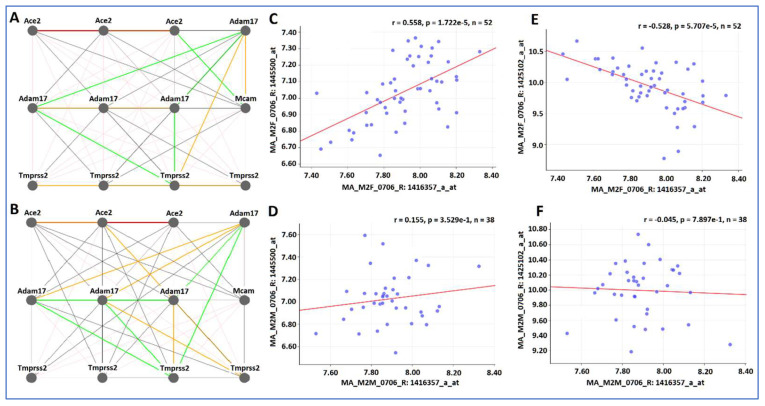
Gene expression network of *Ace2*, *Adam17*, *Tmprss2*, and *Cd146* in kidney among female and male mice. The 12 nodes in the graph show the selected traits. The node labels are drawn with 18.0 point font, and the edge labels are drawn with a 18.0 point font. In (**A**,**B**), the thick, wide, and solid red lines indicate the value of r is more than 0.75, representing the positive correlation between the genes on both ends of the connection. The thin, solid pent and green lines indicate the absolute value of r is between >0.35–<0.75, represent the positive and negative correlations. The dashed lines indicate the absolute value of r is <0.35, no correlations. (**A**) Pearson correlation coefficients among probes of four genes in the female mice. (**B**) Pearson correlation coefficients in the male mice. (**C**) Positive relationship between probes 1,445,500 of *Adam17* and *Cd146* in the female mice. (**D**) No correlation between probe 1,445,500 from Adam 17 and *Cd146* in the male mice. (**E**) Negative correlation between probe 1,425,102 in *Ace2* and *Cd146* in the female mice. (**F**) No correlation between probe 1,425,102 in *Ace2* and *Cd146* in the male mice.

Gene expression networks of *Ace2 Adam17 Tmprss2* and *Cd146* in kidney were constructed separately for the female and male mice (Figure 2). Because of the nature of multiple probes, the probes of genes connected in the gene network between two genes are not necessary with the same functional feature. Thus, the locations of probes, such as exon, intron, or 3′ UTR, may not be the same. Rather, the gene network represents the best connection among these four genes with expression levels in variety of parts.

Within the same sex, there are high similarities in the expression levels among probes of the same gene. In female mice, there are highly positive correlations among three probes of *Ace2*, four probes of *Tmprss2*, and three probes of *Adam17* (with one exception) (Figure 2A). While there are certain similarities, considerable differences exist between female and male mice. For example, the positive correlation among probes of *Tmprss2* are not strong (Figure 2B). There is a positive correlation between Adam 17 and *Cd146* in the female mice (Figure 2C), while there is no correlation between Adam 17 and *Cd146* in the male mice (Figure 2D). There is a negative relationship between probes of *Ace2* and *Cd146* in the female mice (Figure 2E) but no correlation between them in the male mice (Figure 2F). 

### 3.4. Metrix Correlations among the Same Sex

Three of these four genes have the multiple probes for the same gene. Metrix analysis indicates that within the same sex, probes for the same gene are often positively correlated.

The expression levels of three probes are highly positively correlated in *Ace2* gene within the same sex (Supplementary Table S1). In females, the probe 1,425,102 showed r values of 0.76 and 0.71, to probes 1,452,138 and 1,425,103, respectively. In males, probes are highly correlated. For example, probes 1,425,102 and 1,425,102 showed r values of 0.81 and 0.74, to probes 1,452,138 and 1,425,103, respectively. The correlation of probes between female and male are much weak. Only one pair, between 1,425,102 in the male and 1,452,138 in the female showed a r value of 0.50.

Among the four probes of *Adam17*, 1,445,500 stands out itself by negatively correlating to other three probes in either female or male while positively correlating to itself between female and male (Supplementary Table S2). The remaining three probes, 1,421,857 (last 4 exons), 1,421,858 (mid distal 3’ UTR), 1,421,859 (proximal 3-UTR), showed positive correlations within the same sex, while probe 1,421,857 in females has an r value of 0.452 with 1,421,858 in males.

Among four probes of *Tmprss2*, probes 1,419,154 has an r of 0.586/0.547 with 1,449,369 in the female mice. In males, the probe 1,449,369 has a negative correlation with probe 1,459,510, with r values of −0.444 and −0.368. No correlations were recognized among other probes (Supplementary Table S3).

### 3.5. Metrix Analysis of the Probes between the Sexes

The same probes between sex showed variable correlations. There is no (or weak) correlation in the one probe of *Cd146* between female and male mice.

The expression levels of two of the three probes in *Ace2* gene showed positive correlations between the sex. Probes 1,425,102 showed r values of 0.389 and 0.453 between female and male. Its male probe showed r values of 0.453 to female probe 1,452,183, and its female probe had a r value of 0.410 to the male probe 1,452,183 (Supplementary Table S1).

Among 4 probes of *Tmprss2*, only one probe, 1,449,369 showed a positive correlation between sexes, with r values of 0.417 and 0.457, with female to male and male to female, respectively. Other probes did not show a correlation between sexes (Supplementary Table S3).

Unlike probes in other genes, four probes of *Adam17* showed not only considerable positive correlations, but also a negative correlation between sexes. Specifically, probe 1,421,858 in the male had r values of 0.391, 0.382, 0.452 and −0.511 to four probes, 1,421,858, 1,421,859, 1,421,857, and 1,445,500, respectively, in the female. Probe 1,445,500 in the male had r values of −0.429, −0.397, −0.155 and 0.470 to four probes, 1,421,858, 1,421,859, 1,421,857, and 1,445,500, respectively, in the female (Supplementary Table S2).

Notably, the same probes between sexes never showed a negative correlation among all probes of all genes.

### 3.6. QTL Mapping of Probes of Four Genes

**Figure 3:**
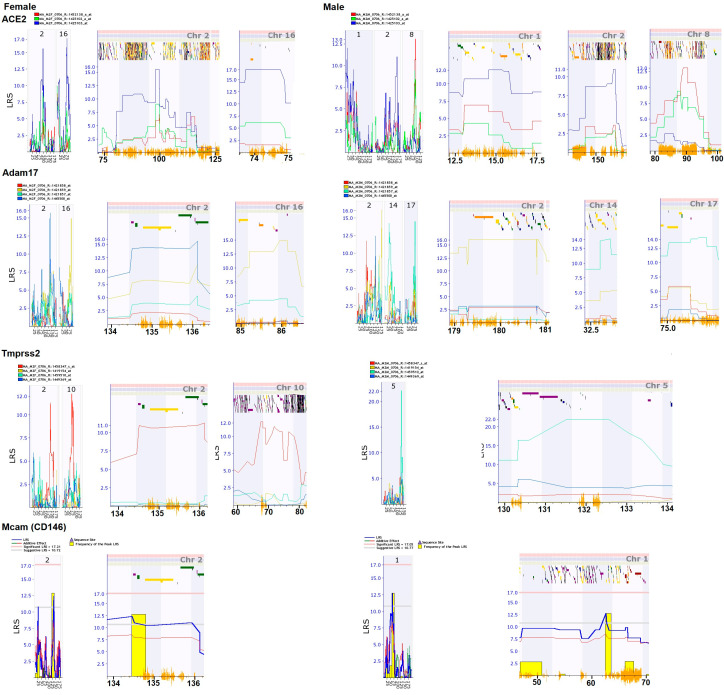
Expression QTL locations of *Ace2*, *Adam17*, *Tmprss2*, and *Cd146* in kidneys among female and male mice. The Y axis on the left of each mappi % figure are the levels of likelihood ratio statistic (LRS) of the QTL locus, while numbers under the axis at the bottoms are the genetic distance on the mapped chr. The small bars and dots of different colors on the top of each figure under the figure labels of female and male are the density of genes in the mapped region A positive additive coefficient (green line) indicates that DBA/2J alleles increase trait values. In contrast, a negative additive coefficient (red line) indicates that C57BL/6J alleles increase trait values. Yellow bars show the number of individuals contribute to the significance of the QTL.

The QTL locations for the expression levels (eQTL) of all of the probes of these four genes are generated with the mapping function of GeneNetwork. Figure 3 shows the QTL position on chromosome (chr) and the peak region of each QTL in the female and male separately. In general, there are similarities and differences in the genomic regions that regulate the expression levels of these four genes between female and male mice (Figure 3). As expected, in most of the cases, the non-coding regions, especially the 3’ UTR regions, encode regulatory sequences and not the exons for gene expression levels.

#### 3.6.1. eQTL for *Ace2*

There are significant differences in eQTL locations between the female and male mice. In females, a major eQTL is located on chr 16 and other eQTL is on Chr 2. The probe mapped on these two locations is 1,425,103, which is from the middle to discal 3’-UTR of the *Ace2* gene. The eQTL in the female on chr 16 is located between 73.75 and 74.77 Mb. It contains two genes, roundabout guidance receptor 2 (robo 2) and microRNA 691 (Mir69). The eQTL on chr 2 was located in the region between 98 and 102 Mb. Thus, the regulatory factors on these two genomic regions act on the region of middle to distal 3’-UTR of *Ace2* to control the expression levels of the *Ace2*.

In males, a suggestive eQTL is located on chr 1, 2, and 8 (Figure 3). The probe mapped on chr 1 and 2 is 1,425,103, same as that in the female. However, the chromosomal locations of eQTL on chr 2 are not overlapped between female and male mice. The eQTL on chr 8 is mapped with probe 1,452,138, which is from last 2–6 and 11 exons of the *Ace2*. The eQTL is located in the region between 88.3 to 90.7 Mb. Within this region, there are 13 genetic elements, including nine known genes. The eQTL on chr 1 for male is located between 13 and 16 Mb. Twenty genetic elements are located within this region, including 11 known genes.

#### 3.6.2. eQTL for *Adam17*

There are significant differences in eQTL positions between female and male mice. In females, a major eQTL is located on the same chr as that of *Ace2* on chr 16 and Chr 2 (Figure 3). The probe mapped on chr2 eQTL is 1,445,500, which is from the intron of the *Adam17*. The probe mapped on chr16 is 1,421,859, which is from the proximal 3-UTR. Thus, regulatory factors on chr 2 and chr 16 regulate the expression levels of *Adam17* by acting on the intron and proximal 3-UTR regions in *Adam17* in the female mice. However, the locations on these chromosomes are different from that of *Ace2*.

In male, suggestive eQTLs are located on chr 2, 14, and 17. On chr 2, the eQTL with highest Maximum LRS (Likelihood Ratio Statistic) is mapped with probe 1,421,859, while a second eQTL is mapped with probe 1,445,500. The eQTL on chr 14 and 17 are mapped with probe 1,421,857, which is from last 4 exons of Adam 17. Therefore, the regulation of expression of *Adam17* in the male mice is not only in the intron and proximal 3-UTR regions but in the region of last four exons.

#### 3.6.3. eQTL for *Tmprss2*

Female eQTLs were mapped on chr 2 and 10. The chromosomal location of the eQTL on chr 2 is the same as that of *Adam17*. Both eQTLs are mapped with probe 145,347, which is from distal 3’ UTR.

In males, one eQTL was mapped on chr 5 (Figure 3) with probe 1,459,510, which is from the intron of the *Tmprss2*.

#### 3.6.4. eEQTL for *Cd146*

There is no significant eQTLs on either female or male mice, and there is no eQTL overlap mapped regions between female and male mice (Figure 3). A potential eQTL in the female is mapped on chr 2, which is also on the same chromosomal position of eQTLs of Adam 17 and *Tmprss2*. The probe of CS146 is form the mid 3’ UTR of the gene. One eQTL is mapped on chr 1 from the male with no overlap with locations of any eQTL of other probes.

### 3.7. Potential Candidate Genes for eQTL Regulation

**Figure 4:**
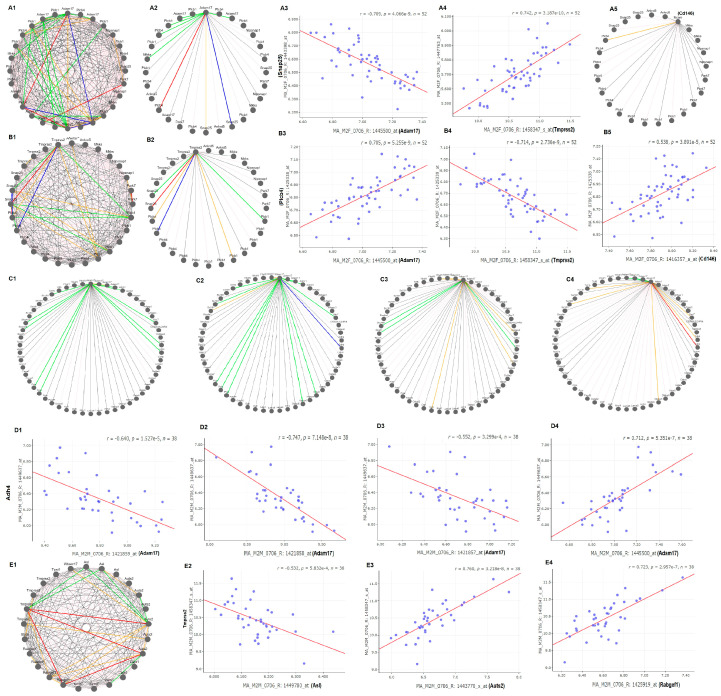
Candidate for eQTL of the *Ace2* complex between female and male mice. In (**A1**–**A4**,**C1**–**C4**,**D1**) the correlations among genes are indicated by lines with different colors and thickness. The thick wide solid red lines and blue lines indicates the absolute value of r is more than 0.75, representing the positive or negative correlation between the genes on both ends of the connection.The thin solid pent and green lines indicate the absolute value of r is between >0.35 and <0.75, represent the positive and negative correlations. The dashed lines indicate the absolute value of r is <0.35, no correlations. (**A1**–**A5**,**B1**–**B5**) are for female. (**C1**–**C4**,**D1**–**D4**,**E1**–**E4**) for male mice. Red and purple colors for positive, blue and green for negative correlation, and other colors for non-correlation in network graphic structures in (**A1**–**A5**,**B1**–**B5**,**C1**–**C4**,**D1**–**D4**,**E1**,**E2**) are correlation scatterplot of expression levels in gene pairs in either positive or negative manner.

In females, candidate genes that regulate the expression of these key genes are mainly on two regions of chr2 (Figure 3). The potential candidates were selected at the eQTL location by their strong co-expression with the four key genes (Figure 4). The major eQTL region for the regulation of *Adam17*, *Tmprss2*, and CD145 is mapped between 134 and 137 Mb. This region contains 21 genetic elements (Supplementary Table S4). Potential candidates with polymorphism, function transcripts, and human counterparts are Haol, Plcb1, Plcb4, Park7, Ankrd5, Snap25, and Mkks. Gene network analysis indicated that among these genes, Plcb4 (phospholipase C, beta) and Snap25 (Synaptosomal-associated protein 25) are the major regulators to the expression of these three genes (Figure 4A1,A2,B1,B2). The expression levels of Plcb4 and Snap25 are correlated in a opposite way to the expression levels of *Adam17* and *Tmprss2*. While the expression of Snap25 is negatively correlated to that of *Adam17*, it is positively correlated to that of *Tmprss2* (Figure 4A3,A4). In the opposite direction, while the expression level of Plcb4 is positively correlated to that of *Adam17*, it is negatively correlated to the *Tmprss2* (Figure 4B3,B4). The expression level of *Cd146* does not correlate to any gene in the list except Plcb4 (Figure 4A5). It is positively correlated to the expression of *Cd146*. One region with suggestive eQTL is for the regulation of *Ace2* and is between Mb which contains 11 genetic elements (Supplementary Table S5). From these elements, potential candidate genes with polymorphism, functional transcripts, and human counterpart are Traf6 and Commd9. The has been no previous report on the connection between these two genes and the *Ace2*. Their expression level is not strongly correlated to that of the *Ace2*. Other eQTLs are located on chr 16 and 10 (Figure 3).

In males, regulation of the Ace/Adma17/*Tmprss2*/*Cd146* is complex. There was no significant eQTL for *Ace2*. The eQTL for *Adam17* is located on chr 2 but with a different location from that of the female mice. The genomic region is from 179 to 181 Mb, which includes 57 genetic elements (Supplementary Table S6). Among them, 20 have polymorphisms, functional transcripts, and human counterpart genes. They are Cdh4, Trf4, Psma7, Ss18l1, Gtpbp5, Hrh3, Osbpl2, Adrm1, Lama5, Rps21, Cables2, Gata5, Slco4a1, Ntsr1, Ogfr, Col9a3, Tcfl5, Ythdf1, Birc7, and Arfgap1. Gene Network analysis showed that the expression of most of these genes are negatively related to the expression level of three probes of *Adam17* (Figure 4C1–C4). Cdh4 plays significant role in the regulation of *Adam17*. One probe of Cdh4, 2,449,637, is negatively correlated to 3 probes of *Adam17* (Figure 4D1–D3) and the same probe is positively correlated to the probe 1,445,500 (Figure 4D4). The other eQTL is the regulator of *Tmprss2*. It is located on chr5 in a region between 130 and 134 Mb. Within the total of 30 elements (Supplementary Table S7), candidates include Asl, Tpst1, Kctd7, Rabgef1, Sbds, Caln1, Wbscr17, and Auts2. Other eQTL are located on chr 1, 2, 8, 14, and 17 (Figure 3). Gene network analysis indicated that while the expressions of most genes are not closely correlated to that of *Tmprss2* (Figure 4E1), Asl, Auts2, and Rabgef1 influence the expression of *Tmprss2*, in negative, positive, and positive ways, respectively (Figure 4E2–E4).

### 3.8. Co-Variation Matrix Comparison and Alternative Indirect Pathway Analysis

To further investigate the differences in the genomic level, we compared the top 100 most correlated genes to one probe of each of these four genes. The top 100 genes with expression levels most correlated to one probe of these four genes are collected with the function of “Calculate Correlations” in the GeneNetwork (Supplementary Tables S8 and S9). For genes with multiple probes, the probe with highest expression level was chosen for the analysis. The same genes between female and male mice are in the range between 0 and 3 among the top 100 most closely co-expressed genes of these four key genes (Supplementary Figure S4). In particular, for the probe of *Cd146*, there is no same gene among the top 100 genes between female and male mice. In contrast, there are 11 identical genes between female and male mice for the control gene, B-actin.

### 3.9. Downstream of Ace2 Pathway

**Figure 5:**
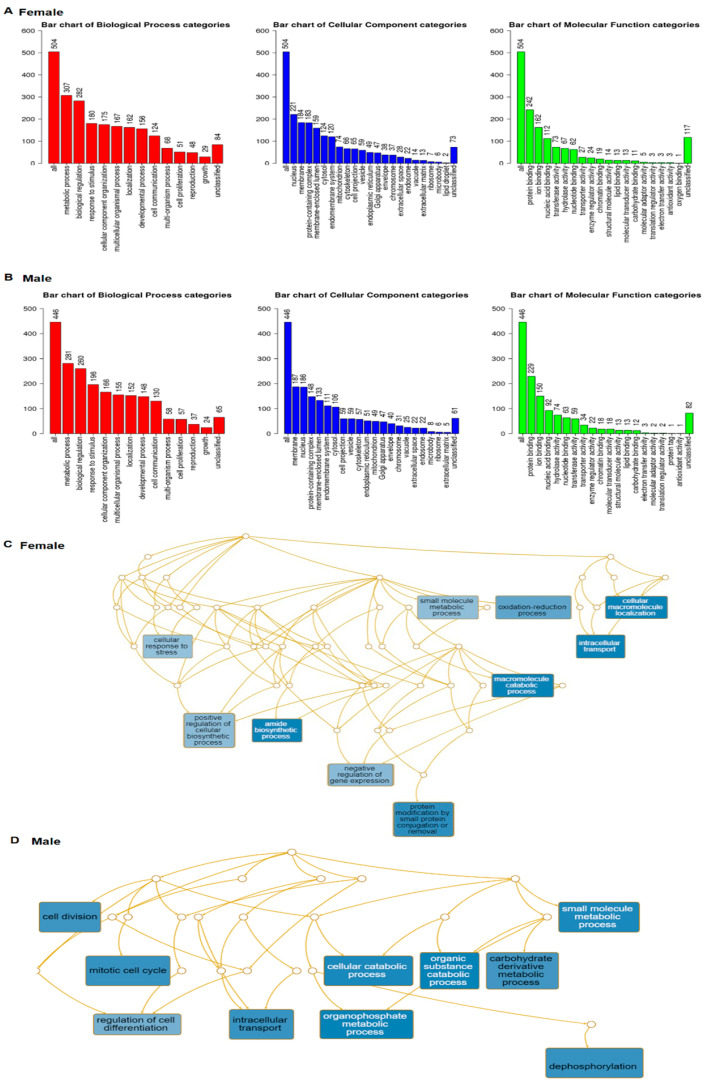
Weighted set coverage of *Ace2* pathways between females and males. Each Biological Process, Cellular Component and Molecular Function category is represented by a red, blue and green bar, respectively. The height of the bar represents the number of IDs in the functional category and. (**A**) Numbers of genes involve different pathways of *Ace2* in the female. (**B**) Numbers of genes involve different pathways of *Ace2* in the male. (**C**). pathway network of *Ace2* in the female. (**D**). pathway network of *Ace2* in the male.

From three probes of *Ace2*, we collected the top 200 most closely correlated genes ranked by the Genetic Correlation comparison to each of 3 probes. From the females, altogether, 569 genes (after excluding the repetitive) (Supplementary Table S10) were used to construct the pathway with WEB-based GEne SeT AnaLysis Toolkit [31]. Go slim summary presented 504 genes in three major categories, including biological process, cellular component, and molecular function (Figure 5A). From the males, a total of 522 genes (after excluding the repetitive) (Supplementary Table S11) were collected. There were 446 genes in the same three categories, which are presented in Figure 5B. There are considerable differences in the gene numbers of each category.

Enrichment results identified the top 10 size constrained weighted sets of genes from both sexes (Figure 5C,D). The functions of these gene sets are also significantly different. Only two gene sets, intracellular transport and small molecule metabolic process, are the same between the sexes.

In females, the top gene sets include positive regulators of the cellular biosynthetic process, negative regulators of gene expression, and protein modification by small protein conjugation or removal, amide biosynthetic process, and cellular response to stress (Figure 5C). In males, the top gene sets are cellular catabolic process, organic substance catabolic process, organophosphate metabolic process, and small molecule metabolic process (Figure 5D).

### 3.10. Potential Sex Difference in the Ace2 Complex

Based on our data, we proposed different pathways in upstream interaction within complex and downstream regulations in the *Ace2* complex.

#### 3.10.1. Upstream Regulation

In females, one major eQTL on chr 2 influences the *Ace2* expression by regulation of the expression levels of *Adam17*, *Tmprss2*, and *Cd146* while a suggestive eQTL may directly regulate the expression of *Ace2*.

**Figure 6:**
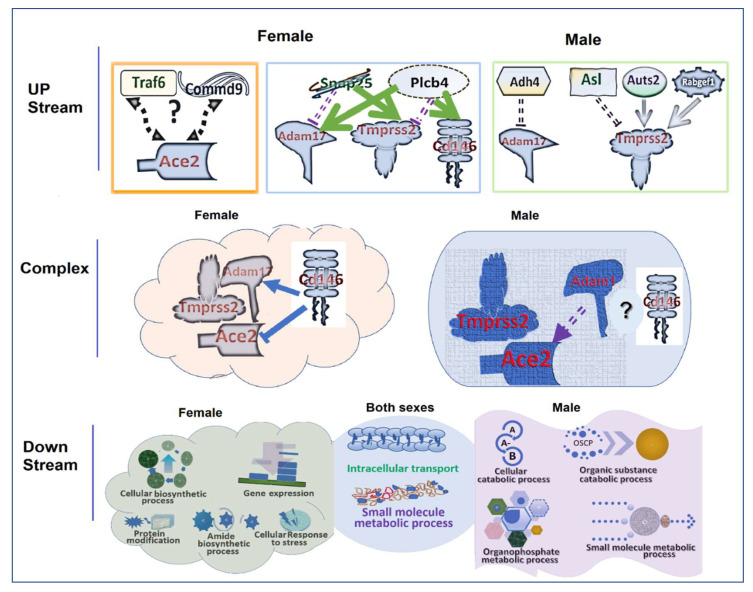
Sex different regulation processes in the *Ace2*/*Adam17*/*Tmprss2* pathways. The process was divided into three stages, the upstream regulation, interplays within the complex, and *Ace2* trigged downstream processes. Arrow ended lines are positive regulations of a genes and/or protein to the other while the T ended lines indicate a negative regulation. Solid lines indicate known reaction while dashed lines indicate proposed reactions. Molecules tiered together indicate they are binding together.

Within the suggestive eQTL, Traf6, and Commd9 are the only candidate genes that may influence the expression of *Ace2* (Figure 6).

Within the major eQTL, two genes may play essential role. One is Snap25. In humans, it is known that intracellular vesicles travel among cellular compartments and deliver their specific cargo to target membranes by membrane fusion. The specificity of cargo delivery and membrane fusion is controlled, in part by the pairing of vesicle v-SNAREs (soluble N-ethylmaleimide-sensitive factor attachment protein receptors) with target membrane t-SNAREs, such as SNAP25 [32]. In females, Snap25 is negatively correlated to that of *Adam17*, positively to that of *Tmprss2* (Figure 6). The other gene is Plcb. In humans, PlCB4 is coupled to metabotropic glutamate receptors. Signal transduction via the PI cycle plays a role in the light response in vertebrate and invertebrate retinas. It is not known whether its function is related to kidney function. Unlike Snap25, its expression level is positively related to that of *Adam17* and negatively to that of *Tmprss2*. In addition, it is positively correlated to that of *Cd146* (Figure 6).

In males, four candidate genes may influence the expression of *Adam17* and *Tmprss2*. Adh4 may negatively regulate the expression of *Adam17*. Three other genes, Asl, Auts2, and Rabgef1 negatively, positively, and positively regulate the expression of *Tmprss2*, respectively (Figure 6).

#### 3.10.2. Interaction within the *Ace2*/*Adam17*/*Tmprss2* Complex

In females, *Cd146* plays an important role in the regulation of the expression levels of *Ace2* by directly interacting with *Ace2* by itself. At the same time, interactions among *Adam17*, *Tmprss2*, and *Cd146* affect the expression of *Ace2* (Figure 6).

In males, *Adam17* may play a bigger role in the regulation of the expression of *Ace2*, while the complex of *Tmprss2*, *Adam17*, and *Cd146* also has some effect on the expression of *Ace2* (Figure 6).

#### 3.10.3. Downstream Pathways

*Ace2* drives the intracellular transport and small molecule metabolic process in both female and male mice. In females, *Ace2* imposes a more positive influence on the cellular biosynthetic process, negatively affecting the gene expression levels. It also modulates the protein modification, amide biosynthetic process, and cellular response to stress (Figure 6).

In males, *Ace2* has more influence on the cellular catabolic process, organic substance catabolic process, organophosphate metabolic process, and small molecule metabolic process (Figure 6).

## 4. Discussion

Our data suggest that key genes in CKD may also have significant roles in the sex difference in COVID-19. The important role of *Ace2* in both diseases has been known. Therefore, we investigated the relative genes in the pathway of *Ace2*, known genes in CKD such as *Adam17*, *Tmprss2*, and *Cd146*. The suggestive low positive correlation from 0.304 to 0.392 in the co-expression of *Ace2* between sexes suggests that the expression level of *Ace2* is possibly relatively stable by itself in both sexes. However, the expression level will be significantly influenced by the other three partners. For example, the correlation between sexes in one probe of *Adam17* is 0.006. Thus, there is no correlation in the expression between female and male. Similarly, two of the four probes of *Tmprss2* showed a none and a negative correlation between sexes. The single probe of *Cd146* also showed no correlation between sexes. These data suggest that the sex differential expression among these genes may influence each other, especially under abnormal or disease conditions. One discovery is that in the female the expression level of *Cd146* is closely related to that of *Adam17* and *Ace2* while in the male such a relationship does not exist. These data suggests that it is important to further investigate whether *Cd146* plays a critical role in the sex difference in either CKD or COVID-19.

Investigating the eQTL of these genes revealed significant sex difference in the upstream regulation of the expression levels of these genes. From the chromosomal loci of these eQTL, different sets of candidate genes regulate the expression of the *Ace2*/*Adam17*/*Tmprss2* complex in females and in males. Full investigation of these genes may enhance our understanding of the COVID-19 pathway, selection of target molecules for COVID-19 treatment, and personalized patient treatment, especially with regard to sex differences. The same patterns of these genes in the sex difference suggest that their function may lead to the sex difference in pathology and epidemiology of the COVID-19.

The sex difference among these genes appears in all spectrums, ranging from expression levels, gene network as well as regulations. First of all, the expression levels of these genes in the female are not associated the expression levels of male. None of the probes in the female showed strong correlation to the same probe in the male mice. Secondly, the gene network among these four genes in the female mice are different from these in the male mice. Furthermore, detailed Metrix analysis indicated that strong positive correlations exist with probes of the same sex but not in the same probe between sexes. These sex differences may reflect the gender difference of COVID-19 in human populations.

The genetic loci that regulate the expression levels of these genes in the female mice are different from that of the male mice. None of eQTL of probes of female mice are at the similar locations of the eQTL of male mice, thus, the whole set of genes demonstrated a significant sex difference, suggesting the genetic regulation of the expression of these genes are different between the female and male mice.

Overall, the genetic regulation of expression levels of these genes in the female showed a much greater similarity than that in the male mice. In females, the probes of four genes are all mapped on the similar position on chr 2. In males, the eQTL locations are spread into several chrs, including chr 1, 2, 5, 8, 14 and 17. Although one eQTL on chr 1 was mapped by probes of *Ace2* and *Cd146* and one eQTL on chr 2 was mapped by probes of *Ace2* and Adam 17, their positions on chr between different genes are not the same. It is worth to further investigate whether such a difference plays an important role in the sex difference in CKD as well as in COVID-19. In particular, it is necessary to conduct a detailed study on the eQTL on chr 2 in female mice.

There is variation in the sex difference of mapped eQTL among these four key genes. For *Ace2*, two and three eQTL locations were identified from the female and the male respectively. The eQTL were from different locations on chr 2. Although one eQTL was located on chr 2 in both sexes, their locations are far away from each other. The eQTL of females is located in region between between 98 Mb and 102 Mb while in males, the location is around 150 Mb. The likelihood for an experimental error that one eQTL was mapped into different location is small. In addition, the two eQTL locations from female had much higher LRS than that from the male. The LRS scores for the eQTL of the male were below the statistic threshold level, at a suggestive level. In particular, an eQTL in a small chr region between 73.75 and 74.77 Mb on chr 16 from the female while in the male there is no sign of any eQTL in this region on chr 16. For *Adam17*, there are similarities and differences in the regulation of its expression. Both sexes have the eQTL on chr 2. In females, only one probe 1,445,500 was mapped on a region between 134.5 Mb and 136 Mb. In males, the eQTL of two probes were mapped on chr 2. One is located around 180 Mb while the other is close to that of the female. Thus, they are potential partially regulated by the same gene(s) on chr 2. The eQTL for other probes showed significant differences. In females, the probe 1,421,859 was mapped on to chr 16, while it did not map to any chr in males. In contract, in males, the eQTL on chrs 14 and 17 were mapped with probe 1,421,857. No eQTL was mapped in females with the probe 1,421,857. For Tmpress2 and *Cd146*, there was no eQTL mapped on the same chr between the probes from females and males. These results emphasize the significant differences between sexes in the regulation of these genes.

One important finding from this study is the importance of the eQTL on chr 2. In females, at least one probe from each of these four genes was mapped on chr 2. In males, probes from two genes, *Ace2* and *Adam17*, were mapped on the chr 2. The LRS values in four of these eQTL reached or were close to the significant level. Thus, there is no doubt that these chromosomal regions play important roles in the regulation of these key genes. Future studies on the detailed analysis of the genes in the eQTL regions of chr 2 may discover critical information on the regulation of *Ace2* complex and sex differences. The eQTL locations may be further narrowed down with larger populations or backcrossing strategies.

One issue in the mapping of the eQTL of these genes is the difference among multiple probes. The eQTL with different probes from the same gene were mapped on to different chrs. These probes are from 3’-UTR including distal 3′ UTR, exons and introns, but not from the 5’-UTR. While the regulatory elements in a gene are usually located in 3’-UTR regions, polymorphisms in exon and intron regions also affect the gene expression. Without detailed genomic analysis and experimental confirmation, it is difficult to prioritize or eliminate any of them. However, in the future study of the regulatory mechanisms of these eQTL, it is essential to recognize these differences when searching the regulatory elements and when speculating potential mechanisms.

The gene network was built based on co-expression ecoefficiencies. The algorithm and the methodology for the network has been widely accepted as the standard tool. However, one needs to keep in mind that co expression does not necessarily means a co-regulation or causal relation. One also needs to pay attention to the variations of the r values among different gene pairs. In general, the absolute r values larger than 0.75 is considered as the strong correlation between the genes in the connection. Future studies will identify the causal gene among these multiple candidate genes in different loci. Functional confirmation is necessary to confirm the candidate genes.

We present proposed differential gene pathways in sex differences in the up-stream, within the complex, and the down-stream of the *Ace2*/*Adam17*/*Tmprss2* pathways. Up-stream, several eQTL regions that play important regulatory role in the expression of these genes either in the female only or in the male only were speculated. The candidate genes identified from these eQTL regions are only based on the expression levels and chromosomal positions. Therefore, these genes should not be considered as the only candidates for these eQTL. It is possible that other genes are functionally regulating these key genes in *Ace2*/*Adam17*/*Tmprss2* pathways. Furthermore, about half of these eQTL are at the suggestive level, and therefore their locations may not be definitive. Within their interaction complex, the difference is caused by the *Cd146*. In the down-stream part, the differences and similarities are mainly on the metabolic processes. There is no doubt that these proposed differential expression pathways need to be confirmed by future studies. One important gene in these pathways is the *Cd146*. *Cd146* is well known for its role in relation to chronic renal failure and the expression in the male is different from that in the female [6]. It recently has been linked to the COVID-19 [33]. The sex differential interaction between the *Cd146* and *Ace2*/*Adam17*/*Tmprss2* complex may reflect the sex difference in the mortality of COVID-19 disease. Detailed study on the *Cd146* may explain the molecular mechanisms of regulation of *Ace2*/*Adam17*/*Tmprss2* pathway by *Cd146* between the female and the male.

Finally, the extended list of genes that are closely correlated to female is mostly different from that of the male. Among top 100 genes, only less than three genes are the same between both sexes. There is not even one same gene among top 100 closely related genes between female and male mice. Thus, at the while genome level, there is a significant sex difference in the gene network pathways. The investigation of the top 200 genes from each of the three probes of *Ace2* indicate that there is considerable difference in the downstream molecular pathway. The top sets of genes in the down-stream of female and males are different from each other. Only two of 10 gene sets are the same between sexes.

Our data agrees with the reports that COVID-19 influence of the virus on the urinary system [34,35]. Menon et al. pointed out that patients with kidney disease have high mortality rates from COVID-19 and understanding the disease-specific molecular processes in COVID-19 relative to kidney disease can have a significant impact on public health [33]. Hallak and colleges summarized the potential connection between COVID-19 and urinary tract, reproductive tract, and semen [35]. Recently, SARS-CoV-2 RNA was found in the urine of patients with persistent symptoms [36]. These results suggest that during the COVID-19 pandemic scientific effort must be made to understand the role of the urogenital system in the SARS-CoV-2 infection in the clinical setting. As both COVID-19 and CKD involve in urinary system, there is a possibility that there is a connection between pathways in these two diseases.

Different age groups suffer differently from COVID-19 and CKD. In our study, the data on gene expression profile of kidney were collected from mice between 50 and 99 days of age (GeneNetwork 2). The two parents of the population usually live up to one or even two years. Therefore, these mice are not considered as old mice. It is not known whether the sex difference from this study will be the same in the old mice. In addition, from the top 10 size constrained weighted sets of genes, only two gene sets, intracellular transport and small molecule metabolic process, are the same between the sexes. The genes in the set of intracellular transport include more than 50 genes such as Tmed4, Dnm3, Vipas39, Bach2, and Calm1. The genes in the set of small molecule metabolic process include more than 60 important genes for small molecules, such as *Acad11, Abhd4, Acy1, Adk,* and *Atp6v1b2*. These genes are the same between sexes most likely because their essential function in both sexes. Detailed study of the similarity and difference between sex in these two diseases may reveal important gene targets for the pharmaceutical application.

Our gene set comparison suggests that sex differences in these diseases are reflected in important gene sets, such as oxidation reduction process, cellular response to stress, cellular macromolecule localization, mitotic cell cycle, organic substance catabolic process, and carbohydrate derivative metabolic process. Abnormity in these pathways may lead to the increased small vessel permeability, oxidative damage due to mitochondrial dysfunction, tissue hypoxia, and eventually sex difference in the autoimmunity in response to disease pathogen. Finally, *Tmprss2* may play a critical role in the sex difference in the *Ace2*/*Adam17*/*Tmprss2* complex. It has been known that androgen signaling promotes co-recruitment of androgen receptor and topoisomerase II-beta to sites of TMPRSS2-ERG genomic breakpoints [37]. Furthermore, androgen stimulation resulted in the de novo production of TMPRSS2-ERG fusion transcripts. Out data show *Tmprss2* in the middle of the action of the sex differential complex in the upstream and *Ace2*/*Adam17*/*Tmprss2* complex. We therefore speculate that the sex difference in both COVID-19 and CKD may be brought about by the androgen stimulated *Tmprss2*-ERG fusion transcript. Future study on the link between the androgen and the sex difference in the *Ace2*/*Adam17*/*Tmprss2* complex in COVID-19 and CKD is necessary.

## 5. Conclusions

Our data found a potential sex difference and pathways that may function in both CKD and COVID-19 molecular processes. The sex difference is mainly reflected in the interactions between the *Cd146* and *Ace2*/*Adam17*/*Tmprss2* complex. Future study on whether sex hormones play a role in these interactions is necessary.

## Data Availability

Data are collected during the period between 4 December 2020 and 18 April 2022, and available from GeneNetwork at http://www.genenetwork.org.

## Supplementary Materials

The following supporting information can be downloaded at: https://www.mdpi.com/article/10.3390/jpm12071190/s1, Supplementary Table S1. Correlation Matrix among probes of Ace2. Supplementary Table S2. Correlation Matrix among probes of Adam17. Supplementary Table S3. Correlation Matrix among probes of Tmprss2. Supplementary Table S4. Candidate eQTL chr2 female Adma17. Supplementary Table S5. Candidate eQTL chr2 female ace2. Supplementary Table S6. Candidate eQTL chr2 male adam17. Supplementary Table S7. Candidate eQTL chr5 male Tmprss2. Supplementary Table S8. Top 100 genes in the female for 3 probes in Ace2. Supplementary Table S9. Top 100 genes in the male for 3 probes in Ace2. Supplementary Table S10. Top 200 genes closely correlated to the Ace2 probes in the female mice. Supplementary Table S11. Top 200 genes in the male for 3 probes in Ace2. Supplementary Figure S1. Expression levels of Adam17 in kidney between female and male. Supplementary Figure S2. Expression levels of Tmprss2 in kidney between female and male. Supplementary Figure S3. Expression levels of Mcam (CD146) in kidney between female and male. Supplementary Figure S4. Similarity of top 100 most correlated genes to probes of four genes.
