# Supplementary material for: Potential Similarities in Sex Difference in Key Genes and Their Expression, Network, EQTL and Pathways between COVID-19 and Chronic Kidney Disease Based on Mouse Model
Source: J Pers Med. 2022 Jul 21;12(7):1190. doi: 10.3390/jpm12071190 (PMC9323909; doi:10.3390/jpm12071190)

Supplemental Table S1. Correlation Matrix among probes of Ace2*

	Spearman Rank Correlation (rho)						
Pearson		Trait1	Trait2	Trait3	Trait4	Trait5	Trait6
	**Trait 1: MA_M2M_0706_R::1452138_a_at** Ace2 on Chr X @ 164.167855 Mb angiotensin I converting enzyme (peptidyl-dipeptidase A) 2 last 2~6 and 11 exons	*n* 38	0.822 38	0.720 38	0.247 34	0.292 34	0.201 34
	**Trait 2: MA_M2M_0706_R::1425102_a_at** Ace2 on Chr X @ 164.182645 Mb angiotensin I converting enzyme (peptidyl-dipeptidase A) 2 3'-UTR and last 4 exons	0.806 38	*n* 38	0.738 38	0.501 34	0.453 34	0.204 34
	**Trait 3: MA_M2M_0706_R::1425103_at** Ace2 on Chr X @ 164.187853 Mb angiotensin I converting enzyme (peptidyl-dipeptidase A) 2 middle to distal 3'-UTR	0.742 38	0.683 38	*n* 38	0.221 34	0.314 34	0.188 34
	**Trait 4: MA_M2F_0706_R::1452138_a_at** Ace2 on Chr X @ 164.167855 Mb angiotensin I converting enzyme (peptidyl-dipeptidase A) 2 last 2~6 and 11 exons	0.245 34	0.410 34	0.209 34	*n* 52	0.721 52	0.495 52
	**Trait 5: MA_M2F_0706_R::1425102_a_at** Ace2 on Chr X @ 164.182645 Mb angiotensin I converting enzyme (peptidyl-dipeptidase A) 2 3'-UTR and last 4 exons	0.248 34	0.389 34	0.289 34	0.763 52	*n* 52	0.706 52
	**Trait 6: MA_M2F_0706_R::1425103_at** Ace2 on Chr X @ 164.187853 Mb angiotensin I converting enzyme (peptidyl-dipeptidase A) 2 middle to distal 3'-UTR	0.202 34	0.218 34	0.325 34	0.492 52	0.695 52	*n* 52

*Lower left cells show Pearson product-moment correlations; upper right cells provide Spearman rank order correlations. Each cell also contains the number of cases (n). Red color = values higher than 0.7. Orange = between 0.5 and 0.7. Blue = Values lower than -0.7; Green = between -0.5 and -0.7.

Supplemental Table S2. Correlation Matrix among probes of Adam17.

	Spearman Rank Correlation (rho)								
		Trait1	Trait2	Trait3	Trait4	Trait5	Trait6	Trait7	Trait8
Pearson r	Trait 1: MA_M2F_0706_R::1421858_at Adam17 on Chr 12 @ 21.323941 Mb a disintegrin and metalloproteinase domain 17 mid distal 3' UTR	*n* 52	0.408 52	0.276 52	-0.414 52	0.410 34	0.213 34	0.262 34	-0.447 34
	Trait 2: MA_M2F_0706_R::1421859_at Adam17 on Chr 12 @ 21.324648 Mb a disintegrin and metalloproteinase domain 17 proximal 3-UTR	0.402 52	*n* 52	0.543 52	-0.545 52	0.386 34	0.255 34	0.073 34	-0.378 34
	Trait 3: MA_M2F_0706_R::1421857_at Adam17 on Chr 12 @ 21.325298 Mb a disintegrin and metalloproteinase domain 17 last 4 exons	0.244 52	0.520 52	*n* 52	-0.539 52	0.502 34	0.185 34	0.077 34	-0.148 34
	Trait 4: MA_M2F_0706_R::1445500_at Adam17 on Chr 12 @ 21.353419 Mb a disintegrin and metalloproteinase domain 17	-0.389 52	-0.531 52	-0.501 52	*n* 52	-0.541 34	-0.307 34	-0.198 34	0.503 34
	Trait 5: MA_M2M_0706_R::1421858_at Adam17 on Chr 12 @ 21.323941 Mb a disintegrin and metalloproteinase domain 17 mid distal 3' UTR	0.391 34	0.382 34	0.452 34	-0.511 34	*n* 38	0.690 38	0.420 38	-0.602 38
	Trait 6: MA_M2M_0706_R::1421859_at Adam17 on Chr 12 @ 21.324648 Mb a disintegrin and metalloproteinase domain 17 proximal 3-UTR	0.184 34	0.281 34	0.155 34	-0.241 34	0.697 38	*n* 38	0.546 38	-0.582 38

	**Trait 7:** MA_M2M_0706_R::1421857_at<br>Adam17 on Chr 12 @ 21.325298 Mb<br>a disintegrin and metalloproteinase domain 17 last 4 exons	**0.136**<br>**34**	**0.082**<br>**34**	**-0.000**<br>**34**	**-0.161**<br>**34**	**0.355**<br>**38**	**0.571**<br>**38**	***n***<br>**38**	**-0.415**<br>**38**
	**Trait 8:** MA_M2M_0706_R::1445500_at<br>Adam17 on Chr 12 @ 21.353419 Mb<br>a disintegrin and metalloproteinase domain 17	**-0.429**<br>**34**	**-0.397**<br>**34**	**-0.155**<br>**34**	**0.470**<br>**34**	**-0.568**<br>**38**	**-0.563**<br>**38**	**-0.403**<br>**38**	***n***<br>**38**

* *Lower left cells show Pearson product-moment correlations; upper right cells provide Spearman rank order correlations. Each cell also contains the number of cases (n). Red color = values higher than 0.7. Orange = between 0.5 and 0.7. Blue = Values lower than -0.7; Green = between -0.5 and -0.7.

Supplemental Table S3. Correlation Matrix among probes of Temprss2.

	Spearman Rank Correlation (rho)								
Pearson r		**Trait 1**	**Trait 2**	**Trait 3**	**Trait 4**	**Trait 5**	**Trait 6**	**Trait 7**	**Trait 8**
	**Trait 1:** MA_M2F_0706_R::1458347_s_at<br>Tmprss2 on Chr 16 @ 97.564715 Mb<br>transmembrane protease, serine 2<br>3' UTR	***n***<br>**52**	**0.103**<br>**52**	**-0.074**<br>**52**	**-0.151**<br>**52**	**0.417**<br>**34**	**0.229**<br>**34**	**-0.049**<br>**34**	**0.032**<br>**34**
	**Trait 2:** MA_M2F_0706_R::1419154_at<br>Tmprss2 on Chr 16 @ 97.564874 Mb<br>transmembrane protease, serine 2	**0.131**<br>**52**	***n***<br>**52**	**0.004**<br>**52**	**0.586**<br>**52**	**0.013**<br>**34**	**0.235**<br>**34**	**-0.016**<br>**34**	**0.035**<br>**34**
	**Trait 3:** MA_M2F_0706_R::1459510_at<br>Tmprss2 on Chr 16 @ 97.566141 Mb<br>transmembrane protease, serine 2	**-0.100**<br>**52**	**-0.092**<br>**52**	***n***<br>**52**	**-0.024**<br>**52**	**-0.039**<br>**34**	**0.302**<br>**34**	**0.215**<br>**34**	**-0.208**<br>**34**

	**Trait 4:** **MA_M2F_0706_R::1449369_at** Tmprss2 on Chr 16 @ 97.567022 Mb transmembrane protease, serine 2 exons 10, 11, and 12	-0.177<br>52	0.547<br>52	-0.005<br>52	*n*<br>52	-0.202<br>34	0.123<br>34	0.053<br>34	-0.124<br>34
	**Trait 5:** **MA_M2M_0706_R::1458347_s_at** Tmprss2 on Chr 16 @ 97.564715 Mb transmembrane protease, serine 2 3' UTR	0.457<br>34	-0.004<br>34	-0.065<br>34	-0.278<br>34	*n*<br>38	0.272<br>38	-0.140<br>38	-0.159<br>38
	**Trait 6:** **MA_M2M_0706_R::1419154_at** Tmprss2 on Chr 16 @ 97.564874 Mb transmembrane protease, serine 2	0.211<br>34	0.319<br>34	0.298<br>34	0.026<br>34	0.349<br>38	*n*<br>38	-0.059<br>38	0.201<br>38
	**Trait 7:** **MA_M2M_0706_R::1459510_at** Tmprss2 on Chr 16 @ 97.566141 Mb transmembrane protease, serine 2	-0.038<br>34	0.031<br>34	0.184<br>34	0.090<br>34	-0.151<br>38	-0.094<br>38	*n*<br>38	-0.368<br>38
	**Trait 8:** **MA_M2M_0706_R::1449369_at** Tmprss2 on Chr 16 @ 97.567022 Mb transmembrane protease, serine 2 exons 10, 11, and 12	0.037<br>34	0.069<br>34	-0.240<br>34	-0.111<br>34	-0.107<br>38	0.195<br>38	-0.444<br>38	*n*<br>38

*Lower left cells show Pearson product-moment correlations; upper right cells provide Spearman rank order correlations. Each cell also contains the number of cases (n). Red color = values higher than 0.7. Orange = between 0.5 and 0.7. Blue = Values lower than -0.7; Green = between -0.5 and -0.7.

Supplemental Table S4. Candidate genes on Chr 2 in female for Adma17

Index	Symbol	Mb Start	Length (Kb)	SNP Count	SNP Density	Avg Expr	Human Chr	Mb Start (hg19)	Gene Description	PolymiRTS Database >>	Gene Weaver Info Content >>
1	Hao1	134.497360	56.992	5	0.087732	--	20	67.115672	hydroxyacid oxidase 1, liver		

2	**Tmx4**	**134.594501**	**49.620**	**18**	0.362757	--	--	**--**	thioredoxin-related transmembrane protein 4 (Tmx4), mRNA.		
3	**Txndc13**	**134.594502**	**49.619**	**18**	0.362764	--	--	**--**	thioredoxin domain containing 13		
4	**Plcb1**	**134.786163**	**689.095**	**1440**	2.089697	--	20	**74.685334**	phospholipase C, beta 1		
5	**4930545L23Rik**	**135.169572**	**46.044**	**81**	1.759187	--	--	**--**	RIKEN cDNA 4930545L23 gene		
6	**9630028H03Rik**	**135.580529**	**2.691**	0	0	--	--	**--**	RIKEN cDNA 9630028H03 gene		
7	**Plcb4**	**135.659546**	**323.580**	**260**	0.803511	--	20	**32.325903**	phospholipase C, beta 4		
8	**AK044842**	**135.949852**	**1.617**	0	0	--	--	**--**	9.5 days embryo parthenogenote cDNA, RIKEN full-length enriched library, clone:B130007K04 product:unclassifiable, full insert sequence.		
9	**6330527O06Rik**	**136.057926**	**11.991**	**1**	0.083396	--	--	**--**	RIKEN cDNA 6330527O06 gene		
10	**Lamp5**	**136.057926**	**11.991**	**1**	0.083396	--	--	**--**	lysosomal-associated membrane protein family, member 5 (Lamp5), mRNA.		
11	**Pak7**	**136.081087**	**306.880**	**105**	0.342153	--	20	**7.949947**	p21 protein (Cdc42/Rac)-		

									activated kinase 7		
12 ☐	2900018K06Rik	136.120256	0.465	0	0	--	--	--	RIKEN cDNA 2900018K06 gene		
13 ☐	BC034902	136.501909	13.605	1	0.073502	--	--	--	cDNA sequence BC034902		
14 ☐	Ankrd5	136.532320	23.534	0	0	--	20	74.439111	ankyrin repeat domain 5		
15 ☐	Ankef1	136.532320	23.568	0	0	--	--	--	ankyrin repeat and EF-hand domain containing 1 (Ankef1), mRNA.		
16 ☐	Snap25	136.713449	68.979	2	0.028994	--	20	143.828428	synaptosomal-associated protein 25		
17 ☐	Mkks	136.873780	17.626	1	0.056734	--	20	102.315276	McKusick-Kaufman syndrome		
18 ☐	2210009G21Rik	136.891217	178.432	5	0.028022	--	--	--	RIKEN cDNA 2210009G21 gene		
19 ☐	AK164084	136.892288	2.817	0	0	--	--	--	12 days embryo spinal cord cDNA, RIKEN full-length enriched library, clone:C530005O09 product:unclassifiable, full insert sequence.		
20 ☐	AK148781	136.897096	3.329	1	0.300391	--	--	--	2 days neonate sympathetic ganglion cDNA, RIKEN full-length enriched		

									library, clone:7120446C05 product:unclassifiable, full insert sequence.		
21 ☐	**Slx4ip**	**136.899350**	**170.428**	**5**	0.029338	--	--	**--**	SLX4 interacting protein (Slx4ip), transcript variant 2, mRNA.		

Supplemental Table S5. Candidate genes for Ace2 eQTL in female on Chr 2.

	Index	Symbol	Mb Start	Length (Kb)	SNP Count	SNP Density	Avg Expr	Human Chr	Mb Start (hg19)	Gene Description
☐	1	LOC241572	100.587586	0.161	0	0	--	--	--	similar to Pyruvate kinase, muscle
☐	2	B230118H07Rik	101.560780	60.266	5	0.082966	--	--	--	RIKEN cDNA B230118H07 gene
☐	3	A130042O14Rik	101.600743	3.772	0	0	--	--	--	RIKEN cDNA A1300

	Index	Symbol	Mb Start	Length (Kb)	SNP Count	SNP Density	Avg Expr	Human Chr	Mb Start (hg19)	Gene Description
										42O14 gene
☐	4	Rag2	101.629346	1.584	0	0	--	11	36.570070	recombination activating gene 2
☐	5	Rag1	101.641672	3.123	0	0	--	11	36.546138	recombination activating gene 1
☐	6	Traf6	101.678419	23.249	68	2.924857	--	11	36.467298	TNF receptor-associated factor 6
☐	7	2600010E01Rik	101.714284	83.423	100	1.198710	--	--	--	RIKEN cDNA 2600010E01 gene
☐	8	Prr5l	101.714284	83.423	100	1.198710	--	--	--	proline rich 5 like (Prr5l), transcript variant 1, mRNA.

	Index	Symbol	Mb Start	Length (Kb)	SNP Count	SNP Density	Avg Expr	Human Chr	Mb Start (hg19)	Gene Description
☐	9	AK045896	101.87339 3	1.633	5	3.061849	--	--	--	adult male corpora quadrigemina cDNA, RIKEN full-length enriched libra...
☐	10	Commd9	101.88626 1	15.378	9	0.585252	--	11	36.25208 5	COMM domain containing 9
☐	11	Ldlrad3	101.95020 0	236.260	413	1.748074	--	--	--	low density lipoprotein receptor class A domain containing 3

Supplemental Table S6. Candidate genes for eQTL of Adam17 in male

Index	Symbol	Mb Start	Length (Kb)	SNP Count	SNP Density	Avg Expr	Human Chr	Mb Start (hg19)	Gene Description	PolymiRTS Database >>	Gene Weaver Info Content >>
1	4930591A17Rik	179.414935	1.945	26	13.367609	--	--	--	RIKEN cDNA 4930591A17 gene		
2	Cdh4	179.442430	405.274	1109	2.736420	--	20	102.134202	cadherin 4		
3	4930449I21Rik	179.650732	0.890	0	0	--	--	--	RIKEN cDNA 4930449I21 gene		
4	Taf4a	179.912145	64.501	90	1.395327	--	--	--	TAF4A RNA polymerase II, TATA box binding protein (TBP)-associated factor		
5	Taf4	179.912145	64.501	90	1.395327	--	20	80.246375	TATA-box binding protein associated factor 4 (Taf4), mRNA.		
6	4921531C22Rik	179.976852	2.161	1	0.462749	--	--	--	RIKEN cDNA 4921531C22 gene		
7	Lsm14b	180.024986	10.475	18	1.718377	--	--	--	LSM family member 14B		
8	Psma7	180.036366	6.098	0	0	--	20	60.145179	proteasome (prosome, macropain) subunit, alpha type 7		

9	**Ss18l1**	**180.042482**	**27.719**	**5**	0.180382	--	20	**60.152216**	SS18, nBAF chromatin remodeling complex subunit like 1		
10	**Gtpbp5**	**180.070592**	**15.310**	**3**	0.195950	--	20	**27.656376**	GTP binding protein 5		
11	**Mtg2**	**180.070592**	**15.310**	**3**	0.195950	--	--	**--**	mitochondrial ribosome associated GTPase 2 (Mtg2), transcript variant 3, mRNA.		
12	**Hrh3**	**180.099464**	**4.943**	**1**	0.202306	--	20	**12.977847**	histamine receptor H3		
13	**Osbpl2**	**180.119365**	**39.927**	**18**	0.450823	--	20	**122.921857**	oxysterol binding protein-like 2		
14	**Adrm1**	**180.171587**	**4.696**	**3**	0.638842	--	20	**105.677744**	adhesion regulating molecule 1		
15	**Lama5**	**180.176372**	**49.487**	**57**	1.151818	--	20	**51.330493**	laminin, alpha 5		
16	**Mir7005**	**180.179754**	**0.069**	0	0	--	--	**--**	microRNA 7005 (Mir7005), microRNA.		
17	**Rps21**	**180.257378**	**1.064**	**2**	1.879699	--	20	**136.147461**	ribosomal protein S21		
18	**Mir3091**	**180.257535**	**0.076**	0	0	--	--	**--**	microRNA 3091 (Mir3091), microRNA.		
19	**Cables2**	**180.258538**	**14.927**	**8**	0.535942	--	20	**123.278127**	CDK5 and Abl enzyme substrate 2		
20	**BC066135**	**180.277645**	**12.234**	**14**	1.144352	--	--	**--**	cDNA sequence BC066135		
21	**Rbbp8nl**	**180.277645**	**12.234**	**14**	1.144352	--	--	**--**	RBBP8 N-terminal like (Rbbp8nl), mRNA.		

22	**Gata5**	**180.325087**	**9.592**	**24**	2.502085	--	20	**111.734283**	GATA binding protein 5		
23	**Gata5os**	**180.332856**	**7.876**	**25**	3.174200	--	--	**--**	GATA binding protein 5, opposite strand (Gata5os), long non-coding RNA.		
24	**B230312C02Rik**	**180.370857**	**15.028**	**31**	2.062816	--	--	**--**	RIKEN cDNA B230312C02 gene		
25	**EG622283**	**180.385603**	**16.199**	**68**	4.197790	--	--	**--**	predicted gene, EG622283		
26	**Gm6307**	**180.385603**	**16.199**	**68**	4.197790	--	--	**--**	predicted gene 6307 (Gm6307), long non-coding RNA.		
27	**Mir1a-1**	**180.389047**	**0.077**	0	0	--	--	**--**	microRNA 1a-1 (Mir1a-1), microRNA.		
28	**Mir133a-2**	**180.398378**	**0.104**	0	0	--	--	**--**	microRNA 133a-2 (Mir133a-2), microRNA.		
29	**AK165607**	**180.426754**	**28.083**	**4**	0.142435	--	--	**--**	RCB-0035 WEHI-3 cDNA, RIKEN full-length enriched library, clone:G430047L19 product:unclassifiable, full insert sequence.		
30	**Slco4a1**	**180.456333**	**18.520**	**3**	0.161987	--	20	**31.452489**	solute carrier organic anion transporter family, member 4a1		
31	**Ntsr1**	**180.499975**	**44.244**	**3**	0.067806	--	20	**24.712268**	neurotensin receptor 1		

32	**1600027N09Rik**	**180.581303**	**4.331**	**12**	2.770723	--	--	**--**	RIKEN cDNA 1600027N09 gene			
33	**Mrgbp**	**180.581303**	**4.331**	**12**	2.770723	--	--	**--**	MRG/MORF 4L binding protein (Mrgbp), mRNA.			
34	**LOC552877**	**180.587170**	**1.523**	0	0	--	--	**--**	hypothetical LOC552877			
35	**Ogfr**	**180.589406**	**6.425**	**21**	3.268482	--	20	**22.746192**	opioid growth factor receptor			
36	**Col9a3**	**180.603184**	**10.231**	**27**	2.639038	--	20	**3.583981**	collagen, type IX, alpha 3			
37	**Tcfl5**	**180.621956**	**20.735**	**5**	0.241138	--	20	**154.983125**	transcription factor-like 5 (basic helix-loop-helix)			
38	**Dido1**	**180.680953**	**29.046**	**12**	0.413138	--	--	**--**	death inducer-obliterator 1			
39	**C130092D22Rik**	**180.703199**	**1.493**	0	0	--	--	**--**	RIKEN cDNA C130092D22 gene			
40	**Gid8**	**180.710327**	**11.272**	**3**	0.266146	--	--	**--**	GID complex subunit 8 (Gid8), transcript variant 2, mRNA.			
41	**2310003C23Rik**	**180.712545**	**5.972**	**2**	0.334896	--	--	**--**	RIKEN cDNA 2310003C23 gene			
42	**1700019H03Rik**	**180.725338**	**16.940**	**4**	0.236128	--	--	**--**	RIKEN cDNA 1700019H03 gene			
43	**Slc17a9**	**180.725338**	**16.940**	**4**	0.236128	--	--	**--**	solute carrier family 17, member 9 (Slc17a9), mRNA.			
44	**Bhlhe23**	**180.774380**	**2.520**	0	0	--	--	**--**	basic helix-loop-helix			

									family, member e23 (Bhlhe23), mRNA.		
45	**Bhlhb4**	**180.774657**	**2.243**	0	0	--	--	**--**	basic helix-loop-helix domain containing, class B4		
46	**AK045142**	**180.829489**	**60.224**	**25**	0.415117	--	--	**--**	9.5 days embryo parthenogenote cDNA, RIKEN full-length enriched library, clone:B130039P15 product:unclassifiable, full insert sequence.		
47	**2810461L16Rik**	**180.890683**	**0.283**	0	0	--	--	**--**	RIKEN cDNA 2810461L16 gene		
48	**Mir124a-3**	**180.894039**	**0.068**	0	0	--	--	**--**	microRNA 124a-3 (Mir124a-3), microRNA.		
49	**Ythdf1**	**180.904376**	**16.560**	**4**	0.241546	--	20	**61.297228**	YTH domain family 1		
50	**4833420G11Rik**	**180.915362**	**0.050**	0	0	--	--	**--**	RIKEN cDNA 4833420G11 gene		
51	**Birc7**	**180.929022**	**4.988**	0	0	--	20	**48.430955**	baculoviral IAP repeat-containing 7 (livin)		
52	**Nkain4**	**180.934771**	**19.928**	0	0	--	--	**--**	Na+/K+ transporting ATPase interacting 4		
53	**AK040057**	**180.954463**	**12.349**	**2**	0.161956	--	--	**--**	0 day neonate thymus cDNA, RIKEN full-length enriched		

									library, clone:A430053N14 product:unclassifiable, full insert sequence.		
54	Arfgap1	180.967299	15.225	9	0.591133	--	20	45.039223	ADP-ribosylation factor GTPase activating protein 1		
55	AK030308	180.984231	3.044	3	0.985545	--	--	--	11 days pregnant adult female ovary and uterus cDNA, RIKEN full-length enriched library, clone:5031418M13 product:unclassifiable, full insert sequence.		
56	9230112E08Rik	180.984414	2.857	2	0.700035	--	--	--	RIKEN cDNA 9230112E08 gene		
57	Col20a1	180.986534	31.006	112	3.612204	--	--	--	collagen, type XX, alpha 1		

Supplemental Table S7. Candidate gene for eQTL on chr5 in male for Tmprss2

Index	Symbol	Mb Start	Length (Kb)	SNP Count	SNP Density	Avg	Human Chr	Mb Start (hg19)	Gene Description	PolymiRTS Datab	Gene Weaver

						Expr				ase >>	Info Content >>
1	Gusb	129.989020	13.808	0	0	--	7	64.869824	glucuronidase, beta		
2	Asl	130.011502	12.829	2	0.155897	--	7	64.984963	argininosuccinate lyase		
3	AK212710	130.024182	0.068	0	0	--	--	--	cDNA, clone:Y2G0125N20, strand:unspecified.		
4	Crcp	130.029305	31.478	0	0	--	--	--	calcitonin gene-related peptide-receptor component protein		
5	Tpst1	130.079369	56.364	5	0.088709	--	7	65.114463	protein-tyrosine sulfotransferase 1		
6	2210412B16Rik	130.131326	0.498	0	0	--	--	--	RIKEN cDNA 2210412B16 gene		
7	4930487N04Rik	130.143318	1.357	0	0	--	--	--	RIKEN cDNA 4930487N04 gene		
8	Kctd7	130.144887	10.921	3	0.274700	--	7	65.538094	potassium channel tetramerisation domain containing 7		
9	Rabgef1	130.171818	42.519	115	2.704673	--	7	65.649869	RAB guanine nucleotide exchange factor (GEF) 1		
10	0610007L01Rik	130.219743	24.022	20	0.832570	--	--	--	RIKEN cDNA 0610007L01 gene		
11	Tmem248	130.219743	24.022	20	0.832570	--	--	--	transmembrane protein 248 (Tmem248), transcript variant 2, mRNA.		
12	EG625540	130.220458	2.607	5	1.917913	--	--	--	predicted gene, EG625540		

13	**Sbds**	**130.245731**	**9.799**	**7**	0.714359	--	7	**65.896839**	SBDS ribosome assembly guanine nucleotide exchange factor		
14	**Tyw1**	**130.257036**	**84.531**	**130**	1.537897	--	--	**--**	tRNA-yW synthesizing protein 1 homolog (S. cerevisiae)		
15	**A330070K13Rik**	**130.378850**	**5.781**	**2**	0.345961	--	--	**--**	RIKEN cDNA A330070K13 gene		
16	**Caln1**	**130.448124**	**392.521**	**15**	0.038215	--	7	**70.692565**	calneuron 1		
17	**C030007I01Rik**	**130.653423**	**0.050**	0	0	--	--	**--**	RIKEN cDNA C030007I01 gene		
18	**Wbscr17**	**130.874950**	**432.572**	**9**	0.020806	--	7	**70.042439**	Williams-Beuren syndrome chromosome region 17 homolog (human)		
19	**Gcap8**	**130.958922**	**1.113**	0	0	--	--	**--**	granule cell antiserum positive 8		
20	**LOC545809**	**131.391600**	**23.118**	0	0	--	--	**--**	hypothetical protein LOC545809		
21	**Auts2**	**131.437681**	**37.492**	**6**	0.160034	--	7	**68.508556**	autism susceptibility candidate 2		
22	**3110001N23Rik**	**131.524950**	**0.713**	0	0	--	--	**--**	RIKEN cDNA 3110001N23 gene		
23	**9330177L23Rik**	**131.593811**	**1.100**	0	0	--	--	**--**	RIKEN cDNA 9330177L23 gene		
24	**2810432F15Rik**	**131.624841**	**0.700**	0	0	--	--	**--**	RIKEN cDNA 2810432F15 gene		
25	**4930563F08Rik**	**131.880268**	**3.500**	**3**	0.857143	--	--	**--**	RIKEN cDNA 4930563F08 gene		
26	**C230071H17Rik**	**131.882965**	**2.161**	**1**	0.462749	--	--	**--**	RIKEN cDNA C230071H17 gene		

27	AL023051	131.93 2395	2.28 4	0	0	--	--	--	expressed sequence AL023051		
28	AK140583	131.98 8009	4.07 6	0	0	--	--	--	10 days neonate cerebellum cDNA, RIKEN full-length enriched library, clone:B930034J 01 product:hypothe tical protein, full insert sequence.		
29	2610011E 03Rik	131.99 6178	2.23 5	0	0	--	--	--	RIKEN cDNA 2610011E03 gene		
30	AK076755	133.53 4589	63.8 17	2	0.03 1340	--	--	--	adult male testis cDNA, RIKEN full-length enriched library, clone:49304 32F18 product:uncl assifiable, full insert sequence.		

Supplemental Table S8. Top 100 genes in female for 3 probes in Ace2

	Dataset	Trait ID	Symbol	Description	Location	Mean	N Cases	Max LRS	Max LRS Location Chr and Mb	Add
1	MA_M2F_0706_R	1425103_at	*Ace2*	angiotensin I converting enzyme (peptidyl-dipeptidase A) 2; middle to distal 3'-UTR	ChrX: 164.187853	10.112	65	17.2	Chr16: 73.747956	-0.131
2	MA_M2F_0706_R	1425102_a_at	*Ace2*	angiotensin I converting enzyme (peptidyl-dipeptidase A) 2; 3'-UTR and last 4 exons	ChrX: 164.182645	10.046	65	12.7	Chr14: 3.000000	0.186
3	MA_M2F_0706_R	1417530_a_at	*Srp9*	signal recognition particle 9	Chr1: 182.131342	11.639	65	11.0	Chr8: 25.520489	-0.102
4	MA_M2F_0706_R	1422789_at	*Aldh1a2*	aldehyde dehydrogenase family 1, subfamily A2; 3' UTR	Chr9: 71.295684	7.918	65	18.5	Chr17: 21.686210	-0.180
5	MA_M2F_0706_R	1415683_at	*Nmt1*	N-myristoyltransferase 1; exons 10 and 11, and 3' UTR	Chr11: 103.064733	11.775	65	11.8	Chr10: 24.000000	0.085
6	MA_M2F_0706_R	1434037_s_at	*Kat2b*	K(lysine) acetyltransferase 2B (p300/CREB-binding protein-associated factor); distal 3' UTR	Chr17: 53.672145	11.464	65	14.0	Chr10: 19.937470	0.095
7	MA_M2F_0706_R	1428969_at	*Kiaa1012*	protein TRS85 homolog (ER-Golgi transport); 3' UTR	Chr18: 20.817479	11.054	65	11.4	Chr14: 3.000000	0.101
8	MA_M2F_0706_R	1416678_at	*Cops3*	COP9 (constitutive photomorphogenic) homolog, subunit 3; last four exons and 3' UTR	Chr11: 59.817915	11.640	65	9.5	Chr9: 107.639250	0.087
9	MA_M2F_0706_R	1416472_at	*Syap1*	synapse associated protein 1; mid-distal 3' UTR	ChrX: 162.857076	12.641	65	9.9	Chr16: 85.961783	-0.079

10 ☐	MA_M2F_0706_R	1453880_s_at	*1700041C02Rik*	RIKEN cDNA 1700041C02 gene	Chr4: 119.377209	5.871	65	16.6	Chr9: 88.498812	0.035
11 ☐	MA_M2F_0706_R	1437391_x_at	*Mrpl44*	mitochondrial ribosomal protein L44	Chr1: 79.781249	11.205	65	26.3	Chr15: 89.576205	0.191
12 ☐	MA_M2F_0706_R	1423486_at	*Cript*	postsynaptic protein CRIPT (cysteine-rich PDZ-binding protein); last several exons and 3' UTR	Chr17: 87.034276	12.203	65	10.2	Chr15: 87.476581	0.079
13 ☐	MA_M2F_0706_R	1415749_a_at	*Rragc*	Ras-related GTP binding C; mid-distal 3' UTR	Chr4: 123.936450	12.155	65	16.6	Chr9: 88.498812	0.093
14 ☐	MA_M2F_0706_R	1420138_at	*Slc19a1*	solute carrier family 19 (sodium/hydrogen exchanger), member 1	Chr10: 77.050144	11.889	65	9.5	Chr15: 95.949674	0.113
15 ☐	MA_M2F_0706_R	1452086_at	*2610027O18Rik*	RIKEN cDNA 2610027O18 gene	Chr12: 73.280555	7.543	65	13.9	Chr9: 99.750326	0.052
16 ☐	MA_M2F_0706_R	1424209_at	*Rarsl*	arginyl-tRNA synthetase-like; exons 15, 16, 17, 18 19, 20	Chr4: 34.656065	10.679	65	14.4	Chr2: 170.826972	0.084
17 ☐	MA_M2F_0706_R	1448238_at	*C14orf166*	CLE7 homolog (UPF0568 protein); 3' half	Chr14: 19.812115	12.645	65	11.2	Chr16: 73.747956	-0.086
18 ☐	MA_M2F_0706_R	1417054_a_at	*Sf3b14*	splicing factor 3B, 14 kDa subunit; last 2 exons and proximal 3' UTR	Chr12: 4.826831	12.053	65	10.6	Chr15: 91.405127	0.147
19 ☐	MA_M2F_0706_R	1448206_at	*Psma2*	proteasome (prosome, macropain) subunit, alpha type 2; last five exons and 3' UTR	Chr13: 14.619304	13.033	65	9.7	Chr16: 74.917702	-0.079
20 ☐	MA_M2F_0706_R	1421118_a_at	*Gpr56*	G protein-coupled receptor 56; 3' UTR	Chr8: 95.013029	11.029	65	11.5	Chr19: 15.292517	0.172
21 ☐	MA_M2F_0706_R	1437450_x_at	*C14orf166*	CLE7 homolog (UPF0568 protein)	Chr14: 19.811971	12.200	65	13.4	Chr16: 73.747956	-0.104

22 ☐	MA_M2F_0706_R	1433545_s_at	*Acad11*	acyl-Coenzyme A dehydrogenase family, member 11; mid distal 3' UTR	Chr9: 104.127137	13.085	65	14.4	Chr9: 99.750326	0.129
23 ☐	MA_M2F_0706_R	1434665_at	*Aga*	aspartylglucosaminidase; exons 6, 7, and 8	Chr8: 53.521136	10.853	65	11.1	Chr9: 88.498812	0.110
24 ☐	MA_M2F_0706_R	1434900_at	*Mkl1*	MKL (megakaryoblastic leukemia)/myocardin-like 1; mid distal 3' UTR	Chr15: 81.012534	9.424	65	16.6	Chr15: 91.405127	-0.104
25 ☐	MA_M2F_0706_R	1423711_at	*Ndufaf1*	NADH dehydrogenase (ubiquinone) 1 alpha subcomplex, assembly factor 1; last 3 exons and 3' UTR	Chr2: 119.655607	9.734	65	11.6	Chr14: 3.000000	0.134
26 ☐	MA_M2F_0706_R	1424700_at	*Tmem38b*	transmembrane protein 38B (trimeric intracellular cation channel type B); last exon	Chr4: 53.859998	12.660	65	13.8	Chr16: 73.747956	0.124
27 ☐	MA_M2F_0706_R	1424828_a_at	*Fh1*	fumarate hydratase, mitochondrial (fumarase); exons 8, 9, and 10, and proximal 3' UTR	Chr1: 175.601417	13.455	65	12.0	Chr1: 163.338902	0.068
28 ☐	MA_M2F_0706_R	1416596_at	*Slc44a4*	solute carrier family 44, member 4 (choline transporter-like); last five exons	Chr17: 34.928445	12.257	65	11.7	Chr9: 107.639250	0.119
29 ☐	MA_M2F_0706_R	1448102_a_at	*Wdr61*	WD repeat domain 61; mid proximal 3' UTR	Chr9: 54.717273	11.646	65	9.4	Chr4: 96.896949	0.109
30 ☐	MA_M2F_0706_R	1415783_at	*Vps35*	vacuolar protein sorting 35; 3' UTR	Chr8: 85.260458	13.460	65	11.6	Chr16: 73.747956	-0.090
31 ☐	MA_M2F_0706_R	1452737_at	*Smim15*	small integral membrane protein 15; 3' UTR	Chr13: 108.048608	11.910	65	10.4	Chr10: 116.640226	-0.115
32 ☐	MA_M2F_0706_R	1460131_at	*2810040C05Rik*	RIKEN cDNA 2810040C05 gene	Chr8: 26.131805	6.936	65	12.6	Chr9: 99.750326	-0.048

33 ☐	MA_M2F_0706_R	1430500_s_at	*Mtx2*	metaxin 2; 3 exons	Chr2: 74.869348	11.466	65	15.1	Chr4: 96.896949	0.137
34 ☐	MA_M2F_0706_R	1416270_at	*Polr2g*	polymerase (RNA) II (DNA directed) polypeptide G; exons 3, 4, 5, 7, and 3' UTR	Chr19: 8.793198	11.581	65	10.6	Chr16: 89.532874	-0.080
35 ☐	MA_M2F_0706_R	1417710_at	*Mettl9*	methyltransferase like 9; last exon and proximal half of 3' UTR	Chr7: 121.076249	12.659	65	11.3	Chr1: 73.418156	0.085
36 ☐	MA_M2F_0706_R	1421185_at	*UbiE2*	RIKEN cDNA 3300001H21 gene	Chr15: 100.328217	7.776	65	13.4	Chr9: 94.904747	-0.095
37 ☐	MA_M2F_0706_R	1416096_at	*AI413782*	protein LOC63894; mid 3' UTR	Chr12: 87.239058	10.350	65	9.7	Chr9: 107.824821	0.081
38 ☐	MA_M2F_0706_R	1451719_at	*Crsp6*	cofactor required for Sp1 transcriptional activation, subunit 6	Chr9: 15.260462	6.667	65	15.3	Chr16: 17.412078	0.058
39 ☐	MA_M2F_0706_R	1428645_at	*Gnai3*	guanine nucleotide binding protein, alpha inhibiting 3; distal 3' UTR	Chr3: 108.107540	11.397	65	14.1	Chr3: 134.523036	-0.140
40 ☐	MA_M2F_0706_R	1434587_x_at	*Ptdss2*	phosphatidylserine synthase 2	Chr7: 141.155733	10.985	65	11.4	Chr4: 141.236455	-0.065
41 ☐	MA_M2F_0706_R	1438018_at	*Hook1*	hook 1 (microtubule linker, endocytic membrane trafficking); distal 3' UTR	Chr4: 96.024792	11.882	65	12.1	Chr15: 91.405127	0.126
42 ☐	MA_M2F_0706_R	1455286_at	*Btbd1*	BTB (POZ) domain containing 1; distal 3' UTR	Chr7: 81.792103	12.326	65	10.4	Chr16: 88.032782	-0.085
43 ☐	MA_M2F_0706_R	1456015_x_at	*Ndufv1*	NADH dehydrogenase (ubiquinone) flavoprotein 1; last exon and 3' UTR	Chr19: 4.007528	14.611	65	14.0	Chr3: 129.482611	-0.098
44 ☐	MA_M2F_0706_R	1429088_at	*Lbh*	limb bud and heart development (lupus brain antigen 1); distal 3' UTR	Chr17: 72.941355	9.300	65	10.8	Chr6: 44.030269	0.158

45 ☐	MA_M2F_0706_R	1449135_at	*Sox18*	SRY-box containing gene 18	Chr2: 181.670065	9.162	65	12.3	Chr16: 88.032782	0.104
46 ☐	MA_M2F_0706_R	1421019_at	*1700021F05Rik*	RIKEN cDNA 1700021F05; exons 1 and 3 and 3' UTR	Chr10: 43.525190	10.835	65	17.9	Chr15: 91.405127	0.148
47 ☐	MA_M2F_0706_R	1431423_a_at	*Med8*	mediator of RNA polymerase II transcription, subunit 8 homolog; last 2 exons and proximal half of 3' UTR	Chr4: 118.413625	10.483	65	11.9	Chr4: 102.851020	0.103
48 ☐	MA_M2F_0706_R	1421164_a_at	*Arhgef1*	Rho guanine nucleotide exchange factor (GEF) 1	Chr7: 24.925880	10.459	65	11.7	Chr4: 81.726726	-0.094
49 ☐	MA_M2F_0706_R	1434038_at	*Dnajc13*	DnaJ (Hsp40) homolog, subfamily C, member 13	Chr9: 104.151791	11.426	65	16.1	Chr9: 99.750326	0.117
50 ☐	MA_M2F_0706_R	1421104_at	*Mpa2*	macrophage activation 2	Chr10: 77.268946	7.246	65	10.7	Chr19: 23.135216	-0.054
51 ☐	MA_M2F_0706_R	1434447_at	*Met*	met proto-oncogene; distal 3' UTR	Chr6: 17.573398	11.796	65	12.1	Chr19: 14.967297	-0.124
52 ☐	MA_M2F_0706_R	1420776_a_at	*Auh*	AU RNA binding protein/enoyl-coenzyme A hydratase (mitochondrial, 3-methylglutaconic aciduria, type I); last four exons and proximal half of 3' UTR	Chr13: 52.835320	12.010	65	16.4	Chr13: 51.479103	0.104
53 ☐	MA_M2F_0706_R	1435966_x_at	*Mrpl13*	mitochondrial ribosomal protein L13; Proximal, mid 3' UTR, and intorn	Chr15: 55.534119	7.208	65	13.9	Chr18: 82.240604	-0.123
54 ☐	MA_M2F_0706_R	1422880_at	*Sypl*	synaptophysin-like protein; distal 3' UTR	Chr12: 32.976333	13.122	65	11.5	Chr4: 81.958676	0.117
55 ☐	MA_M2F_0706_R	1460745_at	*A630098A13Rik*	RIKEN cDNA A630098A13 gene	Chr14: 53.961232	6.670	65	10.2	Chr7: 89.123287	-0.039

56 ☐	MA_M2F_0706_R	1436139_at	*Mdga2*	MAM domain containing glycosylphosphatidylinositol anchor 2; putative far 3' UTR element	Chr12: 66.466121	5.517	65	14.0	Chr6: 3.266392	0.023
57 ☐	MA_M2F_0706_R	1452768_at	*Tex261*	testis expressed gene 261	Chr6: 83.769296	10.962	65	9.9	Chr16: 67.046635	0.101
58 ☐	MA_M2F_0706_R	1450699_at	*Selenbp1*	selenium binding protein 1	Chr3: 94.944489	14.224	65	21.8	Chr9: 99.750326	0.113
59 ☐	MA_M2F_0706_R	1423651_at	*Isca1*	iron-sulfur cluster assembly 1 homolog; distal 3' UTR	Chr13: 21.496566	13.170	65	11.7	Chr9: 107.824821	0.079
60 ☐	MA_M2F_0706_R	1422854_at	*Shc1*	src homology 2 domain-containing transforming protein C1	Chr3: 89.429142	10.818	65	15.8	Chr15: 91.405127	-0.184
61 ☐	MA_M2F_0706_R	1415966_a_at	*Ndufv1*	NADH dehydrogenase (ubiquinone) flavoprotein 1; last exon	Chr19: 4.007602	14.081	65	10.1	Chr16: 73.747956	-0.097
62 ☐	MA_M2F_0706_R	1424130_a_at	*Ptrf*	polymerase I and transcript release factor	Chr11: 100.957029	8.882	65	9.6	Chr16: 70.194758	0.178
63 ☐	MA_M2F_0706_R	1423781_at	*Appbp1*	amyloid beta precursor protein binding protein 1; exons 16, 18, 19, 20 and proximal 3' UTR	Chr8: 104.513201	10.361	65	15.9	Chr4: 96.896949	0.134
64 ☐	MA_M2F_0706_R	1420495_a_at	*Vps26*	vacuolar protein sorting 26 (retromer complex, involved in retrograde transport of proteins from endosomes to the trans-Golgi network); distal 3' UTR	Chr10: 62.455304	11.491	65	17.2	Chr5: 99.299507	0.078
65 ☐	MA_M2F_0706_R	1452005_at	*Dlat*	dihydrolipoamide S-acetyltransferase (E2 component of pyruvate dehydrogenase complex); distal 3' UTR	Chr9: 50.634731	10.157	65	10.7	Chr9: 40.213083	-0.136

66 ☐	MA_M2F_0706_R	1456748_a_at	*Nipsnap1*	4-nitrophenylphosphatase domain and non-neuronal SNAP25-like protein homolog 1; distal 3' UTR	Chr11: 4.894050	11.565	65	11.3	Chr9: 83.778825	0.146
67 ☐	MA_M2F_0706_R	1426256_at	*Timm17a*	translocator of inner mitochondrial membrane 17a	Chr1: 135.301708	12.121	65	14.7	Chr15: 89.576205	0.114
68 ☐	MA_M2F_0706_R	1415813_at	*Api5*	apoptosis inhibitor 5; distal 3' UTR (transQTL on Chr 4 in BXD eye data)	Chr2: 94.411933	10.893	65	10.9	Chr2: 102.660525	-0.093
69 ☐	MA_M2F_0706_R	1426529_a_at	*Tagln2*	transgelin 2; mid and distal 3' UTR	Chr1: 172.506826	12.074	65	22.4	Chr1: 172.235364	-0.174
70 ☐	MA_M2F_0706_R	1417633_at	*Sod3*	superoxide dismutase 3, extracellular	Chr5: 52.368952	14.321	65	11.6	ChrX: 102.126656	-0.170
71 ☐	MA_M2F_0706_R	1435178_x_at	*Anapc5*	anaphase-promoting complex subunit 5	Chr5: 11.351956	12.933	65	10.7	Chr1: 178.015151	0.065
72 ☐	MA_M2F_0706_R	1438208_at	*1110033K02Rik*	ESTs, Highly similar to serine/threonine protein kinase TAO2 [Rattus norvegicus] [R.norvegicus]	Chr7: 126.869685	8.377	65	8.5	Chr15: 89.576205	-0.141
73 ☐	MA_M2F_0706_R	1424918_at	*Tbc1d19*	TBC1 domain family, member 19	Chr5: 53.887402	10.323	65	13.8	ChrX: 71.441039	-0.142
74 ☐	MA_M2F_0706_R	1446188_at	*Lrriq2*	leucine-rich repeats and IQ motif containing 2	Chr16: 55.918903	7.194	65	13.3	Chr9: 88.498812	-0.047
75 ☐	MA_M2F_0706_R	1452812_at	*Lphn1*	latrophilin 1	Chr8: 83.939489	8.548	65	16.5	Chr4: 86.450967	-0.133
76 ☐	MA_M2F_0706_R	1415867_at	*Cct4*	chaperonin subunit 4 (delta); exons 12 and 13 (of 14), long isoform	Chr11: 23.002199	12.718	65	14.7	Chr4: 75.824944	0.093

77	MA_M2F_0706_R	1439029_at	*Gpt2*	glutamic pyruvate transaminase (alanine aminotransferase) 2	Chr8: 85.523253	9.944	65	14.3	Chr19: 23.853320	0.212
78	MA_M2F_0706_R	1452662_a_at	*Eif2s1*	eukaryotic translation initiation factor 2, subunit 1 alpha; last two exons and proximal half of 3' UTR	Chr12: 78.884789	10.839	65	10.6	Chr16: 89.532874	-0.085
79	MA_M2F_0706_R	1450243_a_at	*Dscr1l1*	Down syndrome critical region gene 1-like 1; last three exons	Chr17: 44.017779	6.708	65	15.1	Chr15: 91.405127	-0.089
80	MA_M2F_0706_R	1460295_s_at	*Il6st*	interleukin 6 signal transducer; proximal to mid 3' UTR	Chr13: 112.505179	9.448	65	11.2	Chr15: 91.405127	-0.173
81	MA_M2F_0706_R	1448736_a_at	*Hprt*	hypoxanthine guanine phosphoribosyl transferase; last exon and 3' UTR	ChrX: 53.021104	11.706	65	9.4	Chr2: 83.413222	-0.066
82	MA_M2F_0706_R	1455604_at	*AI427138*	expressed sequence AI427138; distal 3' UTR	Chr1: 64.730740	8.702	65	8.7	Chr14: 48.527624	0.115
83	MA_M2F_0706_R	1431665_a_at	*Timm8b*	translocase of inner mitochondrial membrane 8 homolog b (yeast); 5' UTR, both exons and proximal 3'	Chr9: 50.604007	12.782	65	11.1	Chr4: 96.896949	0.125
84	MA_M2F_0706_R	1435636_at	*Ago2*	argonaute RISC catalytic component 2 (eukaryotic translation initiation factor 2C, 2); distal 3' UTR	Chr15: 73.099051	9.907	65	14.1	Chr15: 89.576205	-0.123
85	MA_M2F_0706_R	1423044_at	*Prosc*	proline synthetase co-transcribed	Chr8: 27.054003	12.857	65	13.7	Chr9: 99.750326	0.096
86	MA_M2F_0706_R	1448317_at	*Tmem128*	transmembrane protein 128; mid 3' UTR	Chr5: 38.269083	9.653	65	12.3	Chr19: 37.868206	-0.145
87	MA_M2F_0706_R	1424681_a_at	*Psma5*	proteasome (prosome, macropain) subunit, alpha type 5; putative exon (transQTL on Chr 4 in BXD eye data)	Chr3: 108.267833	11.233	65	11.1	Chr8: 85.923280	-0.069

88 ☐	MA_M2F_0706_R	1451521_x_at	*Wbscr1*	Williams-Beuren syndrome chromosome region 1; distal 3' UTR	Chr5: 134.620018	11.837	65	10.6	Chr11: 36.172994	0.068
89 ☐	MA_M2F_0706_R	1450159_at	*Rem1*	rad and gem related GTP binding protein 1	Chr2: 152.634893	6.891	65	15.6	Chr2: 107.153805	0.067
90 ☐	MA_M2F_0706_R	1416058_s_at	*Atp5c1*	ATP synthase, H+ transporting, mitochondrial F1 complex, gamma polypeptide 1; last two exons	Chr2: 10.059028	15.595	65	6.9	Chr2: 176.000000	0.069
91 ☐	MA_M2F_0706_R	1433535_x_at	*Cct2*	chaperonin subunit 2 (beta)	Chr10: 103.893749	12.756	65	16.1	Chr16: 63.773114	-0.107
92 ☐	MA_M2F_0706_R	1417652_a_at	*Tbca*	tubulin cofactor a; exon 3 and last intron	Chr13: 94.837108	12.976	65	9.8	Chr15: 91.405127	0.116
93 ☐	MA_M2F_0706_R	1416163_at	*Cops4*	COP9 (constitutive photomorphogenic) homolog, subunit 4; last two exons and proximal 3' UTR	Chr5: 100.543853	10.157	65	10.6	Chr14: 19.755208	0.104
94 ☐	MA_M2F_0706_R	1416859_at	*Fkbp3*	FK506 binding protein 3; last four exons	Chr12: 65.063678	10.787	65	16.2	Chr3: 134.523036	-0.122
95 ☐	MA_M2F_0706_R	1417573_at	*Mmadhc*	methylmalonic aciduria (cobalamin deficiency) cblD type, with homocystinuria; last two exons and 3' UTR	Chr2: 50.279951	11.670	65	10.5	Chr3: 134.523036	-0.097
96 ☐	MA_M2F_0706_R	1416668_at	*Ttc35*	tetratricopeptide repeat domain 35 (nonmembrane-spanning 40 kD 0-linked glycosyltransferase); three exons	Chr15: 43.527518	11.951	65	13.1	Chr15: 91.405127	0.124
97 ☐	MA_M2F_0706_R	1457677_at	*C920016K16Rik*	RIKEN cDNA C920016K16; distal 3' UTR	Chr17: 33.004869	8.038	65	12.4	Chr1: 191.333382	-0.061

98 ☐	MA_M2F_0706_R	1453731_a_at	*Tmem77*	RIKEN cDNA 2610318G18; last two exons and proximal 3' UTR	Chr3: 106.573608	12.404	65	11.3	Chr9: 3.400000	-0.094
99 ☐	MA_M2F_0706_R	1436215_at	*Ipmk*	inositol polyphosphate multikinase; distal 3' UTR	Chr10: 71.385472	10.813	65	13.8	Chr15: 91.405127	0.140
100 ☐	MA_M2F_0706_R	1423459_at	*Cops2*	COP9 (constitutive photomorphogenic) homolog, subunit 2; last exon and proximal 3' UTR	Chr2: 125.832025	10.439	65	13.5	Chr1: 76.451132	0.118
101 ☐	MA_M2F_0706_R	1418817_at	*Chmp1b*	chromatin modifying protein 1B	Chr18: 67.207239	10.045	65	14.9	Chr14: 27.000000	0.205
102 ☐	MA_M2F_0706_R	1419984_s_at	*Zfp644*	zinc finger protein 644; mid to distal 3' UTR	Chr5: 106.617110	9.828	65	12.3	Chr11: 83.064807	0.111
103 ☐	MA_M2F_0706_R	1416653_at	*Stxbp3a*	syntaxin binding protein 3a; proximal 3' UTR	Chr3: 108.793540	9.536	65	8.2	Chr8: 80.868085	-0.089
104 ☐	MA_M2F_0706_R	1452785_at	*C18orf55*	human chromosome 18 open reading frame 55; last exons and 3' UTR (test Mendelian 10.86 in BXD)	Chr18: 84.947626	9.571	65	14.0	Chr14: 3.000000	0.132
105 ☐	MA_M2F_0706_R	1424642_at	*Thoc1*	THO complex 1	Chr18: 9.992178	9.675	65	14.7	Chr2: 181.014276	0.121
106 ☐	MA_M2F_0706_R	1452676_a_at	*Pnpt1*	polyribonucleotide nucleotidyltransferase 1; last four exons including proximal 3' UTR	Chr11: 29.159348	9.388	65	15.7	Chr11: 24.905498	-0.186
107 ☐	MA_M2F_0706_R	1417122_at	*Vav3*	vav 3 oncogene (Rho family guanine nucleotide exchange factor); mid distal 3' UTR	Chr3: 109.685110	8.002	65	9.6	Chr7: 84.149847	0.156
108 ☐	MA_M2F_0706_R	1435127_a_at	*Osgepl1*	O-sialoglycoprotein endopeptidase-like 1	Chr1: 53.320085	9.391	65	10.8	Chr16: 3.500000	-0.115

109	MA_M2F_0706_R	1421022_x_at	*Acyp1*	acylphosphatase 1, erythrocyte (common) type	Chr12: 85.272453	10.369	65	12.6	Chr4: 96.896949	0.180
110	MA_M2F_0706_R	1426473_at	*Dnajc9*	DnaJ (Hsp40) homolog, subfamily C, member 9	Chr14: 20.385026	8.961	65	10.9	Chr15: 68.818097	-0.094
111	MA_M2F_0706_R	1424324_at	*A930014I12Rik*	RIKEN cDNA A930014I12 gene	Chr18: 10.566702	9.520	65	17.3	Chr11: 83.064807	0.187
112	MA_M2F_0706_R	1448896_at	*Pigf*	phosphatidylinositol glycan, class F	Chr17: 86.997281	9.622	65	13.3	Chr11: 83.064807	0.156
113	MA_M2F_0706_R	1448555_at	*D15Ertd682e*	DNA segment, Chr 15, ERATO Doi 682, expressed	Chr15: 97.678396	8.509	65	12.8	Chr14: 9.528965	0.122
114	MA_M2F_0706_R	1460258_at	*Lect1*	leukocyte cell derived chemotaxin 1	Chr14: 79.637747	6.847	65	14.1	Chr16: 3.500000	0.090
115	MA_M2F_0706_R	1421202_at	*Chrna4*	cholinergic receptor, nicotinic, alpha 4 (high affinity nicotine receptor with Chrnb2); last two exons and proximal 3' UTR	Chr2: 181.024702	8.688	65	10.9	Chr13: 3.150000	-0.100
116	MA_M2F_0706_R	1417140_a_at	*Ptpn2*	protein tyrosine phosphatase, non-receptor type 2; last exon and 3' UTR	Chr18: 67.665655	8.719	65	14.9	Chr14: 9.528965	0.147
117	MA_M2F_0706_R	1424230_at	*Exoc6*	exocyst complex component 6	Chr19: 37.682952	8.747	65	13.8	ChrX: 61.058643	-0.143
118	MA_M2F_0706_R	1434404_at	*Fam73a*	family with sequence similarity 73, member A	Chr3: 152.274018	7.884	65	13.8	Chr14: 3.000000	0.145
119	MA_M2F_0706_R	1427197_at	*Atr*	ataxia telangiectasia and rad3 related; last four coding exons	Chr9: 95.945370	7.738	65	12.3	Chr3: 39.650830	-0.160
120	MA_M2F_0706_R	1435682_at	*Lars2*	leucyl-tRNA synthetase, mitochondrial; distal 3' UTR (test Mendelian	Chr9: 123.462289	9.884	65	11.9	Chr14: 3.000000	0.093

				1.04, apparent transQTL)						
121 ☐	MA_M2F_ 0706_R	[1426269 _at]	*Sybl1*	synaptobrevin like 1 (pseudoautosomal region); distal 3' UTR	ChrX: 1.000000	10.8 34	65	9.4	Chr16: 3.500000	- 0.15 2
122 ☐	MA_M2F_ 0706_R	[1415981 _at]	*Herpud2*	HERPUD family member 2; 3' UTR	Chr9: 25.108158	10.2 15	65	10.7	Chr11: 83.064807	0.15 6
123 ☐	MA_M2F_ 0706_R	[1419641 _at]	*Purb*	purine rich element binding protein B	Chr11: 6.473439	9.15 2	65	16.5	Chr14: 3.000000	0.19 1
124 ☐	MA_M2F_ 0706_R	[1452138 _a_at]	*Ace2*	angiotensin I converting enzyme (peptidyl-dipeptidase A) 2; last 2~6 and 11 exons	ChrX: 164.167855	10.0 33	65	12.1	Chr14: 3.000000	0.15 6
125 ☐	MA_M2F_ 0706_R	[1419065 _at]	*5730521E 12Rik*	RIKEN cDNA 5730521E12 gene	Chr10: 52.404060	8.44 2	65	10.1	Chr14: 48.527624	0.22 4
126 ☐	MA_M2F_ 0706_R	[1428551 _at]	*Trmt11*	tRNA methyltransferase 11 homolog; last 2 exons and 3' UTR	Chr10: 30.534740	7.83 3	65	12.8	Chr11: 83.064807	0.11 6
127 ☐	MA_M2F_ 0706_R	[1434607 _at]	*Ddx52*	DEAD (Asp-Glu-Ala-Asp) box polypeptide 52	Chr11: 83.959487	7.08 3	65	13.2	Chr14: 9.528965	0.18 7
128 ☐	MA_M2F_ 0706_R	[1452385 _at]	*Usp53*	ubiquitin specific protease 53	Chr3: 122.931918	6.64 7	65	13.8	Chr14: 114.751225	0.17 5
129 ☐	MA_M2F_ 0706_R	[1439334 _at]	*A330009 G12*	ESTs	ChrX: 141.727603	5.53 3	65	10.8	Chr14: 9.528965	0.03 6
130 ☐	MA_M2F_ 0706_R	[1448922 _at]	*Dusp19*	dual specificity phosphatase 19	Chr2: 80.621210	7.16 2	65	15.1	Chr14: 24.000000	0.15 3
131 ☐	MA_M2F_ 0706_R	[1448703 _at]	*Lsm8*	LSM8 homolog, U6 small nuclear RNA associated (S. cerevisiae)	Chr6: 18.851648	9.28 7	65	12.2	Chr9: 29.940961	- 0.17 0
132 ☐	MA_M2F_ 0706_R	[1452090 _a_at]	*Olfm3*	olfactomedin 3; distal 3' UTR	Chr3: 115.124528	7.78 3	65	11.2	Chr14: 30.957748	0.23 4

133 ☐	MA_M2F_0706_R	1451968_at	*Xrcc5*	X-ray repair complementing defective repair in Chinese hamster cells 5; last five exons	Chr1: 72.381622	9.455	65	11.2	Chr8: 25.520489	-0.096
134 ☐	MA_M2F_0706_R	1452239_at	*Gt(ROSA)26Sor*	gene trap ROSA 26, Philippe Soriano (between Thumpd3 and Setd5); putative 3' end exon or 3' UTR, expressed even in normal mice	Chr6: 113.070606	7.082	65	13.9	Chr11: 83.064807	0.193
135 ☐	MA_M2F_0706_R	1460430_at	*Rap2c*	RAP2C, member of RAS oncogene family	ChrX: 51.004526	8.210	65	13.6	Chr14: 3.000000	0.183
136 ☐	MA_M2F_0706_R	1428829_at	*Dennd1b*	DENN/MADD domain containing 1B; putative far 3' UTR	Chr1: 139.175456	9.165	65	13.4	Chr14: 9.528965	0.186
137 ☐	MA_M2F_0706_R	1417506_at	*Gmnn*	geminin (cell cycle regulatory, replication inhibition); last three exons and proximal 3' UTR	Chr13: 24.751994	8.534	65	9.2	Chr4: 156.121747	0.093
138 ☐	MA_M2F_0706_R	1434682_at	*Zfp770*	zinc finger protein 770; distal 3' UTR	Chr2: 114.193761	7.485	65	16.6	Chr11: 83.064807	0.106
139 ☐	MA_M2F_0706_R	1425495_at	*Zfp62*	zinc finger protein 62	Chr11: 49.218192	6.712	65	18.6	Chr14: 3.000000	0.193
140 ☐	MA_M2F_0706_R	1428629_at	*6330417C12Rik*	RIKEN cDNA 6330417C12 gene	Chr19: 40.917374	7.183	65	10.0	Chr8: 80.868085	-0.150
141 ☐	MA_M2F_0706_R	1435828_at	*Maf*	musculoaponeurotic fibrosarcoma (v-maf) AS42 transcription factor proto-oncogene; distal 3' UTR	Chr8: 115.683054	8.048	65	9.9	Chr14: 19.755208	0.155
142 ☐	MA_M2F_0706_R	1439266_a_at	*Polr3k*	polymerase (RNA) III (DNA directed) polypeptide K	Chr2: 181.868495	7.426	65	16.2	Chr14: 19.755208	0.207
143 ☐	MA_M2F_0706_R	1440742_at	*LOC239447*	ESTs	Chr18: 6.491461	6.615	65	14.6	Chr14: 19.755208	0.103

144 ☐	MA_M2F_0706_R	1433750_at	*Slc31a1*	solute carrier family 31, member 1; distal 3' UTR	Chr4: 62.391176	10.141	65	13.9	Chr11: 22.916002	-0.166
145 ☐	MA_M2F_0706_R	1422302_s_at	*Ftl1*	ferritin light polypeptide 1; exons 2 and 4 (of 4)	Chr7: 45.458120	15.955	65	18.0	Chr14: 55.622689	-0.150
146 ☐	MA_M2F_0706_R	1452972_at	*1700013G20Rik*	RIKEN cDNA 1700013G20 gene	Chr12: 9.034923	9.434	65	10.8	Chr17: 10.720847	-0.128
147 ☐	MA_M2F_0706_R	1452093_at	*Tmem185b*	transmembrane protein 185B; 3' UTR	Chr1: 119.527628	9.697	65	15.1	Chr14: 3.000000	0.125
148 ☐	MA_M2F_0706_R	1448979_at	*Muted*	muted	Chr13: 38.602741	9.480	65	15.7	Chr14: 9.528965	0.142
149 ☐	MA_M2F_0706_R	1434563_at	*Rps6kc1*	ribosomal protein S6 kinase polypeptide 1; mid-proximal 3' UTR or intron	Chr1: 190.773292	7.684	65	13.5	Chr14: 19.755208	-0.106
150 ☐	MA_M2F_0706_R	1448712_at	*Chm*	choroidermia; mid 3'UTR	ChrX: 113.041468	8.544	65	14.2	Chr14: 9.528965	0.175
151 ☐	MA_M2F_0706_R	1456038_at	*Fbxl4*	F-box and leucine-rich repeat protein 4; 2 exons and proximal 3'UTR	Chr4: 22.427368	8.935	65	11.1	Chr14: 9.528965	0.160
152 ☐	MA_M2F_0706_R	1448540_a_at	*0610012G03Rik*	RIKEN cDNA 0610012G03 gene	Chr16: 31.947861	10.690	65	12.3	ChrX: 70.094108	-0.124
153 ☐	MA_M2F_0706_R	1428652_at	*0610010F05Rik*	RIKEN cDNA 0610010F05; distal 3' UTR	Chr11: 23.573924	8.242	65	14.3	Chr14: 9.528965	0.189
154 ☐	MA_M2F_0706_R	1434750_at	*Exoc3*	exocyst complex component 3; last four exons and proximal 3' UTR	Chr13: 74.172009	9.699	65	11.4	Chr8: 94.374289	-0.115
155 ☐	MA_M2F_0706_R	1429351_at	*Klhl24*	kelch-like 24; mid 3' UTR	Chr16: 20.124883	10.148	65	9.3	Chr13: 115.551768	-0.190

156 ☐	MA_M2F_0706_R	1435529_at	*Ifit1*	interferon-induced protein with tetratricopeptide repeats 1 related sequence, OTTMUSG00000016644; 3' UTR of Ifit1-related sequence (from 2010002M12Rik)	Chr19: 34.593014	7.121	65	16.6	Chr14: 19.755208	0.150
157 ☐	MA_M2F_0706_R	1418579_at	*Cetn2*	centrin 2	ChrX: 72.913915	9.910	65	17.8	ChrX: 71.441039	-0.236
158 ☐	MA_M2F_0706_R	1434099_at	*Ppargc1a*	peroxisome proliferative activated receptor, gamma, coactivator 1 alpha; far 3' UTR	Chr5: 51.454320	9.157	65	11.1	Chr1: 191.601182	-0.140
159 ☐	MA_M2F_0706_R	1429108_at	*Msl2l1*	male-specific lethal 2-like 1; 3' UTR	Chr13: 66.774460	7.431	65	15.8	Chr14: 30.957748	0.145
160 ☐	MA_M2F_0706_R	1435893_at	*Vldlr*	very low density lipoprotein receptor; putative far 3' UTR	Chr19: 27.253653	9.829	65	15.9	Chr11: 83.064807	0.183
161 ☐	MA_M2F_0706_R	1456744_x_at	*Flcn*	folliculin; distal 3'UTR	Chr11: 59.791771	5.895	65	12.4	Chr11: 83.064807	0.059
162 ☐	MA_M2F_0706_R	1434561_at	*Asxl1*	additional sex combs like 1; distal 3' UTR	Chr2: 153.403453	9.136	65	11.0	Chr14: 24.000000	0.133
163 ☐	MA_M2F_0706_R	1417438_at	*Rdh14*	retinol dehydrogenase 14 (all-trans and 9-cis)	Chr12: 10.395066	10.554	65	9.9	ChrX: 64.161107	-0.119
164 ☐	MA_M2F_0706_R	1434719_at	*A2m*	alpha-2-macroglobulin; last 7 exons except last exon	Chr6: 121.674940	6.859	65	7.9	Chr14: 19.755208	-0.077
165 ☐	MA_M2F_0706_R	1460182_at	*Snx4*	sorting nexin 4; mid to distal 3'UTR	Chr16: 33.298991	9.587	65	16.0	Chr14: 9.528965	0.151
166 ☐	MA_M2F_0706_R	1417077_at	*Bcap29*	B-cell receptor-associated protein 29; mid 3' UTR	Chr12: 31.595531	8.748	65	9.4	Chr10: 55.515150	-0.222

167	MA_M2F_0706_R	1418066_at	*Cfl2*	cofilin 2, muscle; distal 3' UTR	Chr12: 54.858855	9.192	65	17.8	ChrX: 47.876769	-0.199
168	MA_M2F_0706_R	1450987_a_at	*2310004I24Rik*	RIKEN cDNA 2310004I24 gene	Chr11: 67.038167	9.113	65	12.8	Chr14: 27.000000	0.119
169	MA_M2F_0706_R	1417704_a_at	*Arhgap6*	Rho GTPase activating protein 6	ChrX: 169.303893	6.311	65	11.3	Chr8: 94.374289	-0.070
170	MA_M2F_0706_R	1450950_at	*Cspg6*	structural maintenance of chromosomes 3 (CNS age-associated); exons 26 and 27 and proximal 3' UTR (transQTL on Chr 12 in BXD hippocampus data)	Chr19: 53.641939	10.706	65	9.9	Chr11: 18.283704	-0.096
171	MA_M2F_0706_R	1459712_at	*Zfp182*	zinc finger protein 182	ChrX: 21.026222	5.703	65	11.3	Chr14: 3.000000	0.048
172	MA_M2F_0706_R	1418651_at	*Spata6*	spermatogenesis associated 6	Chr4: 111.828580	8.411	65	10.5	Chr16: 3.500000	-0.097
173	MA_M2F_0706_R	1446471_at	*B130066H01Rik*	15 days embryo head cDNA, RIKEN full-length enriched library, clone:D930045O10 product:unknown EST, full insert sequence.	Chr7: 129.649450	7.061	65	23.2	Chr14: 9.528965	-0.068
174	MA_M2F_0706_R	1417319_at	*Pvrl3*	poliovirus receptor-related 3	Chr16: 46.447261	8.254	65	14.6	Chr14: 24.000000	0.192
175	MA_M2F_0706_R	1449341_a_at	*Stom*	stomatin	Chr2: 35.315873	9.342	65	8.7	Chr11: 24.905498	0.111
176	MA_M2F_0706_R	1419462_s_at	*Gtl3*	gene trap locus 3	Chr8: 95.420551	8.746	65	14.9	Chr8: 94.374289	-0.212
177	MA_M2F_0706_R	1421888_x_at	*Aplp2*	amyloid beta (A4) precursor-like protein 2; distal 3' UTR	Chr9: 31.149603	10.659	65	10.2	Chr3: 39.650830	0.074

178 ☐	MA_M2F_0706_R	1427114_at	*Ttc19*	tetratricopeptide repeat domain 19 (mitochondrial)	Chr11: 62.314658	7.098	65	15.2	Chr14: 9.528965	0.155
179 ☐	MA_M2F_0706_R	1418640_at	*Sirt1*	sirtuin 1, NAD+ dependent histone deacetylase, class 1 (longevity, caloric restriction associated, mitochondrial biogenesis, cell survival); distal 3' UTR	Chr10: 63.319162	8.837	65	12.8	Chr14: 3.000000	0.227
180 ☐	MA_M2F_0706_R	1418048_at	*1110059G10Rik*	RIKEN cDNA 1110059G10 gene	Chr9: 122.947701	10.525	65	16.5	Chr11: 83.064807	0.152
181 ☐	MA_M2F_0706_R	1428415_at	*2310020H19Rik*	RIKEN cDNA 2310020H19 gene	Chr12: 84.418169	7.440	65	15.2	Chr12: 51.647704	0.121
182 ☐	MA_M2F_0706_R	1425979_a_at	*Fbf1*	Fas (TNFRSF6) binding factor 1	Chr11: 116.142298	10.206	65	15.2	Chr14: 115.744394	-0.122
183 ☐	MA_M2F_0706_R	1454947_a_at	*Ub1cp1*	ubiquitin-like domain containing CTD phosphatase 1; distal 3' UTR	Chr11: 44.454597	10.071	65	10.3	Chr1: 95.094462	0.141
184 ☐	MA_M2F_0706_R	1455633_at	*Zfp647*	zinc finger protein 647; last exon and 3' UTR	Chr15: 76.910433	6.571	65	12.6	Chr14: 24.000000	0.122
185 ☐	MA_M2F_0706_R	1449960_at	*Nptx2*	neuronal pentraxin 2; last exon and 3' UTR	Chr5: 144.556182	6.458	65	8.8	Chr14: 19.755208	0.057
186 ☐	MA_M2F_0706_R	1450418_a_at	*Yipf4*	Yip1 domain family, member 4; 3' UTR	Chr17: 74.499383	9.199	65	10.1	Chr7: 84.149847	0.140
187 ☐	MA_M2F_0706_R	1435116_at	*Kiaa1383*	KIAA1383, RIKEN cDNA 4933403G14; 3' UTR	Chr8: 125.672794	6.191	65	12.1	Chr14: 9.528965	0.088
188 ☐	MA_M2F_0706_R	1441200_at	*Klf3*	Kruppel-like factor 3 (basic)	Chr5: 64.824218	10.853	65	11.6	Chr14: 55.622689	-0.098
189 ☐	MA_M2F_0706_R	1437511_x_at	*Mclc*	myeloid cell leukemia sequence 1, related sequence 1	Chr3: 108.678619	9.610	65	14.1	Chr14: 24.000000	0.164

190	MA_M2F_0706_R	1420139_s_at	*Krr1*	KRR1, small subunit (SSU) processome component, homolog; mid 3' UTR	Chr10: 111.985871	9.281	65	11.7	Chr14: 24.000000	0.190
191	MA_M2F_0706_R	1441342_at	*Dpp4*	dipeptidylpeptidase 4	Chr2: 62.330153	7.859	65	13.0	Chr14: 9.528965	0.220
192	MA_M2F_0706_R	1434960_at	*Taf9b*	TAF9B RNA polymerase II, TATA box binding protein (TBP)-associated factor; distal 3' UTR	ChrX: 106.207073	8.542	65	12.1	Chr14: 3.000000	0.173
193	MA_M2F_0706_R	1428775_at	*Kiaa0391*	mitochondrial ribonuclease P protein 3	Chr12: 55.377219	8.427	65	15.8	Chr12: 46.816738	0.109
194	MA_M2F_0706_R	1448917_at	*Thrap6*	thyroid hormone receptor associated protein 6; 3 exons and proximal 3'UTR	Chr15: 52.719396	10.231	65	10.0	Chr8: 80.868085	-0.097
195	MA_M2F_0706_R	1416794_at	*Atl2*	atlastin GTPase 2 (ADP-ribosylation factor-like 6 interacting protein 2); exon 9	Chr17: 79.852664	8.921	65	8.8	Chr15: 72.500609	-0.153
196	MA_M2F_0706_R	1436706_at	*Tmem32*	transmembrane protein 32	ChrX: 56.586781	8.848	65	13.5	Chr14: 3.000000	0.199
197	MA_M2F_0706_R	1419276_at	*Enpp1*	ectonucleotide pyrophosphatase/phosphodiesterase 1	Chr10: 24.645301	9.196	65	10.5	Chr2: 134.800968	0.170
198	MA_M2F_0706_R	1431252_a_at	*Zfp655*	zinc finger protein 655	Chr5: 145.244666	7.540	65	15.0	ChrX: 61.058643	-0.188
199	MA_M2F_0706_R	1420959_at	*Asph*	aspartate-beta-hydroxylase; mid-distal 3' UTR	Chr4: 9.451848	8.133	65	12.9	Chr14: 19.755208	0.215
200	MA_M2F_0706_R	1416115_at	*Orc3l*	origin recognition complex, subunit 3-like (S. cerevisiae)	Chr4: 34.572464	9.689	65	12.2	Chr2: 134.462008	0.117
201	MA_M2F_0706_R	1421908_a_at	*Tcf12*	transcription factor 12; distal 3' UTR	Chr9: 71.845794	9.714	65	14.8	Chr15: 69.108222	-0.166

202 ☐	MA_M2F_0706_R	1417442_a_at	*Pex3*	peroxisomal biogenesis factor 3	Chr10: 13.532375	9.350	65	11.7	Chr13: 25.000000	0.144
203 ☐	MA_M2F_0706_R	1420850_at	*Crnkl1*	Crn, crooked neck-like 1 (Drosophila)	Chr2: 145.920593	7.867	65	11.9	Chr15: 68.818097	-0.109
204 ☐	MA_M2F_0706_R	1427831_s_at	*Zfp260*	zinc finger protein 260	Chr7: 30.105601	7.368	65	13.7	Chr14: 3.000000	0.220
205 ☐	MA_M2F_0706_R	1418650_at	*Spata6*	spermatogenesis associated 6	Chr4: 111.799138	8.253	65	11.8	Chr10: 64.710346	-0.088
206 ☐	MA_M2F_0706_R	1430623_s_at	*5830411E10Rik*	RIKEN cDNA 5830411E10 gene	Chr1: 51.470742	9.677	65	11.6	Chr10: 67.616312	-0.160
207 ☐	MA_M2F_0706_R	1420977_at	*Man1a2*	mannosidase, alpha, class 1A, member 2	Chr3: 100.565465	8.629	65	20.6	Chr14: 9.528965	0.208
208 ☐	MA_M2F_0706_R	1450762_s_at	*Zfp191*	zinc finger protein 191; proximal 3' UTR	Chr18: 24.013534	7.585	65	8.4	Chr15: 63.392263	-0.073
209 ☐	MA_M2F_0706_R	1421139_a_at	*Zfp386*	zinc finger protein 386 (Kruppel-like); last exon	Chr12: 116.059259	7.447	65	11.5	Chr14: 3.000000	0.198
210 ☐	MA_M2F_0706_R	1425022_at	*Usp3*	ubiquitin specific protease 3; last 4 exons and proximal 3' UTR	Chr9: 66.518371	10.137	65	8.9	Chr10: 48.377966	-0.104
211 ☐	MA_M2F_0706_R	1415789_a_at	*LOC665689*	similar to ubiquitin-like domain containing CTD phosphatase 1; putative exon (from ESTs)	Chr11: 16.635159	9.069	65	13.2	Chr11: 42.818374	0.286
212 ☐	MA_M2F_0706_R	1420999_at	*Cnot4*	CCR4-NOT transcription complex, subunit 4	Chr6: 35.046068	8.044	65	9.0	Chr2: 134.800968	0.132
213 ☐	MA_M2F_0706_R	1429250_at	*Dnchc2*	dynein, cytoplasmic, heavy chain 2; last 4 exons (transQTL on Chr 4 in BXD eye data)	Chr9: 6.929469	9.350	65	10.0	Chr2: 138.500000	0.110

214	MA_M2F_0706_R	1451619_at	*Golph3l*	golgi phosphoprotein 3-like; last exon and proximal 3' UTR	Chr3: 95.617448	8.351	65	10.4	ChrX: 84.597186	-0.174
215	MA_M2F_0706_R	1431784_a_at	*2310066N05Rik*	RIKEN cDNA 2310066I18 gene	Chr3: 146.512172	8.362	65	12.7	ChrX: 64.161107	-0.138
216	MA_M2F_0706_R	1421045_at	*Mrc2*	mannose receptor, C type 2	Chr11: 105.350580	8.407	65	12.8	Chr9: 99.750326	-0.171
217	MA_M2F_0706_R	1421448_at	*Garnl1*	GTPase activating RANGAP domain-like 1; exons 32, 33, 34, and 35	Chr12: 55.640606	8.533	65	14.9	ChrX: 73.428893	-0.235
218	MA_M2F_0706_R	1419089_at	*Timp3*	tissue inhibitor of metalloproteinase 3	Chr10: 86.347860	14.442	65	15.7	Chr14: 19.755208	-0.149
219	MA_M2F_0706_R	1451337_at	*Psmf1*	proteasome (prosome, macropain) inhibitor subunit 1; last 2 exons and proximal 3' UTR	Chr2: 151.718622	9.984	65	12.3	Chr10: 67.616312	-0.123
220	MA_M2F_0706_R	1429186_a_at	*Cdadc1*	cytidine and dCMP deaminase domain containing 1	Chr14: 59.586352	8.359	65	9.5	Chr2: 134.800968	0.152
221	MA_M2F_0706_R	1426456_a_at	*Miz1*	Msx-interacting-zinc finger; exons 8, 9, 10, and 11	Chr18: 77.133220	9.302	65	15.7	ChrX: 73.876578	-0.152
222	MA_M2F_0706_R	1451317_at	*Ythdf2*	YTH domain family 2; last two exons and proximal 3' UTR	Chr4: 132.186852	9.743	65	11.8	Chr15: 69.108222	-0.183
223	MA_M2F_0706_R	1460399_at	*BC018601*	cDNA sequence BC018601	Chr11: 5.529291	9.077	65	11.2	Chr12: 29.713200	0.106
224	MA_M2F_0706_R	1456867_x_at	*Ergic3*	ERGIC and golgi 3; last exon	Chr2: 156.018075	11.707	65	10.4	Chr7: 142.462521	-0.087
225	MA_M2F_0706_R	1451730_at	*Zfp62*	zinc finger protein 62	Chr11: 49.217237	7.295	65	11.0	Chr14: 3.000000	0.126

226 ☐	MA_M2F_0706_R	1434979_at	*4933403F05Rik*	RIKEN cDNA 4933403F05 gene	Chr18: 68.267767	8.473	65	16.6	ChrX: 73.876578	-0.203
227 ☐	MA_M2F_0706_R	1419803_s_at	*Ccdc12*	coiled-coil domain containing 12; last three exons and proximal 3' UTR	Chr9: 110.711126	11.139	65	12.8	Chr1: 5.641533	0.080
228 ☐	MA_M2F_0706_R	1418968_at	*Rb1cc1*	RB1-inducible coiled-coil 1; mid 3'UTR	Chr1: 6.274578	10.245	65	10.1	Chr10: 53.743148	-0.097
229 ☐	MA_M2F_0706_R	1428845_at	*Bclaf1*	BCL2-associated transcription factor 1; distal 3' UTR (transQTL on Chr 4 in BXD eye data)	Chr10: 20.341571	10.077	65	9.7	Chr16: 3.500000	-0.152
230 ☐	MA_M2F_0706_R	1448127_at	*Rrm1*	ribonucleotide reductase M1; distal 3' UTR	Chr7: 102.459425	8.437	65	11.4	Chr2: 136.127470	0.158
231 ☐	MA_M2F_0706_R	1453282_at	*Cxadr*	coxsackievirus and adenovirus receptor	Chr16: 78.339777	8.041	65	13.1	Chr14: 19.755208	0.144
232 ☐	MA_M2F_0706_R	1426842_at	*Ythdf3*	YTH domain family 3; proximal and mid 3' UTR	Chr3: 16.214439	10.305	65	10.2	Chr14: 3.000000	0.176
233 ☐	MA_M2F_0706_R	1423961_at	*Wdr26*	WD repeat domain 26 (putative Mtv7, Mls1); putative far 3' UTR	Chr1: 181.176207	9.623	65	13.3	Chr15: 67.990415	-0.160
234 ☐	MA_M2F_0706_R	1427658_at	*Ctbs*	chitobiase, di-N-acetyl-	Chr3: 146.459677	8.061	65	11.6	Chr2: 134.800968	0.187
235 ☐	MA_M2F_0706_R	1426216_at	*Cog6*	component of oligomeric golgi complex 6	Chr3: 52.982493	10.023	65	13.6	ChrX: 92.675500	-0.134
236 ☐	MA_M2F_0706_R	1449666_at	*Atrnl1*	attractin like 1; antisense of distal 3' UTR	Chr19: 58.132875	8.404	65	12.5	Chr10: 73.927005	0.049
237 ☐	MA_M2F_0706_R	1434332_at	*Zzz3*	zinc finger, ZZ domain containing 3; mid to distal 3' UTR (transQTL on chr 4 in BXD Eye Data)	Chr3: 152.458886	10.318	65	13.1	Chr2: 181.014276	0.115

238 ☐	MA_M2F_0706_R	1424280_at	*Mospd1*	motile sperm domain containing 1; mid to distal 3' UTR	ChrX: 53.345058	9.189	65	9.3	Chr11: 4.784489	-0.132
239 ☐	MA_M2F_0706_R	1428335_a_at	*Scfd1*	sec1 family domain containing 1; last six exons of short form message	Chr12: 51.431515	10.265	65	8.8	ChrX: 64.161107	-0.147
240 ☐	MA_M2F_0706_R	1418222_at	*Noa1*	nitric oxide associated 1; 4 exons	Chr5: 77.294479	8.948	65	11.1	Chr7: 66.458029	0.136
241 ☐	MA_M2F_0706_R	1451135_at	*Gtf2b*	general transcription factor IIB; 2 exons and 3'UTR	Chr3: 142.781484	10.855	65	12.4	Chr16: 11.961405	-0.076
242 ☐	MA_M2F_0706_R	1455439_a_at	*Lgals1*	lectin, galactose binding, soluble 1; last exon and proximal 3' UTR	Chr15: 78.930026	13.086	65	10.7	Chr6: 49.309149	0.171
243 ☐	MA_M2F_0706_R	1456255_at	*Kiaa0368*	proteasome-associated protein ECM29 homolog	Chr4: 58.844163	6.439	65	8.7	Chr15: 63.392263	-0.085
244 ☐	MA_M2F_0706_R	1451124_at	*Sod1*	superoxide dismutase 1, soluble; first four exons and 3' UTR	Chr16: 90.222782	13.768	65	8.3	Chr2: 17.898115	0.198
245 ☐	MA_M2F_0706_R	1428307_at	*Zdhhc13*	zinc finger, DHHC domain containing 13; 2 exons and 3'UTR	Chr7: 48.826869	9.834	65	11.9	Chr10: 67.616312	-0.100
246 ☐	MA_M2F_0706_R	1418974_at	*Blzf1*	basic leucine zipper nuclear factor 1	Chr1: 164.292202	8.466	65	9.2	Chr12: 51.647704	0.097
247 ☐	MA_M2F_0706_R	1437295_at	*Pkn2*	protein kinase N2	Chr3: 142.792158	9.473	65	13.0	Chr15: 69.108222	-0.195
248 ☐	MA_M2F_0706_R	1450348_at	*Slc19a3*	solute carrier family 19 (sodium/hydrogen exchanger), member 3	Chr1: 83.014703	7.710	65	10.4	Chr3: 25.041091	0.159
249 ☐	MA_M2F_0706_R	1454929_s_at	*AU018122*	hypothetical protein E130307D12	Chr17: 56.606034	10.913	65	13.9	Chr2: 138.500000	-0.140

250	MA_M2F_0706_R	1431745_a_at	*Zc3h14*	zinc finger CCCH type containing 14; exons 13, 14, 15, and 16 (transQTL on Chr 4 in BXD eye data)	Chr12: 98.783584	11.764	65	9.5	Chr14: 81.380779	-0.074
251	MA_M2F_0706_R	1427466_at	*Pigu*	phosphatidylinositol glycan anchor biosynthesis, class U (Pigu)	Chr2: 155.278639	7.436	65	14.9	Chr15: 28.321742	0.109
252	MA_M2F_0706_R	1450745_at	*C1galt1*	core 1 synthase, glycoprotein-N-acetylgalactosamine 3-beta-galactosyltransferase	Chr6: 7.871503	9.260	65	9.8	ChrX: 84.597186	-0.168
253	MA_M2F_0706_R	1426824_at	*Psme4*	proteasome (prosome, macropain) activator subunit 4	Chr11: 30.856055	10.899	65	16.3	ChrX: 73.028443	-0.227
254	MA_M2F_0706_R	1423612_at	*Clp1*	ATP/GTP-binding protein; last exon and proximal 3' UTR	Chr2: 84.723372	8.531	65	14.8	Chr2: 134.800968	0.158
255	MA_M2F_0706_R	1444141_at	*Snx13*	sorting nexin 13; exons 13 through 17	Chr12: 35.106769	6.774	65	10.6	Chr3: 128.292533	0.091
256	MA_M2F_0706_R	1453263_at	*Mak10*	MAK10 homolog, amino-acid N-acetyltransferase subunit, (S. cerevisiae)	Chr13: 59.600932	7.823	65	9.0	Chr2: 134.800968	0.116
257	MA_M2F_0706_R	1453533_at	*4933403O08Rik*	RIKEN cDNA 4933403O08 gene	ChrX: 112.243333	6.049	65	10.3	Chr10: 121.639039	0.043
258	MA_M2F_0706_R	1427321_s_at	*Cxadr*	coxsackievirus and adenovirus receptor; distal 3' UTR	Chr16: 78.338806	8.548	65	12.8	Chr10: 67.616312	-0.155
259	MA_M2F_0706_R	1451770_s_at	*Dhx9*	DEAH (Asp-Glu-Ala-His) box polypeptide 9	Chr1: 153.455871	10.770	65	10.6	Chr18: 65.174965	-0.126
260	MA_M2F_0706_R	1425199_a_at	*Epb4.1l5*	erythrocyte protein band 4.1-like 5; last exon and 3'UTR	Chr1: 119.595376	10.901	65	11.8	Chr2: 181.014276	0.125

261 ☐	MA_M2F_0706_R	1435646_at	*Ikbkg*	inhibitor of kappaB kinase gamma	ChrX: 74.449985	7.849	65	13.6	ChrX: 73.876578	-0.181
262 ☐	MA_M2F_0706_R	1419049_at	*Pcnx*	pecanex homolog; mid 3' UTR	Chr12: 81.998196	6.302	65	9.5	Chr10: 73.927005	-0.056
263 ☐	MA_M2F_0706_R	1424397_at	*Dhx36*	DEAH (Asp-Glu-Ala-His) box polypeptide 36	Chr3: 62.470459	7.069	65	12.8	Chr14: 3.000000	0.133
264 ☐	MA_M2F_0706_R	1416132_at	*Efr3a*	EFR3 homolog A (hearing loss associated, activity-dependent plasticity-associated multi-pass membrane KIAA0143 protein); five exons	Chr15: 65.854698	7.781	65	11.3	Chr10: 67.616312	-0.151
265 ☐	MA_M2F_0706_R	1422772_at	*C1galt1*	core 1 synthase, glycoprotein-N-acetylgalactosamine 3-beta-galactosyltransferase; putative far 3' UTR	Chr6: 7.872849	8.974	65	13.5	ChrX: 64.161107	-0.256
266 ☐	MA_M2F_0706_R	1457949_at	*Nt5c2*	5'-nucleotidase, cytosolic II; putative exon	Chr19: 46.885497	7.808	65	9.6	Chr10: 53.743148	-0.089
267 ☐	MA_M2F_0706_R	1426411_a_at	*Strbp*	spermatid perinuclear RNA binding protein (interleukin enhancer binding factor 3-like)	Chr2: 37.586519	7.887	65	11.6	ChrX: 73.428893	-0.191
268 ☐	MA_M2F_0706_R	1434918_at	*Sox6*	SRY-box containing gene 6	Chr7: 115.471185	9.714	65	11.0	Chr11: 83.064807	0.113
269 ☐	MA_M2F_0706_R	1438935_at	*BB234005*	expressed sequence BB234005	Chr10: 128.362483	6.408	65	10.4	Chr14: 9.528965	0.040
270 ☐	MA_M2F_0706_R	1448309_at	*Ap3m1*	adaptor-related protein complex 3, mu 1 subunit; last four exons and proximal 3' UTR	Chr14: 21.036673	8.785	65	12.4	ChrX: 61.058643	-0.179
271 ☐	MA_M2F_0706_R	1418369_at	*Prim1*	DNA primase, p49 subunit	Chr10: 128.023845	8.677	65	10.9	Chr11: 83.064807	0.171

272 ☐	MA_M2F_0706_R	1438264_a_at	*Tpp2*	tripeptidyl peptidase II (serine exopeptidase of the 26S proteasome); three exons	Chr1: 43.980362	8.214	65	8.6	Chr14: 3.000000	0.191
273 ☐	MA_M2F_0706_R	1428087_at	*Dnm1l*	dynamin 1-like (mitochondrial fission); proximal 3' UTR	Chr16: 16.313539	10.478	65	9.6	Chr10: 68.340621	-0.097
274 ☐	MA_M2F_0706_R	1460207_s_at	*E2f5*	E2F transcription factor 5	Chr3: 14.605796	10.160	65	12.8	Chr2: 181.014276	0.155
275 ☐	MA_M2F_0706_R	1438365_x_at	*Laptm4b*	lysosomal-associated protein transmembrane 4B; distal 3' UTR	Chr15: 34.284138	13.172	65	11.7	Chr2: 136.127470	-0.134
276 ☐	MA_M2F_0706_R	1450899_at	*Nedd1*	neural precursor cell expressed, developmentally down-regulated gene 1	Chr10: 92.685169	9.371	65	13.7	Chr14: 3.000000	0.157
277 ☐	MA_M2F_0706_R	1455541_a_at	*4430402I18Rik*	RIKEN cDNA 4430402I18 gene	Chr19: 28.927648	6.988	65	23.1	Chr2: 134.519309	0.132
278 ☐	MA_M2F_0706_R	1416584_at	*Man2b2*	mannosidase 2, alpha B2; second, third, and fourth exon from last, and mid 3' UTR	Chr5: 36.806997	9.356	65	9.5	Chr13: 115.551768	0.132
279 ☐	MA_M2F_0706_R	1425023_at	*Usp3*	ubiquitin specific protease 3	Chr9: 66.517750	7.999	65	11.0	Chr4: 102.851020	0.128
280 ☐	MA_M2F_0706_R	1443962_at	*Tfdp2*	transcription factor Dp 2	Chr9: 96.310545	7.909	65	9.4	Chr2: 136.127470	0.120
281 ☐	MA_M2F_0706_R	1425491_at	*Bmpr1a*	bone morphogenetic protein receptor, type 1A	Chr14: 34.414005	8.247	65	18.8	ChrX: 73.428893	-0.170
282 ☐	MA_M2F_0706_R	1452496_at	*Atp11c*	Atpase, class VI, type 11C	ChrX: 60.236631	6.884	65	9.6	Chr11: 103.979646	-0.102
283 ☐	MA_M2F_0706_R	1448348_at	*Gpiap1*	cell cycle associated protein 1 (cytoplasmic activation- and proliferation-	Chr2: 103.773010	9.668	65	12.2	Chr15: 89.576205	-0.207

				associated protein 1); exons 9 through 12						
284	MA_M2F_0706_R	1452780_at	*Gtf3c2*	general transcription factor IIIC, polypeptide 2, beta; last exon and 3' UTR (complex 3' UTR structure)	Chr5: 31.157418	9.868	65	8.5	ChrX: 84.597186	-0.097
285	MA_M2F_0706_R	1431811_a_at	*Fbxo34*	F-box only protein 34; 3' UTR from proximal to distal	Chr14: 47.531295	9.179	65	7.3	Chr14: 19.755208	0.102

Supplemental Table S9. Top 100 genes in male for 3 probes in Ace2

	Dataset	Trait ID	Symbol	Description	Location	Mean	N Cases	Max LRS	Max LRS Location Chr and Mb	Add
1	MA_M2M_0706_R	1425103_at	*Ace2*	angiotensin I converting enzyme (peptidyl-dipeptidase A) 2; middle to distal 3'-UTR	ChrX: 164.187853	10.201	45	12.0	Chr1: 14.576853	0.169
2	MA_M2M_0706_R	1425102_a_at	*Ace2*	angiotensin I converting enzyme (peptidyl-dipeptidase A) 2; 3'-UTR and last 4 exons	ChrX: 164.182645	10.127	45	10.2	Chr9: 107.639250	0.185
3	MA_M2M_0706_R	1438719_at	*AI585793*	expressed sequence AI585793	Chr18: 32.232049	7.583	45	13.0	Chr18: 31.556239	-0.209
4	MA_M2M_0706_R	1452138_a_at	*Ace2*	angiotensin I converting enzyme (peptidyl-dipeptidase A) 2; last 2~6 and 11 exons	ChrX: 164.167855	10.125	45	13.1	Chr8: 89.094303	0.214

5	MA_M2M_0706_R	1459392_at	*Phf8*	PHD finger protein 8	ChrX: 151.625300	7.100	45	11.4	Chr6: 125.820102	0.082
6	MA_M2M_0706_R	1439661_at	*Slc16a14*	RIKEN cDNA 1110004H10 gene	Chr1: 84.905979	8.885	45	16.1	Chr3: 65.524002	-0.349
7	MA_M2M_0706_R	1436874_x_at	*Slc25a5*	solute carrier family 25 (mitochondrial carrier, adenine nucleotide translocator), member 5; last two exons and 3' UTR	ChrX: 36.797832	16.402	45	8.3	ChrX: 102.414526	0.081
8	MA_M2M_0706_R	1452609_at	*1190005I06Rik*	RIKEN cDNA 1190005I06 gene	Chr8: 120.608622	6.404	45	12.1	Chr3: 69.025720	0.067
9	MA_M2M_0706_R	1460380_at	*Dsg2*	desmoglein 2 (calcium-binding transmembrane glycoprotein, arrhythmogenic right ventricular dysplasia, familial 10)	Chr18: 20.602254	7.394	45	8.0	Chr15: 102.320887	0.069
10	MA_M2M_0706_R	1423044_at	*Prosc*	proline synthetase co-transcribed	Chr8: 27.054003	12.981	45	13.2	Chr10: 110.416695	-0.125
11	MA_M2M_0706_R	1430561_at	*Dnajb14*	DnaJ (Hsp40) homolog, subfamily B, member 14; proximal and mid 3' UTR	Chr3: 137.908489	8.283	45	11.7	Chr2: 78.100320	0.259
12	MA_M2M_0706_R	1438736_at	*Thoc2*	THO complex 2; four exons	ChrX: 41.822401	8.389	45	8.7	Chr3: 53.053282	-0.152
13	MA_M2M_0706_R	1458499_at	*Pde10a*	phosphodiesterase 10A; half distal 3' UTR	Chr17: 8.985059	6.988	45	12.8	Chr2: 169.897347	0.060
14	MA_M2M_0706_R	1452270_s_at	*Cubn*	cubilin (intrinsic factor-cobalamin receptor)	Chr2: 13.276377	14.228	45	17.2	ChrX: 102.126656	0.186
15	MA_M2M_0706_R	1430542_a_at	*Slc25a5*	solute carrier family 25 (mitochondrial carrier, adenine nucleotide translocator), member 5	ChrX: 36.798388	16.091	45	10.0	Chr7: 71.696966	0.089
16	MA_M2M_0706_R	1440486_at	*C12orf41*	human chromosome 12 open reading frame 41; far 3' UTR	Chr15: 98.517815	7.542	45	8.7	Chr13: 3.150000	0.081
17	MA_M2M_0706_R	1436747_at	*LOC280487*	pol polyprotein	Chr11: 116.438594	12.033	45	13.7	Chr13: 103.949936	-0.144
18	MA_M2M_0706_R	1459699_at	*Tem7*	ESTs	Chr4: 136.203427	6.241	45	10.2	Chr10: 110.416695	0.073

19	MA_M2M_0706_R	1451837_at	*Ap3b2*	adaptor-related protein complex 3, beta 2 subunit	Chr7: 81.474401	6.410	45	9.6	Chr6: 32.610603	0.072
20	MA_M2M_0706_R	1420500_at	*Dnajc1*	DnaJ (Hsp40) homolog, subfamily C, member 1; last 2 exons and 3' UTR	Chr2: 18.217204	8.557	45	12.0	Chr13: 103.949936	0.169
21	MA_M2M_0706_R	1448803_at	*Golga4*	golgi autoantigen, golgin subfamily a, 4	Chr9: 118.556220	8.990	45	10.5	Chr7: 6.024860	0.285
22	MA_M2M_0706_R	1422495_a_at	*Hmgn1*	high mobility group nucleosomal binding domain 1; mid 3' UTR	Chr16: 18.938220	13.533	45	9.3	Chr10: 87.643399	-0.098
23	MA_M2M_0706_R	1432448_at	*2600006K01Rik*	RIKEN cDNA 2600006K01 gene	Chr2: 29.868759	6.842	45	8.0	Chr12: 115.550550	0.069
24	MA_M2M_0706_R	1447571_at	*Elk4*	ELK4, member of ETS oncogene family	Chr1: 132.026246	10.159	45	8.4	Chr2: 70.613187	-0.089
25	MA_M2M_0706_R	1456979_at	*Zhx3*	zinc fingers and homeoboxes 3	Chr2: 160.856133	8.270	45	6.8	Chr9: 68.339182	0.120
26	MA_M2M_0706_R	1421613_at	*H2afy2*	H2A histone family, member Y2	Chr15: 62.217666	5.902	45	9.6	ChrX: 115.338127	0.050
27	MA_M2M_0706_R	1443053_at	*Ptprd*	protein tyrosine phosphatase, receptor type, D; intron 1 (from EST AK053007)	Chr4: 76.435514	7.633	45	33.9	Chr4: 75.840751	-0.372
28	MA_M2M_0706_R	1417623_at	*Slc12a2*	solute carrier family 12, member 2	Chr18: 57.930155	7.756	45	14.1	Chr11: 33.490349	0.261
29	MA_M2M_0706_R	1445669_at	*Spry4*	sprouty homolog 4 (Drosophila)	Chr18: 38.586291	6.072	45	6.5	Chr9: 29.939029	-0.054
30	MA_M2M_0706_R	1437082_at	*Akap9*	A kinase (PRKA) anchor protein (yotiao) 9; exons 3, 4, and 5 and distal end of intron 3	Chr5: 3.954395	6.823	45	13.1	Chr2: 178.717318	0.120
31	MA_M2M_0706_R	1457639_at	*Atp6v1h*	ATPase, H+ transporting, lysosomal, V1 subunit H; intronic or alternative splicing (AK081492)	Chr1: 5.099623	6.534	45	9.4	Chr2: 69.342849	0.116
32	MA_M2M_0706_R	1436983_at	*Crebbp*	CREB binding protein; two exons toward 3' end of gene	Chr16: 4.093487	8.419	45	11.1	Chr2: 78.100320	0.285
33	MA_M2M_0706_R	1447014_at	*AK144361*	AK144361 EST expressed in CNS; 3' end, possible association with Ccdc115	Chr1: 58.510627	7.288	45	9.7	Chr3: 54.220588	0.075

34 ☐	MA_M2M_0706_R	1456717_at	*Tead1*	TEA domain family member 1 (SV40 transcriptional enhancer factor, Sveinsson's chorioretinal atrophy)	Chr7: 112.681339	5.970	45	8.6	Chr3: 129.497470	0.148
35 ☐	MA_M2M_0706_R	1433862_at	*Espl1*	extra spindle poles-like 1 (S. cerevisiae)	Chr15: 102.324051	6.435	45	9.1	Chr3: 69.025720	0.055
36 ☐	MA_M2M_0706_R	1460612_at	*Ranbp3*	RAN binding protein 3	--	7.075	45	9.7	Chr1: 82.904438	0.101
37 ☐	MA_M2M_0706_R	1444430_at	*Armc8*	armadillo repeat containing 8	Chr9: 99.506820	6.708	45	13.4	Chr6: 16.056743	-0.131
38 ☐	MA_M2M_0706_R	1442277_at	*Chka*	choline kinase alpha; last intron or 3' UTR	Chr19: 3.865325	8.170	45	8.2	Chr2: 69.724603	0.171
39 ☐	MA_M2M_0706_R	1456340_at	*2610205E22Rik*	RIKEN cDNA 2610205E22 gene	Chr2: 30.817111	8.020	45	11.7	Chr2: 78.100320	0.207
40 ☐	MA_M2M_0706_R	1457233_at	*Dnaja2*	DnaJ (Hsp40) homolog, subfamily A, member 2	Chr8: 85.554238	8.731	45	17.9	Chr8: 83.183208	-0.170
41 ☐	MA_M2M_0706_R	1446508_at	*D430017M14Rik*	13 days embryo male testis cDNA, RIKEN full-length enriched library, clone:6030447N08 product:unknown EST, full insert sequence.	Chr15: 68.343881	7.051	45	17.1	Chr4: 88.045738	-0.120
42 ☐	MA_M2M_0706_R	1432980_at	*Dock4*	dedicator of cytokinesis 4	Chr12: 40.770053	5.977	45	8.9	Chr6: 54.397647	0.062
43 ☐	MA_M2M_0706_R	1451494_at	*Wac*	WW domain containing adaptor with coiled-coil; exons 12 and 14, and proximal 3' UTR	Chr18: 7.926111	10.545	45	11.2	Chr18: 61.000000	-0.172
44 ☐	MA_M2M_0706_R	1449292_at	*Rb1cc1*	RB1-inducible coiled-coil 1; exon 13	Chr1: 6.248798	7.287	45	7.9	Chr7: 6.024860	0.269
45 ☐	MA_M2M_0706_R	1447109_at	*Elavl1*	ELAV (embryonic lethal, abnormal vision, Drosophila)-like 1 (Hu antigen R); intron	Chr8: 4.291945	6.584	45	8.2	Chr9: 110.638628	0.072
46 ☐	MA_M2M_0706_R	1459347_at	*C130032J12Rik*	16 days neonate cerebellum cDNA, RIKEN full-length enriched library, clone:9630040P08 product:hypothetical protein, full insert sequence.	Chr11: 77.912165	7.794	45	15.1	Chr17: 83.262662	-0.081

47 ☐	MA_M2M_ 0706_R	1421412 _at	*Gsc*	goosecoid	Chr12: 104.471487	6.27 1	45	11.5	Chr6: 23.372761	0.05 0
48 ☐	MA_M2M_ 0706_R	1425038 _at	*Slc22a19*	solute carrier family 22 (organic anion transporter), member 19	Chr19: 7.673214	12.5 74	45	18.0	Chr14: 74.747362	0.23 1
49 ☐	MA_M2M_ 0706_R	1425119 _at	*Oas1b*	2'-5' oligoadenylate synthetase 1B	Chr5: 120.823572	8.64 7	45	11.4	ChrX: 102.424804	0.10 2
50 ☐	MA_M2M_ 0706_R	1449825 _at	*Actrt1*	activity response to ethanol 4	ChrX: 46.329998	6.79 2	45	12.4	Chr6: 124.528197	0.08 9
51 ☐	MA_M2M_ 0706_R	1425565 _at	*Rest*	RE1-silencing transcription factor; last exon (5' end)	Chr5: 77.280782	9.44 6	45	8.4	Chr2: 18.263847	- 0.15 6
52 ☐	MA_M2M_ 0706_R	1423772 _x_at	*Slc25a5*	solute carrier family 25 (mitochondrial carrier, adenine nucleotide translocator), member 5; last two exons and 3' UTR	ChrX: 36.797736	16.0 17	45	12.0	Chr14: 39.833599	0.09 0
53 ☐	MA_M2M_ 0706_R	1460654 _at	*Slc30a3*	solute carrier family 30 (zinc transporter), member 3; 3' UTR	Chr5: 31.086248	8.82 2	45	12.0	Chr2: 70.613187	- 0.08 5
54 ☐	MA_M2M_ 0706_R	1418012 _at	*Sh3glb1*	SH3-domain GRB2-like B1 (endophilin); mid 3' UTR	Chr3: 144.686958	10.1 53	45	9.7	ChrX: 133.505754	- 0.16 9
55 ☐	MA_M2M_ 0706_R	1426586 _at	*Slc25a11*	solute carrier family 25 (mitochondrial carrier; oxoglutarate carrier), member 11; exons 5,6, and 8, and proximal 3' UTR	Chr11: 70.644618	13.2 09	45	13.3	Chr2: 104.427306	- 0.11 3
56 ☐	MA_M2M_ 0706_R	1427309 _at	*Pars2*	prolyl-tRNA synthetase (mitochondrial); 3' UTR	Chr4: 106.654706	8.91 0	45	12.3	Chr5: 21.026344	- 0.11 0
57 ☐	MA_M2M_ 0706_R	1423714 _at	*Asf1b*	ASF1 anti-silencing function 1 homolog B (histone chaperone); 3' UTR	Chr8: 83.969636	8.02 2	45	11.1	ChrX: 115.338127	0.07 3
58 ☐	MA_M2M_ 0706_R	1448842 _at	*Cdo1*	cysteine dioxygenase 1, cytosolic	Chr18: 46.713542	12.5 20	45	19.8	Chr18: 46.275839	0.24 0
59 ☐	MA_M2M_ 0706_R	1420247 _at	*Glb1l*	galactosidase, beta 1-like	Chr1: 75.199222	6.37 0	45	12.5	Chr6: 124.528197	0.04 5
60 ☐	MA_M2M_ 0706_R	1425156 _at	*Gbp7*	guanylate binding protein 7	Chr3: 142.546476	8.02 2	45	13.7	Chr3: 139.354330	0.15 1
61 ☐	MA_M2M_ 0706_R	1416309 _at	*Nusap1*	nucleolar and spindle associated protein 1	Chr2: 119.649197	6.99 3	45	15.9	Chr9: 107.650604	0.13 3

62 ☐	MA_M2M_0706_R	1423775_s_at	*Prc1*	protein regulator of cytokinesis 1	Chr7: 80.315784	7.776	45	9.4	Chr3: 65.524002	0.073
63 ☐	MA_M2M_0706_R	1454426_at	*4930519E07Rik*	RIKEN cDNA 4930519E07 gene	Chr5: 41.282301	7.171	45	10.8	Chr11: 74.110266	0.057
64 ☐	MA_M2M_0706_R	1420583_a_at	*Rora*	retinoic acid receptor-related orphan receptor alpha; last four exons and proximal 3' UTR	Chr9: 69.374033	9.232	45	13.6	Chr4: 96.896949	-0.202
65 ☐	MA_M2M_0706_R	1430820_a_at	*Bbx*	bobby sox HMG-BOX transcription factor; exons 6, 7, and 8	Chr16: 50.224892	7.643	45	11.2	Chr5: 25.445855	0.271
66 ☐	MA_M2M_0706_R	1458028_at	*LOC231291*	hypothetical protein LOC231291	Chr11: 78.846291	8.093	45	11.9	Chr6: 125.820102	0.077
67 ☐	MA_M2M_0706_R	1442603_at	*Pb1*	PAX interacting (with transcription-activation domain) protein 1	Chr14: 31.060130	6.178	45	8.6	Chr4: 5.599068	-0.086
68 ☐	MA_M2M_0706_R	1427703_at	*Pafah1b1*	platelet-activating factor acetylhydrolase, isoform 1b, beta 1 subunit	Chr11: 74.678355	7.406	45	7.9	Chr3: 151.382601	0.088
69 ☐	MA_M2M_0706_R	1439816_at	*4930418G15Rik*	RIKEN cDNA 4930418G15 gene; far 3' UTR or unknown neighboring gene	Chr1: 9.960201	7.671	45	47.9	Chr1: 10.162992	-0.715
70 ☐	MA_M2M_0706_R	1428040_at	*Onecut3*	one cut domain, family member 3	Chr10: 80.495963	6.030	45	13.5	Chr4: 88.045738	0.039
71 ☐	MA_M2M_0706_R	1441927_at	*Syt7*	synaptotagmin 7; distal 3' UTR	Chr19: 10.451478	8.169	45	8.2	Chr12: 37.639776	-0.198
72 ☐	MA_M2M_0706_R	1441580_at	*Sgpp2*	sphingosine-1-phosphate phosphatase 2	Chr1: 78.392722	7.537	45	11.8	Chr4: 131.999242	-0.078
73 ☐	MA_M2M_0706_R	1450650_at	*Myo10*	myosin X (conventional non-muscle myosin heavy chain IIB, May-Hegglin anomaly); last three exons and 3' UTR	Chr15: 25.808154	9.524	45	10.2	Chr1: 37.639250	-0.209
74 ☐	MA_M2M_0706_R	1456611_at	*Fam13a*	Precm1 protein, family with sequence similarity 13, member A; mid-distal 3' UTR	Chr6: 58.934091	10.482	45	14.0	Chr3: 65.524002	0.209
75 ☐	MA_M2M_0706_R	1453841_at	*Laf4*	lymphoid nuclear protein related to AF4	Chr1: 38.554285	5.980	45	10.6	Chr6: 43.826811	0.056

76 ☐	MA_M2M_0706_R	1424089_a_at	*Tcf4*	transcription factor 4; last three exons and proximal 3' UTR	Chr18: 69.681618	7.593	45	12.9	Chr10: 115.872274	0.131
77 ☐	MA_M2M_0706_R	1437862_at	*Rbm25*	RNA binding motif protein 25; distal 3' UTR of short form message	Chr12: 83.663292	9.099	45	16.1	Chr7: 6.024860	0.409
78 ☐	MA_M2M_0706_R	1449439_at	*Klf7*	Kruppel-like factor 7 (ubiquitous); far 3' UTR	Chr1: 64.032879	7.895	45	12.1	Chr5: 131.541036	-0.136
79 ☐	MA_M2M_0706_R	1415967_at	*Ndufv1*	NADH dehydrogenase (ubiquinone) flavoprotein 1, 51kDa (NADH:ubiquinone oxidoreductase complex I component); exons 7, 8, and 9 (of 10)	Chr19: 4.007837	14.021	45	11.7	ChrX: 114.159505	0.086
80 ☐	MA_M2M_0706_R	1442362_at	*Gm104*	predicted gene 104 downstream of Exo1; possible 3' UTR extension of Exo1	Chr1: 175.921524	6.516	45	13.0	Chr5: 127.781724	0.058
81 ☐	MA_M2M_0706_R	1444678_at	*2810403D21Rik*	RIKEN cDNA 2810403D21 gene	ChrX: 108.874391	5.599	45	14.1	Chr9: 105.774381	0.035
82 ☐	MA_M2M_0706_R	1415929_at	*Map1lc3b*	microtubule-associated protein 1 light chain 3 beta (autophagy-related ubiquitin-like modifier LC3 B); distal 3' UTR	Chr8: 121.597478	13.329	45	11.8	Chr3: 65.524002	0.102
83 ☐	MA_M2M_0706_R	1460151_at	--	polymorphic LTR element; NACHT, leucine rich repeat and PYD containing 1	Chr1: 13.621363	8.231	45	24.6	Chr1: 13.073341	-0.322
84 ☐	MA_M2M_0706_R	1434801_x_at	*Slc25a5*	solute carrier family 25 (mitochondrial carrier, adenine nucleotide translocator), member 5; last two exons and 3' UTR	ChrX: 36.797782	15.746	45	11.8	Chr17: 64.993854	-0.097
85 ☐	MA_M2M_0706_R	1457318_at	*A330008L17Rik*	RIKEN cDNA A330008L17 gene	Chr8: 99.421618	7.361	45	10.8	Chr10: 72.780330	-0.098
86 ☐	MA_M2M_0706_R	1456965_at	*Kcnc1*	potassium voltage gated channel, Shaw-related subfamily, member 1 (delayed rectifier, large fast spike repolarization current, TEA sensitive); antisense in 5' UTR or promoter	Chr7: 46.397015	8.154	45	11.8	Chr14: 74.919381	0.102

87	MA_M2M_0706_R	1432916_at	*5730407I07Rik*	RIKEN cDNA 5730407I07 gene	Chr15: 33.599002	6.720	45	5.4	Chr7: 120.814862	0.073
88	MA_M2M_0706_R	1451126_at	*Maf1*	MAF1 homolog, negative regulator of RNA polymerase III; last exon and proximal 3' UTR	Chr15: 76.353710	11.785	45	10.7	Chr2: 70.613187	-0.099
89	MA_M2M_0706_R	1455964_at	*Cdk12*	cyclin-dependent kinase 12; far 3' UTR	Chr11: 98.253037	7.124	45	12.6	Chr14: 39.196761	-0.083
90	MA_M2M_0706_R	1457041_at	*1700026B20Rik*	small nucleolar RNA host gene (non-protein coding) 10 (similar to human small Cajal body-specific RNA 13_	Chr12: 105.030533	7.387	45	9.7	Chr1: 37.639250	0.093
91	MA_M2M_0706_R	1430649_at	*Baalc*	brain and acute leukemia, cytoplasmic	Chr15: 38.949833	8.243	45	12.0	Chr2: 139.724059	-0.079
92	MA_M2M_0706_R	1415878_at	*Rrm1*	ribonucleotide reductase M1	Chr7: 102.468420	8.552	45	12.5	Chr1: 80.325854	0.073
93	MA_M2M_0706_R	1438634_x_at	*Lasp1*	LIM and SH3 protein 1; distal 3' UTR	Chr11: 97.838653	11.906	45	10.7	Chr2: 113.445288	0.199
94	MA_M2M_0706_R	1417587_at	*Timeless*	timeless homolog (Drosophila)	Chr10: 128.243314	6.721	45	10.9	Chr9: 85.995347	0.080
95	MA_M2M_0706_R	1456319_at	*Mcpt9*	mast cell protease 9	Chr14: 53.568242	7.141	45	10.5	Chr14: 117.936145	-0.219
96	MA_M2M_0706_R	1436746_at	*Prkwnk1*	protein kinase, lysine deficient 1; exons 4, 6 and 7	Chr6: 119.969525	9.367	45	9.9	Chr2: 18.263847	-0.355
97	MA_M2M_0706_R	1444563_at	*AW494124*	ESTs	Chr2: 167.348354	8.082	45	12.3	Chr2: 80.327515	-0.125
98	MA_M2M_0706_R	1448021_at	*Fam46c*	family with sequence similarity 46, member C (protein LOC54855); far 3' UTR (putative)	Chr3: 100.468088	6.889	45	14.0	Chr17: 71.025688	0.201
99	MA_M2M_0706_R	1448447_at	*Vps28*	vacuolar protein sorting 28; last 5 exons, intron 7, and 3' UTR	Chr15: 76.622156	11.886	45	13.0	Chr7: 71.410581	0.106
100	MA_M2M_0706_R	1443212_at	*Large*	like-glycosyltransferase; intron 1	Chr8: 73.329777	6.474	45	17.2	Chr10: 116.134021	0.055
101	MA_M2M_0706_R	1427302_at	*Enpp3*	ectonucleotide pyrophosphatase/phosphodiesterase 3	Chr10: 24.774787	10.693	45	21.0	Chr8: 86.388770	0.168

102 ☐	MA_M2M_0706_R	1460619_at	*4931419K03Rik*	RIKEN cDNA 4931419K03 gene	Chr1: 40.772316	8.816	45	10.6	ChrX: 50.932422	0.187
103 ☐	MA_M2M_0706_R	1422476_at	*Ifi30*	interferon gamma inducible protein 30	Chr8: 70.762817	11.763	45	11.5	Chr4: 88.045738	0.158
104 ☐	MA_M2M_0706_R	1430796_at	*Trim14*	tripartite motif-containing 14	Chr4: 46.509689	5.999	45	10.5	Chr5: 133.062783	0.032
105 ☐	MA_M2M_0706_R	1443544_at	*C18orf1*	transmembrane neocortically enriched protein with low density lipoprotein receptor domain, human chromosome 18 open reading frame 1	Chr18: 67.977763	7.044	45	14.7	Chr1: 91.100378	-0.099
106 ☐	MA_M2M_0706_R	1417025_at	*H2-Eb1*	histocompatibility 2, class II antigen E beta	Chr17: 34.314357	10.962	45	14.4	Chr8: 123.289925	-0.279
107 ☐	MA_M2M_0706_R	1458761_at	*Itpa*	inosine triphosphatase (nucleoside triphosphate pyrophosphatase)	Chr2: 130.665163	5.732	45	15.8	Chr15: 12.687693	-0.043
108 ☐	MA_M2M_0706_R	1420202_at	*S100a3*	Mus musculus transcribed sequence	--	5.577	45	12.5	Chr19: 27.531578	-0.043
109 ☐	MA_M2M_0706_R	1450646_at	*Cyp51a1*	cytochrome P450, family 51, subfamily A, polypeptide 1 (endoplasmic reticulum, lanosterol 14-alpha demethylase); proximal 3' UTR	Chr5: 4.082591	11.949	45	14.1	Chr12: 84.895759	0.217
110 ☐	MA_M2M_0706_R	1457443_at	*Gpatc2*	G patch domain containing 2	Chr1: 187.239847	5.972	45	10.0	Chr2: 79.343444	0.031
111 ☐	MA_M2M_0706_R	1442766_at	*Ppp4r1*	ESTs	Chr17: 65.819859	7.900	45	7.7	Chr8: 123.289925	-0.078
112 ☐	MA_M2M_0706_R	1426290_at	*1500031M22Rik*	RIKEN cDNA 1500031M22 gene	Chr13: 106.952343	7.086	45	11.0	Chr9: 105.774381	0.058
113 ☐	MA_M2M_0706_R	1416697_at	*Dpp4*	dipeptidylpeptidase 4; mid 3' UTR	Chr2: 62.331853	10.157	45	9.9	Chr4: 88.045738	0.176
114 ☐	MA_M2M_0706_R	1435290_x_at	*H2-Aa*	histocompatibility 2, class II antigen A, alpha	Chr17: 34.282793	12.100	45	7.4	Chr16: 27.360729	-0.376
115 ☐	MA_M2M_0706_R	1427701_a_at	*Six4*	sine oculis-related homeobox 4 homolog (Drosophila)	Chr12: 73.108658	6.335	45	12.5	Chr10: 57.752461	-0.063
116 ☐	MA_M2M_0706_R	1444801_at	*2900041M22Rik*	RIKEN cDNA 2900041M22 gene	Chr11: 117.613259	5.836	45	10.6	Chr10: 55.101486	-0.031

117 ☐	MA_M2M_0706_R	1455013_at	*Arih2*	ariadne RBR E3 ubiquitin protein ligase 2	Chr9: 108.603136	9.527	45	14.8	Chr9: 107.831131	0.121
118 ☐	MA_M2M_0706_R	1444826_at	*4933427D06*	ESTs	Chr7: 78.892287	6.043	45	10.5	Chr8: 127.830308	0.053
119 ☐	MA_M2M_0706_R	1421595_at	*AB041544*	hypothetical protein, MNCb-2622; putative 3' UTR (from EST AB041544)	Chr17: 83.574143	6.111	45	10.4	Chr8: 117.545014	0.036
120 ☐	MA_M2M_0706_R	1451449_at	*4933407N01Rik*	RIKEN cDNA 4933407N01 gene	Chr11: 30.931644	10.554	45	11.5	Chr7: 36.693524	-0.159
121 ☐	MA_M2M_0706_R	1443870_at	*Abcc4*	ATP-binding cassette, sub-family C (CFTR/MRP), member 4; distal 3' UTR	Chr14: 118.482729	8.698	45	11.2	Chr13: 50.000000	0.218
122 ☐	MA_M2M_0706_R	1442072_at	*1110049L02Rik*	RIKEN cDNA 1110049L02 gene	Chr9: 56.203799	6.309	45	11.7	Chr16: 10.595499	-0.055
123 ☐	MA_M2M_0706_R	1428334_at	*Ostm1*	osteopetrosis associated transmembrane protein 1; distal 3'UTR	Chr10: 42.701869	10.087	45	15.7	Chr19: 8.707699	0.170
124 ☐	MA_M2M_0706_R	1447224_at	*Edg5*	endothelial differentiation, sphingolipid G-protein-coupled receptor 5; putative far 3' UTR (possible neighbor gene)	Chr9: 20.962394	6.096	45	10.5	Chr15: 101.561023	0.040
125 ☐	MA_M2M_0706_R	1418294_at	*Epb4.1l4b*	erythrocyte protein band 4.1-like 4b	Chr4: 57.061849	8.437	45	8.2	Chr15: 25.698695	-0.119
126 ☐	MA_M2M_0706_R	1455980_a_at	*8430435B07Rik*	ESTs, Weakly similar to GAS2_MOUSE Growth-arrest-specific protein 2 (GAS-2) [M.musculus]	Chr10: 89.412598	5.951	45	11.4	Chr18: 82.255306	0.048
127 ☐	MA_M2M_0706_R	1418605_at	*Nr2c1*	nuclear receptor subfamily 2, group C, member 1; last exon and 3' UTR	Chr10: 94.195174	6.877	45	9.4	Chr18: 80.000000	0.080
128 ☐	MA_M2M_0706_R	1434649_at	*Ccm2*	cerebral cavernous malformation 2 homolog; last exon and proximal half of 3' UTR	Chr11: 6.596167	8.359	45	12.5	Chr15: 12.672705	-0.058
129 ☐	MA_M2M_0706_R	1458415_at	*Clec2e*	C-type lectin domain family 2, member e	Chr6: 146.783700	7.753	45	12.4	Chr7: 46.356949	0.090

130 ☐	MA_M2M_0706_R	1427748_at	*Igh-4*	immunoglobulin heavy chain 4 (serum IgG1)	--	5.748	45	13.9	Chr16: 3.500000	-0.040
131 ☐	MA_M2M_0706_R	1415741_at	*Tmem165*	transmembrane protein 165; 3' UTR	Chr5: 76.208683	11.195	45	7.9	Chr5: 45.290189	0.128
132 ☐	MA_M2M_0706_R	1459554_at	*Otoa*	ESTs, Weakly similar to L1 repeat, Tf subfamily, member 30 [] [M.musculus]	Chr11: 41.313788	5.784	45	10.8	Chr13: 105.184837	0.042
133 ☐	MA_M2M_0706_R	1445038_at	*C730043O17*	hypothetical protein C730043O17	Chr18: 35.876721	7.222	45	13.9	ChrX: 9.334336	-0.047
134 ☐	MA_M2M_0706_R	1422775_at	*Blk*	B lymphoid kinase (oncogene); last exon and proximal half of 3' UTR	Chr14: 63.373004	6.880	45	9.3	Chr9: 98.579310	-0.068
135 ☐	MA_M2M_0706_R	1451408_at	*Trub2*	TruB pseudouridine (psi) synthase homolog 2 (E. coli); proximal 3' UTR	Chr2: 29.777041	8.272	45	11.2	Chr17: 57.106511	0.066
136 ☐	MA_M2M_0706_R	1423527_at	*4921510H08Rik*	RIKEN cDNA 4921510H08 gene	Chr10: 97.694208	5.788	45	11.1	Chr19: 32.357503	-0.040
137 ☐	MA_M2M_0706_R	1425776_a_at	*C87436*	expressed sequence C87436	Chr6: 86.469778	7.815	45	10.8	Chr9: 115.524788	0.097
138 ☐	MA_M2M_0706_R	1430992_s_at	*Cisd2*	CDGSH iron sulfur domain 2	Chr3: 135.407040	7.742	45	6.7	Chr8: 126.582424	0.129
139 ☐	MA_M2M_0706_R	1428164_at	*Nudt9*	nudix (nucleoside diphosphate linked moiety X)-type motif 9	Chr5: 104.061732	10.994	45	10.7	Chr15: 25.732086	-0.128
140 ☐	MA_M2M_0706_R	1451440_at	*Chodl*	chondrolectin	Chr16: 78.951211	6.772	45	10.1	Chr9: 29.939029	0.083
141 ☐	MA_M2M_0706_R	1423767_at	*Aip1*	A interacting protein 1; last three exons and proximal 3' UTR	Chr17: 74.299611	10.838	45	11.3	Chr9: 107.639250	0.110
142 ☐	MA_M2M_0706_R	1438858_x_at	*H2-Aa*	histocompatibility 2, class II antigen A, alpha	Chr17: 34.282770	11.943	45	10.3	Chr2: 134.519309	-0.314
143 ☐	MA_M2M_0706_R	1446111_at	*D14Ertd611e*	DNA segment, Chr 14, ERATO Doi 611, expressed	Chr14: 93.948174	5.980	45	12.5	Chr5: 118.917989	-0.042
144 ☐	MA_M2M_0706_R	1436038_a_at	*Dscr5*	Down syndrome critical region homolog 5 (human)	Chr16: 94.364735	11.777	45	14.2	Chr12: 107.923472	0.128
145 ☐	MA_M2M_0706_R	1430490_at	*Tcfap2a*	transcription factor AP-2, alpha; antisense in 5' UTR (from AK017409)	Chr13: 40.734609	6.173	45	8.8	Chr4: 88.807538	0.045

146 ☐	MA_M2M_0706_R	1431297_a_at	*Crnde*	colorectal neoplasia differentially expressed (non-protein coding)	Chr8: 92.326387	6.501	45	18.7	Chr18: 78.806816	-0.079
147 ☐	MA_M2M_0706_R	1429390_at	*Acpl2*	acid phosphatase-like 2	Chr9: 96.823644	7.330	45	9.1	Chr18: 78.806816	0.124
148 ☐	MA_M2M_0706_R	1453149_at	*Mftc*	major facilitator superfamily domain containing 1	Chr15: 39.095072	7.004	45	9.9	Chr9: 95.329897	0.085
149 ☐	MA_M2M_0706_R	1459271_at	*Cetn2*	centrin 2	ChrX: 72.911975	6.607	45	11.9	Chr18: 78.806816	-0.063
150 ☐	MA_M2M_0706_R	1451296_x_at	*Pabpc4*	poly A binding protein, cytoplasmic 4; last exon and 3' UTR	Chr4: 123.297871	9.987	45	12.6	Chr17: 21.686210	0.101
151 ☐	MA_M2M_0706_R	1428812_at	*1700040L02Rik*	RIKEN cDNA 1700040L02 gene	Chr10: 68.431234	7.569	45	12.7	Chr13: 101.209232	0.151
152 ☐	MA_M2M_0706_R	1417559_at	*Sfxn1*	sideroflexin 1 (flexed tail, tricarboxylate carrier protein, iron homeostasis)	Chr13: 54.093053	12.597	45	7.7	Chr18: 76.826488	0.184
153 ☐	MA_M2M_0706_R	1449047_at	*1600020H07Rik*	RIKEN cDNA 1600020H07 gene	Chr14: 31.615360	9.381	45	16.7	Chr7: 6.677334	0.378
154 ☐	MA_M2M_0706_R	1452423_at	*Pclo*	piccolo (presynaptic cytomatrix protein); last three exons and proximal 3' UTR	Chr5: 14.855613	5.643	45	19.3	Chr5: 141.387458	-0.045
155 ☐	MA_M2M_0706_R	1452237_at	*Hrb*	HIV-1 Rev binding protein; mid-distal 3' UTR	Chr1: 82.895811	7.792	45	11.6	Chr18: 80.000000	0.206
156 ☐	MA_M2M_0706_R	1452937_s_at	*Ccdc28b*	coiled coil domain containing 28B; exon	Chr4: 129.620698	7.524	45	15.8	Chr19: 35.573941	-0.123
157 ☐	MA_M2M_0706_R	1443042_at	*D130047N11Rik*	RIKEN cDNA D130047N11 gene	Chr16: 72.817619	6.795	45	11.7	Chr9: 95.329897	-0.066
158 ☐	MA_M2M_0706_R	1428213_at	*2410003A14Rik*	RIKEN cDNA 2410003A14 gene	Chr7: 130.533576	11.436	45	15.4	Chr4: 88.045738	0.114
159 ☐	MA_M2M_0706_R	1448883_at	*Lgmn*	legumain cysteine protease; last two exons and proximal 3' UTR	Chr12: 102.394497	13.431	45	10.4	Chr15: 91.405127	0.186
160 ☐	MA_M2M_0706_R	1450759_at	*Bmp6*	bone morphogenetic protein 6	Chr13: 38.499601	10.572	45	12.3	Chr2: 149.665875	-0.199
161 ☐	MA_M2M_0706_R	1431008_at	*H2-Q1*	histocompatibility 2, Q region locus 1	Chr17: 35.384916	10.084	45	21.0	Chr13: 110.449360	-0.462

162 ☐	MA_M2M_0706_R	1425868_at	*Hist2h2bb*	histone cluster 2, H2bb; far 3' UTR (long form)	Chr3: 96.278524	6.564	45	13.8	Chr18: 78.806816	0.058
163 ☐	MA_M2M_0706_R	1452200_at	*Cdkn2aipnl*	CDKN2A interacting protein N-terminal like; mid distal 3' UTR	Chr11: 51.976754	8.617	45	11.9	Chr13: 111.021029	0.126
164 ☐	MA_M2M_0706_R	1416963_at	*Ubadc1*	ubiquitin associated domain containing 1	Chr2: 25.998617	10.703	45	16.0	Chr12: 86.105698	0.138
165 ☐	MA_M2M_0706_R	1451125_at	*Paip2b*	poly(A) binding protein interacting protein 2B; distal 3' UTR	Chr6: 83.806140	8.694	45	11.2	Chr12: 117.389926	-0.128
166 ☐	MA_M2M_0706_R	1430794_at	*4933426K07Rik*	RIKEN cDNA 4933426K07 gene	Chr11: 57.655491	7.035	45	10.6	Chr16: 26.422169	0.070
167 ☐	MA_M2M_0706_R	1452246_at	*Ostf1*	osteoclast stimulating factor 1	Chr19: 18.584675	10.691	45	28.3	Chr19: 18.172211	0.253
168 ☐	MA_M2M_0706_R	1437151_at	*Usp22*	ubiquitin specific protease 22	Chr11: 61.152155	10.093	45	16.3	Chr19: 28.403278	-0.126
169 ☐	MA_M2M_0706_R	1427699_a_at	*Ptpn11*	protein tyrosine phosphatase, non-receptor type 11	Chr5: 121.143016	7.744	45	10.3	Chr7: 26.713260	0.089
170 ☐	MA_M2M_0706_R	1444048_at	*AI666576*	ESTs	Chr9: 22.440388	7.059	45	12.6	Chr13: 110.449360	-0.064
171 ☐	MA_M2M_0706_R	1450192_at	*Lhcgr*	luteinizing hormone/choriogonadotropin receptor	Chr17: 88.741574	5.952	45	10.4	Chr4: 86.450967	-0.033
172 ☐	MA_M2M_0706_R	1417616_at	*St6galnac2*	ST6 (alpha-N-acetyl-neuraminyl-2,3-beta-galactosyl-1,3)-N-acetylgalactosaminide alpha-2,6-sialyltransferase 2	Chr11: 116.676736	11.572	45	10.4	Chr1: 3.010274	0.116
173 ☐	MA_M2M_0706_R	1417935_at	*Mkrn2*	makorin, ring finger protein, 2	Chr6: 115.618089	9.496	45	9.9	Chr5: 117.789650	-0.119
174 ☐	MA_M2M_0706_R	1454694_a_at	*Top2a*	topoisomerase (DNA) II alpha; last exons and 3' UTR	Chr11: 98.993073	6.912	45	9.1	Chr9: 3.400000	-0.131
175 ☐	MA_M2M_0706_R	1416029_at	*Tieg1*	TGFB inducible early growth response 1; distal 3' UTR	Chr15: 38.294544	7.459	45	9.6	Chr19: 44.599198	-0.172
176 ☐	MA_M2M_0706_R	1424607_a_at	*LOC432823*	endogenous retroviral sequence MGC37588; repetitive element	ChrUn: 1.000000	11.045	45	11.9	Chr3: 41.542098	0.269
177 ☐	MA_M2M_0706_R	1451005_at	*Sumo1*	small ubiquitin-like modifier 1 (SMT3 suppressor of mif two 3 homolog 1);	Chr1: 59.639734	12.000	45	11.6	Chr5: 127.617133	0.112

				proximal and mid 3' UTR						
178 ☐	MA_M2M_0706_R	1452134_at	*Tmem175*	transmembrane protein 175; last exon and proximal 3' UTR	Chr5: 108.646099	8.506	45	10.1	Chr18: 78.806816	0.118
179 ☐	MA_M2M_0706_R	1415753_at	*Fam108a*	abhydrolase domain-containing protein FAM108A; last two exons and proximal 3' UTR	Chr10: 80.583725	11.533	45	7.1	Chr17: 10.720847	-0.122
180 ☐	MA_M2M_0706_R	1451825_a_at	*Copz1*	coatomer protein complex, subunit zeta 1	Chr15: 103.299251	11.799	45	12.5	Chr12: 36.689834	0.086
181 ☐	MA_M2M_0706_R	1434771_at	*0610011F06Rik*	RIKEN cDNA 0610011F06 gene	Chr17: 25.876764	12.779	45	13.8	Chr18: 15.638699	-0.117
182 ☐	MA_M2M_0706_R	1436657_at	*Prcd*	progressive rod-cone degeneration (pineal and retinal photoreceptor associated); exon and 3' UTR (from AK137302)	Chr11: 116.657560	7.849	45	8.5	Chr7: 114.699004	0.059
183 ☐	MA_M2M_0706_R	1416030_a_at	*Mcm7*	minichromosome maintenance deficient 7 (S. cerevisiae)	Chr5: 138.165990	9.862	45	14.6	Chr12: 9.742519	0.168
184 ☐	MA_M2M_0706_R	1424003_at	*Sgk196*	protein kinase-like protein Sugen kinase 196; mid 3' UTR	Chr8: 25.981541	6.593	45	14.4	Chr1: 107.486168	0.058
185 ☐	MA_M2M_0706_R	1435628_x_at	*A430108B07Rik*	RIKEN cDNA A430108B07 gene	Chr10: 25.895642	7.881	45	14.3	Chr14: 117.841013	-0.216
186 ☐	MA_M2M_0706_R	1421776_at	*Olfr74*	olfactory receptor 74; exon	Chr2: 87.973725	5.988	45	13.6	Chr13: 103.151983	0.070
187 ☐	MA_M2M_0706_R	1417594_at	*Gkap1*	G kinase anchoring protein 1; last 5 exons and 3' UTR	Chr13: 58.233391	11.023	45	14.8	Chr4: 62.707256	0.162
188 ☐	MA_M2M_0706_R	1418629_a_at	*Khdrbs1*	KH domain containing, RNA binding, signal transduction associated 1; mid distal 3' UTR	Chr4: 129.715175	9.678	45	10.7	Chr18: 15.747404	0.126
189 ☐	MA_M2M_0706_R	1424128_x_at	*Aurkb*	aurora kinase B; mid 3' UTR	Chr11: 69.051092	6.399	45	17.0	Chr17: 63.598705	0.046
190 ☐	MA_M2M_0706_R	1430617_at	*5730547N13Rik*	RIKEN cDNA 5730547N13 gene	Chr2: 119.609857	6.428	45	8.3	Chr9: 103.625467	0.034
191 ☐	MA_M2M_0706_R	1448429_at	*Gyg1*	glycogenin 1; proximal to mid 3' UTR	Chr3: 20.122324	10.175	45	11.3	Chr9: 107.639250	0.100

192	MA_M2M_0706_R	1428803_at	*Acot6*	acyl-coenzyme A thioesterase 6	Chr12: 84.110781	7.725	45	9.7	ChrX: 37.500000	0.103
193	MA_M2M_0706_R	1448200_at	*Tcn2*	transcobalamin 2; last 2 exons and 3' UTR	Chr11: 3.917372	16.251	45	15.8	Chr15: 91.405127	0.127
194	MA_M2M_0706_R	1429556_at	*Tead1*	TEA domain family member 1 (SV40 transcriptional enhancer factor, Sveinsson's chorioretinal atrophy); distal 3'UTR	Chr7: 112.905932	10.670	45	11.0	Chr15: 94.266750	-0.156
195	MA_M2M_0706_R	1449067_at	*Slc2a2*	solute carrier family 2 (facilitated glucose transporter), member 2; distal 3' UTR	Chr3: 28.727844	13.426	45	13.1	Chr17: 21.686210	0.282
196	MA_M2M_0706_R	1452582_at	*Galm*	galactose mutarotase; mid 3' UTR	Chr17: 80.184508	12.125	45	12.1	ChrX: 114.159505	0.125
197	MA_M2M_0706_R	1418517_at	*Irx3*	Iroquois related homeobox 3	Chr8: 91.798530	11.401	45	8.5	Chr18: 16.414681	-0.151
198	MA_M2M_0706_R	1416901_at	*Npc2*	Niemann Pick type C2; proximal 3' UTR	Chr12: 84.756822	12.119	45	11.1	ChrX: 114.159505	0.083
199	MA_M2M_0706_R	1460575_at	*D3Ertd194e*	DNA segment, Chr 3, ERATO Doi 194, expressed	Chr3: 58.555538	8.811	45	12.5	Chr15: 95.949674	0.123
200	MA_M2M_0706_R	1456130_at	*Pcdh7*	protocadherin 7	Chr6: 12.311625	6.255	45	14.4	Chr15: 93.021696	-0.103
201	MA_M2M_0706_R	1423068_at	*Ift172*	intraflagellar transport 172 protein; 4 of last 5 exons and proximal 3' UTR	Chr5: 31.253314	9.056	45	12.5	Chr10: 13.687795	-0.138
202	MA_M2M_0706_R	1460221_at	*Ptges3*	prostaglandin E synthase 3 (cytosolic); mid-proximal 3' UTR	Chr10: 128.076319	12.499	45	11.2	Chr15: 94.266750	-0.201
203	MA_M2M_0706_R	1426990_at	*Cubn*	cubilin (intrinsic factor-cobalamin receptor)	Chr2: 13.278386	13.765	45	17.9	ChrX: 102.126656	0.214
204	MA_M2M_0706_R	1420618_at	*Cpeb4*	cytoplasmic polyadenylation element binding protein 4; distal 3' UTR	Chr11: 31.935120	13.126	45	10.1	Chr3: 113.087963	0.187
205	MA_M2M_0706_R	1427705_a_at	*Nfkb1*	nuclear factor of kappa light chain gene enhancer in B-cells 1, p105; mid proximal 3' UTR	Chr3: 135.584947	9.357	45	14.9	Chr1: 178.156680	0.085

206 ☐	MA_M2M_0706_R	1438718_at	*Fgf9*	fibroblast growth factor 9; distal half of 3' UTR	Chr14: 58.109862	7.584	45	9.4	ChrX: 102.126656	-0.129
207 ☐	MA_M2M_0706_R	1425020_at	*Ubxd2a*	UBX domain protein 2A (p97 adaptor protein); far 3' UTR	Chr12: 4.877133	13.142	45	16.4	Chr11: 62.251912	0.118
208 ☐	MA_M2M_0706_R	1438221_at	*C130065N10Rik*	RIKEN cDNA C130065N10 gene	Chr1: 58.522945	9.708	45	18.7	Chr1: 58.115573	-0.239
209 ☐	MA_M2M_0706_R	1422441_x_at	*Cdk4*	cyclin-dependent kinase 4; last two exons and proximal 3' UTR	Chr10: 127.066179	10.275	45	17.7	Chr13: 53.477510	0.158
210 ☐	MA_M2M_0706_R	1460406_at	*Trpc1*	transient receptor potential cation channel, subfamily C, member 1	Chr9: 95.752943	11.106	45	9.8	Chr11: 99.734855	0.134
211 ☐	MA_M2M_0706_R	1453623_a_at	*Rad23a*	RAD23a homolog (S. cerevisiae)	Chr8: 84.840492	7.922	45	12.4	Chr8: 80.868085	0.239
212 ☐	MA_M2M_0706_R	1454609_x_at	*6430527G18Rik*	RIKEN cDNA 6430527G18 gene	Chr12: 86.881028	9.633	45	8.5	Chr13: 53.477510	-0.112
213 ☐	MA_M2M_0706_R	1438937_x_at	*Ang*	angiogenin, ribonuclease A family, member 1; last (only) exon and proximal 3' UTR	Chr14: 51.101813	6.469	45	10.8	Chr18: 76.870082	0.177
214 ☐	MA_M2M_0706_R	1433722_at	*5730522G15Rik*	RIKEN cDNA 5730522G15 gene	Chr7: 75.754024	10.460	45	7.6	Chr4: 40.731591	0.112
215 ☐	MA_M2M_0706_R	1426656_at	*4930504E06Rik*	RIKEN cDNA 4930504E06 gene	Chr3: 95.295369	11.232	45	15.5	Chr7: 31.360167	-0.131
216 ☐	MA_M2M_0706_R	1453851_a_at	*Gadd45g*	growth arrest and DNA-damage-inducible 45 gamma	Chr13: 51.847877	9.356	45	13.0	Chr5: 77.779809	-0.305
217 ☐	MA_M2M_0706_R	1423809_at	*Tcf19*	transcription factor 19	Chr17: 35.512810	7.752	45	20.9	Chr9: 3.400000	-0.102
218 ☐	MA_M2M_0706_R	1452148_at	*Lrpap1*	low density lipoprotein receptor-related protein associated protein 1; distal 3' UTR	Chr5: 35.091531	11.408	45	14.0	Chr2: 80.327515	-0.154
219 ☐	MA_M2M_0706_R	1433622_at	*Gemin4*	gem (nuclear organelle) associated protein 4	Chr11: 76.210878	7.866	45	10.1	Chr7: 3.078244	-0.097
220 ☐	MA_M2M_0706_R	1440037_at	*Pbx1*	pre B-cell leukemia transcription factor 1; 3' UTR of short form message (intron 2)	Chr1: 168.424573	10.578	45	13.7	Chr3: 27.192039	0.282

221 ☐	MA_M2M_0706_R	1451276_at	*Uhrf1bp1l*	UHRF1 (ICBP90) binding protein 1-like	Chr10: 89.819273	10.926	45	7.9	Chr2: 109.782694	-0.116
222 ☐	MA_M2M_0706_R	1434701_at	*Alkbh5*	alkB, alkylation repair homolog 5; distal 3' UTR	Chr11: 60.557920	12.396	45	9.9	Chr18: 82.106750	-0.119
223 ☐	MA_M2M_0706_R	1437117_at	*Centb1*	centaurin, beta 1	Chr11: 69.882381	8.483	45	11.2	Chr15: 62.238376	0.092
224 ☐	MA_M2M_0706_R	1418146_a_at	*Rbl2*	retinoblastoma-like 2; proximal and mid 3' UTR	Chr8: 91.122460	9.162	45	13.4	Chr18: 76.826488	0.191
225 ☐	MA_M2M_0706_R	1423643_at	*Ddx39*	DEAD (Asp-Glu-Ala-Asp) box polypeptide 39	Chr8: 83.722458	9.361	45	13.2	Chr15: 7.167980	-0.174
226 ☐	MA_M2M_0706_R	1457949_at	*Nt5c2*	5'-nucleotidase, cytosolic II; putative exon	Chr19: 46.885497	7.837	45	20.5	Chr11: 62.251912	-0.122
227 ☐	MA_M2M_0706_R	1421631_at	*Pcdhb1*	protocadherin beta 1	Chr18: 37.266924	6.484	45	9.1	Chr13: 108.957958	0.048
228 ☐	MA_M2M_0706_R	1441208_at	*Hdhd2*	haloacid dehalogenase-like hydrolase domain containing; putative deep 3' UTR	Chr18: 76.973281	8.228	45	13.8	Chr11: 73.268056	-0.101
229 ☐	MA_M2M_0706_R	1449481_at	*Slc25a13*	solute carrier family 25 (mitochondrial carrier, adenine nucleotide translocator), member 13 (citrin deficiency associated)	Chr6: 6.041795	11.836	45	18.1	Chr6: 23.242013	0.192
230 ☐	MA_M2M_0706_R	1435860_at	*Slc5a6*	solute carrier family 5 (sodium-dependent vitamin transporter), member 6	Chr5: 31.036558	8.135	45	9.3	Chr8: 127.830308	0.172
231 ☐	MA_M2M_0706_R	1434748_at	*Ckap2*	cytoskeleton associated protein 2	Chr8: 22.168836	6.856	45	11.9	Chr15: 7.167980	-0.061
232 ☐	MA_M2M_0706_R	1455939_x_at	*Srp14*	signal recognition particle 14	Chr2: 118.475915	12.318	45	7.4	Chr2: 109.782694	0.138
233 ☐	MA_M2M_0706_R	1415917_at	*Mthfd1*	methylenetetrahydrofolate dehydrogenase (NADP+ dependent), methenyltetrahydrofolate cyclohydrolase, formyltetrahydrofolate synthase; last 3 exons	Chr12: 76.314424	12.892	45	11.4	Chr7: 73.746984	0.098
234 ☐	MA_M2M_0706_R	1436583_at	*2600005O03Rik*	RIKEN cDNA 2600005O03 gene	Chr15: 75.538760	5.994	45	11.1	Chr2: 109.782694	0.040

235 ☐	MA_M2M_0706_R	1416644_a_at	*Sema3b*	sema domain, immunoglobulin domain (Ig), short basic domain, secreted, (semaphorin) 3B; exons 12 through 16	Chr9: 107.599602	8.654	45	10.3	ChrX: 47.876769	0.105
236 ☐	MA_M2M_0706_R	1433159_at	*5330429L19Rik*	RIKEN cDNA 5330429L19 gene	Chr14: 64.653515	6.798	45	8.7	Chr13: 106.343092	-0.070
237 ☐	MA_M2M_0706_R	1450777_at	*Xrn2*	5'-3' exoribonuclease 2; exons 22, 23, 24, and 25	Chr2: 147.061459	8.907	45	17.0	Chr15: 93.021696	0.137
238 ☐	MA_M2M_0706_R	1456878_at	*AI646023*	expressed sequence AI646023	Chr10: 75.550162	6.319	45	12.0	Chr15: 67.990415	0.065
239 ☐	MA_M2M_0706_R	1426593_a_at	*Fbxo22*	F-box only protein 22	Chr9: 55.224277	12.590	45	11.6	Chr11: 62.251912	0.123
240 ☐	MA_M2M_0706_R	1425025_at	*Tmem106a*	transmembrane protein 106A; last two exons and proximal 3' UTR	Chr11: 101.589980	12.294	45	14.4	Chr15: 93.021696	0.200
241 ☐	MA_M2M_0706_R	1442397_at	*Nfx1*	nuclear transcription factor, X-box binding 1	Chr4: 41.013293	7.413	45	14.3	Chr15: 94.266750	0.080
242 ☐	MA_M2M_0706_R	1439364_a_at	*Mmp2*	matrix metalloproteinase 2; mid distal 3' UTR	Chr8: 92.853173	7.741	45	19.8	Chr8: 90.648338	0.158
243 ☐	MA_M2M_0706_R	1437267_x_at	*Hnrnph1*	heterogeneous nuclear ribonucleoprotein H1; distal 3' UTR	Chr11: 50.386367	7.915	45	9.6	ChrX: 42.224690	-0.092
244 ☐	MA_M2M_0706_R	1423050_s_at	*Hnrnpu*	heterogeneous nuclear ribonucleoprotein U; distal 3' UTR	Chr1: 178.328459	12.639	45	9.0	Chr4: 45.489430	0.108
245 ☐	MA_M2M_0706_R	1451108_at	*Rnf185*	ring finger protein 185	Chr11: 3.416211	9.874	45	10.4	Chr18: 78.806816	0.116
246 ☐	MA_M2M_0706_R	1459092_at	*Slco6c1*	Mus musculus transcribed sequence with weak similarity to protein sp:Q9Y6L6 (H.sapiens) OAT6_HUMAN Liver-specific organic anion transporter (Organic anion transport polypeptide 2) (OATP 2)	Chr1: 97.081173	5.680	45	10.8	Chr4: 120.293959	0.040
247 ☐	MA_M2M_0706_R	1436542_at	*Ptger1*	prostaglandin E receptor 1 (subtype EP1)	Chr8: 83.666864	9.830	45	15.1	Chr8: 80.868085	-0.144
248 ☐	MA_M2M_0706_R	1416340_a_at	*Man2b1*	mannosidase 2, alpha B1; last four exons and 3' UTR	Chr8: 85.096414	13.168	45	14.2	ChrX: 102.126656	0.132

249	MA_M2M_0706_R	1428270_at	*Glt8d1*	glycosyltransferase 8 domain containing 1; last 2 exons and proximal 3' UTR	Chr14: 31.011163	9.776	45	8.2	Chr3: 28.727954	-0.128
250	MA_M2M_0706_R	1446538_at	*Kpna3*	karyopherin (importin) alpha 3; intron (from EST AK083505)	Chr14: 61.405964	7.335	45	9.7	Chr6: 24.108037	-0.075
251	MA_M2M_0706_R	1438740_at	*Nmt2*	N-myristoyltransferase 2	Chr2: 3.326553	7.543	45	13.8	Chr8: 123.289925	-0.094
252	MA_M2M_0706_R	1419975_at	*Scp2*	sterol carrier protein 2, liver; mid-distal 3' UTR	Chr4: 108.043986	10.166	45	10.2	ChrX: 50.730573	-0.297
253	MA_M2M_0706_R	1438198_at	*Bri3bp*	BRI3 binding protein (cervical cancer oncogene binding protein); distal 3' UTR	Chr5: 125.459423	8.235	45	11.2	Chr18: 58.397406	0.090
254	MA_M2M_0706_R	1452193_a_at	*Wasl*	Wiskott-Aldrich syndrome-like; distal 3' UTR	Chr6: 24.613837	12.234	45	15.2	Chr11: 62.251912	0.162
255	MA_M2M_0706_R	1423670_a_at	*Srpr*	signal recognition particle receptor (ER docking protein); mid to distal 3' UTR	Chr9: 35.216428	12.399	45	10.3	ChrX: 102.424804	0.078
256	MA_M2M_0706_R	1454611_a_at	*Calm1*	calmodulin 1; proximal to mid 3'UTR	Chr12: 100.207086	13.046	45	12.8	ChrX: 102.126656	-0.143
257	MA_M2M_0706_R	1454296_at	*Ralbp1*	ralA binding protein 1	Chr17: 65.871706	6.782	45	11.5	Chr1: 21.638463	-0.048
258	MA_M2M_0706_R	1422801_at	*G3bp*	Ras-GTPase-activating protein SH3-domain binding protein (interferon, alpha-inducible protein); mid 3' UTR	Chr11: 55.499735	11.763	45	7.4	Chr11: 99.734855	-0.142
259	MA_M2M_0706_R	1454495_at	*4930432H08Rik*	RIKEN cDNA 4930432H08 gene	Chr5: 106.364646	6.507	45	9.6	Chr17: 43.098144	0.063
260	MA_M2M_0706_R	1417810_a_at	*Pacsin2*	protein kinase C and casein kinase substrate in neurons 2; distal 3' UTR	Chr15: 83.376200	12.514	45	9.8	Chr17: 10.720847	0.127
261	MA_M2M_0706_R	1426456_a_at	*Miz1*	Msx-interacting-zinc finger; exons 8, 9, 10, and 11	Chr18: 77.133220	9.441	45	18.9	Chr18: 78.806816	0.147
262	MA_M2M_0706_R	1452734_at	*Rnaset2*	ribonuclease T2	Chr17: 6.988754	14.725	45	13.7	Chr17: 9.373070	0.112
263	MA_M2M_0706_R	1424036_at	*Prpf6*	PRP6 pre-mRNA splicing factor 6 homolog; last 4 exons	Chr2: 181.651122	10.056	45	11.7	Chr18: 78.806816	0.098

Supplemental Table S10. Top 200 genes closely correlated to the Ace2 probes in female mice.

	Dataset	Trait ID	Symbol	Description	Location	Mean	N Cases	Max LRS	Max LRS Location Chr and Mb	Add
1	MA_M2F_0706_R	1425103_at	*Ace2*	angiotensin I converting enzyme (peptidyl-dipeptidase A) 2; middle to distal 3'-UTR	ChrX: 164.187853	10.112	65	17.2	Chr16: 73.747956	-0.131
2	MA_M2F_0706_R	1422789_at	*Aldh1a2*	aldehyde dehydrogenase family 1, subfamily A2; 3' UTR	Chr9: 71.295684	7.918	65	18.5	Chr17: 21.686210	-0.180
3	MA_M2F_0706_R	1425102_a_at	*Ace2*	angiotensin I converting enzyme (peptidyl-dipeptidase A) 2; 3'-UTR and last 4 exons	ChrX: 164.182645	10.046	65	12.7	Chr14: 3.000000	0.186
4	MA_M2F_0706_R	1424209_at	*Rarsl*	arginyl-tRNA synthetase-like; exons 15, 16, 17, 18 19, 20	Chr4: 34.656065	10.679	65	14.4	Chr2: 170.826972	0.084
5	MA_M2F_0706_R	1420138_at	*Slc19a1*	solute carrier family 19 (sodium/hydrogen exchanger), member 1	Chr10: 77.050144	11.889	65	9.5	Chr15: 95.949674	0.113
6	MA_M2F_0706_R	1424828_a_at	*Fh1*	fumarate hydratase, mitochondrial (fumarase); exons 8, 9, and 10, and proximal 3' UTR	Chr1: 175.601417	13.455	65	12.0	Chr1: 163.338902	0.068
7	MA_M2F_0706_R	1415683_at	*Nmt1*	N-myristoyltransferase 1; exons 10 and 11, and 3' UTR	Chr11: 103.064733	11.775	65	11.8	Chr10: 24.000000	0.085
8	MA_M2F_0706_R	1434900_at	*Mkl1*	MKL (megakaryoblastic leukemia)/myocardin-like 1; mid distal 3' UTR	Chr15: 81.012534	9.424	65	16.6	Chr15: 91.405127	-0.104

9	MA_M2F_0706_R	1438018_at	*Hook1*	hook 1 (microtubule linker, endocytic membrane trafficking); distal 3' UTR	Chr4: 96.024792	11.882	65	12.1	Chr15: 91.405127	0.126
10	MA_M2F_0706_R	1428969_at	*Kiaa1012*	protein TRS85 homolog (ER-Golgi transport); 3' UTR	Chr18: 20.817479	11.054	65	11.4	Chr14: 3.000000	0.101
11	MA_M2F_0706_R	1448206_at	*Psma2*	proteasome (prosome, macropain) subunit, alpha type 2; last five exons and 3' UTR	Chr13: 14.619304	13.033	65	9.7	Chr16: 74.917702	-0.079
12	MA_M2F_0706_R	1452005_at	*Dlat*	dihydrolipoamide S-acetyltransferase (E2 component of pyruvate dehydrogenase complex); distal 3' UTR	Chr9: 50.634731	10.157	65	10.7	Chr9: 40.213083	-0.136
13	MA_M2F_0706_R	1419519_at	*Igf1*	insulin-like growth factor 1 (somatomedin C); 3' UTR	Chr10: 87.931240	7.422	65	8.9	Chr9: 99.750326	-0.153
14	MA_M2F_0706_R	1421019_at	*1700021F05Rik*	RIKEN cDNA 1700021F05; exons 1 and 3 and 3' UTR	Chr10: 43.525190	10.835	65	17.9	Chr15: 91.405127	0.148
15	MA_M2F_0706_R	1453880_s_at	*1700041C02Rik*	RIKEN cDNA 1700041C02 gene	Chr4: 119.377209	5.871	65	16.6	Chr9: 88.498812	0.035
16	MA_M2F_0706_R	1416472_at	*Syap1*	synapse associated protein 1; mid-distal 3' UTR	ChrX: 162.857076	12.641	65	9.9	Chr16: 85.961783	-0.079
17	MA_M2F_0706_R	1417054_a_at	*Sf3b14*	splicing factor 3B, 14 kDa subunit; last 2 exons and proximal 3' UTR	Chr12: 4.826831	12.053	65	10.6	Chr15: 91.405127	0.147
18	MA_M2F_0706_R	1415749_a_at	*Rragc*	Ras-related GTP binding C; mid-distal 3' UTR	Chr4: 123.936450	12.155	65	16.6	Chr9: 88.498812	0.093
19	MA_M2F_0706_R	1433545_s_at	*Acad11*	acyl-Coenzyme A dehydrogenase family, member 11; mid distal 3' UTR	Chr9: 104.127137	13.085	65	14.4	Chr9: 99.750326	0.129
20	MA_M2F_0706_R	1426823_s_at	*Psme4*	proteasome (prosome, macropain) activator subunit 4	Chr11: 30.879986	12.909	65	21.7	Chr11: 29.493209	-0.179
21	MA_M2F_0706_R	1437391_x_at	*Mrpl44*	mitochondrial ribosomal protein L44	Chr1: 79.781249	11.205	65	26.3	Chr15: 89.576205	0.191
22	MA_M2F_0706_R	1428645_at	*Gnai3*	guanine nucleotide binding protein, alpha inhibiting 3; distal 3' UTR	Chr3: 108.107540	11.397	65	14.1	Chr3: 134.523036	-0.140

23 ☐	MA_M2F_0706_R	1417827_at	*Ngly1*	N-glycanase 1; proximal and mid 3' UTR	Chr14: 16.311019	11.104	65	14.3	Chr8: 80.868085	-0.115
24 ☐	MA_M2F_0706_R	1417530_a_at	*Srp9*	signal recognition particle 9	Chr1: 182.131342	11.639	65	11.0	Chr8: 25.520489	-0.102
25 ☐	MA_M2F_0706_R	1423709_s_at	*Farslb*	phenylalanine-tRNA synthetase-like, beta subunit; last 2 exons	Chr1: 78.443633	9.873	65	15.0	Chr6: 42.000000	-0.130
26 ☐	MA_M2F_0706_R	1435178_x_at	*Anapc5*	anaphase-promoting complex subunit 5	Chr5: 11.351956	12.933	65	10.7	Chr1: 178.015151	0.065
27 ☐	MA_M2F_0706_R	1422854_at	*Shc1*	src homology 2 domain-containing transforming protein C1	Chr3: 89.429142	10.818	65	15.8	Chr15: 91.405127	-0.184
28 ☐	MA_M2F_0706_R	1448238_at	*C14orf166*	CLE7 homolog (UPF0568 protein); 3' half	Chr14: 19.812115	12.645	65	11.2	Chr16: 73.747956	-0.086
29 ☐	MA_M2F_0706_R	1424918_at	*Tbc1d19*	TBC1 domain family, member 19	Chr5: 53.887402	10.323	65	13.8	ChrX: 71.441039	-0.142
30 ☐	MA_M2F_0706_R	1448102_a_at	*Wdr61*	WD repeat domain 61; mid proximal 3' UTR	Chr9: 54.717273	11.646	65	9.4	Chr4: 96.896949	0.109
31 ☐	MA_M2F_0706_R	1452737_at	*Smim15*	small integral membrane protein 15; 3' UTR	Chr13: 108.048608	11.910	65	10.4	Chr10: 116.640226	-0.115
32 ☐	MA_M2F_0706_R	1417710_at	*Mettl9*	methyltransferase like 9; last exon and proximal half of 3' UTR	Chr7: 121.076249	12.659	65	11.3	Chr1: 73.418156	0.085
33 ☐	MA_M2F_0706_R	1423127_at	*Impa1*	inositol (myo)-1(or 4)-monophosphatase 1	Chr3: 10.314235	10.724	65	11.4	Chr3: 9.082402	-0.151
34 ☐	MA_M2F_0706_R	1420495_a_at	*Vps26*	vacuolar protein sorting 26 (retromer complex, involved in retrograde transport of proteins from endosomes to the trans-Golgi network); distal 3' UTR	Chr10: 62.455304	11.491	65	17.2	Chr5: 99.299507	0.078
35 ☐	MA_M2F_0706_R	1423651_at	*Isca1*	iron-sulfur cluster assembly 1 homolog; distal 3' UTR	Chr13: 21.496566	13.170	65	11.7	Chr9: 107.824821	0.079
36 ☐	MA_M2F_0706_R	1423711_at	*Ndufaf1*	NADH dehydrogenase (ubiquinone) 1 alpha subcomplex, assembly factor 1; last 3 exons and 3' UTR	Chr2: 119.655607	9.734	65	11.6	Chr14: 3.000000	0.134

37 ☐	MA_M2F_0706_R	1452211_at	*Psme4*	proteasome (prosome, macropain) activator subunit 4; 3'UTR	Chr11: 30.879764	12.074	65	22.3	Chr11: 29.493209	-0.167
38 ☐	MA_M2F_0706_R	1435966_x_at	*Mrpl13*	mitochondrial ribosomal protein L13; Proximal, mid 3' UTR, and intorn	Chr15: 55.534119	7.208	65	13.9	Chr18: 82.240604	-0.123
39 ☐	MA_M2F_0706_R	1424770_at	*Cald1*	caldesmon 1; exons 2 and 3 (last two of a short isoform)	Chr6: 34.745616	9.541	65	10.5	Chr10: 22.000000	-0.168
40 ☐	MA_M2F_0706_R	1450040_at	*Timp2*	tissue inhibitor of metalloproteinase 2; mid 3' UTR	Chr11: 118.302339	10.776	65	12.7	Chr4: 96.896949	-0.119
41 ☐	MA_M2F_0706_R	1417573_at	*Mmadhc*	methylmalonic aciduria (cobalamin deficiency) cblD type, with homocystinuria; last two exons and 3' UTR	Chr2: 50.279951	11.670	65	10.5	Chr3: 134.523036	-0.097
42 ☐	MA_M2F_0706_R	1434587_x_at	*Ptdss2*	phosphatidylserine synthase 2	Chr7: 141.155733	10.985	65	11.4	Chr4: 141.236455	-0.065
43 ☐	MA_M2F_0706_R	1415813_at	*Api5*	apoptosis inhibitor 5; distal 3' UTR (transQTL on Chr 4 in BXD eye data)	Chr2: 94.411933	10.893	65	10.9	Chr2: 102.660525	-0.093
44 ☐	MA_M2F_0706_R	1448492_a_at	*Psmd12*	proteasome (prosome, macropain) 26S subunit, non-ATPase, 12	Chr11: 107.495733	11.237	65	13.4	Chr9: 99.750326	0.076
45 ☐	MA_M2F_0706_R	1428490_at	*C1galt1*	core 1 synthase, glycoprotein-N-acetylgalactosamine 3-beta-galactosyltransferase	Chr6: 7.875102	10.287	65	12.3	Chr4: 96.896949	0.131
46 ☐	MA_M2F_0706_R	1424700_at	*Tmem38b*	transmembrane protein 38B (trimeric intracellular cation channel type B); last exon	Chr4: 53.859998	12.660	65	13.8	Chr16: 73.747956	0.124
47 ☐	MA_M2F_0706_R	1416838_at	*Mut*	methylmalonyl-Coenzyme A mutase; last three exons including proximal 3' UTR	Chr17: 40.958449	13.211	65	12.6	Chr17: 9.373070	0.106
48 ☐	MA_M2F_0706_R	1422471_at	*Pex13*	peroxisomal biogenesis factor 13	Chr11: 23.648970	11.367	65	10.6	Chr16: 63.773114	-0.111
49 ☐	MA_M2F_0706_R	1452086_at	*2610027O18Rik*	RIKEN cDNA 2610027O18 gene	Chr12: 73.280555	7.543	65	13.9	Chr9: 99.750326	0.052
50 ☐	MA_M2F_0706_R	1416096_at	*AI413782*	protein LOC63894; mid 3' UTR	Chr12: 87.239058	10.350	65	9.7	Chr9: 107.824821	0.081

51 ☐	MA_M2F_0706_R	1431423_a_at	*Med8*	mediator of RNA polymerase II transcription, subunit 8 homolog; last 2 exons and proximal half of 3' UTR	Chr4: 118.413625	10.483	65	11.9	Chr4: 102.851020	0.103
52 ☐	MA_M2F_0706_R	1426759_at	*Map4k3*	mitogen-activated protein kinase kinase kinase kinase 3; distal 3' UTR	Chr17: 80.580539	10.460	65	9.9	Chr1: 173.633577	0.115
53 ☐	MA_M2F_0706_R	1455007_s_at	*Gpt2*	glutamic pyruvate transaminase (alanine aminotransferase) 2; mid distal 3' UTR	Chr8: 85.526839	9.429	65	14.7	Chr14: 107.221502	0.223
54 ☐	MA_M2F_0706_R	1434037_s_at	*Kat2b*	K(lysine) acetyltransferase 2B (p300/CREB-binding protein-associated factor); distal 3' UTR	Chr17: 53.672145	11.464	65	14.0	Chr10: 19.937470	0.095
55 ☐	MA_M2F_0706_R	1416466_at	*Vapa*	vesicle-associated membrane protein, associated protein A; 3' UTR	Chr17: 65.580288	13.258	65	10.0	Chr15: 91.405127	0.082
56 ☐	MA_M2F_0706_R	1427911_at	*2610307O08Rik*	RIKEN cDNA 2610307O08 gene	Chr18: 35.734294	9.200	65	13.0	Chr16: 73.747956	0.087
57 ☐	MA_M2F_0706_R	1452662_a_at	*Eif2s1*	eukaryotic translation initiation factor 2, subunit 1 alpha; last two exons and proximal half of 3' UTR	Chr12: 78.884789	10.839	65	10.6	Chr16: 89.532874	-0.085
58 ☐	MA_M2F_0706_R	1455286_at	*Btbd1*	BTB (POZ) domain containing 1; distal 3' UTR	Chr7: 81.792103	12.326	65	10.4	Chr16: 88.032782	-0.085
59 ☐	MA_M2F_0706_R	1417316_at	*Them2*	thioesterase superfamily member 2	Chr13: 24.818001	13.114	65	13.5	ChrX: 24.000000	-0.080
60 ☐	MA_M2F_0706_R	1427362_x_at	*Hoxc6*	homeo box C6; 3' UTR	Chr15: 103.011286	9.316	65	16.7	Chr4: 78.900946	-0.124
61 ☐	MA_M2F_0706_R	1436919_at	*Tp53i11*	transformation related protein 53 inducible protein 11; distal 3' UTR	Chr2: 93.201169	9.062	65	12.0	Chr10: 17.820266	-0.120
62 ☐	MA_M2F_0706_R	1423781_at	*Appbp1*	amyloid beta precursor protein binding protein 1; exons 16, 18, 19, 20 and proximal 3' UTR	Chr8: 104.513201	10.361	65	15.9	Chr4: 96.896949	0.134
63 ☐	MA_M2F_0706_R	1422880_at	*Sypl*	synaptophysin-like protein; distal 3' UTR	Chr12: 32.976333	13.122	65	11.5	Chr4: 81.958676	0.117

64 ☐	MA_M2F_0706_R	1437143_a_at	*Txndc1*	thioredoxin domain containing 1; distal 3' UTR	Chr12: 70.467464	11.361	65	18.4	Chr15: 91.405127	0.153
65 ☐	MA_M2F_0706_R	1434447_at	*Met*	met proto-oncogene; distal 3' UTR	Chr6: 17.573398	11.796	65	12.1	Chr19: 14.967297	-0.124
66 ☐	MA_M2F_0706_R	1426256_at	*Timm17a*	translocator of inner mitochondrial membrane 17a	Chr1: 135.301708	12.121	65	14.7	Chr15: 89.576205	0.114
67 ☐	MA_M2F_0706_R	1415783_at	*Vps35*	vacuolar protein sorting 35; 3' UTR	Chr8: 85.260458	13.460	65	11.6	Chr16: 73.747956	-0.090
68 ☐	MA_M2F_0706_R	1422009_at	*Atp1b2*	ATPase, Na+/K+ transporting, beta 2 polypeptide	Chr11: 69.601309	7.800	65	13.5	Chr9: 99.750326	-0.108
69 ☐	MA_M2F_0706_R	1450243_a_at	*Dscr1l1*	Down syndrome critical region gene 1-like 1; last three exons	Chr17: 44.017779	6.708	65	15.1	Chr15: 91.405127	-0.089
70 ☐	MA_M2F_0706_R	1450376_at	*Mxi1*	Max interacting protein 1	Chr19: 53.372691	11.983	65	19.4	Chr9: 105.556898	0.126
71 ☐	MA_M2F_0706_R	1433846_s_at	*Fam175b*	abraxas brother 1, family with sequence similarity 175 member B; distal 3' UTR	Chr7: 132.884531	10.475	65	11.8	Chr10: 24.000000	0.082
72 ☐	MA_M2F_0706_R	1421164_a_at	*Arhgef1*	Rho guanine nucleotide exchange factor (GEF) 1	Chr7: 24.925880	10.459	65	11.7	Chr4: 81.726726	-0.094
73 ☐	MA_M2F_0706_R	1417936_at	*Ccl9*	chemokine (C-C motif) ligand 9	Chr11: 83.574911	7.362	65	11.7	Chr4: 150.657930	-0.142
74 ☐	MA_M2F_0706_R	1421185_at	*UbiE2*	RIKEN cDNA 3300001H21 gene	Chr15: 100.328217	7.776	65	13.4	Chr9: 94.904747	-0.095
75 ☐	MA_M2F_0706_R	1433463_at	*Brox*	BRO1 domain and CAAX motif containing; distal 3' UTR	Chr6: 112.608270	10.926	65	9.0	Chr13: 45.359287	0.080
76 ☐	MA_M2F_0706_R	1423073_at	*Cmpk*	cytidylate kinase; distal half of 3' UTR	Chr4: 114.960709	13.443	65	10.0	Chr2: 80.054211	-0.078
77 ☐	MA_M2F_0706_R	1437450_x_at	*C14orf166*	CLE7 homolog (UPF0568 protein)	Chr14: 19.811971	12.200	65	13.4	Chr16: 73.747956	-0.104
78 ☐	MA_M2F_0706_R	1436215_at	*Ipmk*	inositol polyphosphate multikinase; distal 3' UTR	Chr10: 71.385472	10.813	65	13.8	Chr15: 91.405127	0.140
79 ☐	MA_M2F_0706_R	1416596_at	*Slc44a4*	solute carrier family 44, member 4 (choline transporter-like); last five exons	Chr17: 34.928445	12.257	65	11.7	Chr9: 107.639250	0.119

80 ☐	MA_M2F_0706_R	1451719_at	*Crsp6*	cofactor required for Sp1 transcriptional activation, subunit 6	Chr9: 15.260462	6.667	65	15.3	Chr16: 17.412078	0.058
81 ☐	MA_M2F_0706_R	1429088_at	*Lbh*	limb bud and heart development (lupus brain antigen 1); distal 3' UTR	Chr17: 72.941355	9.300	65	10.8	Chr6: 44.030269	0.158
82 ☐	MA_M2F_0706_R	1435636_at	*Ago2*	argonaute RISC catalytic component 2 (eukaryotic translation initiation factor 2C, 2); distal 3' UTR	Chr15: 73.099051	9.907	65	14.1	Chr15: 89.576205	-0.123
83 ☐	MA_M2F_0706_R	1451247_at	*Mfsd1*	major facilitator superfamily domain containing 1; distal 3'UTR	Chr3: 67.603676	12.654	65	13.8	Chr15: 91.405127	0.103
84 ☐	MA_M2F_0706_R	1421104_at	*Mpa2*	macrophage activation 2	Chr10: 77.268946	7.246	65	10.7	Chr19: 23.135216	-0.054
85 ☐	MA_M2F_0706_R	1434038_at	*Dnajc13*	DnaJ (Hsp40) homolog, subfamily C, member 13	Chr9: 104.151791	11.426	65	16.1	Chr9: 99.750326	0.117
86 ☐	MA_M2F_0706_R	1416678_at	*Cops3*	COP9 (constitutive photomorphogenic) homolog, subunit 3; last four exons and 3' UTR	Chr11: 59.817915	11.640	65	9.5	Chr9: 107.639250	0.087
87 ☐	MA_M2F_0706_R	1436139_at	*Mdga2*	MAM domain containing glycosylphosphatidylinositol anchor 2; putative far 3' UTR element	Chr12: 66.466121	5.517	65	14.0	Chr6: 3.266392	0.023
88 ☐	MA_M2F_0706_R	1460745_at	*A630098A13Rik*	RIKEN cDNA A630098A13 gene	Chr14: 53.961232	6.670	65	10.2	Chr7: 89.123287	-0.039
89 ☐	MA_M2F_0706_R	1415966_a_at	*Ndufv1*	NADH dehydrogenase (ubiquinone) flavoprotein 1; last exon	Chr19: 4.007602	14.081	65	10.1	Chr16: 73.747956	-0.097
90 ☐	MA_M2F_0706_R	1416570_s_at	*Gfm1*	G elongation factor 1	Chr3: 67.473475	11.851	65	9.8	Chr3: 65.564009	-0.126
91 ☐	MA_M2F_0706_R	1421374_a_at	*Fxyd1*	FXYD domain-containing ion transport regulator 1; last 6 exons except last exon and proximal 3' UTR	Chr7: 31.053025	11.031	65	16.0	Chr16: 89.532874	0.200
92 ☐	MA_M2F_0706_R	1455626_at	*Hoxa9*	homeo box A9	Chr6: 52.223121	11.691	65	11.5	Chr15: 91.405127	0.120

93	MA_M2F_0706_R	1438647_x_at	*Cetn2*	centrin 2	ChrX: 72.913746	10.870	65	17.4	Chr16: 73.747956	-0.167
94	MA_M2F_0706_R	1452812_at	*Lphn1*	latrophilin 1	Chr8: 83.939489	8.548	65	16.5	Chr4: 86.450967	-0.133
95	MA_M2F_0706_R	1422442_at	*Smu1*	smu-1 suppressor of mec-8 and unc-52 homolog (C. elegans); 3 exons and 3'UTR	Chr4: 40.737149	12.387	65	10.6	Chr4: 119.382248	0.067
96	MA_M2F_0706_R	1424274_at	*Uso1*	USO1 homolog, vesicle docking protein (vesicle docking protein); mid and mid-distal 3' UTR	Chr5: 92.202219	11.228	65	11.9	Chr8: 24.838961	-0.144
97	MA_M2F_0706_R	1434665_at	*Aga*	aspartylglucosaminidase; exons 6, 7, and 8	Chr8: 53.521136	10.853	65	11.1	Chr9: 88.498812	0.110
98	MA_M2F_0706_R	1416923_a_at	*Bnip3l*	BCL2/adenovirus E1B 19kDa-interacting protein 3-like	Chr14: 66.985274	11.039	65	13.2	Chr10: 19.937470	0.133
99	MA_M2F_0706_R	1424148_a_at	*Stap2*	signal transducing adaptor family member 2; exon	Chr17: 55.997780	10.110	65	12.2	Chr16: 73.747956	0.210
100	MA_M2F_0706_R	1426668_at	*Slc30a9*	solute carrier family 30 (zinc transporter), member 9; distal half of 3' UTR	Chr5: 67.355241	11.648	65	16.1	Chr10: 17.820266	0.153
101	MA_M2F_0706_R	1420847_a_at	*Fgfr2*	Fgfr2 fibroblast growth factor receptor 2; last 3 exons and 3' UTR	Chr7: 130.163853	7.734	65	14.7	Chr15: 91.405127	-0.111
102	MA_M2F_0706_R	1419426_s_at	*Ccl21*	CCL complex region (chemokine (C-C motif) ligand 21, Ccl19, Ccl27, Il11ra2) probe set, RIKEN cDNA 4933409K07 gene; last two exons and 3' UTR (copy number variation in mice, segmental duplication)	Chr4: 41.904067	10.263	65	12.3	Chr16: 69.651524	0.181
103	MA_M2F_0706_R	1453269_at	*Unc5b*	unc-5 homolog B (C. elegans)	Chr10: 60.764623	7.274	65	11.2	Chr19: 3.337223	-0.051
104	MA_M2F_0706_R	1435965_at	*Cnot3*	CCR4-NOT transcription complex, subunit 3; last exon and 3' UTR	Chr3: 96.454606	10.641	65	8.5	Chr16: 85.961783	0.129
105	MA_M2F_0706_R	1448856_a_at	*Msra*	methionine sulfoxide reductase A; 3' UTR	Chr14: 64.122780	13.960	65	10.6	Chr1: 46.908195	0.091

106 ☐	MA_M2F_0706_R	1439029_at	*Gpt2*	glutamic pyruvate transaminase (alanine aminotransferase) 2	Chr8: 85.523253	9.944	65	14.3	Chr19: 23.853320	0.212
107 ☐	MA_M2F_0706_R	1450721_at	*Acp1*	acid phosphatase 1, soluble; proximal 3' UTR	Chr12: 30.895096	11.825	65	19.7	Chr15: 91.405127	0.118
108 ☐	MA_M2F_0706_R	1452466_a_at	*Rbm6*	RNA binding motif protein 6; sixth, fifth, and fourth to last exons	Chr9: 107.779589	9.800	65	14.1	Chr16: 63.773114	0.105
109 ☐	MA_M2F_0706_R	1460295_s_at	*Il6st*	interleukin 6 signal transducer; proximal to mid 3' UTR	Chr13: 112.505179	9.448	65	11.2	Chr15: 91.405127	-0.173
110 ☐	MA_M2F_0706_R	1417512_at	*Evi5*	ecotropic viral integration site 5	Chr5: 107.744913	10.948	65	11.6	Chr3: 39.650830	-0.142
111 ☐	MA_M2F_0706_R	1450159_at	*Rem1*	rad and gem related GTP binding protein 1	Chr2: 152.634893	6.891	65	15.6	Chr2: 107.153805	0.067
112 ☐	MA_M2F_0706_R	1421836_at	*Mtap7*	microtubule-associated protein 7 (male sterility and histoincompatibility); distal 3' UTR	Chr10: 20.280929	12.783	65	80.3	Chr10: 19.937470	0.455
113 ☐	MA_M2F_0706_R	1458268_s_at	*Igfbp3*	insulin-like growth factor binding protein 3	Chr11: 7.206603	12.214	65	15.7	Chr15: 89.576205	-0.234
114 ☐	MA_M2F_0706_R	1417258_at	*Cct5*	chaperonin subunit 5 (epsilon); exons 8, 9, 10	Chr15: 31.592291	12.880	65	10.4	Chr15: 93.454108	0.089
115 ☐	MA_M2F_0706_R	1428594_at	*Garnl1*	GTPase activating RANGAP domain-like 1; distal 3' UTR	Chr12: 55.603286	10.125	65	6.9	Chr10: 9.609272	0.081
116 ☐	MA_M2F_0706_R	1433527_at	*Ireb2*	iron responsive element binding protein 2; distal 3'UTR	Chr9: 54.911952	10.221	65	11.4	Chr2: 149.665875	-0.123
117 ☐	MA_M2F_0706_R	1460429_at	*Cdc5l*	cell division cycle 5-like; last 2 exons and 3' UTR	Chr17: 45.392040	10.655	65	12.8	Chr16: 63.773114	-0.111
118 ☐	MA_M2F_0706_R	1448809_at	*Cse1l*	chromosome segregation 1-like; last 2 exons and 3' UTR	Chr2: 166.945703	10.313	65	12.0	Chr15: 91.405127	0.119
119 ☐	MA_M2F_0706_R	1433535_x_at	*Cct2*	chaperonin subunit 2 (beta)	Chr10: 103.893749	12.756	65	16.1	Chr16: 63.773114	-0.107
120 ☐	MA_M2F_0706_R	1449935_a_at	*Dnaja3*	DnaJ (Hsp40) homolog, subfamily A, member 3; mid-distal 3' UTR	Chr16: 4.707167	11.562	65	14.7	Chr4: 155.225671	0.088
121 ☐	MA_M2F_0706_R	1416270_at	*Polr2g*	polymerase (RNA) II (DNA directed)	Chr19: 8.793198	11.581	65	10.6	Chr16: 89.532874	-0.080

				polypeptide G; exons 3, 4, 5, 7, and 3' UTR						
122 ☐	MA_M2F_0706_R	1460688_s_at	*AA407659*	expressed sequence AA407659	Chr5: 115.122729	11.914	65	14.9	Chr4: 78.900946	0.139
123 ☐	MA_M2F_0706_R	1423486_at	*Cript*	postsynaptic protein CRIPT (cysteine-rich PDZ-binding protein); last several exons and 3' UTR	Chr17: 87.034276	12.203	65	10.2	Chr15: 87.476581	0.079
124 ☐	MA_M2F_0706_R	1423746_at	*Txndc5*	thioredoxin domain containing 5	Chr13: 38.501121	11.683	65	20.1	Chr9: 99.750326	-0.160
125 ☐	MA_M2F_0706_R	1450699_at	*Selenbp1*	selenium binding protein 1	Chr3: 94.944489	14.224	65	21.8	Chr9: 99.750326	0.113
126 ☐	MA_M2F_0706_R	1426529_a_at	*Tagln2*	transgelin 2; mid and distal 3' UTR	Chr1: 172.506826	12.074	65	22.4	Chr1: 172.235364	-0.174
127 ☐	MA_M2F_0706_R	1415678_at	*Ppm1a*	protein phosphatase, Mg2+/Mn2+ dependent 1A; 3' UTR	Chr12: 72.793964	11.854	65	14.1	Chr4: 78.900946	0.105
128 ☐	MA_M2F_0706_R	1449418_s_at	*Fbxo36*	DNA segment, Chr 1, ERATO Doi 757, expressed	Chr1: 84.896587	8.758	65	14.4	Chr16: 63.773114	-0.107
129 ☐	MA_M2F_0706_R	1451369_at	*Commd5*	COMM domain containing 5; middle of only exon	Chr15: 76.900714	11.258	65	15.0	Chr9: 88.498812	-0.179
130 ☐	MA_M2F_0706_R	1434132_at	*Spg8*	spastic paraplegia 8 (strumpellin); last three exons and proximal 3' UTR	Chr15: 59.332020	11.795	65	7.7	Chr5: 117.810543	0.071
131 ☐	MA_M2F_0706_R	1432717_at	*Glcci1*	glucocorticoid induced transcript 1	Chr6: 8.415255	6.598	65	7.7	Chr16: 63.773114	-0.036
132 ☐	MA_M2F_0706_R	1460131_at	*2810040C05Rik*	RIKEN cDNA 2810040C05 gene	Chr8: 26.131805	6.936	65	12.6	Chr9: 99.750326	-0.048
133 ☐	MA_M2F_0706_R	1456748_a_at	*Nipsnap1*	4-nitrophenylphosphatase domain and non-neuronal SNAP25-like protein homolog 1; distal 3' UTR	Chr11: 4.894050	11.565	65	11.3	Chr9: 83.778825	0.146
134 ☐	MA_M2F_0706_R	1420776_a_at	*Auh*	AU RNA binding protein/enoyl-coenzyme A hydratase (mitochondrial, 3-methylglutaconic aciduria, type I); last four exons and proximal half of 3' UTR	Chr13: 52.835320	12.010	65	16.4	Chr13: 51.479103	0.104

135 ☐	MA_M2F_0706_R	1415775_at	*Rbbp7*	retinoblastoma binding protein 7; last two exons and 3' UTR	ChrX: 162.777771	12.328	65	10.2	Chr3: 134.523036	-0.084
136 ☐	MA_M2F_0706_R	1448654_at	*Mtch2*	mitochondrial carrier homolog 2; distal 3' UTR	Chr2: 90.866101	10.282	65	15.4	Chr4: 102.851020	0.190
137 ☐	MA_M2F_0706_R	1448860_at	*Rem2*	rad and gem related GTP binding protein 2; 3' UTR	Chr14: 54.479912	8.149	65	11.2	Chr15: 93.454108	-0.074
138 ☐	MA_M2F_0706_R	1455604_at	*AI427138*	expressed sequence AI427138; distal 3' UTR	Chr1: 64.730740	8.702	65	8.7	Chr14: 48.527624	0.115
139 ☐	MA_M2F_0706_R	1452093_at	*Tmem185b*	transmembrane protein 185B; 3' UTR	Chr1: 119.527628	9.697	65	15.1	Chr14: 3.000000	0.125
140 ☐	MA_M2F_0706_R	1415901_at	*Plod3*	procollagen-lysine, 2-oxoglutarate 5-dioxygenase 3 (RER protein critical for stability of crosslinks. Ehlers-Danlos syndrome type VIB have deficiencies in lysyl hydroxylase); last two exons and proximal 3' UTR	Chr5: 136.994931	10.958	65	18.5	Chr5: 135.879195	0.095
141 ☐	MA_M2F_0706_R	1421118_a_at	*Gpr56*	G protein-coupled receptor 56; 3' UTR	Chr8: 95.013029	11.029	65	11.5	Chr19: 15.292517	0.172
142 ☐	MA_M2F_0706_R	1425143_a_at	*Ndufs1*	NADH dehydrogenase (ubiquinone) Fe-S protein 1; last 3 exons and proximal 3' UTR	Chr1: 63.143795	13.516	65	15.0	Chr14: 107.221502	-0.084
143 ☐	MA_M2F_0706_R	1425554_a_at	*Cdc16*	CDC16 cell division cycle 16 homolog (S. cerevisiae); 2 exons and 3'UTR	Chr8: 13.779240	11.253	65	10.9	Chr15: 91.405127	0.083
144 ☐	MA_M2F_0706_R	1424427_at	*Tada1l*	transcriptional adaptor 1 (HFI1 homolog, yeast) like (SPT3-associated factor 42); mid and distal 3' UTR	Chr1: 166.393042	8.625	65	11.7	Chr2: 148.055322	-0.109
145 ☐	MA_M2F_0706_R	1428662_a_at	*Hopx*	HOP homeobox; mid and mid distal 3' UTR	Chr5: 77.087372	11.255	65	11.8	Chr9: 88.498812	-0.119
146 ☐	MA_M2F_0706_R	1455925_at	*Prdm8*	PR domain containing 8; mid 3' UTR	Chr5: 98.186885	5.803	65	10.2	Chr14: 55.622689	0.025
147 ☐	MA_M2F_0706_R	1431109_at	*5430406M13Rik*	RIKEN cDNA 5430406M13 gene	Chr18: 51.303995	6.610	65	10.3	Chr4: 96.896949	-0.062

148	MA_M2F_0706_R	1429278_at	*2410170E07Rik*	RIKEN cDNA 2410170E07 gene	Chr7: 140.992613	10.331	65	18.5	Chr9: 99.750326	0.141
149	MA_M2F_0706_R	1428955_x_at	*Slc9a3r2*	solute carrier family 9 (sodium/hydrogen exchanger), isoform 3 regulator 2	Chr17: 24.639295	9.942	65	13.5	Chr9: 107.639250	-0.113
150	MA_M2F_0706_R	1454159_a_at	*Igfbp2*	insulin-like growth factor binding protein 2, 36kDa (trabecular meshwork); last two exons and 3' UTR	Chr1: 72.849669	7.762	65	9.2	Chr14: 55.622689	-0.269
151	MA_M2F_0706_R	1423197_a_at	*Smek2*	SMEK homolog 2 suppressor of mek1	Chr11: 29.220542	9.139	65	9.0	Chr14: 3.000000	0.114
152	MA_M2F_0706_R	1448610_a_at	*Sod2*	superoxide dismutase 2, mitochondrial; last exon and proximal 3' UTR	Chr17: 13.015161	13.375	65	17.9	Chr15: 89.576205	0.140
153	MA_M2F_0706_R	1453104_at	*Mapk1*	mitogen activated protein kinase 1 (ERK2); distal 3' UTR	Chr16: 17.046931	11.216	65	10.7	Chr15: 91.405127	0.102
154	MA_M2F_0706_R	1430500_s_at	*Mtx2*	metaxin 2; 3 exons	Chr2: 74.869348	11.466	65	15.1	Chr4: 96.896949	0.137
155	MA_M2F_0706_R	1448434_at	*Rnf103*	ring finger protein 103; last exon and proximal 3' UTR	Chr6: 71.510307	10.664	65	9.0	Chr8: 24.838961	-0.129
156	MA_M2F_0706_R	1423044_at	*Prosc*	proline synthetase co-transcribed	Chr8: 27.054003	12.857	65	13.7	Chr9: 99.750326	0.096
157	MA_M2F_0706_R	1420846_at	*Mrps2*	mitochondrial ribosomal protein S2 (28S subunit); distal half of 3' UTR	Chr2: 28.470640	10.632	65	13.5	Chr4: 102.851020	0.094
158	MA_M2F_0706_R	1416859_at	*Fkbp3*	FK506 binding protein 3; last four exons	Chr12: 65.063678	10.787	65	16.2	Chr3: 134.523036	-0.122
159	MA_M2F_0706_R	1457677_at	*C920016K16Rik*	RIKEN cDNA C920016K16; distal 3' UTR	Chr17: 33.004869	8.038	65	12.4	Chr1: 191.333382	-0.061
160	MA_M2F_0706_R	1439411_a_at	*Xpo7*	exportin 7; 3' UTR	Chr14: 70.664564	8.787	65	13.8	Chr15: 91.405127	0.211
161	MA_M2F_0706_R	1460341_at	*Plekhb2*	pleckstrin homology domain containing, family B (evectins) member 2; distal 3' UTR	Chr1: 34.879012	13.239	65	16.4	Chr9: 107.824821	0.176
162	MA_M2F_0706_R	1456530_x_at	*Elovl1*	elongation of very long chain fatty acids (FEN1/Elo2, SUR4/Elo3)-like 1; mid 3' UTR	Chr4: 118.432469	11.661	65	16.3	Chr15: 89.576205	0.162

163	MA_M2F_0706_R	1450897_at	*Arhgap5*	Rho GTPase activating protein 5	Chr12: 52.568389	11.311	65	10.0	Chr16: 66.730522	-0.121
164	MA_M2F_0706_R	1417243_at	*Nip30*	hypothetical protein LOC80011; 3' UTR	Chr8: 94.575017	10.372	65	16.2	Chr9: 88.498812	0.084
165	MA_M2F_0706_R	1451522_s_at	*Lrch4*	leucine-rich repeats and calponin homology (CH) domain containing 4; last exon and 3' UTR	Chr5: 137.640054	11.046	65	15.3	Chr1: 163.338902	-0.111
166	MA_M2F_0706_R	1423346_at	*Degs1*	degenerative spermatocyte homolog 1; mid to distal 3' UTR	Chr1: 182.276000	12.383	65	15.5	Chr15: 91.405127	0.132
167	MA_M2F_0706_R	1430129_a_at	*Commd8*	COMM domain containing 8; proximal 3' UTR	Chr5: 72.159618	10.280	65	11.0	Chr4: 5.599068	-0.096
168	MA_M2F_0706_R	1419352_at	*C11orf73*	human chromosome 11 open reading frame 73; exons 2, 3, and 4	Chr7: 89.920136	10.503	65	18.7	Chr7: 84.149847	0.144
169	MA_M2F_0706_R	1455988_a_at	*Cct6a*	chaperonin subunit 6a (zeta); 3' UTR	Chr5: 129.845865	13.793	65	13.6	Chr16: 74.917702	-0.071
170	MA_M2F_0706_R	1420711_a_at	*Pxmp3*	peroxisomal membrane protein 3; only exon and proximal 3' UTR	Chr3: 5.560691	10.867	65	18.5	Chr4: 96.896949	0.157
171	MA_M2F_0706_R	1428328_at	*Nup50*	nucleoporin 50; distal 3' UTR	Chr15: 84.942471	9.865	65	14.2	Chr15: 91.405127	0.116
172	MA_M2F_0706_R	1418000_a_at	*Itm2b*	integral membrane protein 2B; last four exons	Chr14: 73.365788	16.711	65	13.5	Chr14: 47.060301	-0.104
173	MA_M2F_0706_R	1452172_at	*2810421I24Rik*	RIKEN cDNA 2810421I24 gene	Chr1: 63.748029	9.287	65	12.6	Chr4: 96.896949	0.161
174	MA_M2F_0706_R	1450112_a_at	*Gas2*	growth arrest specific 2	Chr7: 51.993933	13.345	65	17.0	Chr16: 74.899626	-0.136
175	MA_M2F_0706_R	1415915_at	*Ddx1*	DEAD (Asp-Glu-Ala-Asp) box polypeptide 1; last 4 exons and 3' UTR	Chr12: 13.219383	11.091	65	10.4	Chr4: 121.044402	0.098
176	MA_M2F_0706_R	1418929_at	*Ift57*	intraflagellar transport 57 (estrogen-related receptor beta like 1); 4 exons and 3'UTR	Chr16: 49.763742	9.323	65	15.2	Chr6: 42.000000	-0.118
177	MA_M2F_0706_R	1452203_at	*Obfc2a*	oligonucleotide/oligosaccharide-binding fold; distal 3' UTR	Chr1: 51.469698	10.797	65	17.3	Chr10: 116.134021	-0.167

178 ☐	MA_M2F_0706_R	1438425_at	*Gtf3c1*	general transcription factor III C 1	Chr7: 125.670486	7.085	65	11.0	Chr15: 91.892917	-0.110
179 ☐	MA_M2F_0706_R	1424013_at	*Etf1*	eukaryotic translation termination factor 1; far 3' UTR	Chr18: 34.902978	10.823	65	8.6	Chr3: 134.523036	-0.086
180 ☐	MA_M2F_0706_R	1443754_x_at	*Lsamp*	limbic system-associated membrane protein; intron 1	Chr16: 41.667831	6.130	65	17.0	Chr4: 78.900946	-0.094
181 ☐	MA_M2F_0706_R	1421751_a_at	*Psmd14*	proteasome (prosome, macropain) 26S subunit, non-ATPase, 14; 2 exons and 3'UTR	Chr2: 61.799980	12.178	65	12.0	Chr15: 91.892917	0.121
182 ☐	MA_M2F_0706_R	1423505_at	*Tagln*	transgelin; last two exons and 3' UTR	Chr9: 45.930244	10.635	65	10.8	Chr4: 75.824944	-0.167
183 ☐	MA_M2F_0706_R	1424681_a_at	*Psma5*	proteasome (prosome, macropain) subunit, alpha type 5; putative exon (transQTL on Chr 4 in BXD eye data)	Chr3: 108.267833	11.233	65	11.1	Chr8: 85.923280	-0.069
184 ☐	MA_M2F_0706_R	1417018_at	*Efemp2*	epidermal growth factor-containing fibulin-like extracellular matrix protein 2	Chr19: 5.480267	9.230	65	16.1	Chr4: 78.900946	-0.151
185 ☐	MA_M2F_0706_R	1452153_at	*Fbxo18*	F-box protein 18	Chr2: 11.742673	10.259	65	12.2	Chr15: 89.576205	0.082
186 ☐	MA_M2F_0706_R	1423773_at	*Gpbp1*	GC-rich promoter binding protein 1 (vascular wall-linked protein); last two exons and 3' UTR	Chr13: 111.426379	11.002	65	15.0	Chr9: 99.750326	0.118
187 ☐	MA_M2F_0706_R	1434749_at	*BC067068*	cDNA sequence BC067068	Chr10: 105.763310	7.239	65	12.9	Chr4: 78.900946	0.042
188 ☐	MA_M2F_0706_R	1424283_at	*Jtb*	jumping translocation breakpoint; exon 3, 4, 5,and 3' UTR	Chr3: 90.235381	12.158	65	16.1	Chr14: 49.074404	0.125
189 ☐	MA_M2F_0706_R	1436665_a_at	*Ltbp4*	latent transforming growth factor beta binding protein 4; exons 31 and 32	Chr7: 27.306091	10.249	65	12.7	Chr16: 74.899626	0.215
190 ☐	MA_M2F_0706_R	1443830_x_at	*Rnf103*	ring finger protein 103; distal 3' UTR	Chr6: 71.510731	8.727	65	14.1	Chr16: 85.961783	-0.125
191 ☐	MA_M2F_0706_R	1417594_at	*Gkap1*	G kinase anchoring protein 1; last 5 exons and 3' UTR	Chr13: 58.233391	10.894	65	14.7	Chr9: 99.750326	0.124
192 ☐	MA_M2F_0706_R	1427605_at	*Hoxb3*	homeo box B3	Chr11: 96.346583	8.983	65	16.1	Chr4: 86.450967	-0.135

193	MA_M2F_0706_R	1449135_at	*Sox18*	SRY-box containing gene 18	Chr2: 181.670065	9.162	65	12.3	Chr16: 88.032782	0.104
194	MA_M2F_0706_R	1420822_s_at	*Sgpp1*	sphingosine-1-phosphate phosphatase 1; distal 3' UTR	Chr12: 75.714301	11.720	65	13.1	Chr4: 155.493057	0.135
195	MA_M2F_0706_R	1423517_at	*Cct6a*	chaperonin subunit 6a (zeta)	Chr5: 129.845633	14.113	65	13.1	Chr14: 107.221502	-0.067
196	MA_M2F_0706_R	1419970_at	*Slc35a5*	solute carrier family 35, member A5 (probable UDP-sugar transporter protein); mid 3' UTR	Chr16: 45.141755	7.730	65	10.1	Chr9: 105.774381	-0.119
197	MA_M2F_0706_R	1451547_at	*Iyd*	iodotyrosine deiodinase; 3' UTR	Chr10: 3.554323	11.225	65	14.9	Chr14: 49.789692	0.166
198	MA_M2F_0706_R	1438843_x_at	*Mtch2*	mitochondrial carrier homolog 2	Chr2: 90.861358	11.614	65	9.9	Chr1: 191.333382	-0.066
199	MA_M2F_0706_R	1448346_at	*Cfl1*	cofilin 1 (non-muscle, 18 kDa phosphoprotein); last exon and proximal 3' UTR	Chr19: 5.493442	12.760	65	14.2	Chr6: 42.000000	0.213
200	MA_M2F_0706_R	1415916_a_at	*Mthfd1*	methylenetetrahydrofolate dehydrogenase (NADP+ dependent), methenyltetrahydrofolate cyclohydrolase, formyltetrahydrofolate synthase; last exon and 3' UTR	Chr12: 76.317661	13.147	65	15.7	Chr3: 129.482611	-0.155
201	MA_M2F_0706_R	1452138_a_at	*Ace2*	angiotensin I converting enzyme (peptidyl-dipeptidase A) 2; last 2~6 and 11 exons	ChrX: 164.167855	10.033	65	12.1	Chr14: 3.000000	0.156
202	MA_M2F_0706_R	1419984_s_at	*Zfp644*	zinc finger protein 644; mid to distal 3' UTR	Chr5: 106.617110	9.828	65	12.3	Chr11: 83.064807	0.111
203	MA_M2F_0706_R	1424324_at	*A930014I12Rik*	RIKEN cDNA A930014I12 gene	Chr18: 10.566702	9.520	65	17.3	Chr11: 83.064807	0.187
204	MA_M2F_0706_R	1434682_at	*Zfp770*	zinc finger protein 770; distal 3' UTR	Chr2: 114.193761	7.485	65	16.6	Chr11: 83.064807	0.106
205	MA_M2F_0706_R	1416653_at	*Stxbp3a*	syntaxin binding protein 3a; proximal 3' UTR	Chr3: 108.793540	9.536	65	8.2	Chr8: 80.868085	-0.089
206	MA_M2F_0706_R	1434607_at	*Ddx52*	DEAD (Asp-Glu-Ala-Asp) box polypeptide 52	Chr11: 83.959487	7.083	65	13.2	Chr14: 9.528965	0.187

207 ☐	MA_M2F_0706_R	1425495_at	*Zfp62*	zinc finger protein 62	Chr11: 49.218192	6.712	65	18.6	Chr14: 3.000000	0.193
208 ☐	MA_M2F_0706_R	1434099_at	*Ppargc1a*	peroxisome proliferative activated receptor, gamma, coactivator 1 alpha; far 3' UTR	Chr5: 51.454320	9.157	65	11.1	Chr1: 191.601182	-0.140
209 ☐	MA_M2F_0706_R	1460258_at	*Lect1*	leukocyte cell derived chemotaxin 1	Chr14: 79.637747	6.847	65	14.1	Chr16: 3.500000	0.090
210 ☐	MA_M2F_0706_R	1426473_at	*Dnajc9*	DnaJ (Hsp40) homolog, subfamily C, member 9	Chr14: 20.385026	8.961	65	10.9	Chr15: 68.818097	-0.094
211 ☐	MA_M2F_0706_R	1417122_at	*Vav3*	vav 3 oncogene (Rho family guanine nucleotide exchange factor); mid distal 3' UTR	Chr3: 109.685110	8.002	65	9.6	Chr7: 84.149847	0.156
212 ☐	MA_M2F_0706_R	1452239_at	*Gt(ROSA)26Sor*	gene trap ROSA 26, Philippe Soriano (between Thumpd3 and Setd5); putative 3' end exon or 3' UTR, expressed even in normal mice	Chr6: 113.070606	7.082	65	13.9	Chr11: 83.064807	0.193
213 ☐	MA_M2F_0706_R	1449576_at	*Eif1ay*	eukaryotic translation initiation factor 1A, Y-linked; distal 3' UTR	ChrX: 159.387418	8.489	65	12.8	Chr14: 3.000000	0.125
214 ☐	MA_M2F_0706_R	1452785_at	*C18orf55*	human chromosome 18 open reading frame 55; last exons and 3' UTR (test Mendelian 10.86 in BXD)	Chr18: 84.947626	9.571	65	14.0	Chr14: 3.000000	0.132
215 ☐	MA_M2F_0706_R	1452676_a_at	*Pnpt1*	polyribonucleotide nucleotidyltransferase 1; last four exons including proximal 3' UTR	Chr11: 29.159348	9.388	65	15.7	Chr11: 24.905498	-0.186
216 ☐	MA_M2F_0706_R	1449960_at	*Nptx2*	neuronal pentraxin 2; last exon and 3' UTR	Chr5: 144.556182	6.458	65	8.8	Chr14: 19.755208	0.057
217 ☐	MA_M2F_0706_R	1424642_at	*Thoc1*	THO complex 1	Chr18: 9.992178	9.675	65	14.7	Chr2: 181.014276	0.121
218 ☐	MA_M2F_0706_R	1454826_at	*Zbtb11*	zinc finger and BTB domain containing 11; 3' UTR	Chr16: 56.008394	9.137	65	13.8	Chr11: 83.064807	0.230
219 ☐	MA_M2F_0706_R	1419641_at	*Purb*	purine rich element binding protein B	Chr11: 6.473439	9.152	65	16.5	Chr14: 3.000000	0.191
220 ☐	MA_M2F_0706_R	1428551_at	*Trmt11*	tRNA methyltransferase 11	Chr10: 30.534740	7.833	65	12.8	Chr11: 83.064807	0.116

				homolog; last 2 exons and 3' UTR						
221	MA_M2F_0706_R	1435947_at	*Stmn4*	stathmin-like 4	Chr19: 23.728467	8.262	65	17.2	Chr14: 9.528965	0.238
222	MA_M2F_0706_R	1419089_at	*Timp3*	tissue inhibitor of metalloproteinase 3	Chr10: 86.347860	14.442	65	15.7	Chr14: 19.755208	-0.149
223	MA_M2F_0706_R	1460430_at	*Rap2c*	RAP2C, member of RAS oncogene family	ChrX: 51.004526	8.210	65	13.6	Chr14: 3.000000	0.183
224	MA_M2F_0706_R	1454917_at	*AU045404*	expressed sequence AU045404	Chr15: 90.232891	8.797	65	14.2	Chr14: 9.528965	0.130
225	MA_M2F_0706_R	1434719_at	*A2m*	alpha-2-macroglobulin; last 7 exons except last exon	Chr6: 121.674940	6.859	65	7.9	Chr14: 19.755208	-0.077
226	MA_M2F_0706_R	1428829_at	*Dennd1b*	DENN/MADD domain containing 1B; putative far 3' UTR	Chr1: 139.175456	9.165	65	13.4	Chr14: 9.528965	0.186
227	MA_M2F_0706_R	1418817_at	*Chmp1b*	chromatin modifying protein 1B	Chr18: 67.207239	10.045	65	14.9	Chr14: 27.000000	0.205
228	MA_M2F_0706_R	1418222_at	*Noa1*	nitric oxide associated 1; 4 exons	Chr5: 77.294479	8.948	65	11.1	Chr7: 66.458029	0.136
229	MA_M2F_0706_R	1417438_at	*Rdh14*	retinol dehydrogenase 14 (all-trans and 9-cis)	Chr12: 10.395066	10.554	65	9.9	ChrX: 64.161107	-0.119
230	MA_M2F_0706_R	1446349_at	*Zfp78*	zinc finger protein 78; extreme distal 3' UTR	Chr7: 6.380471	7.232	65	14.5	Chr8: 24.382366	0.079
231	MA_M2F_0706_R	1435828_at	*Maf*	musculoaponeurotic fibrosarcoma (v-maf) AS42 transcription factor proto-oncogene; distal 3' UTR	Chr8: 115.683054	8.048	65	9.9	Chr14: 19.755208	0.155
232	MA_M2F_0706_R	1460168_at	*Slbp*	stem-loop binding protein	Chr5: 143.099261	9.691	65	12.8	ChrX: 70.094108	-0.158
233	MA_M2F_0706_R	1421968_a_at	*Nipa2*	non imprinted in Prader-Willi/Angelman syndrome 2 homolog (human)	Chr7: 55.932659	8.271	65	14.7	Chr14: 3.000000	0.220
234	MA_M2F_0706_R	1452090_a_at	*Olfm3*	olfactomedin 3; distal 3' UTR	Chr3: 115.124528	7.783	65	11.2	Chr14: 30.957748	0.234
235	MA_M2F_0706_R	1418640_at	*Sirt1*	sirtuin 1, NAD+ dependent histone deacetylase, class 1 (longevity, caloric restriction associated,	Chr10: 63.319162	8.837	65	12.8	Chr14: 3.000000	0.227

				mitochondrial biogenesis, cell survival); distal 3' UTR						
236 ☐	MA_M2F_0706_R	1419462_s_at	*Gtl3*	gene trap locus 3	Chr8: 95.420551	8.746	65	14.9	Chr8: 94.374289	-0.212
237 ☐	MA_M2F_0706_R	1448896_at	*Pigf*	phosphatidylinositol glycan, class F	Chr17: 86.997281	9.622	65	13.3	Chr11: 83.064807	0.156
238 ☐	MA_M2F_0706_R	1439334_at	*A330009G12*	ESTs	ChrX: 141.727603	5.533	65	10.8	Chr14: 9.528965	0.036
239 ☐	MA_M2F_0706_R	1435893_at	*Vldlr*	very low density lipoprotein receptor; putative far 3' UTR	Chr19: 27.253653	9.829	65	15.9	Chr11: 83.064807	0.183
240 ☐	MA_M2F_0706_R	1429121_at	*4921517N04Rik*	RIKEN cDNA 4921517N04 gene	Chr2: 23.510873	6.403	65	13.8	Chr11: 83.064807	0.126
241 ☐	MA_M2F_0706_R	1448555_at	*D15Ertd682e*	DNA segment, Chr 15, ERATO Doi 682, expressed	Chr15: 97.678396	8.509	65	12.8	Chr14: 9.528965	0.122
242 ☐	MA_M2F_0706_R	1429351_at	*Klhl24*	kelch-like 24; mid 3' UTR	Chr16: 20.124883	10.148	65	9.3	Chr13: 115.551768	-0.190
243 ☐	MA_M2F_0706_R	1455973_at	*BB738659*	ESTs	Chr11: 9.068785	6.793	65	13.0	Chr11: 9.165457	-0.252
244 ☐	MA_M2F_0706_R	1459712_at	*Zfp182*	zinc finger protein 182	ChrX: 21.026222	5.703	65	11.3	Chr14: 3.000000	0.048
245 ☐	MA_M2F_0706_R	1440742_at	*LOC239447*	ESTs	Chr18: 6.491461	6.615	65	14.6	Chr14: 19.755208	0.103
246 ☐	MA_M2F_0706_R	1428652_at	*0610010F05Rik*	RIKEN cDNA 0610010F05; distal 3' UTR	Chr11: 23.573924	8.242	65	14.3	Chr14: 9.528965	0.189
247 ☐	MA_M2F_0706_R	1434404_at	*Fam73a*	family with sequence similarity 73, member A	Chr3: 152.274018	7.884	65	13.8	Chr14: 3.000000	0.145
248 ☐	MA_M2F_0706_R	1454740_at	*Mib1*	mindbomb homolog 1; possible far 3' UTR	Chr18: 10.817206	9.656	65	12.4	Chr14: 3.000000	0.238
249 ☐	MA_M2F_0706_R	1448922_at	*Dusp19*	dual specificity phosphatase 19	Chr2: 80.621210	7.162	65	15.1	Chr14: 24.000000	0.153
250 ☐	MA_M2F_0706_R	1434628_a_at	*Rhpn2*	rhophilin, Rho GTPase binding protein 2	Chr7: 35.391927	6.973	65	9.8	Chr14: 3.000000	0.174
251 ☐	MA_M2F_0706_R	1435769_at	*Akap9*	A kinase (PRKA) anchor protein (yotiao) 9; last 2 exons and 3' UTR	Chr5: 4.077940	9.524	65	10.0	Chr8: 80.868085	-0.089

252 ☐	MA_M2F_0706_R	1426476_at	*Rasa1*	RAS p21 protein activator (GTPase activating protein) 1; mid 3' UTR	Chr13: 85.215413	9.256	65	13.4	Chr14: 19.755208	0.203
253 ☐	MA_M2F_0706_R	1449658_at	*AA536748*	AA536748	Chr5: 49.657134	5.759	65	13.7	Chr4: 102.851020	0.037
254 ☐	MA_M2F_0706_R	1421022_x_at	*Acyp1*	acylphosphatase 1, erythrocyte (common) type	Chr12: 85.272453	10.369	65	12.6	Chr4: 96.896949	0.180
255 ☐	MA_M2F_0706_R	1439266_a_at	*Polr3k*	polymerase (RNA) III (DNA directed) polypeptide K	Chr2: 181.868495	7.426	65	16.2	Chr14: 19.755208	0.207
256 ☐	MA_M2F_0706_R	1428985_at	*Ints12*	integrator complex subunit 12; distal 3' UTR	Chr3: 133.110469	8.464	65	12.5	Chr14: 9.528965	0.257
257 ☐	MA_M2F_0706_R	1456744_x_at	*Flcn*	folliculin; distal 3'UTR	Chr11: 59.791771	5.895	65	12.4	Chr11: 83.064807	0.059
258 ☐	MA_M2F_0706_R	1435529_at	*Ifit1*	interferon-induced protein with tetratricopeptide repeats 1 related sequence, OTTMUSG00000016644; 3' UTR of Ifit1-related sequence (from 2010002M12Rik)	Chr19: 34.593014	7.121	65	16.6	Chr14: 19.755208	0.150
259 ☐	MA_M2F_0706_R	1427197_at	*Atr*	ataxia telangiectasia and rad3 related; last four coding exons	Chr9: 95.945370	7.738	65	12.3	Chr3: 39.650830	-0.160
260 ☐	MA_M2F_0706_R	1417319_at	*Pvrl3*	poliovirus receptor-related 3	Chr16: 46.447261	8.254	65	14.6	Chr14: 24.000000	0.192
261 ☐	MA_M2F_0706_R	1417077_at	*Bcap29*	B-cell receptor-associated protein 29; mid 3' UTR	Chr12: 31.595531	8.748	65	9.4	Chr10: 55.515150	-0.222
262 ☐	MA_M2F_0706_R	1453282_at	*Cxadr*	coxsackievirus and adenovirus receptor	Chr16: 78.339777	8.041	65	13.1	Chr14: 19.755208	0.144
263 ☐	MA_M2F_0706_R	1433823_at	*AW456874*	expressed sequence AW456874	Chr13: 48.578313	6.592	65	13.5	Chr8: 3.500000	0.124
264 ☐	MA_M2F_0706_R	1424230_at	*Exoc6*	exocyst complex component 6	Chr19: 37.682952	8.747	65	13.8	ChrX: 61.058643	-0.143
265 ☐	MA_M2F_0706_R	1417704_a_at	*Arhgap6*	Rho GTPase activating protein 6	ChrX: 169.303893	6.311	65	11.3	Chr8: 94.374289	-0.070
266 ☐	MA_M2F_0706_R	1432094_a_at	*Vps50*	VPS50 EARP/GARPII complex subunit; last two exons and 3' UTR	Chr6: 3.600215	9.359	65	12.4	ChrX: 64.161107	-0.205

267 ☐	MA_M2F_0706_R	1435116_at	*Kiaa1383*	KIAA1383, RIKEN cDNA 4933403G14; 3' UTR	Chr8: 125.672794	6.191	65	12.1	Chr14: 9.528965	0.088
268 ☐	MA_M2F_0706_R	1420340_at	*Cspp1*	centrosome and spindle pole associated protein 1; mid 3' UTR of long form	Chr1: 10.067376	5.798	65	10.4	Chr18: 68.185097	0.037
269 ☐	MA_M2F_0706_R	1424075_at	*9430016H08Rik*	hypothetical protein LOC79568; last three exons	Chr1: 57.411776	8.937	65	15.6	Chr4: 96.896949	0.174
270 ☐	MA_M2F_0706_R	1446471_at	*B130066H01Rik*	15 days embryo head cDNA, RIKEN full-length enriched library, clone:D930045O10 product:unknown EST, full insert sequence.	Chr7: 129.649450	7.061	65	23.2	Chr14: 9.528965	-0.068
271 ☐	MA_M2F_0706_R	1435938_at	*2610318C08Rik*	RIKEN cDNA 2610318C08 gene	Chr2: 129.268259	8.392	65	17.5	Chr2: 134.519309	-0.098
272 ☐	MA_M2F_0706_R	1435127_a_at	*Osgepl1*	O-sialoglycoprotein endopeptidase-like 1	Chr1: 53.320085	9.391	65	10.8	Chr16: 3.500000	-0.115
273 ☐	MA_M2F_0706_R	1448653_at	*Eed*	embryonic ectoderm development (polycomb repressive complex 2 member); last three exons and 3' UTR	Chr7: 89.954826	9.601	65	16.4	Chr7: 73.746984	0.240
274 ☐	MA_M2F_0706_R	1423131_at	*Tnip1*	TGF beta-inducible nuclear protein 1; far 3' UTR	Chr13: 97.129610	9.356	65	10.0	Chr14: 24.000000	0.165
275 ☐	MA_M2F_0706_R	1424641_a_at	*Thoc1*	THO complex 1	Chr18: 9.993531	7.371	65	11.8	Chr14: 19.755208	0.280
276 ☐	MA_M2F_0706_R	1450418_a_at	*Yipf4*	Yip1 domain family, member 4; 3' UTR	Chr17: 74.499383	9.199	65	10.1	Chr7: 84.149847	0.140
277 ☐	MA_M2F_0706_R	1417769_at	*Psmc6*	proteasome (prosome, macropain) 26S subunit, ATPase, 6; far 3' UTR	Chr14: 45.349105	6.746	65	11.3	Chr17: 10.720847	-0.175
278 ☐	MA_M2F_0706_R	1426015_s_at	*Asph*	aspartate-beta-hydroxylase	Chr4: 9.575561	9.877	65	8.7	Chr11: 93.000000	0.163
279 ☐	MA_M2F_0706_R	1417861_at	*Ccnc*	cyclin C; distal 3' UTR	Chr4: 21.749997	6.742	65	12.5	Chr14: 19.755208	0.133
280 ☐	MA_M2F_0706_R	1434284_at	*Bdp1*	B double prime 1, subunit of RNA polymerase III transcription initiation factor IIIB; distal 3' UTR	Chr13: 100.018106	9.309	65	12.9	Chr14: 9.528965	0.143

281 ☐	MA_M2F_0706_R	1459885_s_at	*Cox7c*	cytochrome c oxidase, subunit VIIc	Chr13: 86.044921	6.580	65	16.9	ChrX: 42.981911	-0.096
282 ☐	MA_M2F_0706_R	1423612_at	*Clp1*	ATP/GTP-binding protein; last exon and proximal 3' UTR	Chr2: 84.723372	8.531	65	14.8	Chr2: 134.800968	0.158
283 ☐	MA_M2F_0706_R	1452385_at	*Usp53*	ubiquitin specific protease 53	Chr3: 122.931918	6.647	65	13.8	Chr14: 114.751225	0.175
284 ☐	MA_M2F_0706_R	1415981_at	*Herpud2*	HERPUD family member 2; 3' UTR	Chr9: 25.108158	10.215	65	10.7	Chr11: 83.064807	0.156
285 ☐	MA_M2F_0706_R	1420390_s_at	*Zfp354a*	zinc finger protein 354A; proximal 3' UTR	Chr11: 51.070706	6.468	65	16.0	Chr7: 73.746984	0.077
286 ☐	MA_M2F_0706_R	1418651_at	*Spata6*	spermatogenesis associated 6	Chr4: 111.828580	8.411	65	10.5	Chr16: 3.500000	-0.097
287 ☐	MA_M2F_0706_R	1416856_at	*C20orf111*	oxidative stress responsive 1 (peroxide-inducible transcript 1, human chromosome 20 open reading frame 111); 3' UTR	Chr2: 163.405836	10.353	65	12.7	Chr11: 83.064807	0.138
288 ☐	MA_M2F_0706_R	1452256_at	*1110002N22Rik*	RIKEN cDNA 1110002N22 (hydrolase); last exon	Chr11: 80.136947	8.639	65	11.0	Chr14: 24.000000	0.149
289 ☐	MA_M2F_0706_R	1454136_a_at	*C16orf87*	human chromosome 16 open reading frame 87; mid distal 3' UTR	Chr8: 85.409136	8.866	65	25.9	Chr8: 81.951757	-0.315
290 ☐	MA_M2F_0706_R	1421014_a_at	*Clybl*	citrate lyase beta like; exons 5 and 6	Chr14: 122.379201	10.975	65	15.8	Chr4: 102.851020	0.188
291 ☐	MA_M2F_0706_R	1448979_at	*Muted*	muted	Chr13: 38.602741	9.480	65	15.7	Chr14: 9.528965	0.142
292 ☐	MA_M2F_0706_R	1428772_at	*Tbk1*	TANK-binding kinase 1; distal 3'UTR	Chr10: 121.587538	9.799	65	9.5	Chr10: 121.639039	0.157
293 ☐	MA_M2F_0706_R	1425476_at	*Col4a5*	procollagen, type IV, alpha 5	ChrX: 141.688618	10.329	65	16.7	Chr14: 24.000000	0.204
294 ☐	MA_M2F_0706_R	1424372_at	*Mrpl32*	mitochondrial ribosomal protein L32	Chr13: 14.610401	11.353	65	19.8	Chr8: 75.681677	-0.120
295 ☐	MA_M2F_0706_R	1451133_s_at	*8430437G11Rik*	RIKEN cDNA 8430437G11 gene	Chr6: 13.581615	8.029	65	12.9	Chr14: 3.000000	0.121
296 ☐	MA_M2F_0706_R	1426494_at	*Rg9mtd3*	RNA (guanine-9-) methyltransferase domain containing 3	Chr4: 45.307913	7.507	65	23.8	Chr14: 9.528965	0.205

297 ☐	MA_M2F_0706_R	1440799_s_at	*Farp2*	FERM, RhoGEF and pleckstrin domain protein 2; distal 3' UTR and antisense of the long Stk25	Chr1: 93.621605	6.953	65	13.0	Chr6: 42.000000	-0.215
298 ☐	MA_M2F_0706_R	1442836_at	*Xpo7*	exportin 7	Chr14: 70.680650	5.598	65	10.4	Chr13: 115.551768	-0.034
299 ☐	MA_M2F_0706_R	1454766_at	*Amn1*	antagonist of mitotic exit network 1; last four exons	Chr6: 149.157710	7.872	65	13.4	ChrX: 73.428893	-0.211
300 ☐	MA_M2F_0706_R	1428616_at	*Zfp131*	zinc finger protein 131; distal 3' UTR	Chr13: 119.481127	9.074	65	12.4	Chr19: 28.714120	-0.169
301 ☐	MA_M2F_0706_R	1454947_a_at	*Ub1cp1*	ubiquitin-like domain containing CTD phosphatase 1; distal 3' UTR	Chr11: 44.454597	10.071	65	10.3	Chr1: 95.094462	0.141
302 ☐	MA_M2F_0706_R	1426523_a_at	*Gnpda2*	glucosamine-6-phosphate deaminase 2; exons 4, 6 (last), and proximal 3' UTR	Chr5: 69.575857	8.163	65	13.4	Chr14: 3.000000	0.299
303 ☐	MA_M2F_0706_R	1448712_at	*Chm*	choroidermia; mid 3'UTR	ChrX: 113.041468	8.544	65	14.2	Chr14: 9.528965	0.175
304 ☐	MA_M2F_0706_R	1424318_at	*Hspc159*	galectin-related protein (protein LOC29094); distal 3' UTR	Chr11: 20.823747	9.926	65	11.1	Chr11: 82.187018	0.142
305 ☐	MA_M2F_0706_R	1418048_at	*1110059G10Rik*	RIKEN cDNA 1110059G10 gene	Chr9: 122.947701	10.525	65	16.5	Chr11: 83.064807	0.152
306 ☐	MA_M2F_0706_R	1434910_at	*CXorf23*	human chromosome X open reading frame 23; distal half of 3' UTR	ChrX: 159.592515	7.462	65	16.5	Chr14: 9.528965	0.160
307 ☐	MA_M2F_0706_R	1454064_a_at	*Rnf138*	ring finger protein 138; mid 3' UTR	Chr18: 21.026824	8.397	65	13.0	Chr14: 24.000000	0.179
308 ☐	MA_M2F_0706_R	1417140_a_at	*Ptpn2*	protein tyrosine phosphatase, non-receptor type 2; last exon and 3' UTR	Chr18: 67.665655	8.719	65	14.9	Chr14: 9.528965	0.147
309 ☐	MA_M2F_0706_R	1424589_s_at	*Rnpc3*	RNA-binding region (RNP1, RRM) containing 3; last four exons	Chr3: 113.609053	6.364	65	9.3	Chr8: 88.711457	-0.077
310 ☐	MA_M2F_0706_R	1419241_a_at	*Aire*	autoimmune regulator (autoimmune polyendocrinopathy candidiasis ectodermal dystrophy); last two exons and 3' UTR	Chr10: 78.030046	7.881	65	16.9	Chr14: 9.528965	-0.110

311 ☐	MA_M2F_0706_R	1427016_at	*Kiaa1109*	transmembrane protein KIAA1109; last exon and proximal to mid 3' UTR	Chr3: 37.052439	9.507	65	9.9	Chr11: 83.064807	0.189
312 ☐	MA_M2F_0706_R	1447124_at	*Gal3st2*	galactose 3-O-sulfotransferase 2 region; probes in region with segmental duplication	Chr1: 93.858131	10.833	65	13.2	Chr7: 84.149847	-0.147
313 ☐	MA_M2F_0706_R	1421888_x_at	*Aplp2*	amyloid beta (A4) precursor-like protein 2; distal 3' UTR	Chr9: 31.149603	10.659	65	10.2	Chr3: 39.650830	0.074
314 ☐	MA_M2F_0706_R	1438903_at	*Maea*	Mus musculus transcribed sequence	--	6.324	65	15.0	Chr4: 107.548653	0.065
315 ☐	MA_M2F_0706_R	1452126_at	*Zfp160*	zinc finger protein 160	Chr17: 21.028146	7.362	65	17.5	Chr14: 9.528965	0.136
316 ☐	MA_M2F_0706_R	1449090_a_at	*Yes1*	Yamaguchi sarcoma viral (v-yes) oncogene homolog 1; distal 3' UTR	Chr5: 32.686555	7.483	65	9.3	Chr13: 7.000000	0.152
317 ☐	MA_M2F_0706_R	1428427_at	*Fbxl2*	F-box and leucine-rich repeat protein 2; proximal 3' UTR	Chr9: 113.978252	6.140	65	16.7	ChrX: 73.428893	-0.053
318 ☐	MA_M2F_0706_R	1417399_at	*Gas6*	growth arrest specific 6 (Axl ligand); last 2 exons and 3' UTR	Chr8: 13.465414	13.763	65	11.7	Chr9: 99.750326	-0.146
319 ☐	MA_M2F_0706_R	1426269_at	*Sybl1*	synaptobrevin like 1 (pseudoautosomal region); distal 3' UTR	ChrX: 1.000000	10.834	65	9.4	Chr16: 3.500000	-0.152
320 ☐	MA_M2F_0706_R	1429376_s_at	*Anapc10*	anaphase promoting complex subunit 10	Chr8: 79.729186	9.142	65	15.3	Chr14: 19.755208	0.196
321 ☐	MA_M2F_0706_R	1428078_at	*0610013E23Rik*	RIKEN cDNA 0610013E23; proximal 3' UTR	Chr11: 86.495970	9.107	65	13.3	Chr11: 82.187018	0.242
322 ☐	MA_M2F_0706_R	1458193_at	*Fabp9*	fatty acid binding protein 9, testis	Chr3: 10.180050	8.970	65	12.0	ChrX: 70.094108	0.084
323 ☐	MA_M2F_0706_R	1428629_at	*6330417C12Rik*	RIKEN cDNA 6330417C12 gene	Chr19: 40.917374	7.183	65	10.0	Chr8: 80.868085	-0.150
324 ☐	MA_M2F_0706_R	1417478_a_at	*G5pr*	protein phosphatase component G5PR (associated component of GANP up-regulated in GCâ€“B cells); four exons and proximal 3' UTR	Chr12: 55.281558	8.955	65	10.1	Chr14: 9.528965	0.157
325 ☐	MA_M2F_0706_R	1417493_at	*Bmi1*	B lymphoma Mo-MLV insertion region 1; mid 3' UTR	Chr2: 18.685363	10.100	65	11.1	Chr11: 83.064807	0.182

326		MA_M2F_0706_R	1429623_at	*Zfp644*	zinc finger protein 644; mid 3' UTR	Chr5: 106.617461	7.624	65	20.0	Chr14: 19.755208	0.123
327		MA_M2F_0706_R	1456324_at	*Zfp748*	zinc finger protein 748 (KRAB type, regulator of sex-limitation); mid 3' UTR	Chr13: 67.539283	6.526	65	15.1	Chr14: 3.000000	0.144
328		MA_M2F_0706_R	1440704_at	*Lgr8*	leucine-rich repeat-containing G protein-coupled receptor 8	Chr5: 150.081733	6.396	65	13.0	Chr19: 10.708414	0.077
329		MA_M2F_0706_R	1426536_at	*Narg2*	NMDA receptor-regulated gene 2; mid to distal 3' UTR	Chr9: 69.432487	8.334	65	17.7	Chr14: 3.000000	0.213
330		MA_M2F_0706_R	1433865_at	*E330016L19Rik*	RIKEN cDNA E330016L19 gene	ChrX: 27.268743	5.536	65	12.8	Chr13: 112.636263	-0.025
331		MA_M2F_0706_R	1421202_at	*Chrna4*	cholinergic receptor, nicotinic, alpha 4 (high affinity nicotine receptor with Chrnb2); last two exons and proximal 3' UTR	Chr2: 181.024702	8.688	65	10.9	Chr13: 3.150000	-0.100
332		MA_M2F_0706_R	1434267_at	*Nek1*	NIMA (never in mitosis gene a)-related expressed kinase 1 (serine/threonine kinase, polycystic kidney disease, axonal development); distal half of 3' UTR	Chr8: 61.130775	6.748	65	12.2	Chr14: 19.749489	0.119
333		MA_M2F_0706_R	1454507_at	*8430432A02Rik*	RIKEN cDNA 8430432A02 gene	Chr1: 43.093250	5.890	65	15.6	Chr14: 9.528965	0.043
334		MA_M2F_0706_R	1452972_at	*1700013G20Rik*	RIKEN cDNA 1700013G20 gene	Chr12: 9.034923	9.434	65	10.8	Chr17: 10.720847	-0.128
335		MA_M2F_0706_R	1449341_a_at	*Stom*	stomatin	Chr2: 35.315873	9.342	65	8.7	Chr11: 24.905498	0.111
336		MA_M2F_0706_R	1429759_at	*Rps6ka6*	ribosomal protein S6 kinase polypeptide 6	ChrX: 111.388762	7.264	65	9.6	Chr14: 19.755208	0.120
337		MA_M2F_0706_R	1418530_at	*Nup160*	nucleoporin 160	Chr2: 90.735779	7.490	65	15.9	Chr14: 3.000000	0.234
338		MA_M2F_0706_R	1453030_at	*Msl2*	male-specific lethal-2 homolog (Drosophila)	Chr9: 101.102224	7.942	65	18.5	Chr11: 83.064807	0.177
339		MA_M2F_0706_R	1429557_at	*Mcm8*	minichromosome maintenance deficient 8 (S. cerevisiae)	Chr2: 132.839569	6.128	65	13.6	Chr4: 102.851020	0.072

340 ☐	MA_M2F_0706_R	1440142_s_at	*Gfap*	glial fibrillary acidic protein; intron 7 or possible short form 3' UTR	Chr11: 102.890227	5.771	65	15.1	Chr14: 9.528965	0.055
341 ☐	MA_M2F_0706_R	1451783_a_at	*Kifap3*	kinesin-associated protein 3; distal half of 3' UTR	Chr1: 163.916502	9.055	65	15.7	Chr11: 83.064807	0.178
342 ☐	MA_M2F_0706_R	1418381_at	*Zfp148*	zinc finger protein 148; mid-proximal 3' UTR	Chr16: 33.498186	6.265	65	14.1	Chr14: 3.000000	0.187
343 ☐	MA_M2F_0706_R	1416163_at	*Cops4*	COP9 (constitutive photomorphogenic) homolog, subunit 4; last two exons and proximal 3' UTR	Chr5: 100.543853	10.157	65	10.6	Chr14: 19.755208	0.104
344 ☐	MA_M2F_0706_R	1434563_at	*Rps6kc1*	ribosomal protein S6 kinase polypeptide 1; mid-proximal 3' UTR or intron	Chr1: 190.773292	7.684	65	13.5	Chr14: 19.755208	-0.106
345 ☐	MA_M2F_0706_R	1450167_at	*Rab37*	RAB37, member of RAS oncogene family (vesicle trafficking); exons 2, 3, 4, 5, 6, 8, and 9	Chr11: 115.156940	6.874	65	9.4	Chr14: 49.789692	-0.054
346 ☐	MA_M2F_0706_R	1428252_at	*Chmp2b*	chromatin modifying protein 2B (charged multivesicular body protein 2b, vacuolar protein sorting 2-2, frontotemporal lobe dementia); 3' UTR	Chr16: 65.539277	10.515	65	11.1	Chr11: 83.064807	0.162
347 ☐	MA_M2F_0706_R	1423615_at	*Zfp364*	zinc finger protein 364; mid 3'UTR	Chr3: 96.790269	10.319	65	16.5	Chr11: 4.784489	-0.099
348 ☐	MA_M2F_0706_R	1435675_at	*Tbc1d12*	TBC1D12: TBC1 domain family, member 12	Chr19: 38.919081	9.389	65	16.0	Chr14: 19.755208	0.165
349 ☐	MA_M2F_0706_R	1427982_s_at	*Syne2*	synaptic nuclear envelope 2	Chr12: 76.110344	9.159	65	14.2	Chr7: 84.149847	0.229
350 ☐	MA_M2F_0706_R	1435923_at	*Gm237*	gene model 237, (NCBI); distal 3' UTR	Chr10: 67.545156	8.114	65	14.1	Chr11: 83.064807	0.149
351 ☐	MA_M2F_0706_R	1455633_at	*Zfp647*	zinc finger protein 647; last exon and 3' UTR	Chr15: 76.910433	6.571	65	12.6	Chr14: 24.000000	0.122
352 ☐	MA_M2F_0706_R	1435373_at	*Csnk1e*	casein kinase 1, epsilon; intron 7 or alternative short form 3' UTR	Chr15: 79.422854	7.472	65	14.1	Chr13: 112.636263	0.088
353 ☐	MA_M2F_0706_R	1456757_at	*G630024C07Rik*	RIKEN cDNA G630024C07 gene	Chr7: 127.253925	5.848	65	11.3	Chr10: 67.616312	-0.046

354 ☐	MA_M2F_0706_R	1423672_at	*Ttc30b*	tetratricopeptide repeat domain 30B; last exon, proximal and mid 3' UTR	Chr2: 75.936094	8.604	65	13.4	Chr14: 3.000000	0.230
355 ☐	MA_M2F_0706_R	1418066_at	*Cfl2*	cofilin 2, muscle; distal 3' UTR	Chr12: 54.858855	9.192	65	17.8	ChrX: 47.876769	-0.199
356 ☐	MA_M2F_0706_R	1435150_at	*9030221C07*	Mus musculus transcribed sequences	Chr5: 44.227847	6.226	65	9.5	Chr13: 112.636263	-0.113
357 ☐	MA_M2F_0706_R	1434542_at	*Gpt2*	glutamic pyruvate transaminase (alanine aminotransferase) 2; mid 3' UTR	Chr8: 85.526690	8.799	65	10.5	Chr2: 134.800968	-0.158
358 ☐	MA_M2F_0706_R	1452268_at	*Fam76b*	family with sequence similarity 76, member B	Chr9: 13.845945	8.475	65	13.4	ChrX: 64.161107	-0.189
359 ☐	MA_M2F_0706_R	1448045_at	*Ptpn9*	protein tyrosine phosphatase, non-receptor type 9	Chr9: 57.057487	6.074	65	12.4	Chr7: 68.215797	0.052
360 ☐	MA_M2F_0706_R	1435020_at	*Klhdc2*	kelch domain containing 2; putative far 3' UTR (overlaps anti-sense strand of Sdccag1)	Chr12: 69.311244	7.875	65	15.8	Chr14: 24.000000	0.189
361 ☐	MA_M2F_0706_R	1424369_at	*Psmf1*	proteasome (prosome, macropain) inhibitor subunit 1	Chr2: 151.717187	7.313	65	10.1	Chr14: 9.528965	0.192
362 ☐	MA_M2F_0706_R	1455487_at	*Mfsd11*	major facilitator superfamily domain containing 11; proximal 3' UTR	Chr11: 116.874123	10.170	65	15.7	Chr4: 102.851020	0.186
363 ☐	MA_M2F_0706_R	1435001_at	*Plaa*	phospholipase A2, activating protein; mid 3' UTR	Chr4: 94.567229	6.738	65	13.0	Chr14: 19.755208	0.118
364 ☐	MA_M2F_0706_R	1452612_at	*Ltn1*	listerin E3 ubiquitin protein ligase 1	Chr16: 87.378907	7.370	65	11.7	Chr14: 9.528965	0.134
365 ☐	MA_M2F_0706_R	1427114_at	*Ttc19*	tetratricopeptide repeat domain 19 (mitochondrial)	Chr11: 62.314658	7.098	65	15.2	Chr14: 9.528965	0.155
366 ☐	MA_M2F_0706_R	1435103_x_at	*Farslb*	phenylalanine-tRNA synthetase-like, beta subunit; last exon and proximal 3' UTR	Chr1: 78.425104	10.670	65	15.2	Chr6: 43.826811	-0.183
367 ☐	MA_M2F_0706_R	1452896_at	*Gtl3*	gene trap locus 3	Chr8: 95.421200	8.300	65	14.4	Chr8: 94.374289	-0.173
368 ☐	MA_M2F_0706_R	AFFX-LysX-M_at	*AFFX-LysX-M*	Affymetrix bacterial LysX gene control probe set; background signal only	ChrZ: 1.000000	7.262	65	11.4	Chr14: 33.093246	0.200

369 ☐	MA_M2F_0706_R	1418579_at	*Cetn2*	centrin 2	ChrX: 72.913915	9.910	65	17.8	ChrX: 71.441039	-0.236
370 ☐	MA_M2F_0706_R	1416680_at	*Ube3a*	ubiquitin protein ligase E3A; last four exons (long form)	Chr7: 59.288405	10.568	65	31.1	Chr7: 73.746984	0.288
371 ☐	MA_M2F_0706_R	1454234_at	*1700111I05Rik*	RIKEN cDNA 1700111I05 gene	Chr5: 134.901838	8.081	65	18.5	Chr14: 9.546676	-0.091
372 ☐	MA_M2F_0706_R	1417508_at	*Rnf19*	ring finger protein (C3HC4 type) 19	Chr15: 36.240023	9.815	65	16.8	Chr14: 9.528965	0.165
373 ☐	MA_M2F_0706_R	1418968_at	*Rb1cc1*	RB1-inducible coiled-coil 1; mid 3'UTR	Chr1: 6.274578	10.245	65	10.1	Chr10: 53.743148	-0.097
374 ☐	MA_M2F_0706_R	1449214_a_at	*Opa1*	optic atrophy 1, mitochondrial dynamin like GTPase (mitochondrial fusion); exons 23, 24, 25, and 26	Chr16: 29.628750	8.261	65	9.1	Chr4: 107.548653	0.116
375 ☐	MA_M2F_0706_R	1433986_at	*LOC221710*	BC024659 (glycosyl hydrolase 2 family member); distal 3' UTR	Chr13: 41.276072	8.324	65	14.0	Chr14: 9.528965	0.144
376 ☐	MA_M2F_0706_R	1448540_a_at	*0610012G03Rik*	RIKEN cDNA 0610012G03 gene	Chr16: 31.947861	10.690	65	12.3	ChrX: 70.094108	-0.124
377 ☐	MA_M2F_0706_R	1430744_at	*Napsa*	napsin A aspartic peptidase	Chr7: 44.582619	7.970	65	8.8	Chr4: 75.824944	-0.073
378 ☐	MA_M2F_0706_R	1428341_at	*Zfp655*	zinc finger protein 655; distal 3' UTR	Chr5: 145.246762	10.169	65	13.4	ChrX: 70.094108	-0.137
379 ☐	MA_M2F_0706_R	1420561_at	*Trpc7*	transient receptor potential cation channel, subfamily C, member 7; 3' UTR	Chr13: 56.773292	10.223	65	8.3	Chr13: 112.636263	0.111
380 ☐	MA_M2F_0706_R	1453989_at	*Stxbp4*	syntaxin binding protein 4	Chr11: 90.599446	7.728	65	16.5	Chr2: 78.100320	-0.080
381 ☐	MA_M2F_0706_R	1455679_at	*AI852561*	RIKEN cDNA 4930434H03 gene	Chr1: 51.464994	7.380	65	11.0	Chr18: 57.293889	0.131
382 ☐	MA_M2F_0706_R	1415689_s_at	*Zkscan3*	zinc finger with KRAB and SCAN domains 3; mid 3' UTR	Chr13: 21.387433	7.693	65	10.8	Chr3: 74.704013	-0.110
383 ☐	MA_M2F_0706_R	1436770_x_at	*Psma1*	proteasome (prosome, macropain) subunit, alpha type 1; intron, mid 3'UTR, and exon	Chr7: 114.264747	5.620	65	15.2	Chr14: 9.528965	0.058
384 ☐	MA_M2F_0706_R	1419351_a_at	*C11orf73*	human chromosome 11 open reading	Chr7: 89.918925	9.009	65	19.1	Chr7: 84.149847	0.135

				frame 73; last three exons and proximal 3' UTR						
385 ☐	MA_M2F_0706_R	1428749_at	*Dmxl2*	Dmx-like 2; distal 3' UTR	Chr9: 54.365209	9.961	65	12.9	Chr11: 83.064807	0.153
386 ☐	MA_M2F_0706_R	1434869_at	*Tdrd3*	tudor domain containing 3 (transcriptional coactivator, stress granule associated); distal 3' UTR of short form message	Chr14: 87.514185	6.214	65	9.7	Chr12: 31.211373	0.070
387 ☐	MA_M2F_0706_R	1456604_a_at	*Pcmt1*	protein-L-isoaspartate(D-aspartate) O-methyltransferase; proximal 3' UTR	Chr10: 7.630734	10.436	65	13.5	Chr4: 96.896949	0.150
388 ☐	MA_M2F_0706_R	1430535_at	*Tsc22d2*	TSC22 domain family 2; distal 3' UTR	Chr3: 58.461244	8.035	65	9.3	Chr12: 30.182102	0.190
389 ☐	MA_M2F_0706_R	1456038_at	*Fbxl4*	F-box and leucine-rich repeat protein 4; 2 exons and proximal 3'UTR	Chr4: 22.427368	8.935	65	11.1	Chr14: 9.528965	0.160
390 ☐	MA_M2F_0706_R	1435682_at	*Lars2*	leucyl-tRNA synthetase, mitochondrial; distal 3' UTR (test Mendelian 1.04, apparent transQTL)	Chr9: 123.462289	9.884	65	11.9	Chr14: 3.000000	0.093
391 ☐	MA_M2F_0706_R	1420139_s_at	*Krr1*	KRR1, small subunit (SSU) processome component, homolog; mid 3' UTR	Chr10: 111.985871	9.281	65	11.7	Chr14: 24.000000	0.190
392 ☐	MA_M2F_0706_R	1460585_x_at	*Pisd*	phosphatidylserine decarboxylase; mid 3' UTR (non-specific probe also aligns to Pisd-ps1, Ch 11 at 3.124090 Mb)	Chr5: 32.736783	12.657	65	12.8	Chr12: 51.647704	-0.104
393 ☐	MA_M2F_0706_R	1442656_at	*Elovl6*	ELOVL family member 6, elongation of long chain fatty acids (yeast)	Chr3: 129.548768	6.792	65	10.5	Chr1: 35.935430	0.075
394 ☐	MA_M2F_0706_R	1449140_at	*Nudcd2*	NudC domain containing 2; proximal 3' UTR	Chr11: 40.739363	7.811	65	11.4	Chr14: 24.000000	0.156
395 ☐	MA_M2F_0706_R	1436706_at	*Tmem32*	transmembrane protein 32	ChrX: 56.586781	8.848	65	13.5	Chr14: 3.000000	0.199
396 ☐	MA_M2F_0706_R	1431252_a_at	*Zfp655*	zinc finger protein 655	Chr5: 145.244666	7.540	65	15.0	ChrX: 61.058643	-0.188

397 ☐	MA_M2F_0706_R	1427831_s_at	*Zfp260*	zinc finger protein 260	Chr7: 30.105601	7.368	65	13.7	Chr14: 3.000000	0.220
398 ☐	MA_M2F_0706_R	1416794_at	*Atl2*	atlastin GTPase 2 (ADP-ribosylation factor-like 6 interacting protein 2); exon 9	Chr17: 79.852664	8.921	65	8.8	Chr15: 72.500609	-0.153
399 ☐	MA_M2F_0706_R	1430623_s_at	*5830411E10Rik*	RIKEN cDNA 5830411E10 gene	Chr1: 51.470742	9.677	65	11.6	Chr10: 67.616312	-0.160
400 ☐	MA_M2F_0706_R	1421139_a_at	*Zfp386*	zinc finger protein 386 (Kruppel-like); last exon	Chr12: 116.059259	7.447	65	11.5	Chr14: 3.000000	0.198
401 ☐	MA_M2F_0706_R	1451337_at	*Psmf1*	proteasome (prosome, macropain) inhibitor subunit 1; last 2 exons and proximal 3' UTR	Chr2: 151.718622	9.984	65	12.3	Chr10: 67.616312	-0.123
402 ☐	MA_M2F_0706_R	1419276_at	*Enpp1*	ectonucleotide pyrophosphatase/phosphodiesterase 1	Chr10: 24.645301	9.196	65	10.5	Chr2: 134.800968	0.170
403 ☐	MA_M2F_0706_R	1420999_at	*Cnot4*	CCR4-NOT transcription complex, subunit 4	Chr6: 35.046068	8.044	65	9.0	Chr2: 134.800968	0.132
404 ☐	MA_M2F_0706_R	1429186_a_at	*Cdadc1*	cytidine and dCMP deaminase domain containing 1	Chr14: 59.586352	8.359	65	9.5	Chr2: 134.800968	0.152
405 ☐	MA_M2F_0706_R	1416115_at	*Orc3l*	origin recognition complex, subunit 3-like (S. cerevisiae)	Chr4: 34.572464	9.689	65	12.2	Chr2: 134.462008	0.117
406 ☐	MA_M2F_0706_R	1425022_at	*Usp3*	ubiquitin specific protease 3; last 4 exons and proximal 3' UTR	Chr9: 66.518371	10.137	65	8.9	Chr10: 48.377966	-0.104
407 ☐	MA_M2F_0706_R	1420977_at	*Man1a2*	mannosidase, alpha, class 1A, member 2	Chr3: 100.565465	8.629	65	20.6	Chr14: 9.528965	0.208
408 ☐	MA_M2F_0706_R	1450762_s_at	*Zfp191*	zinc finger protein 191; proximal 3' UTR	Chr18: 24.013534	7.585	65	8.4	Chr15: 63.392263	-0.073
409 ☐	MA_M2F_0706_R	1451619_at	*Golph3l*	golgi phosphoprotein 3-like; last exon and proximal 3' UTR	Chr3: 95.617448	8.351	65	10.4	ChrX: 84.597186	-0.174
410 ☐	MA_M2F_0706_R	1421908_a_at	*Tcf12*	transcription factor 12; distal 3' UTR	Chr9: 71.845794	9.714	65	14.8	Chr15: 69.108222	-0.166
411 ☐	MA_M2F_0706_R	1431784_a_at	*2310066N05Rik*	RIKEN cDNA 2310066I18 gene	Chr3: 146.512172	8.362	65	12.7	ChrX: 64.161107	-0.138
412 ☐	MA_M2F_0706_R	1451317_at	*Ythdf2*	YTH domain family 2; last two exons and proximal 3' UTR	Chr4: 132.186852	9.743	65	11.8	Chr15: 69.108222	-0.183

413 ☐	MA_M2F_0706_R	1438264_a_at	*Tpp2*	tripeptidyl peptidase II (serine exopeptidase of the 26S proteasome); three exons	Chr1: 43.980362	8.214	65	8.6	Chr14: 3.000000	0.191
414 ☐	MA_M2F_0706_R	1420959_at	*Asph*	aspartate-beta-hydroxylase; mid-distal 3' UTR	Chr4: 9.451848	8.133	65	12.9	Chr14: 19.755208	0.215
415 ☐	MA_M2F_0706_R	1421448_at	*Garnl1*	GTPase activating RANGAP domain-like 1; exons 32, 33, 34, and 35	Chr12: 55.640606	8.533	65	14.9	ChrX: 73.428893	-0.235
416 ☐	MA_M2F_0706_R	1417442_a_at	*Pex3*	peroxisomal biogenesis factor 3	Chr10: 13.532375	9.350	65	11.7	Chr13: 25.000000	0.144
417 ☐	MA_M2F_0706_R	1419803_s_at	*Ccdc12*	coiled-coil domain containing 12; last three exons and proximal 3' UTR	Chr9: 110.711126	11.139	65	12.8	Chr1: 5.641533	0.080
418 ☐	MA_M2F_0706_R	1437295_at	*Pkn2*	protein kinase N2	Chr3: 142.792158	9.473	65	13.0	Chr15: 69.108222	-0.195
419 ☐	MA_M2F_0706_R	1451770_s_at	*Dhx9*	DEAH (Asp-Glu-Ala-His) box polypeptide 9	Chr1: 153.455871	10.770	65	10.6	Chr18: 65.174965	-0.126
420 ☐	MA_M2F_0706_R	1416132_at	*Efr3a*	EFR3 homolog A (hearing loss associated, activity-dependent plasticity-associated multi-pass membrane KIAA0143 protein); five exons	Chr15: 65.854698	7.781	65	11.3	Chr10: 67.616312	-0.151
421 ☐	MA_M2F_0706_R	1415789_a_at	*LOC665689*	similar to ubiquitin-like domain containing CTD phosphatase 1; putative exon (from ESTs)	Chr11: 16.635159	9.069	65	13.2	Chr11: 42.818374	0.286
422 ☐	MA_M2F_0706_R	1418650_at	*Spata6*	spermatogenesis associated 6	Chr4: 111.799138	8.253	65	11.8	Chr10: 64.710346	-0.088
423 ☐	MA_M2F_0706_R	1451730_at	*Zfp62*	zinc finger protein 62	Chr11: 49.217237	7.295	65	11.0	Chr14: 3.000000	0.126
424 ☐	MA_M2F_0706_R	1434979_at	*4933403F05Rik*	RIKEN cDNA 4933403F05 gene	Chr18: 68.267767	8.473	65	16.6	ChrX: 73.876578	-0.203
425 ☐	MA_M2F_0706_R	1423961_at	*Wdr26*	WD repeat domain 26 (putative Mtv7, Mls1); putative far 3' UTR	Chr1: 181.176207	9.623	65	13.3	Chr15: 67.990415	-0.160
426 ☐	MA_M2F_0706_R	1434332_at	*Zzz3*	zinc finger, ZZ domain containing 3; mid to distal 3' UTR	Chr3: 152.458886	10.318	65	13.1	Chr2: 181.014276	0.115

				(transQTL on chr 4 in BXD Eye Data)						
427 ☐	MA_M2F_0706_R	1460399_at	*BC018601*	cDNA sequence BC018601	Chr11: 5.529291	9.077	65	11.2	Chr12: 29.713200	0.106
428 ☐	MA_M2F_0706_R	1426842_at	*Ythdf3*	YTH domain family 3; proximal and mid 3' UTR	Chr3: 16.214439	10.305	65	10.2	Chr14: 3.000000	0.176
429 ☐	MA_M2F_0706_R	1415857_at	*Emb*	embigin; exon 6 and proximal 3' UTR	Chr13: 117.267470	9.796	65	14.3	Chr10: 67.616312	-0.205
430 ☐	MA_M2F_0706_R	1420850_at	*Crnkl1*	Crn, crooked neck-like 1 (Drosophila)	Chr2: 145.920593	7.867	65	11.9	Chr15: 68.818097	-0.109
431 ☐	MA_M2F_0706_R	1428775_at	*Kiaa0391*	mitochondrial ribonuclease P protein 3	Chr12: 55.377219	8.427	65	15.8	Chr12: 46.816738	0.109
432 ☐	MA_M2F_0706_R	1427321_s_at	*Cxadr*	coxsackievirus and adenovirus receptor; distal 3' UTR	Chr16: 78.338806	8.548	65	12.8	Chr10: 67.616312	-0.155
433 ☐	MA_M2F_0706_R	1426411_a_at	*Strbp*	spermatid perinuclear RNA binding protein (interleukin enhancer binding factor 3-like)	Chr2: 37.586519	7.887	65	11.6	ChrX: 73.428893	-0.191
434 ☐	MA_M2F_0706_R	1456867_x_at	*Ergic3*	ERGIC and golgi 3; last exon	Chr2: 156.018075	11.707	65	10.4	Chr7: 142.462521	-0.087
435 ☐	MA_M2F_0706_R	1448127_at	*Rrm1*	ribonucleotide reductase M1; distal 3' UTR	Chr7: 102.459425	8.437	65	11.4	Chr2: 136.127470	0.158
436 ☐	MA_M2F_0706_R	1428845_at	*Bclaf1*	BCL2-associated transcription factor 1; distal 3' UTR (transQTL on Chr 4 in BXD eye data)	Chr10: 20.341571	10.077	65	9.7	Chr16: 3.500000	-0.152
437 ☐	MA_M2F_0706_R	1456255_at	*Kiaa0368*	proteasome-associated protein ECM29 homolog	Chr4: 58.844163	6.439	65	8.7	Chr15: 63.392263	-0.085
438 ☐	MA_M2F_0706_R	1427658_at	*Ctbs*	chitobiase, di-N-acetyl-	Chr3: 146.459677	8.061	65	11.6	Chr2: 134.800968	0.187
439 ☐	MA_M2F_0706_R	1449666_at	*Atrnl1*	attractin like 1; antisense of distal 3' UTR	Chr19: 58.132875	8.404	65	12.5	Chr10: 73.927005	0.049
440 ☐	MA_M2F_0706_R	1448309_at	*Ap3m1*	adaptor-related protein complex 3, mu 1 subunit; last four exons and proximal 3' UTR	Chr14: 21.036673	8.785	65	12.4	ChrX: 61.058643	-0.179
441 ☐	MA_M2F_0706_R	1426216_at	*Cog6*	component of oligomeric golgi complex 6	Chr3: 52.982493	10.023	65	13.6	ChrX: 92.675500	-0.134

442	MA_M2F_0706_R	1457949_at	*Nt5c2*	5'-nucleotidase, cytosolic II; putative exon	Chr19: 46.885497	7.808	65	9.6	Chr10: 53.743148	-0.089
443	MA_M2F_0706_R	1437348_at	*Fbxo28*	F-box protein 28	Chr1: 182.315610	9.004	65	9.2	Chr2: 136.127470	0.106
444	MA_M2F_0706_R	1427466_at	*Pigu*	phosphatidylinositol glycan anchor biosynthesis, class U (Pigu)	Chr2: 155.278639	7.436	65	14.9	Chr15: 28.321742	0.109
445	MA_M2F_0706_R	1418369_at	*Prim1*	DNA primase, p49 subunit	Chr10: 128.023845	8.677	65	10.9	Chr11: 83.064807	0.171
446	MA_M2F_0706_R	1425491_at	*Bmpr1a*	bone morphogenetic protein receptor, type 1A	Chr14: 34.414005	8.247	65	18.8	ChrX: 73.428893	-0.170
447	MA_M2F_0706_R	1429250_at	*Dnchc2*	dynein, cytoplasmic, heavy chain 2; last 4 exons (transQTL on Chr 4 in BXD eye data)	Chr9: 6.929469	9.350	65	10.0	Chr2: 138.500000	0.110
448	MA_M2F_0706_R	1421880_at	*Mtmr1*	myotubularin related protein 1	ChrX: 71.418081	8.728	65	16.7	ChrX: 73.428893	-0.153
449	MA_M2F_0706_R	1453263_at	*Mak10*	MAK10 homolog, amino-acid N-acetyltransferase subunit, (S. cerevisiae)	Chr13: 59.600932	7.823	65	9.0	Chr2: 134.800968	0.116
450	MA_M2F_0706_R	1418974_at	*Blzf1*	basic leucine zipper nuclear factor 1	Chr1: 164.292202	8.466	65	9.2	Chr12: 51.647704	0.097
451	MA_M2F_0706_R	1434918_at	*Sox6*	SRY-box containing gene 6	Chr7: 115.471185	9.714	65	11.0	Chr11: 83.064807	0.113
452	MA_M2F_0706_R	1452504_s_at	*Ctbs*	chitobiase, di-N-acetyl-	Chr3: 146.458794	9.429	65	11.4	Chr13: 22.380465	0.193
453	MA_M2F_0706_R	1419749_at	*Dnmt2*	DNA methyltransferase 2	Chr2: 13.511244	8.022	65	14.0	Chr14: 3.000000	0.223
454	MA_M2F_0706_R	1426456_a_at	*Miz1*	Msx-interacting-zinc finger; exons 8, 9, 10, and 11	Chr18: 77.133220	9.302	65	15.7	ChrX: 73.876578	-0.152
455	MA_M2F_0706_R	1450915_at	*Ap3b1*	adaptor-related protein complex 3, beta 1 subunit; exons 13, 14, 15, and 16	Chr13: 94.462396	9.153	65	9.8	Chr15: 89.576205	-0.191
456	MA_M2F_0706_R	1448348_at	*Gpiap1*	cell cycle associated protein 1 (cytoplasmic activation- and proliferation-associated protein 1); exons 9 through 12	Chr2: 103.773010	9.668	65	12.2	Chr15: 89.576205	-0.207

457 ☐	MA_M2F_0706_R	1424397_at	*Dhx36*	DEAH (Asp-Glu-Ala-His) box polypeptide 36	Chr3: 62.470459	7.069	65	12.8	Chr14: 3.000000	0.133
458 ☐	MA_M2F_0706_R	1426824_at	*Psme4*	proteasome (prosome, macropain) activator subunit 4	Chr11: 30.856055	10.899	65	16.3	ChrX: 73.028443	-0.227
459 ☐	MA_M2F_0706_R	1425473_at	*Crsp6*	cofactor required for Sp1 transcriptional activation, subunit 6; exon and 3'UTR	Chr9: 15.262099	9.343	65	11.2	Chr4: 107.548653	0.120
460 ☐	MA_M2F_0706_R	1429029_at	*4933405A16Rik*	RIKEN cDNA 4933405A16 gene	Chr3: 131.322799	8.258	65	12.0	Chr12: 51.647704	0.181
461 ☐	MA_M2F_0706_R	1451968_at	*Xrcc5*	X-ray repair complementing defective repair in Chinese hamster cells 5; last five exons	Chr1: 72.381622	9.455	65	11.2	Chr8: 25.520489	-0.096
462 ☐	MA_M2F_0706_R	1455439_a_at	*Lgals1*	lectin, galactose binding, soluble 1; last exon and proximal 3' UTR	Chr15: 78.930026	13.086	65	10.7	Chr6: 49.309149	0.171
463 ☐	MA_M2F_0706_R	1440817_x_at	*Zfp771*	zinc finger protein 771; proximal 3' UTR	Chr7: 127.254639	10.800	65	10.1	Chr1: 35.935430	0.100
464 ☐	MA_M2F_0706_R	1429720_at	*Mak10*	MAK10 homolog, amino-acid N-acetyltransferase subunit, (S. cerevisiae)	Chr13: 59.617941	8.448	65	11.3	Chr2: 134.800968	0.185
465 ☐	MA_M2F_0706_R	1428307_at	*Zdhhc13*	zinc finger, DHHC domain containing 13; 2 exons and 3'UTR	Chr7: 48.826869	9.834	65	11.9	Chr10: 67.616312	-0.100
466 ☐	MA_M2F_0706_R	1434400_at	*Tgif2*	TGFB-induced factor 2	Chr2: 156.855011	8.399	65	17.2	Chr10: 67.616312	0.111
467 ☐	MA_M2F_0706_R	1419548_at	*Kpna1*	karyopherin (importin) alpha 1	Chr16: 36.035423	8.390	65	10.5	Chr14: 3.000000	0.198
468 ☐	MA_M2F_0706_R	1454690_at	*Ikbkg*	inhibitor of kappaB kinase gamma	ChrX: 74.451245	8.521	65	14.0	Chr2: 136.127470	0.111
469 ☐	MA_M2F_0706_R	1423159_at	*Dld*	dihydrolipoamide dehydrogenase; 2 exons and 3'UTR	Chr12: 31.332098	12.505	65	11.4	Chr13: 20.437691	0.124
470 ☐	MA_M2F_0706_R	1423748_at	*Pdk1*	pyruvate dehydrogenase kinase isoenzyme 1; distal 3' UTR	Chr2: 71.901415	9.698	65	10.9	Chr6: 44.021906	-0.121
471 ☐	MA_M2F_0706_R	1425497_a_at	*Prpf4b*	PRP4 pre-mRNA processing factor 4 homolog B; last 3 exons	Chr13: 34.899199	7.694	65	11.9	ChrX: 73.876578	-0.139

472	MA_M2F_0706_R	1443117_at	*Eya1*	eyes absent 1 homolog (Drosophila)	Chr1: 14.301362	6.115	65	8.5	Chr10: 67.616312	0.064
473	MA_M2F_0706_R	1450745_at	*C1galt1*	core 1 synthase, glycoprotein-N-acetylgalactosamine 3-beta-galactosyltransferase	Chr6: 7.871503	9.260	65	9.8	ChrX: 84.597186	-0.168
474	MA_M2F_0706_R	1451124_at	*Sod1*	superoxide dismutase 1, soluble; first four exons and 3' UTR	Chr16: 90.222782	13.768	65	8.3	Chr2: 17.898115	0.198
475	MA_M2F_0706_R	1452062_at	*Prpsap2*	phosphoribosyl pyrophosphate synthetase-associated protein 2	Chr11: 61.729821	9.996	65	9.7	Chr10: 67.616312	-0.104
476	MA_M2F_0706_R	1426463_at	*Gphn*	gephyrin (GABA and glycine receptor scaffolding protein); last four exons and proximal 3' UTR	Chr12: 78.682757	9.513	65	9.3	Chr3: 128.292533	0.167
477	MA_M2F_0706_R	1422772_at	*C1galt1*	core 1 synthase, glycoprotein-N-acetylgalactosamine 3-beta-galactosyltransferase; putative far 3' UTR	Chr6: 7.872849	8.974	65	13.5	ChrX: 64.161107	-0.256
478	MA_M2F_0706_R	1421333_a_at	*Mynn*	myoneurin	Chr3: 30.611514	7.431	65	18.1	ChrX: 73.876578	-0.170
479	MA_M2F_0706_R	1422990_at	*Met*	met proto-oncogene; last 3 exons and proximal 3' UTR	Chr6: 17.563641	8.746	65	13.6	Chr14: 81.380779	-0.212
480	MA_M2F_0706_R	1421508_at	*Odz1*	odd Oz/ten-m homolog 1	ChrX: 42.532423	6.520	65	15.8	Chr10: 73.927005	0.068
481	MA_M2F_0706_R	1444566_at	*Ucp2*	uncoupling protein 2 (mitochondrial, proton carrier)	Chr7: 100.505342	7.751	65	11.0	Chr15: 29.045905	0.063
482	MA_M2F_0706_R	1416706_at	*Rpe*	ribulose-5-phosphate-3-epimerase; last exon and proximal 3' UTR	Chr1: 66.717830	9.394	65	11.7	Chr15: 68.818097	-0.174
483	MA_M2F_0706_R	1459976_s_at	*Sod1*	superoxide dismutase 1, soluble; last exon and proximal 3' UTR	Chr16: 90.226197	13.425	65	11.0	Chr15: 67.990415	0.187
484	MA_M2F_0706_R	1443962_at	*Tfdp2*	transcription factor Dp 2	Chr9: 96.310545	7.909	65	9.4	Chr2: 136.127470	0.120
485	MA_M2F_0706_R	1416061_at	*Tbc1d15*	TBC1 domain family, member 15; last three exons and proximal 3' UTR	Chr10: 115.199419	10.012	65	14.9	Chr10: 55.515150	-0.129
486	MA_M2F_0706_R	1455378_at	*Rimkla*	ribosomal protein S6 modification-like protein; distal 3' UTR	Chr4: 119.465329	6.074	65	9.5	Chr9: 108.969240	-0.040

487 ☐	MA_M2F_0706_R	1454929_s_at	*AU018122*	hypothetical protein E130307D12	Chr17: 56.606034	10.913	65	13.9	Chr2: 138.500000	-0.140
488 ☐	MA_M2F_0706_R	1426264_at	*Dlat*	dihydrolipoamide S-acetyltransferase (E2 component of pyruvate dehydrogenase complex); last exon and proximal 3' UTR	Chr9: 50.636189	10.979	65	10.0	Chr15: 67.990415	-0.101
489 ☐	MA_M2F_0706_R	1422216_at	*Mid2*	midline 2	ChrX: 140.764824	7.309	65	18.7	Chr14: 9.528965	0.138
490 ☐	MA_M2F_0706_R	1455541_a_at	*4430402I18Rik*	RIKEN cDNA 4430402I18 gene	Chr19: 28.927648	6.988	65	23.1	Chr2: 134.519309	0.132
491 ☐	MA_M2F_0706_R	1423822_a_at	*8430437G11Rik*	RIKEN cDNA 8430437G11 gene	Chr6: 13.582027	9.336	65	13.3	Chr16: 3.500000	-0.171
492 ☐	MA_M2F_0706_R	1440790_x_at	*AL024069*	expressed sequence AL024069	Chr1: 84.259943	6.355	65	9.8	Chr10: 67.616312	0.086
493 ☐	MA_M2F_0706_R	1454811_a_at	*Serinc1*	serine incorporator 1; last exon and proximal 3' UTR	Chr10: 57.517129	11.035	65	14.5	ChrX: 59.235496	-0.207
494 ☐	MA_M2F_0706_R	1441208_at	*Hdhd2*	haloacid dehalogenase-like hydrolase domain containing; putative deep 3' UTR	Chr18: 76.973281	8.113	65	12.5	Chr14: 9.546676	0.077
495 ☐	MA_M2F_0706_R	1418527_a_at	*Srsf10*	serine/arginine-rich splicing factor 10; exons 3, 4, and 5	Chr4: 135.861784	8.100	65	11.4	Chr14: 3.000000	0.171
496 ☐	MA_M2F_0706_R	1456671_at	*Tbrg3*	transforming growth factor beta regulated gene 3	Chr15: 82.896065	7.023	65	11.4	Chr10: 67.616312	0.047
497 ☐	MA_M2F_0706_R	1434469_at	*Otud4*	OTU domain containing 4; mid 3' UTR	Chr8: 79.675793	7.926	65	14.9	ChrX: 73.876578	-0.251
498 ☐	MA_M2F_0706_R	1431811_a_at	*Fbxo34*	F-box only protein 34; 3' UTR from proximal to distal	Chr14: 47.531295	9.179	65	7.3	Chr14: 19.755208	0.102
499 ☐	MA_M2F_0706_R	1437809_x_at	*Ets2*	E26 avian leukemia oncogene 2, 3' domain	Chr16: 95.720926	6.756	65	9.3	Chr10: 67.616312	0.123
500 ☐	MA_M2F_0706_R	1454238_a_at	*1700010H22Rik*	RIKEN cDNA 1700010H22 gene	Chr5: 98.564199	7.563	65	13.8	Chr2: 137.000000	-0.096
501 ☐	MA_M2F_0706_R	1422842_at	*Xrn2*	5'-3' exoribonuclease 2; exons 8, 9, and 10	Chr2: 147.027593	8.379	65	10.1	Chr2: 134.800968	0.218
502 ☐	MA_M2F_0706_R	1449532_at	*Chrng*	cholinergic receptor, nicotinic, gamma polypeptide (developmental	Chr1: 87.210631	7.939	65	8.8	Chr14: 9.528965	-0.123

				subunit in muscle replaced by Chrne); last three exons and proximal 3' UTR						
503 ☐	MA_M2F_0706_R	1458791_at	*Gbe1*	glucan (1,4-alpha-), branching enzyme 1	Chr16: 70.490294	5.736	65	14.4	Chr2: 134.519309	0.074
504 ☐	MA_M2F_0706_R	1433908_a_at	*Cttn*	cortactin; distal 3' UTR	Chr7: 144.435770	12.923	65	12.4	Chr7: 52.768893	-0.117
505 ☐	MA_M2F_0706_R	1433582_at	*C3orf58*	human chromosome 3 open reading frame 58; mid 3' UTR	Chr9: 94.518936	7.085	65	10.1	Chr14: 19.755208	0.104
506 ☐	MA_M2F_0706_R	1450348_at	*Slc19a3*	solute carrier family 19 (sodium/hydrogen exchanger), member 3	Chr1: 83.014703	7.710	65	10.4	Chr3: 25.041091	0.159
507 ☐	MA_M2F_0706_R	1452496_at	*Atp11c*	Atpase, class VI, type 11C	ChrX: 60.236631	6.884	65	9.6	Chr11: 103.979646	-0.102
508 ☐	MA_M2F_0706_R	1424280_at	*Mospd1*	motile sperm domain containing 1; mid to distal 3' UTR	ChrX: 53.345058	9.189	65	9.3	Chr11: 4.784489	-0.132
509 ☐	MA_M2F_0706_R	1434127_a_at	*H3f3a*	H3 histone, family 3A; distal 3' UTR	Chr1: 180.802580	15.517	65	12.0	Chr4: 81.726726	0.109
510 ☐	MA_M2F_0706_R	1419501_at	*Polk*	polymerase (DNA directed), kappa	Chr13: 96.483584	7.720	65	9.5	Chr15: 67.990415	-0.046
511 ☐	MA_M2F_0706_R	1416759_at	*Mical1*	microtubule associated monoxygenase, calponin and LIM domain containing 1	Chr10: 41.485859	6.466	65	12.6	Chr2: 134.800968	-0.107
512 ☐	MA_M2F_0706_R	1423198_a_at	*Smek2*	SMEK homolog 2 suppressor of mek1; four exons	Chr11: 29.200761	8.665	65	9.8	Chr2: 134.800968	0.195
513 ☐	MA_M2F_0706_R	1425023_at	*Usp3*	ubiquitin specific protease 3	Chr9: 66.517750	7.999	65	11.0	Chr4: 102.851020	0.128
514 ☐	MA_M2F_0706_R	1444573_at	*C130040D06Rik*	ESTs, Weakly similar to apoptosis-associated tyrosine kinase [] [M.musculus]	Chr7: 45.793185	6.762	65	13.4	Chr2: 134.800968	-0.128
515 ☐	MA_M2F_0706_R	1419471_a_at	*Nudc*	nudC nuclear distribution protein; exon 5	Chr4: 133.534290	9.751	65	11.0	Chr11: 83.064807	-0.116
516 ☐	MA_M2F_0706_R	1427937_at	*Vma21*	vacuolar ATPase assembly integral membrane protein VMA21; 3' UTR (low specficity probes)	ChrX: 71.821526	8.308	65	10.1	Chr14: 3.000000	0.181

517 ☐	MA_M2F_0706_R	1428074_at	*Tmem158*	transmembrane protein 158; 3' UTR	Chr9: 123.259067	8.373	65	10.8	Chr16: 97.368149	-0.130
518 ☐	MA_M2F_0706_R	1455019_x_at	*Ckap4*	cytoskeleton-associated protein 4; distal 3' UTR	Chr10: 84.526376	9.254	65	11.5	Chr10: 67.616312	0.098
519 ☐	MA_M2F_0706_R	1428543_at	*Ppat*	phosphoribosyl pyrophosphate amidotransferase	Chr5: 76.915968	7.279	65	8.5	Chr17: 10.720847	-0.182
520 ☐	MA_M2F_0706_R	1452780_at	*Gtf3c2*	general transcription factor IIIC, polypeptide 2, beta; last exon and 3' UTR (complex 3' UTR structure)	Chr5: 31.157418	9.868	65	8.5	ChrX: 84.597186	-0.097
521 ☐	MA_M2F_0706_R	1453368_at	*2310003H01Rik*	RIKEN cDNA 2310003H01 gene	Chr11: 120.376210	8.276	65	13.0	Chr2: 134.800968	-0.104
522 ☐	MA_M2F_0706_R	1426451_at	*Spg11*	spastic paraplegia 11 (autosomal recessive); last 4 exons and 3' UTR	Chr2: 122.053846	8.990	65	9.0	Chr14: 3.000000	0.131
523 ☐	MA_M2F_0706_R	1423121_at	*Ide*	insulin degrading enzyme	Chr19: 37.269994	7.630	65	14.5	Chr14: 9.528965	0.207
524 ☐	MA_M2F_0706_R	1417514_at	*Ssx2ip*	synovial sarcoma, X breakpoint 2 interacting protein; last three exons and proximal 3' UTR	Chr3: 146.436598	8.375	65	21.8	ChrX: 73.876578	-0.180
525 ☐	MA_M2F_0706_R	1419049_at	*Pcnx*	pecanex homolog; mid 3' UTR	Chr12: 81.998196	6.302	65	9.5	Chr10: 73.927005	-0.056
526 ☐	MA_M2F_0706_R	1433018_at	*4930560O18Rik*	RIKEN cDNA 4930560O18 gene	Chr7: 120.101793	5.954	65	9.7	Chr13: 90.420702	-0.039
527 ☐	MA_M2F_0706_R	1418502_a_at	*Oxr1*	oxidation resistance 1; mid 3' UTR	Chr15: 41.859688	8.971	65	11.7	Chr15: 12.672705	-0.137
528 ☐	MA_M2F_0706_R	1453463_at	*1700019B03Rik*	RIKEN cDNA 1700019B03 gene	Chr8: 3.483752	6.225	65	14.2	Chr2: 134.519309	0.063
529 ☐	MA_M2F_0706_R	1451222_at	*Btf3l4*	basic transcription factor 3-like 4; mid 3' UTR	Chr4: 108.815322	9.795	65	9.9	Chr14: 3.000000	0.147
530 ☐	MA_M2F_0706_R	1425705_a_at	*Ero1lb*	endoplasmic oxidoreductase 1 beta; four exons	Chr13: 12.600282	6.870	65	11.8	ChrX: 73.876578	-0.073
531 ☐	MA_M2F_0706_R	1427258_at	*Trim24*	tripartite motif protein 24; last 2 exons and proximal half of 3' UTR	Chr6: 37.965621	8.653	65	9.2	Chr2: 138.500000	0.143
532 ☐	MA_M2F_0706_R	1437179_at	*Rif1*	replication timing regulatory factor 1	Chr2: 52.103552	7.510	65	11.4	Chr2: 134.800968	0.259

				(telomere-associated protein); exons 20, 21, 22						
533	MA_M2F_0706_R	1453533_at	*4933403O08Rik*	RIKEN cDNA 4933403O08 gene	ChrX: 112.243333	6.049	65	10.3	Chr10: 121.639039	0.043
534	MA_M2F_0706_R	1451500_at	*Ushbp1*	Usher syndrome 1C binding protein 1; last three exons and 3' UTR	Chr8: 71.385386	8.258	65	13.9	Chr10: 64.710346	0.106
535	MA_M2F_0706_R	1450052_at	*Kif2a*	kinesin family member 2A	Chr13: 68.474071	8.025	65	10.0	Chr11: 9.165457	-0.109
536	MA_M2F_0706_R	1438365_x_at	*Laptm4b*	lysosomal-associated protein transmembrane 4B; distal 3' UTR	Chr15: 34.284138	13.172	65	11.7	Chr2: 136.127470	-0.134
537	MA_M2F_0706_R	1449336_a_at	*Slk*	STE20-like kinase (yeast)	Chr19: 47.638928	8.150	65	11.7	Chr14: 3.000000	0.158
538	MA_M2F_0706_R	1435876_at	*C86350*	expressed sequence C86350	Chr6: 130.727814	5.776	65	9.1	Chr14: 9.528965	0.026
539	MA_M2F_0706_R	1429110_a_at	*Nsun4*	NOL1/NOP2/Sun domain family, member 4 (mitochondrial ribosome 5-methylcytosine RNA methyltransferase)	Chr4: 116.034034	8.796	65	10.0	ChrX: 73.028443	-0.138
540	MA_M2F_0706_R	1456347_at	*Lnpep*	leucyl/cystinyl aminopeptidase	Chr17: 17.531573	7.029	65	8.7	Chr3: 128.292533	-0.107
541	MA_M2F_0706_R	1426806_at	*Obfc2a*	oligonucleotide/oligosaccharide-binding fold; mid 3' UTR	Chr1: 51.470336	9.935	65	8.9	ChrX: 71.441039	-0.128
542	MA_M2F_0706_R	1429734_at	*4632434I11Rik*	RIKEN cDNA 4632434I11 gene	Chr7: 92.858364	6.074	65	11.8	Chr14: 9.528965	0.074
543	MA_M2F_0706_R	1444121_at	*LOC231291*	hypothetical protein LOC231291	Chr13: 59.131302	6.200	65	15.0	Chr10: 71.957979	0.073
544	MA_M2F_0706_R	1421044_at	*Mrc2*	mannose receptor, C type 2	Chr11: 105.348315	7.429	65	14.4	Chr14: 3.000000	-0.091
545	MA_M2F_0706_R	1415682_at	*Xpo7*	exportin 7	Chr14: 70.664923	9.069	65	16.1	Chr15: 68.818097	-0.100
546	MA_M2F_0706_R	1426896_at	*Zfp191*	zinc finger protein 191	Chr18: 24.010778	8.131	65	10.6	Chr14: 3.000000	0.196
547	MA_M2F_0706_R	1426265_x_at	*Dlat*	dihydrolipoamide S-acetyltransferase (E2 component of pyruvate	Chr9: 50.636189	11.137	65	13.3	Chr7: 84.149847	0.122

				dehydrogenase complex); last exon and proximal 3' UTR						
548	MA_M2F_0706_R	1418109_at	*Gspt2*	G1 to S phase transition 2; only exon and 3' UTR	ChrX: 94.637967	7.203	65	15.1	Chr2: 134.519309	0.107
549	MA_M2F_0706_R	1438142_s_at	*1700021K14Rik*	RIKEN cDNA 1700021K14 gene	Chr9: 107.997994	6.574	65	11.5	Chr18: 68.914646	-0.060
550	MA_M2F_0706_R	1416162_at	*Rad21*	RAD21 homolog; last four exons	Chr15: 51.964055	9.706	65	8.8	Chr10: 67.616312	-0.179
551	MA_M2F_0706_R	1433724_at	*Fam91a1*	family with sequence similarity 91, member A1 (skeletal muscle cells re-entry induced); mid 3' UTR	Chr15: 58.456028	8.907	65	8.6	Chr2: 134.800968	0.150
552	MA_M2F_0706_R	1451519_at	*Rnf2*	ring finger protein 2	Chr1: 151.470414	9.050	65	11.5	Chr11: 4.784489	-0.142
553	MA_M2F_0706_R	1426563_at	*Zfp553*	zinc finger protein 553; last exon and 3' UTR	Chr7: 127.236909	7.302	65	14.5	Chr14: 9.528965	-0.122
554	MA_M2F_0706_R	1422801_at	*G3bp*	Ras-GTPase-activating protein SH3-domain binding protein (interferon, alpha-inducible protein); mid 3' UTR	Chr11: 55.499735	11.713	65	7.1	Chr15: 67.990415	0.145
555	MA_M2F_0706_R	1460531_at	*Syne1*	spectrin repeat containing, nuclear envelope 1; apparent intron (from AK013759)	Chr10: 5.287650	6.519	65	8.5	Chr9: 41.233417	0.053
556	MA_M2F_0706_R	1437903_at	*Lox*	lysyl oxidase	Chr18: 52.517103	5.877	65	16.5	Chr2: 136.127956	0.051
557	MA_M2F_0706_R	1442486_at	*Leprel1*	leprecan-like 1	Chr16: 23.037936	6.250	65	11.3	Chr2: 156.631489	0.054
558	MA_M2F_0706_R	1441176_at	*Lincr*	ESTs	Chr7: 51.612266	6.185	65	11.6	Chr2: 136.127470	-0.108
559	MA_M2F_0706_R	1425339_at	*Plcb4*	phospholipase C, beta 4; four exons (central exons or 3' exons of short form)	Chr2: 135.971757	6.665	65	11.1	Chr2: 136.127470	0.071
560	MA_M2F_0706_R	1460157_at	*Smc1l2*	SMC (structural maintenance of chromosomes 1)-like 2 (S. cerevisiae)	Chr19: 20.241215	6.008	65	10.3	Chr14: 19.755208	0.037
561	MA_M2F_0706_R	1440377_at	*Tigd5*	tigger transposable element derived 5 homolog	Chr15: 75.913188	6.829	65	16.9	Chr14: 3.000000	-0.100

562 ☐	MA_M2F_0706_R	1422838_at	*Kcnu1*	potassium channel, subfamily U, member 1 (potassium large conductance pH-sensitive channel, subfamily M, alpha member 3)	Chr8: 25.932408	6.054	65	13.6	Chr14: 9.528965	0.050
563 ☐	MA_M2F_0706_R	1442372_at	*A630050E13Rik*	RIKEN cDNA A630050E13 gene	Chr17: 75.532282	6.479	65	8.5	Chr2: 158.947668	-0.048
564 ☐	MA_M2F_0706_R	1427003_at	*Ppp2r5c*	protein phosphatase 2, regulatory subunit B (B56), gamma isoform; 3' end intron or 3' UTR	Chr12: 110.571149	8.542	65	12.5	Chr4: 84.752005	0.117
565 ☐	MA_M2F_0706_R	1430362_at	*Sorbs1*	sorbin and SH3 domain containing 1; intron	Chr19: 40.472821	6.735	65	16.1	ChrX: 71.441039	0.074
566 ☐	MA_M2F_0706_R	1421756_a_at	*Gpr19*	G protein-coupled receptor 19 (strong cisQTL in eye); center and 3' end of exon	Chr6: 134.869544	7.356	65	9.4	Chr14: 9.528965	0.090
567 ☐	MA_M2F_0706_R	1428887_at	*Zfp157*	zinc finger protein 157; last exon	Chr5: 138.456534	6.224	65	10.3	ChrX: 84.597186	-0.065
568 ☐	MA_M2F_0706_R	1422694_at	*Ttyh1*	tweety homolog 1 (Drosophila); proximal 3' UTR	Chr7: 4.134350	6.081	65	11.2	Chr2: 134.800968	-0.062
569 ☐	MA_M2F_0706_R	1425199_a_at	*Epb4.1l5*	erythrocyte protein band 4.1-like 5; last exon and 3'UTR	Chr1: 119.595376	10.901	65	11.8	Chr2: 181.014276	0.125

Supplemental Table S11. Top 200 genes in male for 3 probes in Ace2

Dataset	Trait ID	Symbol	Description	Location	Mean	N Cases	Max LRS ?	Max LRS Location Chr and Mb	Add ?	
1 ☐	MA_M2M_0706_R	1425103_at	*Ace2*	angiotensin I converting enzyme (peptidyl-dipeptidase A) 2; middle to distal 3'-UTR	ChrX: 164.187853	10.201	45	12.0	Chr1: 14.576853	0.169

2	MA_M2M_0706_R	1452138_a_at	*Ace2*	angiotensin I converting enzyme (peptidyl-dipeptidase A) 2; last 2~6 and 11 exons	ChrX: 164.167855	10.125	45	13.1	Chr8: 89.094303	0.214
3	MA_M2M_0706_R	1430561_at	*Dnajb14*	DnaJ (Hsp40) homolog, subfamily B, member 14; proximal and mid 3' UTR	Chr3: 137.908489	8.283	45	11.7	Chr2: 78.100320	0.259
4	MA_M2M_0706_R	1438719_at	*AI585793*	expressed sequence AI585793	Chr18: 32.232049	7.583	45	13.0	Chr18: 31.556239	-0.209
5	MA_M2M_0706_R	1425102_a_at	*Ace2*	angiotensin I converting enzyme (peptidyl-dipeptidase A) 2; 3'-UTR and last 4 exons	ChrX: 164.182645	10.127	45	10.2	Chr9: 107.639250	0.185
6	MA_M2M_0706_R	1438736_at	*Thoc2*	THO complex 2; four exons	ChrX: 41.822401	8.389	45	8.7	Chr3: 53.053282	-0.152
7	MA_M2M_0706_R	1440486_at	*C12orf41*	human chromosome 12 open reading frame 41; far 3' UTR	Chr15: 98.517815	7.542	45	8.7	Chr13: 3.150000	0.081
8	MA_M2M_0706_R	1423341_at	*Cspg4*	chondroitin sulfate proteoglycan 4	Chr9: 56.899348	8.585	45	11.4	Chr6: 40.799296	0.151
9	MA_M2M_0706_R	1457304_at	*Jarid2*	jumonji, AT rich interactive domain 2	Chr13: 44.756689	5.955	45	7.4	Chr5: 24.321400	0.076
10	MA_M2M_0706_R	1439661_at	*Slc16a14*	RIKEN cDNA 1110004H10 gene	Chr1: 84.905979	8.885	45	16.1	Chr3: 65.524002	-0.349
11	MA_M2M_0706_R	1457639_at	*Atp6v1h*	ATPase, H+ transporting, lysosomal, V1 subunit H; intronic or alternative splicing (AK081492)	Chr1: 5.099623	6.534	45	9.4	Chr2: 69.342849	0.116
12	MA_M2M_0706_R	1459392_at	*Phf8*	PHD finger protein 8	ChrX: 151.625300	7.100	45	11.4	Chr6: 125.820102	0.082
13	MA_M2M_0706_R	1436874_x_at	*Slc25a5*	solute carrier family 25 (mitochondrial carrier, adenine nucleotide translocator),	ChrX: 36.797832	16.402	45	8.3	ChrX: 102.414526	0.081

				member 5; last two exons and 3' UTR						
14 ☐	MA_M2M_0706_R	1456717_at	*Tead1*	TEA domain family member 1 (SV40 transcriptional enhancer factor, Sveinsson's chorioretinal atrophy)	Chr7: 112.681339	5.970	45	8.6	Chr3: 129.49747 0	0.148
15 ☐	MA_M2M_0706_R	1442251_at	*Vcpip1*	valosin containing protein (p97)/p47 complex interacting protein 1	Chr1: 9.744461	5.641	45	9.7	Chr9: 29.939029	-0.041
16 ☐	MA_M2M_0706_R	1442277_at	*Chka*	choline kinase alpha; last intron or 3' UTR	Chr19: 3.865325	8.170	45	8.2	Chr2: 69.724603	0.171
17 ☐	MA_M2M_0706_R	1415878_at	*Rrm1*	ribonucleotide reductase M1	Chr7: 102.468420	8.552	45	12.5	Chr1: 80.325854	0.073
18 ☐	MA_M2M_0706_R	1460151_at	--	polymorphic LTR element; NACHT, leucine rich repeat and PYD containing 1	Chr1: 13.621363	8.231	45	24.6	Chr1: 13.073341	-0.322
19 ☐	MA_M2M_0706_R	1451458_at	*Tmem2*	transmembrane protein 2; 4 exons	Chr19: 21.852229	8.663	45	7.4	Chr1: 58.115573	-0.157
20 ☐	MA_M2M_0706_R	1457106_at	*BC052885*	10 days neonate cerebellum cDNA, RIKEN full-length enriched library, clone:6530405K19 product:similar to DEATH RECEPTOR-INTERACTING PROTEIN [Homo sapiens], full insert sequence.	Chr17: 84.191519	7.275	45	10.6	Chr3: 57.334874	0.077
21 ☐	MA_M2M_0706_R	1450849_at	*Hnrnpu*	heterogeneous nuclear ribonucleoprotein U; five central exons	Chr1: 178.330821	12.202	45	13.8	Chr7: 24.937915	0.416
22 ☐	MA_M2M_0706_R	1437082_at	*Akap9*	A kinase (PRKA) anchor protein (yotiao) 9; exons 3, 4, and 5 and distal end of intron 3	Chr5: 3.954395	6.823	45	13.1	Chr2: 178.717318	0.120
23 ☐	MA_M2M_0706_R	1422495_a_at	*Hmgn1*	high mobility group nucleosomal binding domain 1; mid 3' UTR	Chr16: 18.938220	13.533	45	9.3	Chr10: 87.643399	-0.098

24 ☐	MA_M2M_0706_R	1424084_at	*Rod1*	ROD1 regulator of differentiation 1 (S. pombe)	Chr4:59.475963	5.859	45	12.5	Chr1:62.725845	-0.111
25 ☐	MA_M2M_0706_R	1441580_at	*Sgpp2*	sphingosine-1-phosphate phosphatase 2	Chr1:78.392722	7.537	45	11.8	Chr4:131.99242	-0.078
26 ☐	MA_M2M_0706_R	1421412_at	*Gsc*	goosecoid	Chr12:104.471487	6.271	45	11.5	Chr6:23.372761	0.050
27 ☐	MA_M2M_0706_R	1417231_at	*Cldn2*	claudin 2	ChrX:139.810790	14.468	45	15.5	Chr15:93.021696	0.130
28 ☐	MA_M2M_0706_R	1436746_at	*Prkwnk1*	protein kinase, lysine deficient 1; exons 4, 6 and 7	Chr6:119.969525	9.367	45	9.9	Chr2:18.263847	-0.355
29 ☐	MA_M2M_0706_R	1436157_at	*Ccar1*	cell division cycle and apoptosis regulator; exons 14 through 17	Chr10:62.753390	11.141	45	13.6	Chr2:18.263847	-0.257
30 ☐	MA_M2M_0706_R	1436747_at	*LOC280487*	pol polyprotein	Chr11:116.438594	12.033	45	13.7	Chr13:103.949936	-0.144
31 ☐	MA_M2M_0706_R	1420500_at	*Dnajc1*	DnaJ (Hsp40) homolog, subfamily C, member 1; last 2 exons and 3' UTR	Chr2:18.217204	8.557	45	12.0	Chr13:103.949936	0.169
32 ☐	MA_M2M_0706_R	1456979_at	*Zhx3*	zinc fingers and homeoboxes 3	Chr2:160.856133	8.270	45	6.8	Chr9:68.339182	0.120
33 ☐	MA_M2M_0706_R	1446201_at	*6430519O13Rik*	0 day neonate eyeball cDNA, RIKEN full-length enriched library, clone:E130116B10 product:unknown EST, full insert sequence.	Chr9:9.220662	7.465	45	12.2	Chr1:62.725845	-0.169
34 ☐	MA_M2M_0706_R	1424098_at	*Elovl7*	ELOVL family member 7, elongation of long chain fatty acids (yeast); mid 3' UTR	Chr13:108.283633	8.726	45	11.8	Chr5:24.371012	0.203
35 ☐	MA_M2M_0706_R	1458028_at	*LOC231291*	hypothetical protein LOC231291	Chr11:78.846291	8.093	45	11.9	Chr6:125.820102	0.077

36 ☐	MA_M2M_0706_R	1445669_at	*Spry4*	sprouty homolog 4 (Drosophila)	Chr18: 38.586291	6.072	45	6.5	Chr9: 29.939029	-0.054
37 ☐	MA_M2M_0706_R	1456340_at	*2610205E22Rik*	RIKEN cDNA 2610205E22 gene	Chr2: 30.817111	8.020	45	11.7	Chr2: 78.100320	0.207
38 ☐	MA_M2M_0706_R	1449439_at	*Klf7*	Kruppel-like factor 7 (ubiquitous); far 3' UTR	Chr1: 64.032879	7.895	45	12.1	Chr5: 131.541036	-0.136
39 ☐	MA_M2M_0706_R	1445144_at	*A630075K04Rik*	RIKEN cDNA A630075K04 (similar to putative histidine kinase)	Chr15: 55.840826	7.347	45	17.2	Chr6: 43.826811	0.052
40 ☐	MA_M2M_0706_R	1426586_at	*Slc25a11*	solute carrier family 25 (mitochondrial carrier; oxoglutarate carrier), member 11; exons 5,6, and 8, and proximal 3' UTR	Chr11: 70.644618	13.209	45	13.3	Chr2: 104.427306	-0.113
41 ☐	MA_M2M_0706_R	1450650_at	*Myo10*	myosin X (conventional non-muscle myosin heavy chain IIB, May-Hegglin anomaly); last three exons and 3' UTR	Chr15: 25.808154	9.524	45	10.2	Chr1: 37.639250	-0.209
42 ☐	MA_M2M_0706_R	1430820_a_at	*Bbx*	bobby sox HMG-BOX transcription factor; exons 6, 7, and 8	Chr16: 50.224892	7.643	45	11.2	Chr5: 25.445855	0.271
43 ☐	MA_M2M_0706_R	1437862_at	*Rbm25*	RNA binding motif protein 25; distal 3' UTR of short form message	Chr12: 83.663292	9.099	45	16.1	Chr7: 6.024860	0.409
44 ☐	MA_M2M_0706_R	1425836_a_at	*Limk1*	LIM-domain containing, protein kinase; mid 3' UTR (putative miR-134 target)	Chr5: 134.656678	9.628	45	11.2	Chr2: 80.327515	-0.130
45 ☐	MA_M2M_0706_R	1436983_at	*Crebbp*	CREB binding protein; two exons toward 3' end of gene	Chr16: 4.093487	8.419	45	11.1	Chr2: 78.100320	0.285
46 ☐	MA_M2M_0706_R	1441100_at	*Mbtd1*	mbt domain containing 1	Chr11: 93.912698	7.041	45	8.6	Chr4: 121.044402	-0.174
47 ☐	MA_M2M_0706_R	1418024_at	*Narg1*	NMDA receptor-regulated gene 1; four central exons	Chr3: 51.458625	8.303	45	13.8	Chr2: 70.716531	0.283

48 ☐	MA_M2M_0706_R	1443053_at	*Ptprd*	protein tyrosine phosphatase, receptor type, D; intron 1 (from EST AK053007)	Chr4: 76.435514	7.633	45	33.9	Chr4: 75.840751	-0.372
49 ☐	MA_M2M_0706_R	1439832_at	*Wasl*	Wiskott-Aldrich syndrome-like (human)	Chr6: 24.621920	7.056	45	9.6	Chr2: 178.717318	0.142
50 ☐	MA_M2M_0706_R	1430542_a_at	*Slc25a5*	solute carrier family 25 (mitochondrial carrier, adenine nucleotide translocator), member 5	ChrX: 36.798388	16.091	45	10.0	Chr7: 71.696966	0.089
51 ☐	MA_M2M_0706_R	1423137_at	*Rala*	v-ral simian leukemia viral oncogene homolog A (ras related); first three exons	Chr13: 17.888504	9.009	45	11.5	Chr3: 57.673432	-0.262
52 ☐	MA_M2M_0706_R	1449292_at	*Rb1cc1*	RB1-inducible coiled-coil 1; exon 13	Chr1: 6.248798	7.287	45	7.9	Chr7: 6.024860	0.269
53 ☐	MA_M2M_0706_R	1440083_at	*Cdc42*	cell division cycle 42 (activator of Rac); intron 1	Chr4: 137.336477	7.239	45	10.4	Chr2: 70.495989	0.102
54 ☐	MA_M2M_0706_R	1450054_at	*Add1*	adducin 1 (alpha, calmodulin-binding, spectrin-actin network assembly protein); distal 3' UTR (long variant 3' UTR)	Chr5: 34.631716	12.257	45	13.4	Chr5: 139.505838	0.081
55 ☐	MA_M2M_0706_R	1435899_at	*9430079B08Rik*	RIKEN cDNA 9430079B08 gene	Chr1: 106.721229	7.541	45	8.6	Chr6: 51.246390	0.115
56 ☐	MA_M2M_0706_R	1448803_at	*Golga4*	golgi autoantigen, golgin subfamily a, 4	Chr9: 118.556220	8.990	45	10.5	Chr7: 6.024860	0.285
57 ☐	MA_M2M_0706_R	1448996_at	*Rom1*	rod outer segment membrane protein 1 (retinitis pigmentosa, peripherin-related, digenic)	Chr19: 8.927670	7.606	45	11.8	Chr12: 46.420986	-0.069
58 ☐	MA_M2M_0706_R	1425565_at	*Rest*	RE1-silencing transcription factor; last exon (5' end)	Chr5: 77.280782	9.446	45	8.4	Chr2: 18.263847	-0.156
59 ☐	MA_M2M_0706_R	1437457_a_at	*Mtpn*	myotrophin; mid-distal 3' UTR	Chr6: 35.509968	9.714	45	7.3	Chr5: 25.445855	0.215

60 ☐	MA_M2M_0706_R	1454287_at	*4931428A05Rik*	RIKEN cDNA 4931428A05 gene	Chr1: 90.758673	7.086	45	10.5	Chr1: 3.010274	0.121
61 ☐	MA_M2M_0706_R	1417000_at	*Abtb1*	ankyrin repeat and BTB (POZ) domain containing 1	Chr6: 88.836073	8.863	45	7.9	Chr11: 120.95690 0	-0.115
62 ☐	MA_M2M_0706_R	1416309_at	*Nusap1*	nucleolar and spindle associated protein 1	Chr2: 119.649197	6.993	45	15.9	Chr9: 107.65060 4	0.133
63 ☐	MA_M2M_0706_R	1436982_at	*AI848765*	expressed sequence AI848765	Chr15: 80.940450	8.744	45	9.3	Chr3: 53.053282	-0.197
64 ☐	MA_M2M_0706_R	1418414_at	*Kcnh1*	potassium voltage-gated channel, subfamily H (eag-related), member 1	Chr1: 192.506537	7.515	45	8.5	Chr14: 42.240278	0.093
65 ☐	MA_M2M_0706_R	1448883_at	*Lgmn*	legumain cysteine protease; last two exons and proximal 3' UTR	Chr12: 102.394497	13.431	45	10.4	Chr15: 91.405127	0.186
66 ☐	MA_M2M_0706_R	1436875_at	*Dnm3*	dynamin 3; mid 3' UTR	Chr1: 161.990252	5.813	45	22.6	Chr2: 70.716531	0.098
67 ☐	MA_M2M_0706_R	1456822_at	*Rad23b*	RAD23b homolog (S. cerevisiae)	Chr4: 55.368023	5.938	45	12.2	Chr2: 69.724603	0.087
68 ☐	MA_M2M_0706_R	1420384_at	*Col4a3bp*	procollagen, type IV, alpha 3 (Goodpasture antigen) binding protein	Chr13: 96.638320	7.742	45	12.0	Chr5: 25.445855	0.228
69 ☐	MA_M2M_0706_R	1446330_at	*BC042698*	cDNA sequence BC042698 in DENN/MADD domain containing 1B; from EST AK031618 in Dennd1b 5' UTR or promoter	Chr1: 138.971274	6.366	45	10.5	Chr7: 89.835648	-0.073
70 ☐	MA_M2M_0706_R	1438634_x_at	*Lasp1*	LIM and SH3 protein 1; distal 3' UTR	Chr11: 97.838653	11.906	45	10.7	Chr2: 113.45288	0.199
71 ☐	MA_M2M_0706_R	1445481_at	*Slc8a1*	solute carrier family 8 (sodium/calcium exchanger), member 1	Chr17: 81.515399	7.508	45	12.7	Chr2: 107.153805	0.231
72 ☐	MA_M2M_0706_R	1443926_at	*Mrg1*	myeloid ecotropic viral integration	Chr2: 116.046304	6.209	45	13.4	Chr14:	-0.106

				site-related gene 1; intron 7					78.383350	
73 ☐	MA_M2M_0706_R	1457505_at	*Utrn*	utrophin; first intron	Chr10:12.788878	6.531	45	10.5	Chr9:118.00000000	-0.064
74 ☐	MA_M2M_0706_R	1444563_at	*AW494124*	ESTs	Chr2:167.348354	8.082	45	12.3	Chr2:80.327515	-0.125
75 ☐	MA_M2M_0706_R	1457233_at	*Dnaja2*	DnaJ (Hsp40) homolog, subfamily A, member 2	Chr8:85.554238	8.731	45	17.9	Chr8:83.183208	-0.170
76 ☐	MA_M2M_0706_R	1440013_at	*Trim44*	tripartite motif-containing 44	Chr2:102.326920	7.396	45	11.1	Chr2:169.891672	0.114
77 ☐	MA_M2M_0706_R	1442381_at	*Mkln1*	muskelin 1, intracellular mediator containing kelch motifs; last intron	Chr6:31.505617	6.179	45	14.7	Chr11:48.117381	0.071
78 ☐	MA_M2M_0706_R	1423939_a_at	*Yif1*	Yip1 interacting factor homolog (S. cerevisiae)	Chr19:5.092408	12.686	45	10.8	Chr2:78.100320	-0.107
79 ☐	MA_M2M_0706_R	1423714_at	*Asf1b*	ASF1 anti-silencing function 1 homolog B (histone chaperone); 3' UTR	Chr8:83.969636	8.022	45	11.1	ChrX:115.338127	0.073
80 ☐	MA_M2M_0706_R	1429764_at	*Fam101b*	family with sequence similarity 101 member B (regulator of filamin protein B)	Chr11:76.021768	10.851	45	10.7	Chr17:16.345499	-0.226
81 ☐	MA_M2M_0706_R	1455463_at	*Phyhip*	phytanoyl-CoA hydroxylase interacting protein; distal 3' UTR	Chr14:70.468254	7.422	45	9.1	Chr5:139.505838	0.176
82 ☐	MA_M2M_0706_R	1424013_at	*Etf1*	eukaryotic translation termination factor 1; far 3' UTR	Chr18:34.902978	10.839	45	13.8	Chr2:100.989316	-0.089
83 ☐	MA_M2M_0706_R	1437577_at	*Bicd1*	bicaudal D homolog 1 (Drosophila)	Chr11:23.342425	7.399	45	10.2	Chr14:39.833599	-0.138
84 ☐	MA_M2M_0706_R	1454608_x_at	*Ttr*	transthyretin (prealbumin); proximal 3' UTR	Chr18:20.673768	11.921	45	46.6	Chr18:16.414681	-3.283
85 ☐	MA_M2M_0706_R	1452392_a_at	*Wipi1*	WD repeat domain phosphorinositide-interacting protein 1	Chr11:109.576654	7.217	45	10.3	Chr5:131.5	-0.099

									41036	
86 ☐	MA_M2M_0706_R	1420583_a_at	*Rora*	retinoic acid receptor-related orphan receptor alpha; last four exons and proximal 3' UTR	Chr9: 69.374033	9.232	45	13.6	Chr4: 96.896949	-0.202
87 ☐	MA_M2M_0706_R	1415990_at	*Vdac2*	voltage-dependent anion channel 2; exons 8 and 10 and 3' UTR	Chr14: 21.840474	14.070	45	14.1	Chr4: 142.912003	-0.122
88 ☐	MA_M2M_0706_R	1444175_at	*Arfgef1*	ADP-ribosylation factor guanine nucleotide-exchange factor 1(brefeldin A-inhibited); intron (SNP-free)	Chr1: 10.197091	6.092	45	10.8	Chr2: 166.630386	0.076
89 ☐	MA_M2M_0706_R	1426485_at	*Ubxd2*	UBX domain containing 2	Chr1: 128.258830	10.472	45	12.3	Chr2: 145.281646	0.237
90 ☐	MA_M2M_0706_R	1455998_at	*G630041M05Rik*	RIKEN cDNA G630041M05 gene	Chr1: 133.659801	7.579	45	11.5	Chr17: 78.355391	-0.105
91 ☐	MA_M2M_0706_R	1425523_at	*Rbm25*	RNA binding motif protein 25; exons 1, 2, 3, 4, and 5	Chr12: 83.644398	9.929	45	8.8	Chr2: 159.542988	0.208
92 ☐	MA_M2M_0706_R	1456357_at	*Scai*	Scai suppressor of cancer cell invasion; mid 3' UTR (long UTR)	Chr2: 39.069183	8.707	45	12.0	Chr2: 145.281646	0.198
93 ☐	MA_M2M_0706_R	1421508_at	*Odz1*	odd Oz/ten-m homolog 1	ChrX: 42.532423	6.489	45	17.2	Chr6: 51.246390	0.085
94 ☐	MA_M2M_0706_R	1426697_a_at	*Lrpap1*	low density lipoprotein receptor-related protein associated protein 1; distal 3'UTR	Chr5: 35.091708	12.273	45	13.9	Chr2: 80.327515	-0.154
95 ☐	MA_M2M_0706_R	1454338_at	*E130112L15Rik*	RIKEN cDNA E130112L15 gene	Chr1: 162.713021	7.070	45	13.3	Chr2: 75.132893	0.113
96 ☐	MA_M2M_0706_R	1448842_at	*Cdo1*	cysteine dioxygenase 1, cytosolic	Chr18: 46.713542	12.520	45	19.8	Chr18: 46.275839	0.240
97 ☐	MA_M2M_0706_R	1423659_a_at	*Tbc1d17*	TBC1 domain family, member 17; 3' UTR or last 3 exons	Chr7: 44.841352	9.173	45	17.6	Chr3: 54.220900	0.119

98 ☐	MA_M2M_0706_R	1446811_at	*9430023B20Rik*	RIKEN cDNA 9430023B20 gene	Chr18: 6.964283	6.357	45	8.3	Chr5: 131.54103 6	0.052
99 ☐	MA_M2M_0706_R	1453841_at	*Laf4*	lymphoid nuclear protein related to AF4	Chr1: 38.55428 5	5.980	45	10.6	Chr6: 43.82 6811	0.056
100 ☐	MA_M2M_0706_R	1439304_at	*Pam*	peptidylglycine alpha-amidating monooxygenase; intron 1	Chr1: 98.04582 9	7.052	45	8.7	Chr4: 156.12174 7	0.142
101 ☐	MA_M2M_0706_R	1433862_at	*Espl1*	extra spindle poles-like 1 (S. cerevisiae)	Chr15: 102.324051	6.435	45	9.1	Chr3: 69.02 5720	0.055
102 ☐	MA_M2M_0706_R	1436076_at	*Dlgap1*	discs, large (Drosophila) homolog-associated protein 1; far 3' UTR	Chr17: 70.82083 2	5.615	45	13.6	Chr2: 70.49 5989	0.029
103 ☐	MA_M2M_0706_R	1434637_x_at	*Sin3b*	transcriptional regulator, SIN3B (yeast)	Chr8: 72.73344 6	10.680	45	14.6	Chr6: 133.93667 0	0.128
104 ☐	MA_M2M_0706_R	1453773_at	*4931406I20Rik*	RIKEN cDNA 4931406I20 gene	Chr4: 117.285261	6.650	45	10.3	Chr2: 70.61 3187	-0.074
105 ☐	MA_M2M_0706_R	1455913_x_at	*Ttr*	transthyretin (prealbumin); proximal 3' UTR	Chr18: 20.67378 3	11.079	45	48.6	Chr18: 16.41 4681	-3.471
106 ☐	MA_M2M_0706_R	1456159_at	*2900045N06Rik*	RIKEN cDNA 2900045N06; intron (from EST AK042057)	Chr6: 113.131441	6.926	45	11.9	Chr11: 50.38 3261	0.082
107 ☐	MA_M2M_0706_R	1452270_s_at	*Cubn*	cubilin (intrinsic factor-cobalamin receptor)	Chr2: 13.27637 7	14.228	45	17.2	ChrX: 102.12665 6	0.186
108 ☐	MA_M2M_0706_R	1434801_x_at	*Slc25a5*	solute carrier family 25 (mitochondrial carrier, adenine nucleotide translocator), member 5; last two exons and 3' UTR	ChrX: 36.79778 2	15.746	45	11.8	Chr17: 64.99 3854	-0.097
109 ☐	MA_M2M_0706_R	1456677_at	*Herc4*	hect domain and RLD 4	Chr10: 63.24721 1	6.339	45	10.1	Chr3: 151.38260 1	0.066
110 ☐	MA_M2M_0706_R	1425156_at	*Gbp7*	guanylate binding protein 7	Chr3: 142.546476	8.022	45	13.7	Chr3: 139.35433 0	0.151

111	MA_M2M_0706_R	1450324_a_at	*4930521E07Rik*	RIKEN cDNA 4930521E07 gene	Chr19:40.605396	7.354	45	10.2	Chr12:37.639776	-0.100
112	MA_M2M_0706_R	1430606_at	*2310020F24Rik*	RIKEN cDNA 2310020F24 gene	Chr6:38.626778	8.797	45	13.9	Chr1:62.609193	0.112
113	MA_M2M_0706_R	1442935_at	*C81452*	expressed sequence C81452	Chr18:68.865805	6.999	45	15.0	Chr16:44.143640	0.079
114	MA_M2M_0706_R	1416360_at	*Snag1*	sorting nexin associated golgi protein 1 (intracellular trafficking); proximal to mid 3' UTR	Chr13:113.593598	9.791	45	8.3	Chr1:12.938444	-0.127
115	MA_M2M_0706_R	1456831_at	*Arid5b*	AT rich interactive domain 5B (Mrf1 like)	Chr10:68.152649	7.337	45	9.9	Chr2:70.716531	0.092
116	MA_M2M_0706_R	1436752_at	*Tbccd1*	TBCC domain containing 1; 3' UTR	Chr16:22.813672	8.385	45	13.2	Chr6:16.056743	-0.110
117	MA_M2M_0706_R	1454551_at	*9530034D02Rik*	RIKEN cDNA 9530034D02 gene	Chr6:31.331295	6.427	45	10.8	Chr6:124.528197	-0.058
118	MA_M2M_0706_R	1446692_at	*BM115381*	ESTs, Highly similar to S12207 hypothetical protein (B2 element) - mouse [M.musculus]	Chr11:77.696209	6.167	45	11.9	Chr6:9.348282	-0.074
119	MA_M2M_0706_R	1439816_at	*4930418G15Rik*	RIKEN cDNA 4930418G15 gene; far 3' UTR or unknown neighboring gene	Chr1:9.960201	7.671	45	47.9	Chr1:10.162992	-0.715
120	MA_M2M_0706_R	1442109_at	*Fubp1*	far upstream element (FUSE) binding protein 1	Chr3:152.230778	6.470	45	9.9	Chr7:19.429957	0.152
121	MA_M2M_0706_R	1432448_at	*2600006K01Rik*	RIKEN cDNA 2600006K01 gene	Chr2:29.868759	6.842	45	8.0	Chr12:115.50550	0.069
122	MA_M2M_0706_R	1438265_at	*4930413O22Rik*	RIKEN cDNA 4930413O22 gene	Chr1:10.070038	7.737	45	9.4	Chr2:76.018672	0.180
123	MA_M2M_0706_R	1418432_at	*Cab39*	calcium binding protein 39	Chr1:85.849462	9.316	45	9.9	Chr2:70.716531	0.174

124 ☐	MA_M2M_0706_R	1460380_at	*Dsg2*	desmoglein 2 (calcium-binding transmembrane glycoprotein, arrhythmogenic right ventricular dysplasia, familial 10)	Chr18: 20.602254	7.394	45	8.0	Chr15: 102.32088 7	0.069
125 ☐	MA_M2M_0706_R	1431616_at	*Catsperg2*	catsper channel auxiliary subunit gamma 2	Chr7: 29.723821	6.441	45	11.8	Chr12: 37.639776	-0.074
126 ☐	MA_M2M_0706_R	1451494_at	*Wac*	WW domain containing adaptor with coiled-coil; exons 12 and 14, and proximal 3' UTR	Chr18: 7.926111	10.545	45	11.2	Chr18: 61.000000	-0.172
127 ☐	MA_M2M_0706_R	1458239_at	*E2f1*	adult male testis cDNA, RIKEN full-length enriched library, clone:4930408L20 product:unclassifiable, full insert sequence.	Chr7: 24.131364	6.375	45	12.3	Chr10: 110.41669 5	-0.093
128 ☐	MA_M2M_0706_R	1446556_at	*Kcnd1*	potassium voltage-gated channel, Shal-related family, member 1	ChrX: 7.837757	7.036	45	11.2	Chr6: 43.826811	0.080
129 ☐	MA_M2M_0706_R	1444852_at	*Fmo4*	flavin containing monooxygenase 4; antisense in 5' UTR	Chr1: 162.812624	6.618	45	17.0	Chr2: 79.343444	-0.056
130 ☐	MA_M2M_0706_R	1444660_at	*Kiaa1109*	transmembrane protein KIAA1109; intronic or AK085090	Chr3: 37.023574	7.665	45	13.8	Chr7: 107.29397 4	-0.159
131 ☐	MA_M2M_0706_R	1420326_s_at	*Cramp1l*	Crm, cramped-like; putative far 3' UTR (from Homo CRAMP1L)	Chr17: 24.961389	8.842	45	11.3	Chr6: 124.52819 7	-0.124
132 ☐	MA_M2M_0706_R	1455122_at	*Ptchd2*	patched domain containing 2; distal 3' UTR	Chr4: 148.240780	7.440	45	9.6	Chr6: 125.82010 2	0.106
133 ☐	MA_M2M_0706_R	1434088_at	*Zkscan17*	zinc finger with KRAB and SCAN domains 17; distal 3' UTR	Chr11: 59.485589	9.209	45	15.2	Chr1: 17.259053	0.102
134 ☐	MA_M2M_0706_R	1448856_a_at	*Msra*	methionine sulfoxide reductase A; 3' UTR	Chr14: 64.122780	13.992	45	13.2	Chr15: 93.021696	0.125

135 ☐	MA_M2M_0706_R	1423772_x_at	*Slc25a5*	solute carrier family 25 (mitochondrial carrier, adenine nucleotide translocator), member 5; last two exons and 3' UTR	ChrX: 36.797736	16.017	45	12.0	Chr14: 39.833599	0.090
136 ☐	MA_M2M_0706_R	1427309_at	*Pars2*	prolyl-tRNA synthetase (mitochondrial); 3' UTR	Chr4: 106.654706	8.910	45	12.3	Chr5: 21.026344	-0.110
137 ☐	MA_M2M_0706_R	1456898_at	*Pura*	purine rich element binding protein A; far 3' UTR element associated with Pura	Chr18: 36.292229	10.045	45	9.8	Chr2: 11.785864	-0.197
138 ☐	MA_M2M_0706_R	1452094_at	*P4ha1*	procollagen-proline, 2-oxoglutarate 4-dioxygenase (proline 4-hydroxylase), alpha 1 polypeptide	Chr10: 59.371376	8.772	45	11.6	Chr2: 131.056131	0.185
139 ☐	MA_M2M_0706_R	1451485_at	*Luc7l3*	LUC7-like 3 (cAMP regulatory element-associated protein 1); exons 4, 5, and 7	Chr11: 94.297787	7.421	45	15.0	Chr2: 178.717318	0.227
140 ☐	MA_M2M_0706_R	1452734_at	*Rnaset2*	ribonuclease T2	Chr17: 6.988754	14.725	45	13.7	Chr17: 9.373070	0.112
141 ☐	MA_M2M_0706_R	1417131_at	*Cdc25a*	cell division cycle 25 homolog A (S. cerevisiae)	Chr9: 109.892195	7.953	45	12.3	Chr2: 70.495989	0.092
142 ☐	MA_M2M_0706_R	1457122_at	*Glmr*	ESTs	Chr2: 48.971554	5.635	45	11.0	Chr7: 6.024860	0.073
143 ☐	MA_M2M_0706_R	1436535_at	*Ssa2*	Sjogren syndrome antigen A2	Chr1: 143.752721	8.332	45	14.8	Chr2: 78.100320	0.272
144 ☐	MA_M2M_0706_R	1424089_a_at	*Tcf4*	transcription factor 4; last three exons and proximal 3' UTR	Chr18: 69.681618	7.593	45	12.9	Chr10: 115.872274	0.131
145 ☐	MA_M2M_0706_R	1423160_at	*Spred1*	sprouty protein with EVH-1 domain 1, related sequence	Chr2: 117.180081	10.174	45	12.1	Chr12: 38.632033	0.109
146 ☐	MA_M2M_0706_R	1425505_at	*Mylk*	myosin, light polypeptide kinase; last 3 exons	Chr16: 34.995087	8.931	45	16.5	Chr10: 111.073834	0.188

147 ☐	MA_M2M_0706_R	1441575_at	*A630049H14Rik*	12 days embryo male wolffian duct includes surrounding region cDNA, RIKEN full-length enriched library, clone:6720485J18 product:hypothetical protein, full insert sequence.	Chr10: 9.532566	6.187	45	7.0	Chr1: 98.793301	0.036
148 ☐	MA_M2M_0706_R	1440841_at	*Ywhae*	tyrosine 3-monooxygenase/tryptophan 5-monooxygenase activation protein, epsilon polypeptide	Chr11: 75.743295	8.181	45	7.8	Chr7: 104.149021	-0.171
149 ☐	MA_M2M_0706_R	1429505_at	*2310076G13Rik*	RIKEN cDNA 2310076G13 gene	Chr1: 60.336817	10.283	45	15.7	Chr2: 76.018672	0.293
150 ☐	MA_M2M_0706_R	1459218_at	*D330001F17Rik*	RIKEN cDNA D330001F17 gene	Chr15: 76.409024	6.705	45	11.1	Chr1: 62.609193	0.098
151 ☐	MA_M2M_0706_R	1429969_at	*Gimap5*	GTPase, IMAP family member 5	Chr6: 48.755881	6.371	45	15.8	Chr6: 9.348282	0.054
152 ☐	MA_M2M_0706_R	1425686_at	*Cflar*	CASP8 and FADD-like apoptosis regulator	Chr1: 58.754123	9.561	45	9.5	Chr2: 159.542988	0.297
153 ☐	MA_M2M_0706_R	1455560_at	*AK154427*	AK154427 thymus EST	Chr9: 22.085563	7.087	45	9.1	Chr3: 65.524002	0.100
154 ☐	MA_M2M_0706_R	1418331_at	*1110031I02Rik*	RIKEN cDNA 1110031I02 gene	Chr11: 121.177920	10.929	45	9.5	Chr1: 22.104486	0.094
155 ☐	MA_M2M_0706_R	1455580_at	*Usp6nl*	USP6 N-terminal like; distal 3' UTR	Chr2: 6.443279	9.892	45	10.5	Chr6: 6.060085	-0.094
156 ☐	MA_M2M_0706_R	1454178_at	*2610027F03Rik*	RIKEN cDNA 2610027F03 gene	Chr1: 120.688646	7.548	45	14.2	Chr5: 27.285288	-0.055
157 ☐	MA_M2M_0706_R	1438669_at	*Dcaf12*	DDB1 and CUL4 associated factor 12; mid-distal 3' UTR	Chr4: 41.291464	9.815	45	13.2	Chr2: 70.716531	0.142
158 ☐	MA_M2M_0706_R	1450359_at	*Fut1*	fucosyltransferase 1	Chr7: 45.619178	6.890	45	10.6	Chr5: 139.505838	0.109
159 ☐	MA_M2M_0706_R	1419572_a_at	*Abcd4*	ATP-binding cassette, sub-family D (ALD), member 4;	Chr12: 84.602794	8.811	45	9.5	Chr2: 69.342849	-0.138

				last two exons and 3' UTR						
160	MA_M2M_0706_R	1426777_a_at	*Wasl*	Wiskott-Aldrich syndrome-like (human)	Chr6: 24.637670	10.201	45	10.4	Chr2: 145.28164 6	0.256
161	MA_M2M_0706_R	1437091_at	*Accn4*	amiloride-sensitive cation channel 4, pituitary; 3' UTR	Chr1: 75.473751	7.866	45	9.5	Chr4: 121.04440 2	0.116
162	MA_M2M_0706_R	1457392_at	*Sfrs12*	splicing factor, arginine/serine-rich 12	Chr13: 103.740866	6.700	45	13.0	Chr15: 101.56102 3	-0.064
163	MA_M2M_0706_R	1423775_s_at	*Prc1*	protein regulator of cytokinesis 1	Chr7: 80.315784	7.776	45	9.4	Chr3: 65.524002	0.073
164	MA_M2M_0706_R	1447571_at	*Elk4*	ELK4, member of ETS oncogene family	Chr1: 132.026246	10.159	45	8.4	Chr2: 70.613187	-0.089
165	MA_M2M_0706_R	1455585_at	*Rnf168*	ring finger protein 168	Chr16: 32.300484	8.277	45	11.7	Chr5: 133.06278 3	-0.099
166	MA_M2M_0706_R	1417892_a_at	*Sirt3*	sirtuin 3 (NAD-dependent deacetylase); second to last exon and 3' UTR	Chr7: 140.863700	12.082	45	10.4	Chr15: 93.360915	0.139
167	MA_M2M_0706_R	1450968_at	*Uqcrfs1*	ubiquinol-cytochrome c reductase, Rieske iron-sulfur polypeptide 1; exon and 3'UTR	Chr13: 30.540651	15.420	45	7.5	Chr6: 124.52819 7	0.070
168	MA_M2M_0706_R	1449995_at	*4933421I07Rik*	RIKEN cDNA 4933421I07 gene	Chr7: 42.445522	7.164	45	8.4	Chr14: 39.724852	0.115
169	MA_M2M_0706_R	1457041_at	*1700026B20Rik*	small nucleolar RNA host gene (non-protein coding) 10 (similar to human small Cajal body-specific RNA 13_	Chr12: 105.030533	7.387	45	9.7	Chr1: 37.639250	0.093
170	MA_M2M_0706_R	1458385_at	*Hspa4l*	heat shock protein 4 like	Chr3: 40.791144	7.199	45	13.7	Chr2: 69.724603	0.166
171	MA_M2M_0706_R	1439890_at	*Fbxl7*	F-box and leucine-rich repeat protein 7	Chr15: 26.543518	7.782	45	9.5	Chr9: 29.939029	0.106

172 ☐	MA_M2M_0706_R	1459542_at	*Prkg1*	protein kinase, cGMP-dependent, type I; antisense in intron (poor probe set)	Chr19: 30.648761	5.800	45	12.8	Chr1: 3.010274	0.060
173 ☐	MA_M2M_0706_R	1424768_at	*Cald1*	caldesmon 1; mid 3' UTR	Chr6: 34.773553	9.057	45	11.7	Chr15: 91.405127	-0.395
174 ☐	MA_M2M_0706_R	1447195_at	*Elp4*	elongation protein 4 homolog (S. cerevisiae)	Chr2: 105.732360	6.640	45	6.9	Chr16: 44.143640	0.091
175 ☐	MA_M2M_0706_R	1456908_at	*BC023202*	clone IMAGE:3986405, mRNA	Chr12: 24.963801	8.152	45	15.1	Chr2: 159.542988	-0.084
176 ☐	MA_M2M_0706_R	1419529_at	*Il23a*	interleukin 23, alpha subunit p19	Chr10: 128.296260	6.355	45	9.5	Chr2: 18.263847	0.054
177 ☐	MA_M2M_0706_R	1415864_at	*Bpgm*	2,3-bisphosphoglycerate mutase; proximal 3' UTR (possible 3' UTR length variants)	Chr6: 34.504547	8.883	45	11.5	Chr14: 39.196761	-0.210
178 ☐	MA_M2M_0706_R	1453565_at	*Ndufab1*	NADH dehydrogenase (ubiquinone) 1, alpha/beta subcomplex, 1	Chr7: 122.086560	5.941	45	9.5	Chr2: 70.495989	0.102
179 ☐	MA_M2M_0706_R	1448021_at	*Fam46c*	family with sequence similarity 46, member C (protein LOC54855); far 3' UTR (putative)	Chr3: 100.468088	6.889	45	14.0	Chr17: 71.025688	0.201
180 ☐	MA_M2M_0706_R	1453176_a_at	*4933404M02Rik*	RIKEN cDNA 4933404M02 gene	Chr2: 70.531732	6.606	45	9.5	Chr5: 139.505838	0.094
181 ☐	MA_M2M_0706_R	1415814_at	*Atp6v1b2*	ATPase, H+ transporting, V1 subunit B, isoform 2; distal half of 3' UTR	Chr8: 69.113124	13.207	45	10.5	Chr15: 93.021696	0.121
182 ☐	MA_M2M_0706_R	1452378_at	*Ramp2*	receptor (calcitonin) activity modifying protein 2	Chr19: 5.800664	8.889	45	7.1	Chr2: 69.724603	0.276
183 ☐	MA_M2M_0706_R	1443212_at	*Large*	like-glycosyltransferase; intron 1	Chr8: 73.329777	6.474	45	17.2	Chr10: 116.134021	0.055

184	MA_M2M_0706_R	1425315_at	*Sox12*	SRY-box containing gene 12	Chr4: 98.992637	5.972	45	9.0	Chr2: 18.263847	-0.077
185	MA_M2M_0706_R	1439840_at	*Polb*	polymerase (DNA directed), beta	Chr8: 22.644913	6.740	45	13.5	Chr15: 93.360915	-0.174
186	MA_M2M_0706_R	1459888_at	*AI429562*	oviduct EST AK143260	Chr18: 61.647204	7.503	45	10.8	Chr1: 39.140420	0.101
187	MA_M2M_0706_R	1426817_at	*Mki67*	Mki67 protein (stem cell marker); last exon and proximal 3' UTR	Chr7: 135.689846	7.079	45	11.6	Chr2: 157.995222	-0.110
188	MA_M2M_0706_R	1444971_at	*Rbm5*	RNA binding motif protein 5 (renal carcinoma antigen NY-REN-9); rare transcript	Chr9: 107.764397	6.503	45	13.5	Chr4: 59.850763	-0.112
189	MA_M2M_0706_R	1446508_at	*D430017M14Rik*	13 days embryo male testis cDNA, RIKEN full-length enriched library, clone:6030447N08 product:unknown EST, full insert sequence.	Chr15: 68.343881	7.051	45	17.1	Chr4: 88.045738	-0.120
190	MA_M2M_0706_R	1444678_at	*2810403D21Rik*	RIKEN cDNA 2810403D21 gene	ChrX: 108.874391	5.599	45	14.1	Chr9: 105.774381	0.035
191	MA_M2M_0706_R	1439638_at	*Erbb2ip*	Erbb2 interacting protein	Chr13: 103.825620	6.490	45	8.9	Chr2: 78.100320	0.161
192	MA_M2M_0706_R	1458818_at	*D3Ertd162e*	DNA segment, Chr 3, ERATO Doi 162, expressed	Chr3: 132.353460	7.555	45	9.1	Chr5: 139.505838	0.098
193	MA_M2M_0706_R	1442322_at	*Slit2*	slit guidance ligand 2; intron	Chr5: 48.103982	7.037	45	12.7	Chr13: 103.151983	0.076
194	MA_M2M_0706_R	1456796_at	*Snai3*	snail homolog 3 (Drosophila)	Chr8: 122.454257	7.737	45	11.3	Chr2: 134.800968	-0.059
195	MA_M2M_0706_R	1445337_at	*Dnajc13*	DnaJ (Hsp40) homolog, subfamily C, member 13; three central exons	Chr9: 104.228418	8.083	45	12.2	Chr1: 12.938444	-0.245

196 ☐	MA_M2M_0706_R	1444004_at	*Thoc2*	THO complex 2 (TREX (transcription/export complex); three exons of a short form variant	ChrX: 41.827241	8.640	45	16.9	Chr7: 6.677334	0.428
197 ☐	MA_M2M_0706_R	1439095_at	*Sfrs11*	splicing factor, arginine/serine-rich 11	Chr3: 158.024742	8.110	45	8.5	Chr5: 21.748772	0.131
198 ☐	MA_M2M_0706_R	1460726_at	*Adss*	adenylosuccinate synthetase, non muscle	Chr1: 177.763572	11.601	45	12.8	Chr2: 15.000000	0.133
199 ☐	MA_M2M_0706_R	1444090_at	*4632424B03Rik*	RIKEN cDNA 4632423N09 gene	Chr17: 33.644988	7.559	45	8.3	Chr6: 125.820102	0.091
200 ☐	MA_M2M_0706_R	1442595_at	*5830405N20Rik*	RIKEN cDNA 5830405N20 gene	Chr7: 84.580663	6.321	45	13.6	Chr3: 53.269732	0.092
201 ☐	MA_M2M_0706_R	1460619_at	*4931419K03Rik*	RIKEN cDNA 4931419K03 gene	Chr1: 40.772316	8.816	45	10.6	ChrX: 50.932422	0.187
202 ☐	MA_M2M_0706_R	1431008_at	*H2-Q1*	histocompatibility 2, Q region locus 1	Chr17: 35.384916	10.084	45	21.0	Chr13: 110.449360	-0.462
203 ☐	MA_M2M_0706_R	1451449_at	*4933407N01Rik*	RIKEN cDNA 4933407N01 gene	Chr11: 30.931644	10.554	45	11.5	Chr7: 36.693524	-0.159
204 ☐	MA_M2M_0706_R	1450646_at	*Cyp51a1*	cytochrome P450, family 51, subfamily A, polypeptide 1 (endoplasmic reticulum, lanosterol 14-alpha demethylase); proximal 3' UTR	Chr5: 4.082591	11.949	45	14.1	Chr12: 84.895759	0.217
205 ☐	MA_M2M_0706_R	1427302_at	*Enpp3*	ectonucleotide pyrophosphatase/phosphodiesterase 3	Chr10: 24.774787	10.693	45	21.0	Chr8: 86.388770	0.168
206 ☐	MA_M2M_0706_R	1425776_a_at	*C87436*	expressed sequence C87436	Chr6: 86.469778	7.815	45	10.8	Chr9: 115.524788	0.097
207 ☐	MA_M2M_0706_R	1444826_at	*4933427D06*	ESTs	Chr7: 78.892287	6.043	45	10.5	Chr8: 127.830308	0.053
208 ☐	MA_M2M_0706_R	1429278_at	*2410170E07Rik*	RIKEN cDNA 2410170E07 gene	Chr7: 140.992613	10.465	45	8.2	Chr5: 130.3	0.097

									40522	
209	MA_M2M_0706_R	1437151_at	*Usp22*	ubiquitin specific protease 22	Chr11: 61.152155	10.093	45	16.3	Chr19: 28.403278	-0.126
210	MA_M2M_0706_R	1442766_at	*Ppp4r1*	ESTs	Chr17: 65.819859	7.900	45	7.7	Chr8: 123.289925	-0.078
211	MA_M2M_0706_R	1422919_at	*Hrasls*	HRAS-like suppressor	Chr16: 29.217674	6.315	45	8.7	ChrX: 57.768447	0.054
212	MA_M2M_0706_R	1458761_at	*Itpa*	inosine triphosphatase (nucleoside triphosphate pyrophosphatase)	Chr2: 130.665163	5.732	45	15.8	Chr15: 12.687693	-0.043
213	MA_M2M_0706_R	1439016_x_at	*Sprr2a*	small proline-rich protein 2A	Chr3: 92.219217	10.343	45	21.3	Chr4: 33.748049	-0.255
214	MA_M2M_0706_R	1451005_at	*Sumo1*	small ubiquitin-like modifier 1 (SMT3 suppressor of mif two 3 homolog 1); proximal and mid 3' UTR	Chr1: 59.639734	12.000	45	11.6	Chr5: 127.617133	0.112
215	MA_M2M_0706_R	1452237_at	*Hrb*	HIV-1 Rev binding protein; mid-distal 3' UTR	Chr1: 82.895811	7.792	45	11.6	Chr18: 80.000000	0.206
216	MA_M2M_0706_R	1443544_at	*C18orf1*	transmembrane neocortically enriched protein with low density lipoprotein receptor domain, human chromosome 18 open reading frame 1	Chr18: 67.977763	7.044	45	14.7	Chr1: 91.100378	-0.099
217	MA_M2M_0706_R	1443870_at	*Abcc4*	ATP-binding cassette, sub-family C (CFTR/MRP), member 4; distal 3' UTR	Chr14: 118.482729	8.698	45	11.2	Chr13: 50.000000	0.218
218	MA_M2M_0706_R	1449438_at	*Dpm1*	dolichol-phosphate (beta-D) mannosyltransferase 1	Chr2: 168.211418	11.496	45	11.1	Chr12: 107.547951	0.112
219	MA_M2M_0706_R	1420202_at	*S100a3*	Mus musculus transcribed sequence	--	5.577	45	12.5	Chr19:	-0.043

									27.53 1578	
220 ☐	MA_M2M _0706_R	14282 13_at	*2410003A1 4Rik*	RIKEN cDNA 2410003A14 gene	Chr7: 130.5335 76	11. 43 6	45	15.4	Chr4: 88.04 5738	0.1 14
221 ☐	MA_M2M _0706_R	14170 25_at	*H2-Eb1*	histocompatibility 2, class II antigen E beta	Chr17: 34.31435 7	10. 96 2	45	14.4	Chr8: 123.2 8992 5	- 0.2 79
222 ☐	MA_M2M _0706_R	14518 40_at	*Kcnip4*	Kv channel interacting protein 4 (A-type potassium channel modulatory protein 4)	Chr5: 48.39013 5	5.8 91	45	9.1	Chr11 : 100.4 1203 1	0.0 31
223 ☐	MA_M2M _0706_R	14306 17_at	*5730547N1 3Rik*	RIKEN cDNA 5730547N13 gene	Chr2: 119.6098 57	6.4 28	45	8.3	Chr9: 103.6 2546 7	0.0 34
224 ☐	MA_M2M _0706_R	14521 35_at	*Gpx6*	glutathione peroxidase 6	Chr13: 21.31901 1	10. 09 6	45	14.7	Chr3: 25.04 1091	- 0.2 20
225 ☐	MA_M2M _0706_R	14336 22_at	*Gemin4*	gem (nuclear organelle) associated protein 4	Chr11: 76.21087 8	7.8 66	45	10.1	Chr7: 3.078 244	- 0.0 97
226 ☐	MA_M2M _0706_R	14304 90_at	*Tcfap2a*	transcription factor AP-2, alpha; antisense in 5' UTR (from AK017409)	Chr13: 40.73460 9	6.1 73	45	8.8	Chr4: 88.80 7538	0.0 45
227 ☐	MA_M2M _0706_R	14488 60_at	*Rem2*	rad and gem related GTP binding protein 2; 3' UTR	Chr14: 54.47991 2	8.1 98	45	9.6	Chr16 : 10.59 5499	0.0 99
228 ☐	MA_M2M _0706_R	14428 97_at	*2610024E2 0Rik*	RIKEN cDNA 2610024E20 gene	Chr18: 34.49572 5	7.4 84	45	13.5	Chr19 : 32.73 8270	0.1 09
229 ☐	MA_M2M _0706_R	14430 42_at	*D130047N1 1Rik*	RIKEN cDNA D130047N11 gene	Chr16: 72.81761 9	6.7 95	45	11.7	Chr9: 95.32 9897	- 0.0 66
230 ☐	MA_M2M _0706_R	14328 66_at	*Rbbp5*	retinoblastoma binding protein 5	Chr1: 132.4764 81	6.1 12	45	9.5	Chr12 : 107.5 4795 1	0.0 49
231 ☐	MA_M2M _0706_R	14237 67_at	*Aip1*	A interacting protein 1; last three exons and proximal 3' UTR	Chr17: 74.29961 1	10. 83 8	45	11.3	Chr9: 107.6 3925 0	0.1 10
232 ☐	MA_M2M _0706_R	14179 35_at	*Mkrn2*	makorin, ring finger protein, 2	Chr6: 115.6180 89	9.4 96	45	9.9	Chr5: 117.7 8965 0	- 0.1 19

233	MA_M2M_0706_R	1435637_at	*Itfg1*	integrin alpha FG-GAP repeat containing 1 (T-cell immunomodulatory protein); distal 3' UTR	Chr8:85.718072	9.959	45	11.7	Chr15:25.732086	-0.248
234	MA_M2M_0706_R	1416845_at	*Tmem132a*	transmembrane protein 132A (heat shock protein 5 binding protein 1, GRP78-binding protein); last exon and 3' UTR	Chr19:10.857981	9.248	45	9.4	Chr3:27.192039	0.089
235	MA_M2M_0706_R	1444048_at	*AI666576*	ESTs	Chr9:22.440388	7.059	45	12.6	Chr13:110.449360	-0.064
236	MA_M2M_0706_R	1434665_at	*Aga*	aspartylglucosaminidase; exons 6, 7, and 8	Chr8:53.521136	10.866	45	10.4	Chr5:130.340522	0.154
237	MA_M2M_0706_R	1458209_at	*2410012C07Rik*	RIKEN cDNA 2410012C07 gene	Chr7:139.938644	6.871	45	13.4	Chr8:69.741264	-0.063
238	MA_M2M_0706_R	1459554_at	*Otoa*	ESTs, Weakly similar to L1 repeat, Tf subfamily, member 30 [] [M.musculus]	Chr11:41.313788	5.784	45	10.8	Chr13:105.184837	0.042
239	MA_M2M_0706_R	1421174_at	*Irf4*	interferon regulatory factor 4 (pigmentation)	Chr13:30.761404	6.694	45	11.1	Chr17:6.039016	0.037
240	MA_M2M_0706_R	1440006_at	*BC026600*	highly expressed non-coding pituitary and retinal gene BC026600; distal 3' end	Chr17:24.456563	6.108	45	19.9	Chr17:27.061005	0.068
241	MA_M2M_0706_R	1426290_at	*1500031M22Rik*	RIKEN cDNA 1500031M22 gene	Chr13:106.952343	7.086	45	11.0	Chr9:105.774381	0.058
242	MA_M2M_0706_R	1428334_at	*Ostm1*	osteopetrosis associated transmembrane protein 1; distal 3'UTR	Chr10:42.701869	10.087	45	15.7	Chr19:8.707699	0.170
243	MA_M2M_0706_R	1428812_at	*1700040L02Rik*	RIKEN cDNA 1700040L02 gene	Chr10:68.431234	7.569	45	12.7	Chr13:101.209232	0.151

244	MA_M2M_0706_R	1421595_at	*AB041544*	hypothetical protein, MNCb-2622; putative 3' UTR (from EST AB041544)	Chr17:83.574143	6.111	45	10.4	Chr8:117.545014	0.036
245	MA_M2M_0706_R	1430796_at	*Trim14*	tripartite motif-containing 14	Chr4:46.509689	5.999	45	10.5	Chr5:133.062783	0.032
246	MA_M2M_0706_R	1447224_at	*Edg5*	endothelial differentiation, sphingolipid G-protein-coupled receptor 5; putative far 3' UTR (possible neighbor gene)	Chr9:20.962394	6.096	45	10.5	Chr15:101.561023	0.040
247	MA_M2M_0706_R	1441784_at	*Sox14*	ESTs	Chr18:66.319272	5.971	45	8.6	Chr11:99.734855	-0.040
248	MA_M2M_0706_R	1429750_at	*Minpp1*	multiple inositol polyphosphate histidine phosphatase 1	Chr19:32.487637	6.243	45	13.7	Chr16:26.422169	0.073
249	MA_M2M_0706_R	1417492_at	*Ctsb*	cathepsin B (amyloid precursor protein alpha secretase); mid 3' UTR	Chr14:63.144180	9.001	45	12.1	Chr4:75.840751	0.242
250	MA_M2M_0706_R	1455980_a_at	*8430435B07Rik*	ESTs, Weakly similar to GAS2_MOUSE Growth-arrest-specific protein 2 (GAS-2) [M.musculus]	Chr10:89.412598	5.951	45	11.4	Chr18:82.255306	0.048
251	MA_M2M_0706_R	1442362_at	*Gm104*	predicted gene 104 downstream of Exo1; possible 3' UTR extension of Exo1	Chr1:175.921524	6.516	45	13.0	Chr5:127.781724	0.058
252	MA_M2M_0706_R	1453419_at	*Mras*	muscle and microspikes RAS; far 3' UTR	Chr9:99.385470	9.174	45	7.6	Chr15:22.860400	0.087
253	MA_M2M_0706_R	1425184_at	*2810433K01Rik*	RIKEN cDNA 2810433K01 gene	Chr18:74.195320	6.437	45	14.9	Chr14:81.073940	0.052
254	MA_M2M_0706_R	1438213_at	*A830018L16Rik*	RIKEN cDNA A830018L16 gene	Chr1:11.598186	6.194	45	13.5	Chr13:108.957958	0.051

255	MA_M2M_0706_R	1429390_at	*Acpl2*	acid phosphatase-like 2	Chr9: 96.823644	7.330	45	9.1	Chr18: 78.806816	0.124
256	MA_M2M_0706_R	1422391_at	*V1rc3*	vomeronasal 1 receptor, C3	Chr6: 57.137715	5.543	45	7.1	Chr16: 97.718129	-0.026
257	MA_M2M_0706_R	1416901_at	*Npc2*	Niemann Pick type C2; proximal 3' UTR	Chr12: 84.756822	12.119	45	11.1	ChrX: 114.159505	0.083
258	MA_M2M_0706_R	1446929_at	*Bach2*	BTB and CNC homology, basic leucine zipper transcription factor 2; intron	Chr4: 32.246045	6.747	45	15.2	Chr5: 131.541036	-0.061
259	MA_M2M_0706_R	1446558_at	*F730015K02Rik*	RIKEN cDNA F730015K02 gene	Chr9: 64.840586	5.732	45	12.5	Chr9: 99.750326	0.032
260	MA_M2M_0706_R	1428415_at	*2310020H19Rik*	RIKEN cDNA 2310020H19 gene	Chr12: 84.418169	7.386	45	8.5	Chr9: 21.613311	-0.103
261	MA_M2M_0706_R	1450561_a_at	*Surf1*	surfeit gene 1; last four exons (one alternative exon excluded)	Chr2: 26.913441	11.000	45	8.7	Chr13: 111.021029	0.071
262	MA_M2M_0706_R	1445038_at	*C730043O17*	hypothetical protein C730043O17	Chr18: 35.876721	7.222	45	13.9	ChrX: 9.334336	-0.047
263	MA_M2M_0706_R	1426291_at	*Dimt1*	DIM1 dimethyladenosine transferase 1-like; mid to distal 3' UTR	Chr13: 106.959073	6.754	45	9.5	ChrX: 3.231738	0.054
264	MA_M2M_0706_R	1446111_at	*D14Ertd611e*	DNA segment, Chr 14, ERATO Doi 611, expressed	Chr14: 93.948174	5.980	45	12.5	Chr5: 118.917989	-0.042
265	MA_M2M_0706_R	1415741_at	*Tmem165*	transmembrane protein 165; 3' UTR	Chr5: 76.208683	11.195	45	7.9	Chr5: 45.290189	0.128
266	MA_M2M_0706_R	1418146_a_at	*Rbl2*	retinoblastoma-like 2; proximal and mid 3' UTR	Chr8: 91.122460	9.162	45	13.4	Chr18: 76.826488	0.191
267	MA_M2M_0706_R	1417344_at	*C15orf24*	putative ATG/GTP binding protein (hyperthophic cardiomyopathy candidate gene); last three exons,	Chr2: 112.462915	13.144	45	12.3	Chr12: 48.965551	-0.086

				proximal and mid 3' UTR						
268 ☐	MA_M2M_0706_R	1431841_at	*4930434E21Rik*	RIKEN cDNA 4930434E21 gene	Chr5: 149.599205	7.581	45	10.0	Chr4: 156.10096 4	-0.086
269 ☐	MA_M2M_0706_R	1417366_s_at	*Calm1*	calmodulin 1; 5' half of exon 3	Chr12: 100.203574	12.116	45	12.0	Chr4: 75.840751	-0.180
270 ☐	MA_M2M_0706_R	1418536_at	*H2-Q7*	H-2 class I histocompatibility antigen, Q7 alpha Q7 to Q5	Chr17: 35.398424	8.385	45	51.3	Chr17: 33.146326	-0.631
271 ☐	MA_M2M_0706_R	1457806_at	*Dock1*	dedicator of cytokinesis 1; intron of Dock1 (and antisense in C10orf141 3' UTR)	Chr7: 134.883034	7.218	45	13.1	Chr9: 94.905865	-0.079
272 ☐	MA_M2M_0706_R	1443846_x_at	*Pank2*	pantothenate kinase 2 (Hallervorden-Spatz syndrome)	Chr2: 131.283272	7.941	45	8.3	Chr4: 88.045738	-0.102
273 ☐	MA_M2M_0706_R	1458240_at	*Baiap1*	brain-specific angiogenesis inhibitor 3	Chr6: 93.705640	5.992	45	10.2	ChrX: 146.829353	0.047
274 ☐	MA_M2M_0706_R	1455013_at	*Arih2*	ariadne RBR E3 ubiquitin protein ligase 2	Chr9: 108.603136	9.527	45	14.8	Chr9: 107.831131	0.121
275 ☐	MA_M2M_0706_R	1419930_at	*D15Ertd55e*	DNA segment, Chr 15, ERATO Doi 55, expressed	Chr15: 88.711127	5.794	45	13.9	Chr17: 26.814001	0.033
276 ☐	MA_M2M_0706_R	1425868_at	*Hist2h2bb*	histone cluster 2, H2bb; far 3' UTR (long form)	Chr3: 96.278524	6.564	45	13.8	Chr18: 78.806816	0.058
277 ☐	MA_M2M_0706_R	1424652_at	*Fam176a*	family with sequence similarity 176, member A; mid 3' UTR	Chr6: 82.092516	10.637	45	13.8	Chr5: 69.357018	0.145
278 ☐	MA_M2M_0706_R	1457084_at	*Nalp9c*	NACHT, LRR and PYD containing protein 9c; last 3 exons	Chr7: 20.048808	7.457	45	9.6	Chr7: 24.937915	0.057
279 ☐	MA_M2M_0706_R	1418658_at	*Rmdn1*	regulator of microtubule dynamics 1; exons 4, 6, 7, 8, 9	Chr4: 19.588611	9.604	45	10.1	Chr1: 189.222535	-0.141
280 ☐	MA_M2M_0706_R	1416963_at	*Ubadc1*	ubiquitin associated domain containing 1	Chr2: 25.998617	10.703	45	16.0	Chr12:	0.138

									86.105698	
281 ☐	MA_M2M_0706_R	1436038_a_at	*Dscr5*	Down syndrome critical region homolog 5 (human)	Chr16:94.364735	11.777	45	14.2	Chr12:107.92347 2	0.128
282 ☐	MA_M2M_0706_R	1451440_at	*Chodl*	chondrolectin	Chr16:78.951211	6.772	45	10.1	Chr9:29.939029	0.083
283 ☐	MA_M2M_0706_R	1452191_at	*Prcp*	prolylcarboxypeptidase (angiotensinase C); last three coding exons	Chr7:92.927708	9.720	45	18.9	Chr3:27.192039	-0.260
284 ☐	MA_M2M_0706_R	1431297_a_at	*Crnde*	colorectal neoplasia differentially expressed (non-protein coding)	Chr8:92.326387	6.501	45	18.7	Chr18:78.806816	-0.079
285 ☐	MA_M2M_0706_R	1419820_at	*Pkhd1*	polycystic kidney and hepatic disease 1; 3' UTR	Chr1:20.057834	10.952	45	9.3	Chr16:49.849657	0.177
286 ☐	MA_M2M_0706_R	1443962_at	*Tfdp2*	transcription factor Dp 2	Chr9:96.310545	8.001	45	17.9	Chr9:103.625467	0.166
287 ☐	MA_M2M_0706_R	1416030_a_at	*Mcm7*	minichromosome maintenance deficient 7 (S. cerevisiae)	Chr5:138.165990	9.862	45	14.6	Chr12:9.742519	0.168
288 ☐	MA_M2M_0706_R	1434771_at	*0610011F06Rik*	RIKEN cDNA 0610011F06 gene	Chr17:25.876764	12.779	45	13.8	Chr18:15.638699	-0.117
289 ☐	MA_M2M_0706_R	1459724_at	*Zdhhc20*	zinc finger, DHHC domain containing 20; far 3' UTR	Chr14:57.831654	8.054	45	10.5	Chr8:127.830308	-0.085
290 ☐	MA_M2M_0706_R	1423527_at	*4921510H08Rik*	RIKEN cDNA 4921510H08 gene	Chr10:97.694208	5.788	45	11.1	Chr19:32.357503	-0.040
291 ☐	MA_M2M_0706_R	1423289_a_at	*1810029B16Rik*	RIKEN cDNA 1810029B16 gene	Chr8:66.476500	6.918	45	14.0	Chr18:82.106750	0.084
292 ☐	MA_M2M_0706_R	1423643_at	*Ddx39*	DEAD (Asp-Glu-Ala-Asp) box polypeptide 39	Chr8:83.722458	9.361	45	13.2	Chr15:7.167980	-0.174

293 ☐	MA_M2M_0706_R	1452937_s_at	*Ccdc28b*	coiled coil domain containing 28B; exon	Chr4: 129.620698	7.524	45	15.8	Chr19: 35.573941	-0.123
294 ☐	MA_M2M_0706_R	1448703_at	*Lsm8*	LSM8 homolog, U6 small nuclear RNA associated (S. cerevisiae)	Chr6: 18.851648	8.949	45	11.8	Chr19: 32.887053	-0.169
295 ☐	MA_M2M_0706_R	1422248_at	*Irs4*	insulin receptor substrate 4	ChrX: 141.721555	7.300	45	9.0	Chr4: 86.450967	-0.099
296 ☐	MA_M2M_0706_R	1457782_at	*Tln1*	talin 1; intron	Chr4: 43.552404	7.622	45	11.9	Chr13: 110.449360	-0.149
297 ☐	MA_M2M_0706_R	1422775_at	*Blk*	B lymphoid kinase (oncogene); last exon and proximal half of 3' UTR	Chr14: 63.373004	6.880	45	9.3	Chr9: 98.579310	-0.068
298 ☐	MA_M2M_0706_R	1433782_at	*Cldn12*	claudin 12; distal 3' UTR	Chr5: 5.505276	10.494	45	10.5	Chr16: 38.378085	-0.124
299 ☐	MA_M2M_0706_R	1458499_at	*Pde10a*	phosphodiesterase 10A; half distal 3' UTR	Chr17: 8.985059	6.988	45	12.8	Chr2: 169.897347	0.060
300 ☐	MA_M2M_0706_R	1460331_at	*Tm9sf2*	transmembrane 9 superfamily member 2 (putative endosomal transporter or channel, transferrin receptor associated); last five exons	Chr14: 122.150782	12.980	45	10.7	Chr19: 25.666449	-0.137
301 ☐	MA_M2M_0706_R	1450154_at	*Folh1*	folate hydrolase (glutamate carboxypeptidase 2); last three exons and proximal 3' UTR	Chr7: 86.719611	10.133	45	17.4	Chr7: 85.068913	0.415
302 ☐	MA_M2M_0706_R	1450411_at	*Fam122a*	RIKEN cDNA 2900009I07 gene	Chr19: 24.475866	5.781	45	10.4	Chr9: 105.774381	0.049
303 ☐	MA_M2M_0706_R	1452197_at	*Smc4*	structural maintenance of chromosomes 4; last three exons and proximal 3' UTR	Chr3: 69.033935	8.797	45	9.1	Chr9: 3.400000	-0.119

304 ☐	MA_M2M _0706_R	1430325_at	*Ubr1*	ubiquitin protein ligase E3 component n-recognin 1	Chr2: 120.863294	7.022	45	7.3	Chr19: 28.403278	0.065
305 ☐	MA_M2M _0706_R	1439762_x_at	*Adra2c*	adrenergic receptor, alpha 2c	Chr5: 35.281602	5.645	45	11.3	Chr16: 69.289612	-0.035
306 ☐	MA_M2M _0706_R	1447607_at	*Mif*	macrophage migration inhibitory factor (proinflammatory)	Chr10: 97.110884	8.739	45	7.9	Chr15: 10.000000	0.072
307 ☐	MA_M2M _0706_R	1428845_at	*Bclaf1*	BCL2-associated transcription factor 1; distal 3' UTR (transQTL on Chr 4 in BXD eye data)	Chr10: 20.341571	9.897	45	10.4	Chr7: 73.746984	0.183
308 ☐	MA_M2M _0706_R	1459012_at	*A730041O05Rik*	RIKEN cDNA A730041O05 gene	Chr4: 16.165078	5.675	45	8.2	Chr5: 133.062783	0.032
309 ☐	MA_M2M _0706_R	1451296_x_at	*Pabpc4*	poly A binding protein, cytoplasmic 4; last exon and 3' UTR	Chr4: 123.297871	9.987	45	12.6	Chr17: 21.686210	0.101
310 ☐	MA_M2M _0706_R	1450287_at	*Npas3*	neuronal PAS domain protein 3	Chr12: 54.069289	5.850	45	10.8	Chr8: 86.388770	0.034
311 ☐	MA_M2M _0706_R	1416787_at	*Acvr1*	activin A receptor, type 1; antisense in introns	Chr2: 58.479891	8.240	45	9.1	Chr4: 88.554042	-0.068
312 ☐	MA_M2M _0706_R	1458415_at	*Clec2e*	C-type lectin domain family 2, member e	Chr6: 146.783700	7.753	45	12.4	Chr7: 46.356949	0.090
313 ☐	MA_M2M _0706_R	1459849_x_at	*5730538E15Rik*	RIKEN cDNA 5730538E15 gene	Chr1: 9.720454	5.431	45	12.5	Chr5: 135.879195	0.028
314 ☐	MA_M2M _0706_R	1416319_at	*Adk*	adenosine kinase; proximal to mid 3' UTR	Chr14: 21.447972	12.030	45	13.5	Chr15: 102.320887	0.123
315 ☐	MA_M2M _0706_R	1431323_at	*Ccr9*	chemokine (C-C motif) receptor 9	Chr9: 123.695947	6.978	45	13.3	Chr4: 149.906312	-0.091
316 ☐	MA_M2M _0706_R	1453125_at	*Sox11*	SRY-box 11; mid 3' UTR	Chr12: 27.337337	5.757	45	12.8	Chr13: 103.949936	0.043

317	MA_M2M_0706_R	1436542_at	*Ptger1*	prostaglandin E receptor 1 (subtype EP1)	Chr8:83.666864	9.830	45	15.1	Chr8:80.868085	-0.144
318	MA_M2M_0706_R	1424157_at	*Ehd2*	EH-domain containing 2; mid-distal 3' UTR	Chr7:15.949247	8.934	45	14.8	Chr17:21.690414	-0.124
319	MA_M2M_0706_R	1416697_at	*Dpp4*	dipeptidylpeptidase 4; mid 3' UTR	Chr2:62.331853	10.157	45	9.9	Chr4:88.045738	0.176
320	MA_M2M_0706_R	1428164_at	*Nudt9*	nudix (nucleoside diphosphate linked moiety X)-type motif 9	Chr5:104.061732	10.994	45	10.7	Chr15:25.732086	-0.128
321	MA_M2M_0706_R	1427738_at	*D0Kist2*	DNA segment, KIST 2	Chr7:61.201731	5.790	45	8.2	Chr9:99.750326	0.029
322	MA_M2M_0706_R	1417594_at	*Gkap1*	G kinase anchoring protein 1; last 5 exons and 3' UTR	Chr13:58.233391	11.023	45	14.8	Chr4:62.707256	0.162
323	MA_M2M_0706_R	1424347_at	*Ppp6c*	protein phosphatase 6, catalytic subunit	Chr2:39.194994	11.305	45	7.7	Chr19:49.483195	-0.090
324	MA_M2M_0706_R	1431422_a_at	*Dusp14*	dual specificity phosphatase 14	Chr11:84.048492	9.171	45	15.4	Chr3:39.650830	-0.176
325	MA_M2M_0706_R	1433722_at	*5730522G15Rik*	RIKEN cDNA 5730522G15 gene	Chr7:75.754024	10.460	45	7.6	Chr4:40.731591	0.112
326	MA_M2M_0706_R	1439564_at	*1700090G07Rik*	RIKEN cDNA 1700090G07 gene	Chr17:86.917537	8.555	45	7.5	Chr9:64.513351	0.093
327	MA_M2M_0706_R	1444040_at	*Lair1*	leukocyte-associated Ig-like receptor 1	Chr7:4.007164	6.144	45	9.3	Chr9:29.940961	-0.052
328	MA_M2M_0706_R	1433846_s_at	*Fam175b*	abraxas brother 1, family with sequence similarity 175 member B; distal 3' UTR	Chr7:132.884531	10.297	45	10.0	Chr10:82.045440	-0.093
329	MA_M2M_0706_R	1455516_at	*Csrnp3*	cysteine/serine-rich nuclear protein 3; distal 3' UTR	Chr2:66.030963	5.936	45	10.3	Chr19:32.738270	-0.053
330	MA_M2M_0706_R	1452628_at	*Bag5*	BCL2-associated athanogene 5; exon and 3'UTR	Chr12:111.709888	9.104	45	22.8	Chr13:110.449360	0.118

331 ☐	MA_M2M_0706_R	1459286_at	*1700009F06Rik*	RIKEN cDNA 1700009F06 gene	Chr10: 9.695469	5.617	45		10.4	Chr5: 130.340522	0.027
332 ☐	MA_M2M_0706_R	1424607_a_at	*LOC432823*	endogenous retroviral sequence MGC37588; repetitive element	ChrUn: 1.000000	11.045	45		11.9	Chr3: 41.542098	0.269
333 ☐	MA_M2M_0706_R	1430268_at	*Trpm3*	transient receptor potential cation channel, subfamily M, member 3	Chr19: 22.554677	6.888	45		11.1	Chr13: 108.957958	-0.071
334 ☐	MA_M2M_0706_R	1442435_at	*AI317223*	expressed sequence AI317223	Chr11: 75.085192	8.790	45		11.0	Chr7: 6.011490	0.161
335 ☐	MA_M2M_0706_R	1445928_at	*March6*	membrane-associated ring finger (C3HC4) 6	Chr15: 31.484864	7.947	45		9.1	Chr5: 27.285288	0.089
336 ☐	MA_M2M_0706_R	1457443_at	*Gpatc2*	G patch domain containing 2	Chr1: 187.239847	5.972	45		10.0	Chr2: 79.343444	0.031
337 ☐	MA_M2M_0706_R	1428979_at	*Mtf1*	metal response element binding transcription factor 1; putative far 3' UTR	Chr4: 124.848813	8.171	45		12.0	Chr11: 21.343911	-0.129
338 ☐	MA_M2M_0706_R	1424940_s_at	*BC022687*	cDNA sequence BC022687	Chr12: 112.815781	8.967	45		10.3	Chr9: 110.638628	0.150
339 ☐	MA_M2M_0706_R	1442218_at	*Map3k9*	mitogen-activated protein kinase kinase kinase 9 (melanoma-associated); mid distal 3' UTR	Chr12: 81.717910	6.769	45		13.0	Chr13: 110.449360	0.050
340 ☐	MA_M2M_0706_R	1459546_s_at	*Enpp1*	ectonucleotide pyrophosphatase/phosphodiesterase 1; last exon and proximal 3' UTR	Chr10: 24.641520	8.278	45		15.5	Chr4: 74.391573	0.108
341 ☐	MA_M2M_0706_R	1424609_a_at	*LTR_Affy_1424609_a_at*	polymorphic long terminal repeat; poor probe specificity	Chr7: 111.565966	11.088	45		11.6	Chr5: 27.285288	0.246
342 ☐	MA_M2M_0706_R	1448060_at	*Sema6d*	sema domain, transmembrane domain (TM), and cytoplasmic domain, (semaphorin) 6D; proximal 3' UTR	Chr2: 124.665441	7.076	45		8.5	Chr6: 32.610603	-0.061

343 ☐	MA_M2M_0706_R	1444903_at	*March6*	membrane-associated ring finger (C3HC4) 6	Chr15: 31.519471	6.944	45	8.7	Chr13: 103.15198 3	-0.100
344 ☐	MA_M2M_0706_R	1418929_at	*Ift57*	intraflagellar transport 57 (estrogen-related receptor beta like 1); 4 exons and 3'UTR	Chr16: 49.763742	9.360	45	13.2	Chr9: 15.889229	-0.092
345 ☐	MA_M2M_0706_R	1443791_at	*4931406I20Rik*	RIKEN cDNA 4931406I20 gene	Chr9: 53.401100	5.609	45	10.3	Chr13: 103.15198 3	0.031
346 ☐	MA_M2M_0706_R	1455376_at	*Kiaa0564*	KIAA0564 putative ATP-binding protein; last exon and proximal 3' UTR (major BXD39 strain variant or KO)	Chr14: 79.201722	10.413	45	26.2	Chr14: 78.383350	0.217
347 ☐	MA_M2M_0706_R	1450192_at	*Lhcgr*	luteinizing hormone/choriogonadotropin receptor	Chr17: 88.741574	5.952	45	10.4	Chr4: 86.450967	-0.033
348 ☐	MA_M2M_0706_R	1427774_at	*Defb7*	defensin beta 7	Chr8: 19.497557	5.578	45	14.3	Chr15: 101.56102 3	0.037
349 ☐	MA_M2M_0706_R	1423460_at	*Perq1*	PERQ amino acid rich, with GYF domain 1	Chr5: 137.525506	9.329	45	17.4	Chr2: 159.54298 8	-0.134
350 ☐	MA_M2M_0706_R	1430794_at	*4933426K07Rik*	RIKEN cDNA 4933426K07 gene	Chr11: 57.655491	7.035	45	10.6	Chr16: 26.422169	0.070
351 ☐	MA_M2M_0706_R	1420112_at	*Pacs1*	phosphofurin acidic cluster sorting protein 1	Chr19: 5.263963	5.823	45	13.0	Chr4: 81.958676	0.045
352 ☐	MA_M2M_0706_R	1446052_at	*1700082M22Rik*	RIKEN cDNA 1700082M22 gene	ChrX: 79.408693	6.768	45	12.9	Chr1: 91.097803	-0.068
353 ☐	MA_M2M_0706_R	1418294_at	*Epb4.1l4b*	erythrocyte protein band 4.1-like 4b	Chr4: 57.061849	8.437	45	8.2	Chr15: 25.698695	-0.119
354 ☐	MA_M2M_0706_R	1452582_at	*Galm*	galactose mutarotase; mid 3' UTR	Chr17: 80.184508	12.125	45	12.1	ChrX: 114.15950 5	0.125

355 ☐	MA_M2M_0706_R	1418605_at	*Nr2c1*	nuclear receptor subfamily 2, group C, member 1; last exon and 3' UTR	Chr10: 94.195174	6.877	45	9.4	Chr18: 80.000000	0.080
356 ☐	MA_M2M_0706_R	1454647_at	*Nphp3*	nephronophthisis 3 (adolescent, nephronophthisis type 3, tapeto-retinal degeneration); mid 3' UTR (antisense in promoter of Ccrl1)	Chr9: 104.127062	13.266	45	12.4	Chr5: 130.340522	0.143
357 ☐	MA_M2M_0706_R	1450198_at	*Dusp13*	dual specificity phosphatase 13; last three exons	Chr14: 21.733706	8.079	45	12.0	Chr4: 81.958676	-0.077
358 ☐	MA_M2M_0706_R	1416029_at	*Tieg1*	TGFB inducible early growth response 1; distal 3' UTR	Chr15: 38.294544	7.459	45	9.6	Chr19: 44.599198	-0.172
359 ☐	MA_M2M_0706_R	1444135_at	*6332401O19Rik*	RIKEN cDNA 6332401O19 gene	Chr6: 24.952549	8.893	45	20.3	Chr19: 25.441462	0.088
360 ☐	MA_M2M_0706_R	1454423_at	*4930538D17Rik*	hypothetical protein FLJ22529; apparent unspliced intron	Chr19: 46.367329	10.454	45	10.5	Chr4: 88.045738	-0.136
361 ☐	MA_M2M_0706_R	1434132_at	*Spg8*	spastic paraplegia 8 (strumpellin); last three exons and proximal 3' UTR	Chr15: 59.332020	11.907	45	9.3	Chr4: 81.958676	0.086
362 ☐	MA_M2M_0706_R	1423764_s_at	*Mrpl37*	mitochondrial ribosomal protein L37; last three exons	Chr4: 107.057424	10.237	45	13.5	Chr8: 123.289925	0.097
363 ☐	MA_M2M_0706_R	1425336_x_at	*H2-K1*	histocompatibility 2, K1, K region (major histocompatibility complex, class I, C); last exon and 3' UTR	Chr17: 35.266843	13.512	45	18.0	Chr2: 134.462008	-0.321
364 ☐	MA_M2M_0706_R	1422650_a_at	*Riok3*	RIO kinase 3 (yeast)	Chr18: 12.152817	9.875	45	9.6	Chr18: 82.106750	0.139
365 ☐	MA_M2M_0706_R	1445251_at	*Tnfsf13b*	tumor necrosis factor (ligand) superfamily, member 13b (B-lymphocyte stimulator, Delta BAFF); far distal 3' UTR	Chr8: 10.037253	6.684	45	16.9	Chr8: 127.830308	-0.081

366 ☐	MA_M2M _0706_R	1421767_at	*Adk*	adenosine kinase; last two exons and proximal 3' UTR	Chr14: 21.423505	7.221	45	11.8	ChrX: 164.048002	0.067
367 ☐	MA_M2M _0706_R	1443735_at	*6720457D02Rik*	RIKEN cDNA 6720457D02 gene	--	5.637	45	9.0	Chr9: 103.625467	0.033
368 ☐	MA_M2M _0706_R	1420919_at	*Sgk3*	serum/glucocorticoid regulated kinase 3; distal 3' UTR	Chr1: 9.899981	9.925	45	11.7	Chr9: 98.579310	0.110
369 ☐	MA_M2M _0706_R	1449167_at	*Epb41l4a*	erythrocyte protein band 4.1-like 4a	Chr18: 33.797013	8.436	45	11.8	Chr18: 76.870082	0.158
370 ☐	MA_M2M _0706_R	1426190_at	*Aym1*	activator of yeast meiotic promoters 1	Chr5: 113.357415	8.553	45	10.2	Chr18: 78.806816	-0.070
371 ☐	MA_M2M _0706_R	1437492_at	*Mkx*	mohawk; distal 3' UTR	Chr18: 6.935145	5.903	45	10.7	Chr9: 85.995347	0.047
372 ☐	MA_M2M _0706_R	1448609_at	*Tst*	thiosulfate sulfurtransferase, mitochondrial; last 2 exons and 3' UTR	Chr15: 78.399601	12.303	45	12.0	Chr5: 118.917989	-0.158
373 ☐	MA_M2M _0706_R	1452134_at	*Tmem175*	transmembrane protein 175; last exon and proximal 3' UTR	Chr5: 108.646099	8.506	45	10.1	Chr18: 78.806816	0.118
374 ☐	MA_M2M _0706_R	1426453_at	*Pitrm1*	pitrilysin metalloprotease 1	Chr13: 6.578438	9.821	45	11.5	Chr1: 133.168278	-0.126
375 ☐	MA_M2M _0706_R	1418859_at	*Rfxap*	regulatory factor X-associated protein	Chr3: 54.804327	8.314	45	9.8	Chr2: 131.056131	0.137
376 ☐	MA_M2M _0706_R	1428439_at	*Nub1*	Nedd8 ultimate buster 1	Chr5: 24.710367	8.105	45	10.8	Chr5: 25.115971	-0.090
377 ☐	MA_M2M _0706_R	1416181_at	*Mesdc2*	mesoderm development candiate 2 (paraventricular medial hypothalamic signature); mid or distal 3' UTR	Chr7: 83.898706	11.345	45	9.3	Chr7: 89.123287	0.114
378 ☐	MA_M2M _0706_R	1428461_at	*Ppp2r5e*	protein phosphatase 2, regulatory subunit B (B56), epsilon isoform	Chr12: 75.452747	9.609	45	11.5	Chr2: 99.825314	-0.144

379 ☐	MA_M2M_0706_R	1424294_at	*Ppp4r1*	protein phosphatase 4, regulatory subunit 1	Chr17: 65.841282	10.290	45	10.6	Chr13: 108.95795 8	0.123
380 ☐	MA_M2M_0706_R	1423662_at	*Atp6ap2*	ATPase, H+ transporting, lysosomal accessory protein 2; mid 3' UTR	ChrX: 12.616164	13.176	45	9.9	Chr2: 107.15380 5	-0.122
381 ☐	MA_M2M_0706_R	1459406_at	*Cept1*	choline/ethanolaminephosphotransferase 1	Chr3: 106.536090	6.296	45	9.4	Chr2: 145.28164 6	0.048
382 ☐	MA_M2M_0706_R	1425496_at	*Abca3*	ATP-binding cassette, sub-family A (ABC1), member 3 (lipid organization and formation of lamellar bodies); last three exons and proximal 3' UTR	Chr17: 24.408551	11.751	45	12.9	Chr17: 31.632850	0.214
383 ☐	MA_M2M_0706_R	1456947_at	*Pafah1b1*	platelet-activating factor acetylhydrolase, isoform 1b, beta 1 subunit (Miller-Dieker lissencephaly); intron 1 (EST AK039862)	Chr11: 27.567873	7.439	45	12.2	Chr7: 89.123287	-0.124
384 ☐	MA_M2M_0706_R	1454342_at	*C030007D22Rik*	RIKEN cDNA C030007D22 gene	Chr4: 110.435012	5.608	45	9.7	Chr8: 11.279405	-0.029
385 ☐	MA_M2M_0706_R	1459271_at	*Cetn2*	centrin 2	ChrX: 72.911975	6.607	45	11.9	Chr18: 78.806816	-0.063
386 ☐	MA_M2M_0706_R	1423459_at	*Cops2*	COP9 (constitutive photomorphogenic) homolog, subunit 2; last exon and proximal 3' UTR	Chr2: 125.832025	10.402	45	23.6	Chr5: 130.34052 2	0.205
387 ☐	MA_M2M_0706_R	1457302_at	*Slc20a2*	solute carrier family 20, member 2; intron of Slc20a2, last exon of BC035532	Chr8: 22.482842	7.279	45	12.0	Chr8: 24.305481	0.125
388 ☐	MA_M2M_0706_R	1444081_at	*5033428A16Rik*	RIKEN cDNA 5033428A16 gene	Chr8: 88.127404	5.879	45	11.6	Chr1: 91.097803	0.035

389 ☐	MA_M2M_0706_R	1442072_at	*1110049L02Rik*	RIKEN cDNA 1110049L02 gene	Chr9: 56.203799	6.309	45	11.7	Chr16: 10.595499	-0.055
390 ☐	MA_M2M_0706_R	1415708_at	*Tug1*	taurine upregulated gene 1; distal 3' UTR	Chr11: 3.640063	10.813	45	10.3	Chr2: 173.24458	-0.121
391 ☐	MA_M2M_0706_R	1449047_at	*1600020H07Rik*	RIKEN cDNA 1600020H07 gene	Chr14: 31.615360	9.381	45	16.7	Chr7: 6.677334	0.378
392 ☐	MA_M2M_0706_R	1428803_at	*Acot6*	acyl-coenzyme A thioesterase 6	Chr12: 84.110781	7.725	45	9.7	ChrX: 37.500000	0.103
393 ☐	MA_M2M_0706_R	1429556_at	*Tead1*	TEA domain family member 1 (SV40 transcriptional enhancer factor, Sveinsson's chorioretinal atrophy); distal 3'UTR	Chr7: 112.905932	10.670	45	11.0	Chr15: 94.266750	-0.156
394 ☐	MA_M2M_0706_R	1430359_a_at	*9130012B15Rik*	RIKEN cDNA 9130012B15 gene	Chr7: 143.797823	9.595	45	9.3	Chr10: 27.031021	-0.084
395 ☐	MA_M2M_0706_R	1450777_at	*Xrn2*	5'-3' exoribonuclease 2; exons 22, 23, 24, and 25	Chr2: 147.061459	8.907	45	17.0	Chr15: 93.021696	0.137
396 ☐	MA_M2M_0706_R	1449067_at	*Slc2a2*	solute carrier family 2 (facilitated glucose transporter), member 2; distal 3' UTR	Chr3: 28.727844	13.426	45	13.1	Chr17: 21.686210	0.282
397 ☐	MA_M2M_0706_R	1456130_at	*Pcdh7*	protocadherin 7	Chr6: 12.311625	6.255	45	14.4	Chr15: 93.021696	-0.103
398 ☐	MA_M2M_0706_R	1460221_at	*Ptges3*	prostaglandin E synthase 3 (cytosolic); mid-proximal 3' UTR	Chr10: 128.076319	12.499	45	11.2	Chr15: 94.266750	-0.201
399 ☐	MA_M2M_0706_R	1418034_at	*Mrps9*	mitochondrial ribosomal protein S9	Chr1: 42.903338	8.318	45	11.1	Chr7: 24.937915	-0.138
400 ☐	MA_M2M_0706_R	1440037_at	*Pbx1*	pre B-cell leukemia transcription factor 1; 3' UTR of short form message (intron 2)	Chr1: 168.424573	10.578	45	13.7	Chr3: 27.192039	0.282

401 ☐	MA_M2M_0706_R	1451742_a_at	*Ugp2*	UDP-glucose pyrophosphorylase 2	Chr11: 21.323220	13.374	45	12.4	Chr6: 34.483299	0.146
402 ☐	MA_M2M_0706_R	1460165_at	*Ppp1ca*	protein phosphatase 1, catalytic subunit, alpha isoform; last exon and proximal half of 3' UTR	Chr19: 4.195010	13.664	45	10.0	Chr2: 75.132893	-0.103
403 ☐	MA_M2M_0706_R	1460575_at	*D3Ertd194e*	DNA segment, Chr 3, ERATO Doi 194, expressed	Chr3: 58.555538	8.811	45	12.5	Chr15: 95.949674	0.123
404 ☐	MA_M2M_0706_R	1423080_at	*Tomm20*	translocase of outer mitochondrial membrane 20	Chr8: 20.392462	13.652	45	14.3	Chr2: 78.100320	-0.110
405 ☐	MA_M2M_0706_R	1425933_a_at	*Nt5c2*	5'-nucleotidase, cytosolic II; exons 12, 13, 14, and 15	Chr19: 46.889826	9.237	45	12.0	Chr5: 93.156983	-0.266
406 ☐	MA_M2M_0706_R	1438718_at	*Fgf9*	fibroblast growth factor 9; distal half of 3' UTR	Chr14: 58.109862	7.584	45	9.4	ChrX: 102.126656	-0.129
407 ☐	MA_M2M_0706_R	1457073_at	*Ogt*	O-linked N-acetylglucosamine (GlcNAc) transferase (UDP-N-acetylglucosamine:polypeptide-N-acetylglucosaminyl transferase); intron 5	ChrX: 101.662586	8.687	45	11.6	ChrX: 167.394896	-0.139
408 ☐	MA_M2M_0706_R	1455979_at	*Arid1b*	AT rich interactive domain 1B (Swi1 like)	Chr17: 5.343789	9.384	45	9.8	Chr5: 97.810458	0.139
409 ☐	MA_M2M_0706_R	1437024_at	*4122402O22Rik*	RIKEN cDNA 4122402O22 gene	Chr16: 17.644384	6.339	45	9.3	Chr6: 25.228330	0.037
410 ☐	MA_M2M_0706_R	1450674_at	*Cdk5*	cyclin-dependent kinase 5; last five exons	Chr5: 24.419433	9.876	45	11.7	Chr5: 23.184735	-0.120
411 ☐	MA_M2M_0706_R	1453623_a_at	*Rad23a*	RAD23a homolog (S. cerevisiae)	Chr8: 84.840492	7.922	45	12.4	Chr8: 80.868085	0.239
412 ☐	MA_M2M_0706_R	1423809_at	*Tcf19*	transcription factor 19	Chr17: 35.512810	7.752	45	20.9	Chr9: 3.400000	-0.102
413 ☐	MA_M2M_0706_R	1451825_a_at	*Copz1*	coatomer protein complex, subunit zeta 1	Chr15: 103.299251	11.799	45	12.5	Chr12: 36.689834	0.086

414 ☐	MA_M2M_0706_R	1434748_at	*Ckap2*	cytoskeleton associated protein 2	Chr8: 22.168836	6.856	45	11.9	Chr15: 7.167980	-0.061
415 ☐	MA_M2M_0706_R	1434701_at	*Alkbh5*	alkB, alkylation repair homolog 5; distal 3' UTR	Chr11: 60.557920	12.396	45	9.9	Chr18: 82.106750	-0.119
416 ☐	MA_M2M_0706_R	1418517_at	*Irx3*	Iroquois related homeobox 3	Chr8: 91.798530	11.401	45	8.5	Chr18: 16.414681	-0.151
417 ☐	MA_M2M_0706_R	1429636_at	*1700010D01Rik*	RIKEN cDNA 1700010D01 gene	ChrX: 92.490095	6.768	45	6.7	Chr2: 165.782727	0.056
418 ☐	MA_M2M_0706_R	1415917_at	*Mthfd1*	methylenetetrahydrofolate dehydrogenase (NADP+ dependent), methenyltetrahydrofolate cyclohydrolase, formyltetrahydrofolate synthase; last 3 exons	Chr12: 76.314424	12.892	45	11.4	Chr7: 73.746984	0.098
419 ☐	MA_M2M_0706_R	1449481_at	*Slc25a13*	solute carrier family 25 (mitochondrial carrier, adenine nucleotide translocator), member 13 (citrin deficiency associated)	Chr6: 6.041795	11.836	45	18.1	Chr6: 23.242013	0.192
420 ☐	MA_M2M_0706_R	1417383_at	*Entpd5*	ectonucleoside triphosphate diphosphohydrolase 5	Chr12: 84.375162	11.256	45	9.2	ChrX: 47.328644	0.182
421 ☐	MA_M2M_0706_R	1442406_at	*Mcart1*	mitochondrial carrier triple repeat 1	Chr4: 45.406475	7.885	45	6.7	Chr2: 168.436299	0.109
422 ☐	MA_M2M_0706_R	1454611_a_at	*Calm1*	calmodulin 1; proximal to mid 3'UTR	Chr12: 100.207086	13.046	45	12.8	ChrX: 102.126656	-0.143
423 ☐	MA_M2M_0706_R	1423940_at	*Yif1*	Yip1 interacting factor homolog; exons 1, 2, 3, 4, and 5	Chr19: 5.090017	11.972	45	18.4	ChrX: 47.328644	0.108

424 ☐	MA_M2M_0706_R	1460336_at	*Ppargc1a*	peroxisome proliferative activated receptor, gamma, coactivator 1 alpha; last three exons including 3' UTR	Chr5: 51.458065	10.285	45	11.2	Chr15: 97.657322	-0.269
425 ☐	MA_M2M_0706_R	1449369_at	*Tmprss2*	transmembrane protease, serine 2; exons 10, 11, and 12	Chr16: 97.567022	9.278	45	9.0	Chr2: 15.000000	0.140
426 ☐	MA_M2M_0706_R	1426008_a_at	*Slc7a2*	solute carrier family 7 (cationic amino acid transporter, y+ system), member 2	Chr8: 40.916661	6.702	45	6.5	ChrX: 164.51316 7	-0.046
427 ☐	MA_M2M_0706_R	1453195_at	*Snapc4*	small nuclear RNA activating complex, polypeptide 4	Chr2: 26.382936	9.293	45	14.0	Chr15: 91.405127	0.121
428 ☐	MA_M2M_0706_R	1460406_at	*Trpc1*	transient receptor potential cation channel, subfamily C, member 1	Chr9: 95.752943	11.106	45	9.8	Chr11: 99.734855	0.134
429 ☐	MA_M2M_0706_R	1423296_at	*Psmd8*	proteasome (prosome, macropain) 26S subunit, non-ATPase, 8; exons 3, 4, and 5	Chr7: 29.176073	13.070	45	13.5	Chr1: 184.32310 5	0.122
430 ☐	MA_M2M_0706_R	1424392_at	*Adhfe1*	alcohol dehydrogenase, iron containing, 1	Chr1: 9.566898	13.553	45	11.5	ChrX: 102.41452 6	0.133
431 ☐	MA_M2M_0706_R	1417559_at	*Sfxn1*	sideroflexin 1 (flexed tail, tricarboxylate carrier protein, iron homeostasis)	Chr13: 54.093053	12.597	45	7.7	Chr18: 76.826488	0.184
432 ☐	MA_M2M_0706_R	1453851_a_at	*Gadd45g*	growth arrest and DNA-damage-inducible 45 gamma	Chr13: 51.847877	9.356	45	13.0	Chr5: 77.779809	-0.305
433 ☐	MA_M2M_0706_R	1419975_at	*Scp2*	sterol carrier protein 2, liver; mid-distal 3' UTR	Chr4: 108.043986	10.166	45	10.2	ChrX: 50.730573	-0.297
434 ☐	MA_M2M_0706_R	1426990_at	*Cubn*	cubilin (intrinsic factor-cobalamin receptor)	Chr2: 13.278386	13.765	45	17.9	ChrX: 102.12665 6	0.214
435 ☐	MA_M2M_0706_R	1415674_a_at	*Trappc4*	trafficking protein particle complex 4; exons 3 and 4	Chr9: 44.404406	10.457	45	17.7	Chr7: 24.937915	-0.170

436 ☐	MA_M2M_0706_R	1426456_a_at	*Miz1*	Msx-interacting-zinc finger; exons 8, 9, 10, and 11	Chr18: 77.133220	9.441	45	18.9	Chr18: 78.806816	0.147
437 ☐	MA_M2M_0706_R	1425020_at	*Ubxd2a*	UBX domain protein 2A (p97 adaptor protein); far 3' UTR	Chr12: 4.877133	13.142	45	16.4	Chr11: 62.251912	0.118
438 ☐	MA_M2M_0706_R	1427705_a_at	*Nfkb1*	nuclear factor of kappa light chain gene enhancer in B-cells 1, p105; mid proximal 3' UTR	Chr3: 135.584947	9.357	45	14.9	Chr1: 178.15668 0	0.085
439 ☐	MA_M2M_0706_R	1459417_at	*Zfp609*	zinc finger protein 609	Chr9: 65.714936	7.320	45	9.9	Chr8: 24.382366	-0.043
440 ☐	MA_M2M_0706_R	1451747_a_at	*Apg12*	autophagy related 12; mid distal 3' UTR	Chr18: 46.732550	10.396	45	15.2	Chr2: 134.462008	0.134
441 ☐	MA_M2M_0706_R	1423906_at	*Hsbp1*	heat shock factor binding protein 1; mid 3' UTR	Chr8: 119.348382	10.672	45	12.6	Chr12: 76.378311	0.113
442 ☐	MA_M2M_0706_R	1458525_at	*App*	amyloid beta (A4) precursor protein; intron 1	Chr16: 85.155282	9.076	45	13.1	Chr13: 119.390734	-0.185
443 ☐	MA_M2M_0706_R	1426656_at	*4930504E06Rik*	RIKEN cDNA 4930504E06 gene	Chr3: 95.295369	11.232	45	15.5	Chr7: 31.360167	-0.131
444 ☐	MA_M2M_0706_R	1433166_at	*4930485E13Rik*	RIKEN cDNA 4930485E13 gene	ChrUn: 1.000000	6.878	45	10.7	Chr5: 85.578195	-0.037
445 ☐	MA_M2M_0706_R	1452246_at	*Ostf1*	osteoclast stimulating factor 1	Chr19: 18.584675	10.691	45	28.3	Chr19: 18.172211	0.253
446 ☐	MA_M2M_0706_R	1434696_at	*BC037708*	cDNA sequence BC037708	Chr2: 163.045339	9.762	45	14.4	Chr6: 24.108037	0.140
447 ☐	MA_M2M_0706_R	1451149_at	*Pgm2*	phosphoglucomutase 2; last exon and 3' UTR	Chr4: 99.986729	11.267	45	10.5	Chr9: 99.750326	0.105
448 ☐	MA_M2M_0706_R	1437267_x_at	*Hnrnph1*	heterogeneous nuclear ribonucleoprotein H1; distal 3' UTR	Chr11: 50.386367	7.915	45	9.6	ChrX: 42.224690	-0.092
449 ☐	MA_M2M_0706_R	1436502_at	*Mtus1*	mitochondrial tumor suppressor 1; last	Chr8: 40.993209	8.455	45	9.9	Chr11:	-0.154

				exon and proximal 3' UTR					73.268056	
450 ☐	MA_M2M_0706_R	1416096_at	*AI413782*	protein LOC63894; mid 3' UTR	Chr12:87.239058	10.360	45	12.8	Chr11:42.818374	-0.104
451 ☐	MA_M2M_0706_R	1446538_at	*Kpna3*	karyopherin (importin) alpha 3; intron (from EST AK083505)	Chr14:61.405964	7.335	45	9.7	Chr6:24.108037	-0.075
452 ☐	MA_M2M_0706_R	1448200_at	*Tcn2*	transcobalamin 2; last 2 exons and 3' UTR	Chr11:3.917372	16.251	45	15.8	Chr15:91.405127	0.127
453 ☐	MA_M2M_0706_R	1459150_at	*Lrch1*	leucine-rich repeats and calponin homology (CH) domain containing 1; first intron	Chr14:74.875130	6.007	45	10.2	Chr3:54.220588	-0.039
454 ☐	MA_M2M_0706_R	1455571_x_at	*Calm1*	calmodulin 1; mid proximal 3' UTR	Chr12:100.207059	13.160	45	9.2	Chr13:72.874773	-0.113
455 ☐	MA_M2M_0706_R	1426593_a_at	*Fbxo22*	F-box only protein 22	Chr9:55.224277	12.590	45	11.6	Chr11:62.251912	0.123
456 ☐	MA_M2M_0706_R	1448422_at	*Tmed4*	transmembrane emp24 protein transport domain containing 4	Chr11:6.270832	13.128	45	9.2	Chr17:47.728777	-0.080
457 ☐	MA_M2M_0706_R	1430339_at	*2610021K21Rik*	RIKEN cDNA 2610021K21 gene	Chr12:99.717612	5.746	45	11.8	Chr4:88.045738	0.033
458 ☐	MA_M2M_0706_R	1452504_s_at	*Ctbs*	chitobiase, di-N-acetyl-	Chr3:146.458794	10.421	45	7.6	Chr9:98.579310	0.193
459 ☐	MA_M2M_0706_R	1423670_a_at	*Srpr*	signal recognition particle receptor (ER docking protein); mid to distal 3' UTR	Chr9:35.216428	12.399	45	10.3	ChrX:102.42480 4	0.078
460 ☐	MA_M2M_0706_R	1428428_at	*Abhd11*	abhydrolase domain containing 11 (Williams syndrome); last 3 exons and 3' UTR	Chr5:135.011355	11.319	45	7.7	Chr7:40.421635	-0.083
461 ☐	MA_M2M_0706_R	1446356_at	*Ppp2r5e*	protein phosphatase 2, regulatory subunit B (B56), epsilon isoform	Chr12:75.486681	6.896	45	9.8	Chr12:106.833654	-0.045

462 ☐	MA_M2M_0706_R	1453307_a_at	*Anapc5*	anaphase-promoting complex subunit 5; exon 7	Chr5: 10.236971	11.245	45	9.8	Chr5: 135.879195	0.130
463 ☐	MA_M2M_0706_R	1427885_at	*Pold4*	polymerase (DNA-directed), delta 4	Chr19: 4.232508	8.774	45	11.4	Chr11: 64.680151	-0.104
464 ☐	MA_M2M_0706_R	1449862_a_at	*Pi4k2b*	phosphatidylinositol 4-kinase type 2 beta (neuronal calcium sensor-1 effector); exons 8, 9, 11, and 3' UTR	Chr5: 52.760874	7.310	45	9.3	Chr9: 15.889229	-0.086
465 ☐	MA_M2M_0706_R	1416933_at	*Por*	P450 (cytochrome) oxidoreductase (endoplasmic reticulum, NADPH-cytochrome P450 reductase); last two exons and 3' UTR	Chr5: 135.734627	12.861	45	9.6	Chr7: 73.746984	0.141
466 ☐	MA_M2M_0706_R	1452193_a_at	*Wasl*	Wiskott-Aldrich syndrome-like; distal 3' UTR	Chr6: 24.613837	12.234	45	15.2	Chr11: 62.251912	0.162
467 ☐	MA_M2M_0706_R	1428433_at	*Hipk2*	homeodomain interacting protein kinase 2; distal 3' UTR	Chr6: 38.687549	11.843	45	9.1	Chr13: 119.390734	-0.135
468 ☐	MA_M2M_0706_R	1455734_at	*Crbn*	cereblon	Chr6: 106.778989	8.809	45	11.2	Chr18: 82.106750	0.118
469 ☐	MA_M2M_0706_R	1451765_a_at	*Entpd5*	ectonucleoside triphosphate diphosphohydrolase 5	Chr12: 84.376957	11.326	45	11.0	Chr10: 33.257865	-0.169
470 ☐	MA_M2M_0706_R	1444140_at	*Pum1*	pumilio 1 (PUF family RNA-binding protein); intron 2 (from EST AK084282)	Chr4: 130.679429	6.753	45	7.8	Chr2: 168.436299	0.088
471 ☐	MA_M2M_0706_R	1433355_at	*2610021J01Rik*	RIKEN cDNA 2610021J01 gene; possible antisense in Hmgb2 (poor probe specificity)	--	6.282	45	13.1	ChrX: 115.338127	-0.038
472 ☐	MA_M2M_0706_R	1433747_at	*Lnpep*	leucyl/cystinyl aminopeptidase; distal 3' UTR (non-canonical)	Chr17: 17.521436	10.238	45	13.5	Chr13: 117.604982	-0.162

473 ☐	MA_M2M_0706_R	1416340_a_at	*Man2b1*	mannosidase 2, alpha B1; last four exons and 3' UTR	Chr8: 85.096414	13.168	45	14.2	ChrX: 102.12665 6	0.132
474 ☐	MA_M2M_0706_R	1424780_a_at	*Reep3*	receptor accessory protein 3; distal 3' UTR	Chr10: 67.011532	11.252	45	8.7	ChrX: 69.232896	-0.147
475 ☐	MA_M2M_0706_R	1420990_at	*Chd1*	chromodomain helicase DNA binding protein 1; last two exon and proximal 3'-UTR	Chr17: 15.768611	8.886	45	14.4	Chr18: 80.000000	0.171
476 ☐	MA_M2M_0706_R	1423164_at	*Tm7sf1*	transmembrane 7 superfamily member 1	Chr13: 13.358087	9.967	45	15.3	Chr9: 98.579310	0.149
477 ☐	MA_M2M_0706_R	1425025_at	*Tmem106a*	transmembrane protein 106A; last two exons and proximal 3' UTR	Chr11: 101.589980	12.294	45	14.4	Chr15: 93.021696	0.200
478 ☐	MA_M2M_0706_R	1451407_at	*Jam4*	junction adhesion molecule 4	Chr16: 96.403442	10.903	45	14.1	Chr15: 97.760642	0.211
479 ☐	MA_M2M_0706_R	1437496_at	*Acad10*	acyl-Coenzyme A dehydrogenase family member 10; last exon and proximal 3' UTR	Chr5: 121.598449	9.307	45	10.5	Chr13: 119.390734	0.082
480 ☐	MA_M2M_0706_R	1436184_at	*Uckl1*	uridine-cytidine kinase 1-like 1	Chr2: 181.582303	8.569	45	10.7	Chr7: 24.937915	-0.120
481 ☐	MA_M2M_0706_R	1417105_at	*Trapp2cl*	trafficking protein particle complex 2-like; last four exons and proximal 3' UTR	Chr8: 122.613103	11.957	45	18.8	Chr15: 91.405127	0.166
482 ☐	MA_M2M_0706_R	1448309_at	*Ap3m1*	adaptor-related protein complex 3, mu 1 subunit; last four exons and proximal 3' UTR	Chr14: 21.036673	8.958	45	11.2	Chr8: 123.289925	0.176
483 ☐	MA_M2M_0706_R	1434290_at	*Gtdc1*	glycosyltransferase-like domain containing 1; mid to distal 3'UTR	Chr2: 44.564482	7.738	45	16.2	Chr8: 123.289925	0.110
484 ☐	MA_M2M_0706_R	1430971_a_at	*Aqr*	aquarius	Chr2: 114.158933	5.981	45	11.8	Chr11: 24.905498	-0.059
485 ☐	MA_M2M_0706_R	1418365_at	*Ctsh*	cathepsin H	Chr9: 90.074884	13.608	45	10.8	Chr8: 76.227149	0.115

486	MA_M2M_0706_R	1424007_at	*Gdf10*	growth differentiation factor 10, bone morphogenetic protein 3B (bone inducing, Bergmann glial cell marker)	Chr14:33.934703	8.235	45	12.8	Chr11:50.383261	0.153
487	MA_M2M_0706_R	1417442_a_at	*Pex3*	peroxisomal biogenesis factor 3	Chr10:13.532375	9.440	45	10.1	Chr13:117.60498 2	0.155
488	MA_M2M_0706_R	1416170_at	*Trap1*	TNF receptor-associated protein 1	Chr16:4.040139	12.933	45	10.2	Chr4:102.85102 0	0.100
489	MA_M2M_0706_R	1424657_at	*Taok1*	TAO kinase 1; last exon and proximal 3' UTR	Chr11:77.537681	10.464	45	13.1	Chr5:27.285288	0.228
490	MA_M2M_0706_R	1439797_at	*Ppard*	peroxisome proliferator activator receptor delta; distal 3' UTR	Chr17:28.301070	8.829	45	8.9	Chr2:118.88103 2	0.390
491	MA_M2M_0706_R	1441253_at	*Rfx3*	regulatory factor X, 3 (dentate gyrus expression signature, influences HLA class II expression); intron 1	Chr19:27.992455	8.076	45	9.8	Chr3:27.192039	0.112
492	MA_M2M_0706_R	1441312_at	*Cnnm1*	cyclin M1; distal 3' UTR	Chr19:43.496088	6.919	45	13.1	Chr1:193.48124 2	0.082
493	MA_M2M_0706_R	1421613_at	*H2afy2*	H2A histone family, member Y2	Chr15:62.217666	5.902	45	9.6	ChrX:115.33812 7	0.050
494	MA_M2M_0706_R	1434453_at	*BC053071*	hypothetical protein MGC62420	Chr14:24.448840	8.502	45	14.0	Chr7:24.937915	-0.144
495	MA_M2M_0706_R	1431394_a_at	*Lrrk2*	leucine-rich repeat kinase 2 (Parkinson disease 8, autosomal dominant); last exons and proximal 3' UTR	Chr15:91.812217	11.222	45	13.9	Chr6:36.747330	0.209
496	MA_M2M_0706_R	1425022_at	*Usp3*	ubiquitin specific protease 3; last 4 exons and proximal 3' UTR	Chr9:66.518371	10.102	45	12.8	Chr12:12.602500	0.149

497 ☐	MA_M2M_0706_R	1431216_s_at	*Dnajc6*	DnaJ (Hsp40) homolog, subfamily C, member 6; proximal 3' UTR	Chr4: 101.640739	6.677	45	13.1	Chr8: 88.711457	0.091
498 ☐	MA_M2M_0706_R	1434050_at	*AI315068*	expressed sequence AI315068	Chr16: 21.640728	9.564	45	10.2	Chr10: 27.579570	-0.184
499 ☐	MA_M2M_0706_R	1457949_at	*Nt5c2*	5'-nucleotidase, cytosolic II; putative exon	Chr19: 46.885497	7.837	45	20.5	Chr11: 62.251912	-0.122
500 ☐	MA_M2M_0706_R	1456669_at	*AK044157*	fat expressed non-coding sequence AK044157; 3' end of sequence (antisense in last intron of Ptgis)	Chr2: 166.883175	6.832	45	7.4	Chr5: 106.818409	0.075
501 ☐	MA_M2M_0706_R	1445747_at	*Osx*	ESTs	ChrX: 162.735296	6.628	45	12.8	ChrX: 139.352577	-0.067
502 ☐	MA_M2M_0706_R	1425997_a_at	*Pign*	phosphatidylinositol glycan, class N	Chr1: 105.557466	7.725	45	8.5	Chr16: 37.101961	0.064
503 ☐	MA_M2M_0706_R	1426410_at	*Pdk3*	pyruvate dehydrogenase kinase, isoenzyme 3; exons 9, 10, and 11 and proximal 3' UTR (transQTL on Chr 1 in BXD hippocampus data)	ChrX: 93.768916	10.872	45	11.4	Chr13: 45.202912	-0.206
504 ☐	MA_M2M_0706_R	1439254_at	*Akap13*	A kinase (PRKA) anchor protein 13	Chr7: 75.609839	6.396	45	7.7	Chr7: 6.011490	0.132
505 ☐	MA_M2M_0706_R	1417844_at	*Vdrip*	vitamin D receptor interacting protein	Chr14: 73.517967	8.799	45	11.1	Chr17: 75.881027	0.117
506 ☐	MA_M2M_0706_R	1430682_at	*4930555I21Rik*	RIKEN cDNA 4930555I21 gene	Chr4: 135.537270	6.665	45	11.1	Chr16: 11.886422	-0.043
507 ☐	MA_M2M_0706_R	1428028_at	*Gt4-1*	gene trap insertion site 4-1	Chr16: 13.326470	7.529	45	10.3	Chr2: 176.000000	0.082
508 ☐	MA_M2M_0706_R	1428380_at	*Apr3*	apoptosis-related protein 3 (human chromosome 2 open	Chr5: 31.052528	12.591	45	11.0	Chr5: 127.781724	0.121

				reading frame 28); last three exons						
509 ☐	MA_M2M_0706_R	1421848_at	*Slc22a5*	solute carrier family 22 (organic cation transporter), member 5; last 4 exons and proximal 3' UTR	Chr11: 53.865710	11.860	45	7.4	Chr2: 159.44754 9	0.192
510 ☐	MA_M2M_0706_R	1454609_x_at	*6430527G18Rik*	RIKEN cDNA 6430527G18 gene	Chr12: 86.881028	9.633	45	8.5	Chr13: 53.477510	-0.112
511 ☐	MA_M2M_0706_R	1452920_a_at	*Ppil2*	peptidylprolyl isomerase (cyclophilin)-like 2; 6 exons	Chr16: 17.088829	10.168	45	8.3	Chr1: 98.793301	0.110
512 ☐	MA_M2M_0706_R	1419173_at	*Acy1*	aminoacylase 1; last three exons and mid 3' UTR	Chr9: 106.433027	12.598	45	43.0	Chr9: 105.77438 1	0.579
513 ☐	MA_M2M_0706_R	1451622_at	*Lmbrd1*	LMBR1 domain containing 1; last three exons and proximal 3' UTR	Chr1: 24.748801	11.389	45	10.5	Chr11: 7.130166	0.099
514 ☐	MA_M2M_0706_R	1441208_at	*Hdhd2*	haloacid dehalogenase-like hydrolase domain containing; putative deep 3' UTR	Chr18: 76.973281	8.228	45	13.8	Chr11: 73.268056	-0.101
515 ☐	MA_M2M_0706_R	1420509_at	*2810036K01Rik*	RIKEN cDNA 2810036E22 gene	Chr18: 52.488921	8.640	45	13.5	Chr12: 12.791300	0.109
516 ☐	MA_M2M_0706_R	1441220_at	*Magi2*	membrane associated guanylate kinase, WW and PDZ domain containing 2; putative intron	Chr5: 19.786584	8.027	45	14.9	Chr3: 27.192039	0.132
517 ☐	MA_M2M_0706_R	1456668_at	*Zcchc11*	zinc finger, CCHC domain containing 11; intron (from EST AK033820 AND 9230115F04Rik)	Chr4: 108.538206	6.276	45	16.8	Chr2: 35.013636	-0.045
518 ☐	MA_M2M_0706_R	1451276_at	*Uhrf1bp1l*	UHRF1 (ICBP90) binding protein 1-like	Chr10: 89.819273	10.926	45	7.9	Chr2: 109.78269 4	-0.116
519 ☐	MA_M2M_0706_R	1420618_at	*Cpeb4*	cytoplasmic polyadenylation element binding protein 4; distal 3' UTR	Chr11: 31.935120	13.126	45	10.1	Chr3: 113.08796 3	0.187

520 ☐	MA_M2M_0706_R	1432898_at	*4930544L18Rik*	RIKEN cDNA 4930544L18 gene	Chr14: 118.665020	6.126	45	13.6	Chr7: 71.410581	0.050
521 ☐	MA_M2M_0706_R	1434189_at	*Stag1*	stromal antigen 1; distal 3' UTR	Chr9: 100.958100	9.193	45	10.9	Chr8: 3.500000	0.086
522 ☐	MA_M2M_0706_R	1443162_at	*2310031L18Rik*	RIKEN cDNA 2310031L18 gene	Chr4: 57.213165	6.624	45	8.3	Chr15: 93.021696	-0.065

Supplemental Figure S1. Expression levels of Adam17 in kidney between female and male

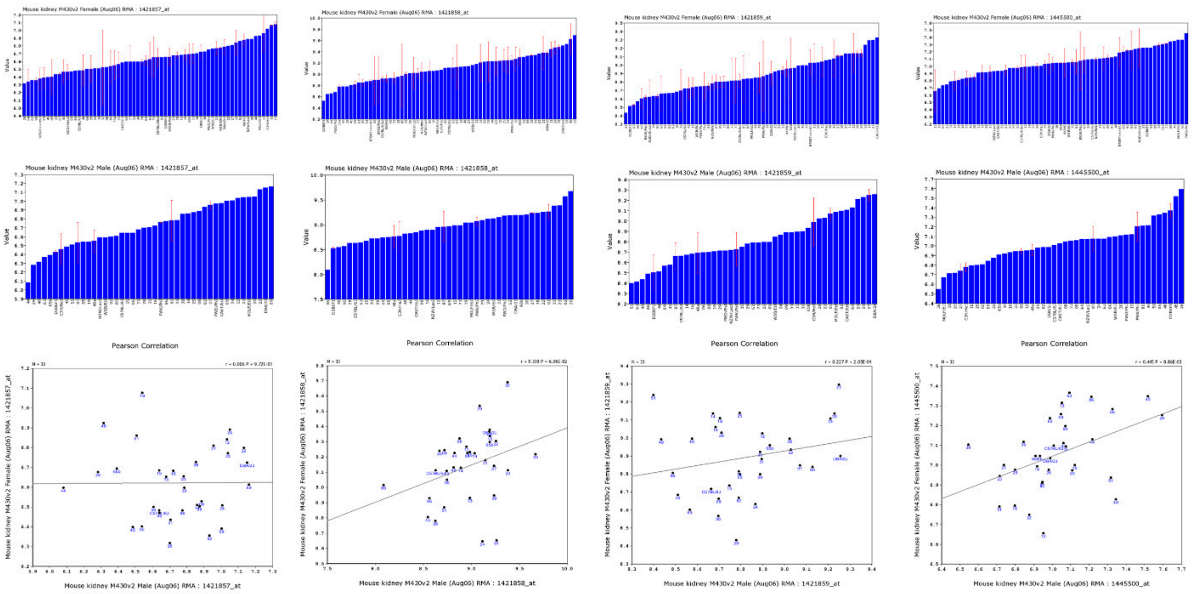

Supplemental Figure S2. Expression levels of Tmprss2 in kidney between female and male

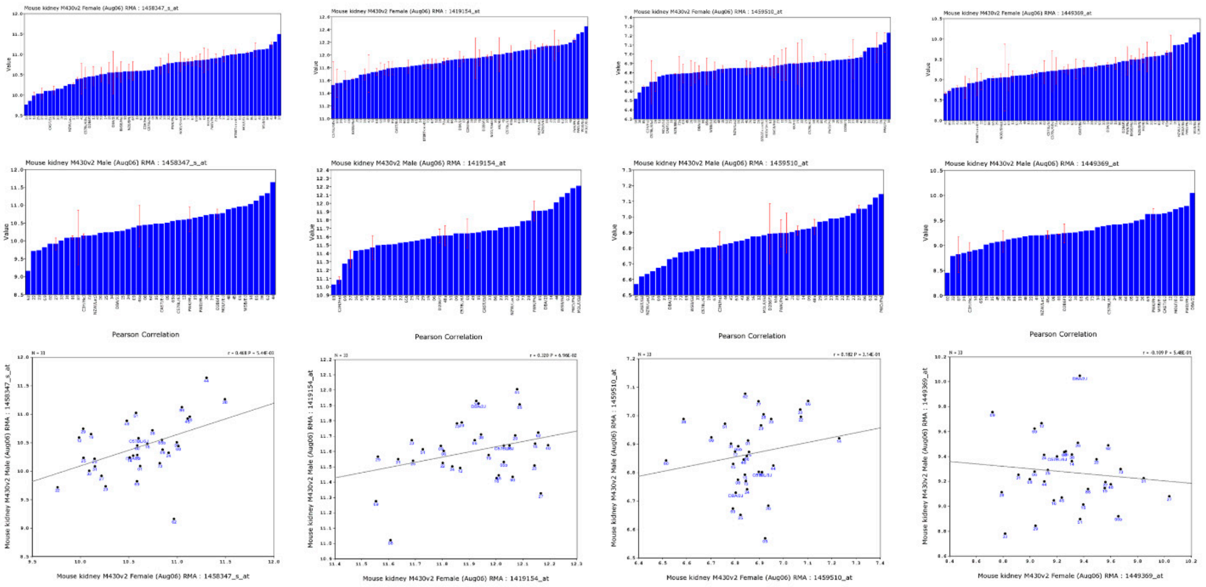

Supplemental Figure S3. Expression levels of Mcam (CD146) in kidney between female and male

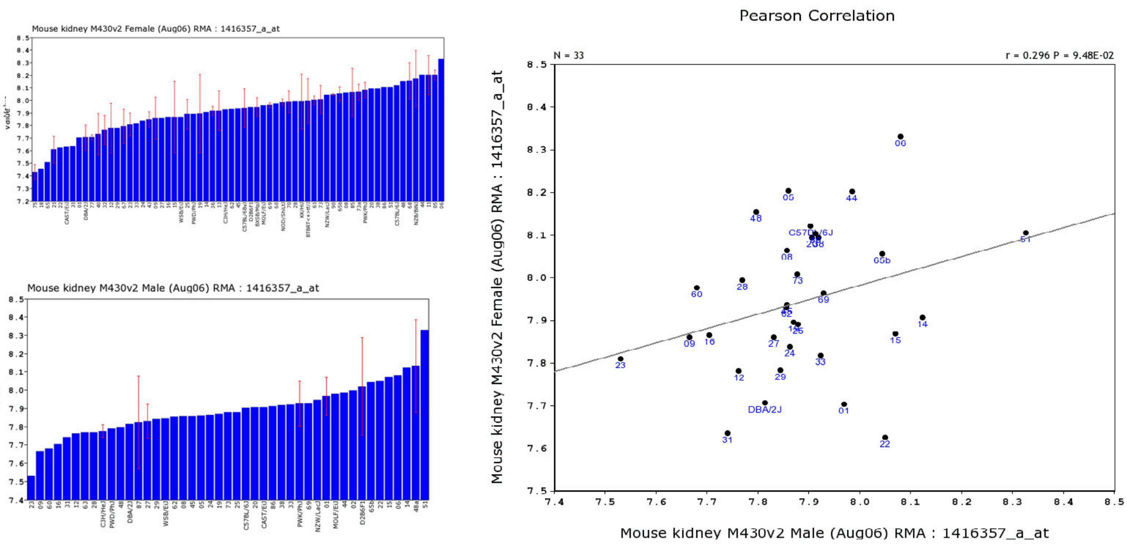

Supplement: Supplementary file 1 [file jpm-12-01190-s001.zip › jpm-1710303-supplementary.pdf]
